# Novel Ring Systems: Spiro[Cycloalkane] Derivatives of Triazolo- and Tetrazolo-Pyridazines

**DOI:** 10.3390/molecules26082140

**Published:** 2021-04-08

**Authors:** Csilla Sepsey Für, Gergő Riszter, Áron SzigetvárI, Miklós Dékány, György Keglevich, László HazaI, Hedvig BölcskeI

**Affiliations:** 1 Department of Organic Chemistry and Technology, Budapest University of Technology and Economics, H-1111 Budapest, Hungary; sepsey.fur.csilla@vbk.bme.hu (C.S.F.); riszter93@gmail.com (G.R.); keglevich.gyorgy@vbk.bme.hu (G.K.); hazai.laszlo@vbk.bme.hu (L.H.); 2 Gedeon Richter Plc. Budapest X, Gyömrői út 19-21, 10 P.O. Box 27, H-1475 Budapest, Hungary; szigetvaria@richter.hu (Á.S.); m.dekany@richter.hu (M.D.)

**Keywords:** *N*-heterocycles, pyridazine derivatives, thionation, diazotization, triazolo-pyridazines, tetrazolo-pyridazines

## Abstract

In orderto synthesize new pyridazine derivatives anellated with different nitrogen heterocyclic moieties, spiro[cycloalkane]pyridazinones were transformed into the corresponding thioxo derivatives via a reaction with phosphorus pentasulfide. The reaction of the formed 2,3-diazaspiro[5.5]undec-3-ene-1-thiones with hydrazine provided the corresponding 1-hydrazono-2,3-diazaspiro[5.5]undec-3-ene, whose diazotization led to the desired spiro[cyclohexane-1,8′-tetrazolo[1,5-b]pyridazines. The reaction of dihydropyridazinethiones with benzhydrazide afforded the corresponding 7*H*-spiro[[1,2,4]triazolo[4,3-b]pyridazin-8,1′-cyclohexanes]. As a result of our work, seven new pyridazinethione intermediates were prepared, which served as starting materials for the synthesis of two kinds of new ring systems: tetrazolo-pyridazines and triazolo-pyridazines. The six new annulated derivatives were characterized by physicochemical parameters. The new *N*-heterocycles are valuable members of the large family of pyridazines.

## 1. Introduction

**Figure 1:**
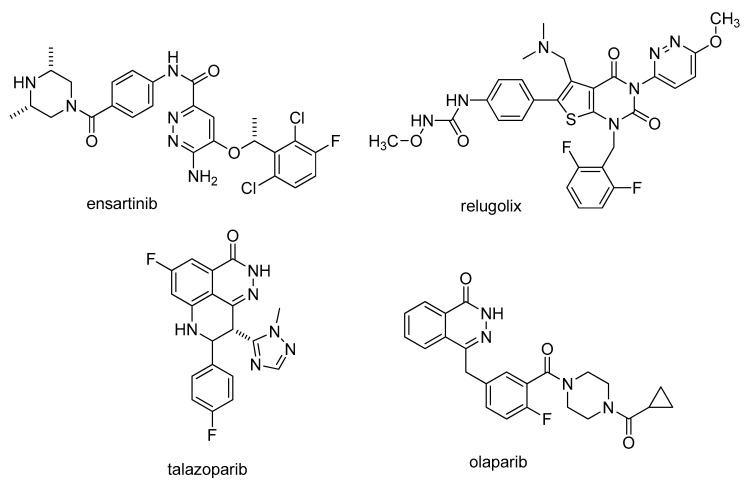
Pyridazine derivatives under clinical development: ensartinib, relugolix, talazoparib, and olaparib.

Pyridazine derivatives are representative members of *N*-heterocycles [1,2]. A large number of pyridazines show various types of bioactivity [3,4,5,6,7]. For example, sulfamethoxypyridazine is an old sulfonamide antibacterial drug [8], while hydralazine (Apresoline) is a well-known antihypertensive agent [9]. The calcium sensitizer levosimendan is useful in congestive heart failure [10]. The monoamine oxidase (MAO) inhibitor is induced by minaprine [11,12] and the tricyclic pipofezine (Azaphen) [13] are well-known antidepressants. The aldose-reductase inhibitor zopolrestat was developed for treating diabetic neuropathy [14]. The pyridazines under development are also noteworthy. The anaplastic lymphoma kinase (ALK) inhibitor ensartinib is in Phase II clinical trials [15]. The gonadotropin releasing hormone (GnRH) receptor antagonist relugolix (Relumina) was approved for the treatment of uterine fibroids [16]. Talazoparib (Talzenna) is a poly adenosine diphosphate (ADP) ribose polymerase (PARP) inhibitor, which was developed for treating advanced breast cancer [17]. Talazoparib has a similar phthalazin-1(*2H*)-one motif with that found in the other PARP inhibitor olaparib (Lynparza) [18] (Figure 1).

The spirocycles have recently gained more attention, as they usually have a high Fsp^3^ character, and the three-dimensionality is better in the case of flat aromatic rings. The use of spiro building blocks better optimizes physicochemical parameters, structural novelty, and higher patentability. There are well-known examples among the spiro compounds, e.g., the anxiolytic agent buspirone, the angiotensin receptor blocker irbesartan, the antifungal natural product griseofulvin, or the spironolactone with sterane skeleton. The к opioid agonist enadoline seemed to be the most potent к selective analgesic. Most of the recently published spiro compounds contain five and six membered rings, but there are examples for 4-membered or 7-membered spirocyclic systems too [19,20,21,22,23].

**Scheme 1:**
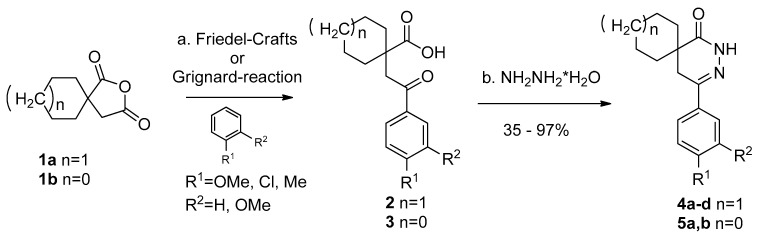
Synthesis of spiro[cycloalkane]pyridazinones (**4a–d** and **5a,b**) [24,25]; (a) **1a,b** (1.0 mmol), Friedel–Crafts reaction: AlCl_3_ (3.0 mmol), ArH derivative (3.0 mmol), 0 °C (30 min) -> room temperature (RT) (24 h); Grignardreaction: Grignard reagent (1.0 mmol), 0 °C (30 min) -> RT (24 h); (b) **2,3** (0.80 mmol), hydrazine hydrate (2.40 mmol), EtOH, reflux, (4 h); (c) **4a–d**, **5a,b** isolated yields after thin layer chromatography (TLC) purification: 35–97% [24,25].

Realizing that the use of the nitrogen containing heterocycles and the spiro motif may be advantageous, a few spiro[cycloalkane]pyridazinone derivatives were synthesized by us (Scheme 1) [24,25]. Depending on the substituents, the starting anhydrides (2-oxaspiro[4.4]nonane-1,3-dione (**1a**) and 2-oxaspiro[4.5]-1,3-dione (**1b**)) were converted to the desired keto-carboxylic acids (**2** and **3**) either by the Friedel–Crafts reaction or the Grignard reaction. This was followed by the ring closure with hydrazine hydrate in refluxing ethanol to give the corresponding pyridazinone derivatives (**4a–d**, **4e** and **5a**,**b**) in high yields [24,25].

In this work, we described the use of spiro[cycloalkane]pyridazinone derivatives (**4a–d**, **5a,b**) in the synthesis of new triazolo-pyridazine and tetrazolo-pyridazine derivatives via the corresponding thioxo intermediates.

## 2. Results and Discussion

Our plan was to synthesize derivatives, where the pyridazine ring was fused with another *N*-heterocyclic moiety (**9** and **10**). 

### 2.1. Synthesis of Pyridazinethiones

**Scheme 2:**
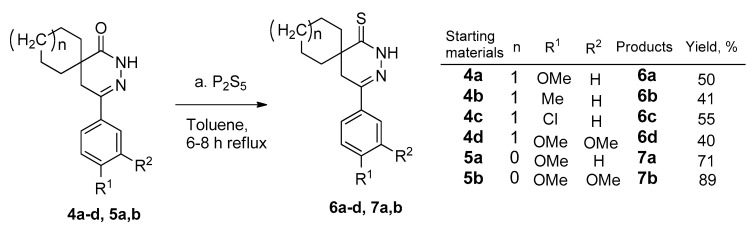
Thionation reaction of the spiro[cycloalkane]pyridazinones (**4a–d** and **5a,b**); (a) **4a–d**, **5a,b** (1.0 mmol), P_2_S_5_ (3.0 mmol), PhMe, reflux, (6–8 h); (b) **6a–d**, **7a,b** isolated yields after TLC purification: 40–89%.

Phosphorus pentasulfide or the Lawesson reagent may be used for the thionation of an amide function [26]. The earlier described spiro[cycloalkane]pyridazinones (**4a–d** and **5a,b**) reacted with phosphorus pentasulfide in the refluxing toluene. According to thin layer chromatography (TLC), the starting material was consumed after 6–8 h. After the workup comprising extraction, concentration, and preparative TLC purification, the products (**6a–d** and **7a,b**) were isolated in yields of 40–89% (Scheme 2). The new compounds (**6a–d** and **7a,b**) were characterized by ^1^H and ^13^C nuclear magnetic resonance spectroscopy (NMR), as well as high resolution mass spectrometry (HRMS).

**Scheme 3:**
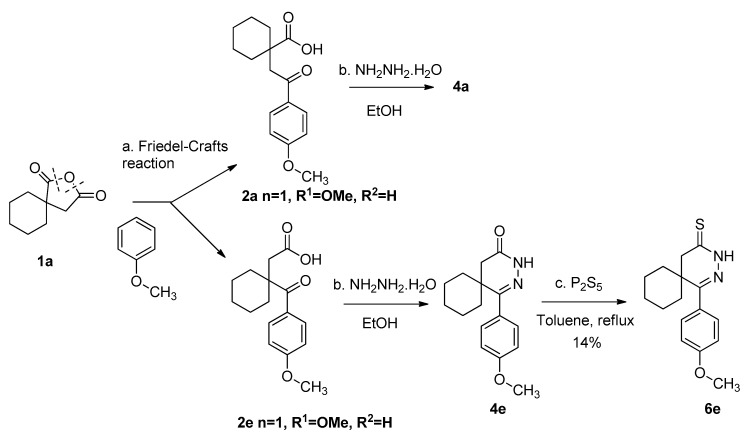
Preparation of 1-(4-methoxyphenyl)-2,3-diazaspiro[5.5]undec-1-ene-4-thione (**6e**): (a) **1a** (1.0 mmol), Friedel–Crafts reaction: AlCl_3_ (3.0 mmol), anizole (3.0 mmol), 0 °C (30 min) -> RT (24 h); (b) **2a,e** (0.80 mmol), hydrazine hydrate (2.40 mmol), EtOH, reflux, (4 h); (c) **2a**,**e** (1.0 mmol), P_2_S_5_ (3.0 mmol), PhMe, reflux, (6–8 h); (d) **2e** isolated yield after TLC purification: 14%.

During the preparation of the *p*-methoxyphenyl[spirocyclohexane]pyridazinone (**4a**), the isomeric pyridazinone derivative (**4e**) was isolated as a byproduct. The formation of the two isomers was explained earlier [24]. The thionation reaction with phosphorus pentasulfide led to the corresponding pyridazinethione (**6e**) in a yield of 14% (Scheme 3). 

### 2.2. Preparing the Tetrazole Derivatives through Hydrazones

**Scheme 4:**
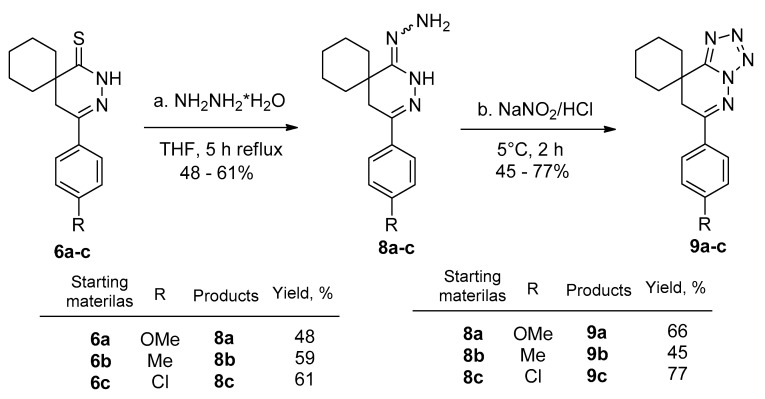
Synthesis of the hydrazono-pyridazinone (**8a–c**) and tetrazole derivatives (**9a–c**) (a) **6a–c** (0.40 mmol), hydrazine monohydrate (1.20 mmol), tetrahydrofuran (THF), reflux, (5 h); (b) **8a–c** (0.34 mmol), NaNO_2_ (0.51 mmol), 0.5 N HCl, 5 °C, (2 h); (c) **8a–c** isolated yields after TLC purification: 48–61%; (d) **9a–c** isolated yields after TLC purification: 45–77%.

After reacting the thioxo derivatives **6a–c** with hydrazine hydrate, the corresponding hydrazones **8a–c** were isolated in 48–61% yields. The 4-(phenyl)-2,3-diazaspiro[5.5]undec-3-ene-1-thiones (**6a–c**) and hydrazine hydrate in THF had to be stirred at reflux for 5 h. It was observed that compounds (**8a–c**) were unstable when standing at room temperature. After a week, they began decomposing. However, after preparation, intermediates **8a–c** were immediately reacted further in a diazotization reaction. The tetrazolo-pyridazinones (**9a–c**) were obtained in 45–77% yields (Scheme 4) after a preparative TLC purification. Compounds **8a–c** and **9a–c** were new and were characterized by ^1^H and ^13^C NMR, as well as HRMS. A similar method was applied in the sphere of benzodiazepines, where the extended conjugation helped the transformation [27]. In the case of spiro compounds **8a–c**, the reaction was new.

Table 1 summarizes the most important physicochemical parameters of the tetrazolo-pyridazinones (**9a–c**). All compounds revealed excellent Fsp^3^ character values: 0.5 ± 0.05. The logP (3.1–3.7) and topological polar surface area (TPSA) values (52.7–61.9) were satisfactory according to the Lipinski rules and Lovering studies [28,29,30,31,32]. For comparison, the launched pyridazinone derivatives sometimes have lower Fsp^3^ characters (e.g., hydralazine: 0, zopolrestate: 0.16, levosimendan: 0.21), while pipofezine and minaprine have higher Fsp^3^ characters (e.g., 0.38 and 0.41, respectively). The drugs under development showed similar Fsp^3^ values (i.e., talazoparib: 0.16, relugolix: 0.21, ensartinib: 0.27, olaparib: 0.34 (Table 2)).

### 2.3. Preparing the Triazole Derivatives from Thioxo Compounds with Acid Hydrazides

**Scheme 5:**
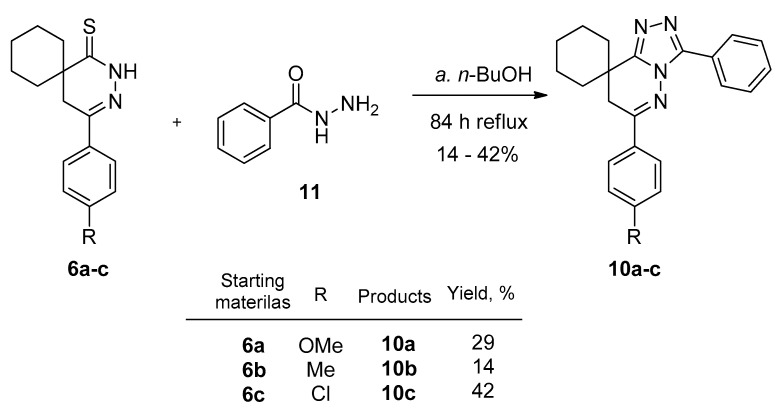
Reaction of thioxo-derivatives (**6a–c**) with benzoic acid hydrazide (**11**) (a) **6a–c** (0.73 mmol), **11** (2.20 mmol), *n*-BuOH, reflux, (84 h); (b) (c) **10a–c** isolated yields after TLC purification: 14–42%.

Our next purpose was to synthesize triazole derivatives starting from the thioxo compounds (**6a–c**). The thioxo compounds (**6a–c**) were refluxed with benzoic acid hydrazide (**11**) in *n*-butanol. After evaporation and chromatographic purification, the triazole-pyridazinone derivatives (**10a–c**) were isolated in yields of 14–42% (Scheme 5). This annulation technique was also applied in the sphere of benzodiazepines [33], where the extended aromatic conjugation may be advantageous for the reaction. Similarly to the preparation of tetrazoles, this reaction is new in case of spiro derivatives **10a–c**. After the preparative TLC chromatographic separation, the three new products (**10a–c**) were characterized by ^1^H and ^13^C NMR, as well as HRMS. Table 3 summarizes the physicochemical parameters of the triazolo-pyridazinones (**10a–c**). The introduction of an additional aromatic ring increased the log P and clogP values, and decreased the Fsp^3^ character.

## 3. Materials and Method

### 3.1. LC-MS Analysis, TLC, and Preparative TLC

Liquid chromatography-mass spectrometry (LC-MS) spectra were recorded on an Agilent 1100/ZQMSD (Santa Clara, CA, USA) instrument equipped with UV (220 nm and 254 nm) and MSAP^+^ and PL-ELS detectors. TLC was carried out using Kieselgel 60 F_254_ (Merck 1.05554.0001, Merck KGaA, Darmstadt, Germany). The analytical samples for NMR and HRMS studies were purified by preparative TLC using Kieselgel 60 F_254_ (Merck 1.07748.1000, Merck KGaA, Darmstadt, Germany) coated glass plates.

### 3.2. NMR Spectroscopy

NMR measurements were performed on a Varian VNMRS 400 MHz (Varian, Palo Alto, CA, USA) NMR spectrometer equipped with a ^15^N-^31^P(^1^H-^19^F) 5 mm one NMR room temperature probe, a Varian VNMRS 500 MHz NMR spectrometer equipped with a ^1^H (^13^C/^15^N) 5 mm pulsed field gradient (PFG) Triple Resonance ^13^C Enhanced Cold Probe, a Varian VNMRS 800 MHz NMR spectrometer equipped with a ^1^H(^13^C/^15^N) Triple Resonance ^13^C Enhanced Salt Tolerant Cold Probe (Varian, Inc., Palo Alto, CA, USA), a Bruker Avance III HDX 500 MHz NMR spectrometer equipped with a ^1^H (^13^C/^15^N) 5 mm TCI CryoProbe, and a Bruker Avance III HDX 800 MHz NMR spectrometer equipped with a ^1^H-^19^F(^13^C/^15^N) 5 mm TCI CryoProbe (Bruker Corporation, Billerica, MA, USA). ^1^H and ^13^C chemical shifts are given on the delta scale as parts per million (ppm) with tetramethylsilane (TMS) (^1^H, ^13^C) or dimethylsulfoxide-*d*_6_(^13^C) as the internal standard (0.00 ppm and 39.4 ppm, respectively). ^1^H-^1^H, direct ^1^H-^13^C, and long-range ^1^H-^13^C scalar spin-spin connectivity were established from 2D. correlation spectroscopy, total correlation spectroscopy, heteronuclear single quantum coherence and heteronuclear multiple bond correlation (COSY, TOCSY, HSQC, and HMBC) experiments. ^1^H-^1^H spatial proximities were determined using two-dimensional nuclear Overhauser effect spectroscopy or rotating-frame Overhauser effect spectroscopy (NOESY or ROESY) experiments. ^15^N Chemical shifts were referenced to nitromethane (0.0 ppm) and were obtained from ^1^H-^15^N HMBC measurements. All pulse sequences were applied using the standard spectrometer software package. All experiments were performed at 298 K. NMR spectra were processed using VnmrJ 2.2 Revision C (Varian, Inc., Palo Alto, CA, USA), Bruker TopSpin 3.5 pl 6 (Bruker Corporation, Billerica, MA, USA) and ACD/Spectrus Processor version 2017.1.3 (Advanced Chemistry Development, Inc., Toronto, ON, Canada).

### 3.3. Mass Spectrometry

HRMS and MS-MS analyses were performed on a Thermo Velos Pro Orbitrap Elite (Thermo Fisher Scientific, Bremen, Germany) system. The ionization method was ESI operated in a positive ion mode. The protonated molecular ion peaks were fragmented by collisison-induced dissociation (CID) at a normalized collision energy of 35%. For the CID experiment helium was used as the collision gas. The samples were dissolved in methanol. Data acquisition and analysis were accomplished with Xcalibur software version 2.0 (Thermo Fisher Scientific, Bremen, Germany).

### 3.4. Synthesis of the Starting Materials

The preparation of the starting pyridazinone derivatives (**4a–e** and **5a,b**) was described earlier [24,25].

### 3.5. General Procedure for the Synthesis of the Pyridazine-Thiones (***6a–e*** and ***7a,b***)

The pyridazinone derivatives (**4a–e** and **5a,b**) (1.0 mmol) were dissolved in toluene (60 mL) and phosphorus pentasulfide (0.67 g, 3.0 mmol) was added. The reaction mixture was stirred at reflux for 6–8 h. After the completion of the reaction, the mixture was washed with 5% NaHCO_3_ solution (80 mL), and then with distilled water (80 mL). The organic layer was dried over MgSO_4_ and then concentrated. The crude product was purified by preparative thin layer chromatography (eluent: heptane:dichloromethane:methanol/5:5:1) to give the thioxo derivatives as a yellow solids (**6a–e** and **7a,b**).

4-(4-Methoxyphenyl)-2,3-diazaspiro[5.5]undec-3-ene-1-thione (**6a**). Yield = 74%, R_f_(heptane:dichloromethane:methanol/5:5:1) = 0.60, ^1^H-NMR (499.9 MHz; CDCl_3_) δ = 1.33–2.04 (m, 10H, cyclohexyl); 2.79 (s, 2H; H_2_-5); 3.86 (s, 3H; C(4′)-OCH_3_); 6.97 (m, 2H; H-3′, H-5′); 7.78 (m, 2H; H-2′, H-6′); 10.28 (br s, 1H; NH-2) ppm; ^13^C-NMR (125.7 MHz; CDCl_3_) δ = 20.2 (C-8, C-10); 25.4 (C-9); 27.6 (C-5); 33.44 (C-7, C-11); 41.14 (C-6); 55.45 (C(4′)-OCH_3_); 114.22 (C-3′, C-5′); 127.74 (C-2′, C-6′); 127.97 (C-1′); 154.54 (C-4), 161.61 (C-4′); 204.34 (C-1) ppm; HRMS: M + H = 289.13657 (delta = −1.2 ppm; C_16_H_21_ON_2_S). HR-ESI-MS-MS (CID = 35%; rel. int. %): 272(4); 255(100); 255(46); 241(4); 166(8); 156(10); 134(6); 121(6).

1-(4-Metoxyphenyl)-2,3-diazaspiro[5.5]undec-1-ene-4-thione (**6e**). Yield = 14%, R_f_(heptane:dichloromethane:methanol/5:5:1) = 0.40, ^1^H NMR (399.8 MHz; CDCl_3_) δ = 1.10–1.24 (m; 1H; Hax-9); 1.45–1.60 (m; 4H; H_2_-8, H_2_-10); (br s; 1H; NH-3); 1.61–1.68 (m; 4H; H_2_-7, H_2_-11); 1.69–1.75 (m; 1H; Heq-9); 3.08 (s; 2H; H_2_-5); 3.84 (s; 3H; C(4′)-OCH_3_); 6.90–6.95 (m; 2H; H-3′, H-5′); 7.30–7.35 (m; 2H; H-2′, H-6′); 10.05 (br s; 1H; NH-3) ppm; ^13^C NMR (100.5 MHz; CDCl_3_) δ = 20.2 (C-8, C-10); 25.4 (C-9); 31.4 (C-7, C-11); 36.8 (C-6); 42.7 (C-5); 55.4 (C(4′)-OCH_3_); 113.7 (C-3′, C-5′); 127.5 (C-1′); 129.5 (C-2′, C-6′); 160.4 (C-4′); 165.8 (C-1); 193.9 (C-4) ppm; HRMS: M + H = 289.13769 (delta = 2.7 ppm; C_16_H_21_ON_2_S). MS-MS (CID = 45%; rel. int. %): 272(6); 257(6); 255(11); 189(38); 181(39); 121(100); 113(12).

4-(*p*-Tolyl)-2,3-diazaspiro[5.5]undec-3-ene-1-thione (**6b**). Yield = 41%, R_f_(heptane:dichloromethane:methanol/5:5:1) = 0.60, LC-MS: UV (220 nm) (M + H = 273): 100%, UV (254 nm) (M + H = 273): 100%, ^1^H NMR (499.9 MHz; DMSO-*d*_6_) *δ* = 1.16–1.84 (m, 10H, cyclohexyl); 2.35 (s, 3H; C(4′)-CH_3_); 2.83 (s, 2H; H_2_-5); 7.29 (m, 2H; H-3′, H-5′); 7.82 (m, 2H; H-2′, H-6′); 12.69 (br s, 1H; NH-2) ppm; ^13^C-NMR (125.7 MHz; DMSO-*d*_6_) *δ* = 20.35 (C-8, C-10); 20.86 (C(4′)-CH_3_); 25.26 (C-9); 26.39 (C-5); 32.75 (C-7, C-11); 40.05 (C-6); 125.93 (C-2′, C-6′); 129.28 (C-3′, C-5′); 132.68 (C-1′); 140.12 (C-4′); 154.26 (C-4); 202.78 (C-1) ppm; HRMS: M + H = 273.14235 (delta = −1.6 ppm; C_16_H_21_N_2_S). MS-MS (CID = 45%; rel. int. %): 256(5); 241(53); 240(100); 239(75); 225(4); 186(4); 171(5); 166(11); 159(18); 156(12); 133(6); 118(5).

4-(4-Chlorophenyl)-2,3-diazaspiro[5.5]undec-3-ene-1-thione (**6c**). Yield = 55%, R_f_(heptane:dichloromethane:methanol/5:5:1) = 0.66, LC-MS: UV (220 nm) (M + H = 293): 100%, UV (254 nm) (M + H = 293): 100%, ^1^H NMR (499.9 MHz; DMSO-*d*_6_) *δ* = 1.16–1.84 (m, 10H, cyclohexyl); 2.86 (s, 2H; H_2_-5); 7.54 (m, 2H; H-3′, H-5′); 7.95 (m, 2H; H-2′, H-6′); 12.78 (brbr s, 1H; NH-2) ppm; ^13^C-NMR (125.7 MHz; DMSO-*d*_6_) *δ* = 20.28 (C-8, C-10); 25.23 (C-9); 26.33 (C-5); 32.85 (C-7, C-11); 40.05 (C-6) 127.76 (C-2′, C-6′); 128.72 (C-3′, C-5′); 134.31 (C-1′); 134.87 (C-4′); 153.11 (C-4); 203.20 (C-1) ppm; HRMS: M + H = 293.08752 (delta = 0.5 ppm; C_15_H_18_N_2_ClS). HR-ESI-MS-MS (CID = 45%; rel. int. %): 276(5); 259(100); 191(23); 179(28); 166(6); 154(5).

4-(3,4-Dimethoxyphenyl)-2,3-diazaspiro[5.5]undec-3-ene-1-thione (**6d**). Yield = 40%, R_f_(heptane:dichloromethane:methanol/5:5:1) = 0.40, ^1^H NMR (499.9 MHz; CDCl_3_) δ = 1.27–1.35 (m; 1H; Hax-9); 1.35–1.45 (m; 2H; Hax-8, Hax-10); 1.50–1.56 (m; 2H; Heq-7, Heq-11); 1.67–1.75 (m; 3H; Heq-8, Heq-10, Heq-9); 2.01–2.08 (m; 2H; Hax-7, Hax-11); 2.79 (s; 2H; H_2_-5); 3.94 (s; 3H; C(4′)-OCH_3_); 3.95 (s; 3H; C(3′)-OCH_3_); 6.91 (d, *J* = 8,4 Hz; 1H; H-5′); 7.29 (dd, J = 8.4, 2.1 Hz; 1H; H-6′); 7.50 (d, *J* = 2.1 Hz; 1H; H-2′); 10.11 (br s; 1H; NH-2) ppm; ^13^C-NMR (125.7 MHz; CDCl_3_) δ = 21.2 (C-8, C-10); 25.4 (C-9); 27.4 (C-5); 33.5 (C-7, C-11); 41.2 (C-6); 55.99, 56.02 (C(3′)-OCH_3_, C(4′)-OCH_3_); 108.1 (C-2′); 110.5 (C-5′); 119.8 (C-6′); 128.2 (C-1′); 149.4 (C-3′); 151.5 (C-4′); 154.4 (C-4); 204.6 (C-1) ppm; HRMS: M + H = 319.14735 (delta = −0.39 ppm; C_17_H_23_O_2_N_2_S). MS-MS (CID = 65%; rel. int. %): 304(5); 302(5); 287(100); 286(75); 285(45); 271(10).

9-(4-Methoxyphenyl)-7,8-diazaspiro[4.5]dec-8-ene-6-thione (**7a**). Yield = 71%, R_f_(heptane:dichloromethane:methanol/5:5:1) = 0.40, ^1^H NMR (499.9 MHz; CDCl_3_) δ = 1.56–1.62 (m; 2H; Hx-1, Hx-4); 1.68–1.77 (m; 2H; Hx-2, Hx-3); 1.87–1.96 (m; 2H; Hy-2, Hy-3); 2.28–2.35 (m; 2H; Hy-1, Hy-4); 2.68 (s; 2H; H_2_-10); 3.86 (s; 3H; C(4′)-OCH_3_); 6.93–6.97 (m; 2H; H-3′, H-5′); 7.71–7.75 (m; 2H; H-2′, H-6′); 10.13 (br s; 1H; NH-7) ppm; ^13^C-NMR (125.7 MHz; CDCl_3_) δ = 25.5 (C-2, C-3); 32.9 (C-10); 38.8 (C-1, C-4); 48.9 (C-5); 55.5 (C(4′)-OCH_3_); 114.2 (C-3′, C-5′); 127.7 (C-2′, C-6′); 128.0 (C-1′); 155.2 (C-9); 161.6 (C-4′); 204.2 (C-6) ppm; HRMS: M + H = 275.12072 (delta = −1.9 ppm; C_15_H_19_ON_2_S). MS-MS (CID = 45%; rel. int. %): 259(3); 242(100); 227(5); 201(6); 152(7); 142(7); 134(3).

9-(3,4-Dimethoxyphenyl)-7,8-diazaspiro[4.5]dec-8-ene-6-thione (**7b**). Yield = 89%, R_f_(heptane:dichloromethane:methanol/5:5:1) = 0.40, ^1^H-NMR (499.9 MHz; CDCl_3_) *δ* = 1.57–1.63 (m; 2H; Hx-1, Hx-4); 1.69–1.78 (m; 2H; Hx-2, Hx-3); 1.87–1.96 (m; 2H; Hy-2, Hy-3); 2.29–2.36 (m; 2H; Hy-1, Hy-4); 2.69 (s; 2H; H_2_-10); 3.93 (s; 3H), 3.95 (s; 3H): C(3′)-OCH_3_, C(4′)-OCH_3_; 6.89 (d, *J* = 8.4 Hz; 1H; H- 5′); 7.23 (dd, *J* = 8.4, 2.0 Hz; 1H; H-6′); 7.47 (d, *J* = 2.0 Hz; 1H; H-2′); 10.23 (br s; 1H; NH-7) ppm; ^13^C-NMR (125.7 MHz; CDCl_3_) *δ* = 25.5 (C-2, C-3); 32.8 (C-10); 38.8 (C-1, C-4); 49.0 (C-5); 55.98, 56.02 (C(3′)-OCH_3_, C(4′)-OCH_3_); 108.1 (C-2′); 110.5 (C-5′); 119.8 (C- 6′); 128.2 (C-1′); 149.3 (C-3′); 151.4 (C-4′); 155.1 (C-9); 204.2 (C-6) ppm; HRMS:M + H = 305.13149 (delta = −1.10 ppm; C_16_H_21_O_2_N_2_S). MS-MS (CID = 35%; rel. int. %): 288(5); 273(100); 272(80); 271(38); 257(12); 231(18); 164(8); 138(7).

### 3.6. General Procedure for the Preparation of Hydrazones (***8a–c***)

A solution of hydrazine monohydrate (0.10 mL, 1.2 mmol) in THF (5 mL) was added to the corresponding pyridazinone derivatives (**6a–c**) (0.40 mmol) in THF (15 mL) dropwise. The reaction mixture was stirred at reflux for 5 h, then the solvent was evaporated. The residue was dissolved in dichloromethane (20 mL) and washed with distilled water (2 × 10 mL). The organic layer was dried over MgSO_4_, filtered and evaporated. The crude product was purified by preparative thin layer chromatography (eluent: heptane:dichloromethane:methanol/5:5:1) to give the hydrazones (**8a–c**).

1-Hydrazono-4-(4-Methoxyphenyl)-2,3-diazaspiro[5.5]undec-3-ene (**8a**). Yield: 48%, Rf(heptane:dichloromethane:methanol/5:5:1) = 0.30, ^1^H NMR (499.9 MHz; DMSO-*d*_6_) δ = 1.20–1.64 (m, 10H, cyclohexyl); 2.62 (s, 2H, H-5); 3.78 (s, 3H, C(4′)-OCH_3_); 5.01 (br s, 2H, NH_2_-2″); 6.95 (m, 2H, H-3′, H-5′); 7.69 (m, 2H, H-2′, H-6′); 9.32 (br s, 1H, NH-2) ppm; ^13^C-NMR (125.7 MHz; DMSO-*d*_6_) δ = 20.7 (C(4′)-CH_3_); 21.1 (C-8, C-10); 25.7(C-9); 31.0 (C-5); 32.6 (C-6); 33.0 (C-7, C-11); 55.1 (C(4′)-OCH_3_); 113.7 (C-3′, C-5′); 126.0 (C-2′, C-6′);130.2 (C-1′); 142.8 (C-4); 144.8 (C-1); 159.2 (C-4′) ppm; HRMS: M + H = 287.18646 (delta = −0.6 ppm; C_16_H_23_ON_4_). HR-ESI-MS-MS (CID = 35%; rel. int. %): 270(99); 255(21); 242(47); 228(22); 216(13); 186(21); 164(100); 148(10); 133(19); 121(23).

1-Hydrazono-4-(*p*-tolyl)-2,3-diazaspiro[5.5]undec-3-ene (**8b**). Yield: 59%, R_f_(heptane:dichloromethane:methanol/5:5:1) = 0.27. ^1^H-NMR (499.9 MHz; DMSO-*d*_6_) δ = 1.18–1.72 (m, 10H, cyclohexyl); 2.31 (s, 3H, C(4′)-CH_3_); 2.62 (s, 2H, H-5); 5.07 (br s, 2H, NH_2_-2″); 7.19 (m, 2H, H-3′, H-5′); 7.64 (m, 2H, H-2′, H-6′); 9.39 (br s, 1H, NH-2) ppm; ^13^C NMR (125.7 MHz; DMSO-*d*_6_) δ = 21.1 (C-8, C-10); 25.7(C-9); 31.1 (C-5); 32.5 (C-6); 33.0 (C-7, C-11); 124.6 (C-2′, C-6′); 128.9 (C-3′, C-5′); 134.9 (C-1′); 137.3 (C-4′); 142.8 (C-4); 144.6 (C-1) ppm; HRMS: M + H = 271.19165 (delta = −0.3 ppm; C_16_H_23_N_4_). HR-ESI-MS-MS (CID = 35%; rel. int. %): 254(70); 239(45); 226(54); 212(40); 181(29); 164(100); 147(67); 131(27); 117(59); 105(24).

4-(4-Chlorophenyl)-1-hydrazono-2,3-diazaspiro[5.5]undec-3-ene (**8c**). Yield = 61%, R_f_(heptane:dichloromethane:methanol/5:5:1) = 0.50, ^1^H-NMR (499.9 MHz; DMSO-*d*_6_) δ = 1.25–1.58 (m, 10H, cyclohexyl); 2.64 (s, 2H, H-5); 5.12 (br s, 2H, NH_2_-2″); 7.43 (m, 2H, H-3′, H-5′); 7.77 (m, 2H, H-2′, H-6′); 9.57 (br s, 1H, NH-2) ppm; ^13^C-NMR (125.7 MHz; DMSO-*d*_6_) δ = 21.1 (C-8, C-10); 25.7(C-9); 31.0 (C-5); 32.4 (C-6); 33.0 (C-7, C-11); 126.3 (C-2′, C-6′); 128.3 (C-3′, C-5′); 132.4 (C-4′); 136.5 (C-1′); 141.4 (C-4); 144.0 (C-1) ppm; HRMS: M + H = 291.13630 (delta = −2.8 ppm; C_15_H_20_N_4_Cl). HR-ESI-MS-MS (CID = 45%; rel. int. %): 274(38); 261(42); 259(46); 246(26); 232(35); 220(21); 204(24); 201(22); 190(100); 167(26); 164(52); 152(22); 137(82); 125(29).

### 3.7. Preparation of Tetrazolo-Pyridazines (***9a–c***)

A solution of sodium nitrite (35.2 mg, 0.51 mmol) in water (3.8 mL) was added dropwise to a suspension of (**8a–c**) (100 mg, 0.34 mmol) in 0.5 N HCI (1.22 mL) at 5 °C. After stirring for 2 h, the reaction mixture was neutralized with a saturated solution of NaHCO_3_ (0.40 mL) and the precipitate was filteredand it was purified by preparative thin layer chromatography (eluent: heptane:dichloromethane:methanol/5:5:1) to give the tetrazolo-pyridazines (**9a–c**).

6′-(4-Methoxyphenyl)-7′H-spiro[cyclohexane-1,8′-tetrazolo[1,5-b]pyridazine] (**9a**). Yield = 66%, LC-MS: UV (220 nm) (M + H = 298): 100%, UV (254 nm) (M + H = 298): 100%, Rf(heptane:dichloromethane:methanol/5:5:1) = 0.42, ^1^H NMR (499.9 MHz; DMSO-*d*_6_) δ = 1.36–1.85 (m, 10H, cyclohexyl); 3.27 (s, 2H, H-7′); 3.87 (s, 3H, C(4″)-OCH_3_); 7.12 (m, 2H, H-3″, H-5″); 8.08 (m, 2H, H-2″, H-6″) ppm; ^13^C NMR (125.7 MHz; DMSO-*d*_6_) δ = 20.98 (C-2, C-5); 24.85 (C-4); 32.28 (C-1 = C-8′); 33.44 (C-2, C-6); 34.98 (C-7′); 114.34 (C-3″, C-5″); 125.73 (C-1″); 129.44 (C-2″, C-6″); 149.62 (C-9′);162.59 (C-4″); 164.16 (C-6′) ppm; HRMS: M + H = 298.16613 (delta = −0.4 ppm; C_16_H_20_ON_5_). HR-ESI-MS-MS (CID = 40%; rel. int. %): 270(100); 242(73); 227(6); 215(8); 200(4); 174(19); 162(27); 160(18); 133(5); 121(4).

6′-(*p*-Tolyl)-7′H-spiro[cyclohexane-1,8′-tetrazolo[1,5-b]pyridazine] (**9b**). Yield = 45%, LC-MS: UV (220 nm) (M + H = 282): 100%, UV (254 nm) (M + H = 282): 100% Rf(heptane:dichloromethane:methanol/5:5:1) = 0.42, ^1^H-NMR (499.9 MHz; DMSO-*d*_6_) δ = 1.36–1.86 (m, 10H, cyclohexyl); 2.41 (s, 3H, C(4″)-CH_3_); 3.29 (s, 2H, H-7′); 7.40 (m, 2H, H-3″, H-5″); 8.00 (m, 2H, H-2″, H-6″) ppm; ^13^C-NMR (125.7 MHz; DMSO-*d*_6_) δ = 20.97 (C-2, C-5, C(4″)-CH_3_); 24.83 (C-4); 32.28 (C-1 = C-8′); 33.48 (C-2, C-6); 35.13 (C-7′); 129.51 (C-3″, C-5″); 127.44 (C-2″, C-6″); 130.80 (C-1″); 142.69 (C-4″); 149.73 (C-9′); 164.72 (C-6′) ppm; HRMS: M + H = 282.17121 (delta = −0.4 ppm; C_16_H_20_N_5_). HR-ESI-MS-MS (CID = 40%; rel. int. %): 254(100); 226(64); 209(7); 184(4); 170(12); 158(9); 146(17); 144(14); 117(6).

6′-(4-Chlorophenyl)-7′*H*-spiro[cyclohexane-1,8′-tetrazolo[1,5-b]pyridazine] (**9c**). Yield = 77%, LC-MS: UV (220 nm) (M + H = 302): 97%, UV (254 nm) (M + H = 302): 98% R_f_(heptane:dichloromethane:methanol/5:5:1) = 0.42, ^1^H NMR (499.9 MHz; DMSO-*d*_6_) δ = 1.40–1.82 (m, 10H, cyclohexyl); 3.22 (s, 2H, H-7′); 7.60 (m, 2H, H-3″, H-5″); 8.12 (m, 2H, H-2″, H-6″) ppm; ^13^C NMR (125.7 MHz; DMSO-*d*_6_) δ = 20.91 (C-2, C-5); 24.80 (C-4); 32.29 (C-1 = C-8′); 33.56 (C-2, C-6); 35.13 (C-7′); 129.01 (C-3″, C-5″); 129.29 (C-2″, C-6″); 132.40 (C-1″); 137.19 (C-4″); 149.70 (C-9′); 163.95 (C-6′) ppm; HRMS: M + H = 302.11716 (delta = 1.5 ppm; C_15_H_17_N_5_Cl). MS-MS (CID = 40%; rel. int. %): 274(100); 246(44); 229(6); 211(13); 190(23); 178(8); 166(13); 164(21); 137(6).

### 3.8. General Procedure for the Preparation of 6-(4-Substituted-phenyl)-3-phenyl-7H-spiro[[1,2,4]triazolo[4,3-b]pyridazin-8,1′-cyclohexanes] (***10a–c***)

Benzhydrazide (**11**) (300 mg, 2.2 mmol) was added to the appropriate thioxo derivative (**6a**–**c**) (0.73 mmol) in *n*-BuOH (25 mL). The reaction mixture was refluxed until the starting material disappeared (84 h). The solvent was evaporated, then the residue was taken up in dichloromethane (15 mL). It was washed with 1% (*w*/*w*) aqueous HCl solution (2 × 10 mL), and then with water (10 mL). The organic layer was dried over MgSO_4_, then filtered and evaporated. The crude product was purified by preparative thin layer chromatography (eluent: heptane:dichloromethane:methanol/5:5:1) to give the triazole derivatives (**10a**–**c**).

6-(4-Methoxyphenyl)-3-phenyl-7*H*-spiro[[1,2,4]triazolo[4,3-b]pyridazin-8,1′-cyclohexane] (**10a**). Yield = 29%, LC-MS: UV (220 nm) (M + H = 373): 93%, UV (254 nm) (M + H = 373): 98%, R_f_(heptane:dichloromethane:methanol/5:5:1) = 0.32, ^1^H-NMR (799.7 MHz; DMSO-*d*_6_) δ = 1.45–1.85 (m, 10H, cyclohexyl); 3.14 (s, 2H, H-7); 3.85 (s, 3H, C(4″)-CH_3_); 7.10 (m, 2H, H-3″, H-5″); 7.53 (m, 1H, H-4‴); 7.57 (m, 2H, H-3‴, H-5‴); 8.01 (m, 2H, H-2″, H-6″); 8.12 (m, 2H, H-2‴, H-6‴) ppm; ^13^C-NMR (201.1 MHz; DMSO-*d*_6_) δ = 21.14 (C-3′, C-5′); 25.17 (C-4′); 32.65 (C-1′ = C-8); 33.63 (C-2′, C-6′); 34.07 (C-7); 55.39 (C(4″)-CH_3_); 114.25 (C-3″, C-5″); 126.59 (C-1‴); 127.03 (C-1″); 127.79 (C-2‴, C-6‴); 128.58 (C-3‴, C-5‴); 128.80 (C-2″, C-6″); 129.70 (C-4‴); 148.97 (C-3); 151.18 (C-9); 161.23 (C-6); 161.86 (C-4″) ppm; HRMS: M + H = 373.20153 (delta = −2.0 ppm; C_23_H_25_ON_4_). HR-ESI-MS-MS (CID = 40%; rel. int. %): 226(100); 148(11).

3-Phenyl-6-(*p*-tolyl)-7*H*-spiro[[1,2,4]triazolo[4,3-b]pyridazin-8,1′-cyclohexane] (**10b**). Yield = 14%, LC-MS: UV (220 nm) (M + H = 357): 87%, UV (254 nm) (M + H = 357): 85%, R_f_(heptane:dichloromethane:methanol/5:5:1) = 0.32, ^1^H-NMR (499.9 MHz; DMSO-*d*_6_) δ = 1.41–1.89 (m, 10H, cyclohexyl); 2.39 (s, 3H, C(4″)-CH_3_); 3.16 (s, 2H, H-7); 7.37 (m, 2H, H-3″, H-5″); 7.54 (m, 1H, H-4‴); 7.58 (m, 2H, H-3‴, H-5‴); 7.93 (m, 2H, H-2″, H-6″); 8.12 (m, 2H, H-2‴, H-6‴) ppm; ^13^C NMR (125.7 MHz; DMSO-*d*_6_) δ = 20.95 (C(4″)-CH_3_); 21.14 (C-3′, C-5′); 25.18 (C-4′); 32.65 (C-1′ = C-8); 33.67 (C-2′, C-6′); 34.21 (C-7); 126.52 (C-1‴); 126.98 (C-2″, C-6″); 127.85 (C-2‴, C-6‴); 128.60 (C-3‴, C-5‴); 129.44 (C-3″, C-5″); 129.75 (C-4‴); 132.09 (C-1″); 141.58 (C-4″); 149.09 (C-3); 151.21 (C-9); 161.73 (C-6) ppm; HRMS: M + H = 357.20934 (delta = 5.5 ppm; C_23_H_25_N_4_). HR-ESI-MS-MS (CID = 35%; rel. int. %): 226(100); 132(8).

6-(4-Chlorophenyl)-3-phenyl-7*H*-spiro[[1,2,4]triazolo[4,3-b]pyridazin-8,1′-cyclohexane] (**10c**). Yield = 42%, LC-MS: UV (220 nm) (M + H = 377): 100%, UV (254 nm) (M + H = 377): 99%, R_f_(heptane:dichloromethane:methanol/5:5:1) = 0.36, ^1^H NMR (499.9 MHz; DMSO-*d*_6_) δ = 1.41–1.90 (m, 10H, cyclohexyl); 3.18 (s, 2H, H-7); 7.51–7.59 (m, 3H, H-3‴, H-4‴, H-5‴); 7.63 (m, 2H, H-3″, H-5″); 8.05 (m, 2H, H-2″, H-6″); 8.09 (m, 2H, H-2‴, H-6‴) ppm; ^13^C-NMR (125.7 MHz; DMSO-*d*_6_) δ = 21.10 (C-3′, C-5′); 25.15 (C-4′); 32.65 (C-1′ = C-8); 33.71 (C-2′, C-6′); 34.20 (C-7); 126.38 (C-1‴); 127.90 (C-2‴, C-6‴); 128.64 (C-3‴, C-5‴); 128.83 (C-2″, C-6″); 128.94 (C-3‴, C-5‴); 129.83 (C-4‴); 133.65 (C-1″); 136.25 (C-4″); 149.24 (C-3); 151.04 (C-9); 160.90 (C-6) ppm; HRMS: M + H = 377.15255 (delta = −0.5 ppm; C_22_H_22_N_4_Cl). HR-ESI-MS-MS (CID = 35%; rel. int. %): 226(100).

## 4. Conclusions

Herein, we soughtto expand the family of spiro[cycloalkane]pyridazinones with further derivatives by anellating the spiro[cyclohexane]pyridazinones and spiro[cyclopentane]pyridazinones with tetrazole or triazole rings. To this end, seven new 4-substituted phenyl-2,3-diazaspiro[5.5]undec-3-ene-1-thiones and nine substituted phenyl-7,8-diazaspiro[4.5]dec-8-ene-6-thiones were prepared by the reaction of the spiro[cycloalkane]pyridazinones with phosphorus pentasulfide. By treating the *p*-substituted 4-phenyl-2,3-diazaspiro[5.5]undec-3-ene-1-thiones (**6a–c**) with hydrazine hydrate, the desired hydrazones were obtained (**8a–c**) to give the tetrazolo-pyridazine after diazotization (**9a–c**). The reaction of the *p*-substituted phenyldiazaspiroundecene-thiones (**6a–c**) with benzhydrazide (**11**) yielded the triazolo-pyridazines (**10a–c**). Eventually two kinds of new ring systems were prepared: a tetrazolo-pyridazine and triazolo-pyridazines.

## Figures and Tables

**Table 1:** The physicochemical parameters of the hydrazone derivatives (**8a–c** and **9a–c**).

Starting Material	R	Product	Fsp^3^	LogP	ClogP	TPSA
**8a**	OMe	9a	0.50	3.08 *	3.55	61.91
**8b**	Me	9b	0.50	3.47 *	4.13	52.68
**8c**	Cl	9c	0.47	3.70 *	4.34	52.68

The physicochemical parameters were calculated using ChemDraw, but only Molinspiration software was suitable for calculating logP values *.

**Table 2:** The physicochemical parameters of drugs and drugs under development ^a^.

Drug	Fsp^3^	LogP	ClogP	TPSA
Hydralazine	0	0.73	1.17	62.77
Zopolrestate	0.16	4.37	2.55	82.33
Levosimendan	0.21	1.23	1.91	113.43
Pipofezin	0.38	1.98	3.33	43.67
Minaprine	0.41	2.70	3.19	49.22
Talazoparib ^a^	0.16	2.58	0.35	81.45
Relugolix ^a^	0.21	4.98	4.58	128.17
Ensartinib ^a^	0.27	4.04	5.22	121.41
Olaparib ^a^	0.34	2.02	1.24	82.08

The physicochemical parameters were calculated using ChemDraw, ^a^ drugs under development.

**Table 3:** The physicochemical parameters of the triazole derivatives (**10a–c**).

Starting Material	R	Product	Fsp^3^	logP	ClogP	TPSA
**6a**	OMe	**10a** *	0.35	5.33	4.36	49.55
**6b**	Me	**10b** *	0.35	5.72	4.94	40.32
**6c**	Cl	**10c** *	0.32	5.95	5.16	40.32

The physicochemical parameters were calculated using ChemDraw, but only Molinspiration software was suitable for calculating logP values *.

## Data Availability

The data presented in this study are available in Supplementary information.

## Supplementary Materials

The followings are available, Figure: NMR and HRMS spectrum.

## Sample Availability

Samples of the compounds prepared are available from the authors.
