# Supplementary material for: Novel Ring Systems: Spiro[Cycloalkane] Derivatives of Triazolo- and Tetrazolo-Pyridazines
Source: Molecules. 2021 Apr 8;26(8):2140. doi: 10.3390/molecules26082140 (PMC8068119; doi:10.3390/molecules26082140)

Supporting Information

Research Article

Novel ring systems: Spiro[cycloalkane] derivatives of Triazolo- and Tetrazolo-pyridazines

Csilla Sepsey Für[1], Gergő Riszter[1], Áron Szigetvári[2], Miklós Dékány[2], György Keglevich[1], László Hazai[1] and Hedvig Bölcskei[1,*]

[1] Department of Organic Chemistry and Technology, Budapest University of Technology and Economics, 1111 Budapest, Hungary; sepsey.fur.csilla@vbk.bme.hu (Cs.S.F.), riszter93@gmail.com (G.R.), keglevich.gyorgy@vbk.bme.hu (G.K), hazai.laszlo@vbk.bme.hu (L.H.), bolcskei.hedvig@vbk.bme.hu (H.B.)

[2] Gedeon Richter Plc. Budapest X., Gyömrői út 19-21., Budapest 10. POB.27. H-1475, Hungary; szigetvaria@richter.hu (Á.Sz.), m.dekany@richter.hu (D.M.)

* Correspondence: bolcskei.hedvig@vbk.bme.hu Tel.: Tel: +36-1-463-2229

^{1}H NMR (499.9 MHz; $CDCl_3$)

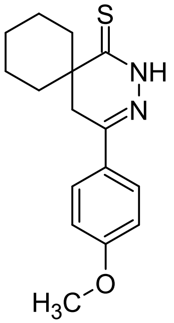

6a

4-(4-Methoxyphenyl)-2,3-diazaspiro[5.5]undec-3-ene-1-thione

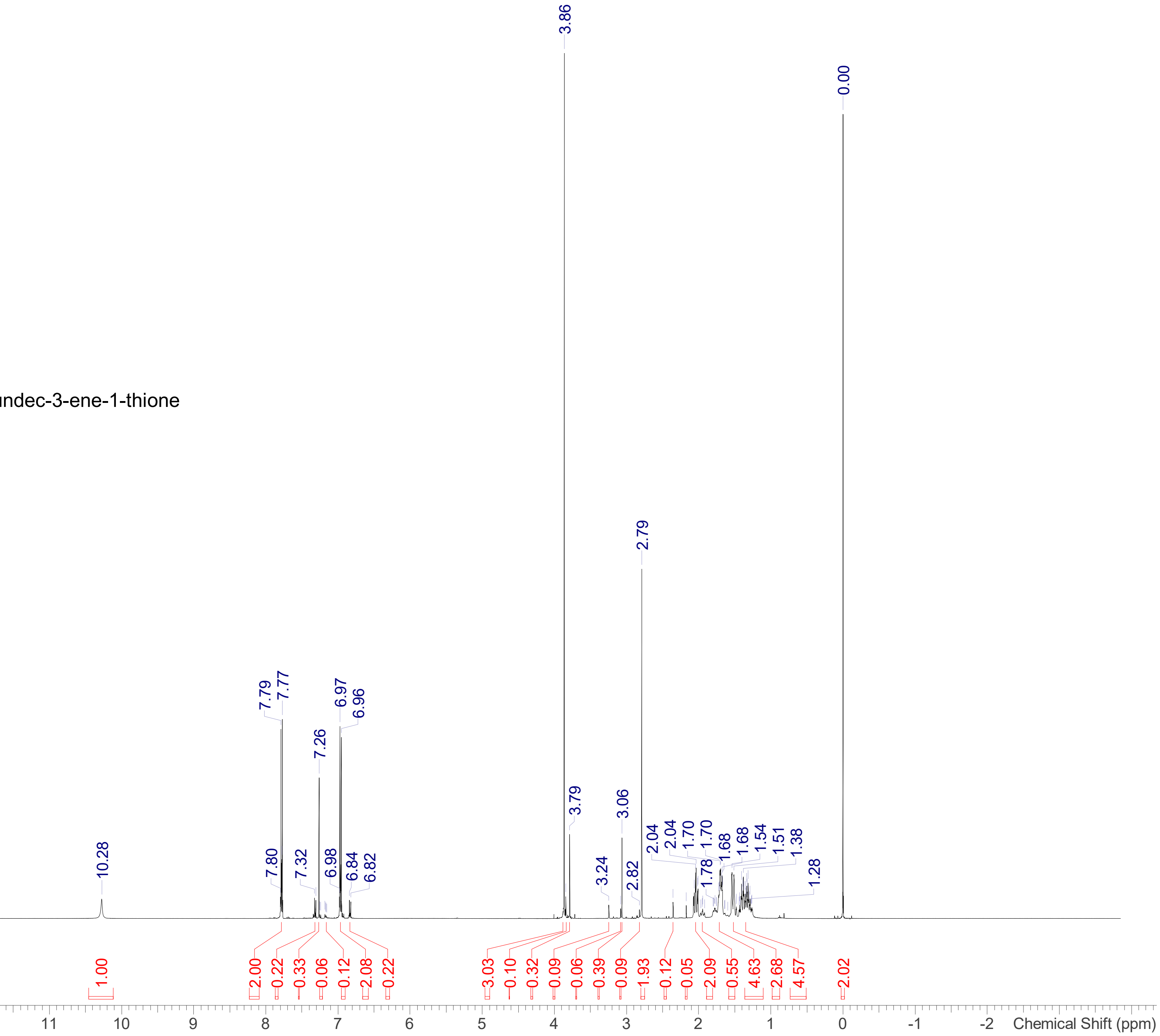

^{1}H NMR (499.9 MHz; $CDCl_3$)

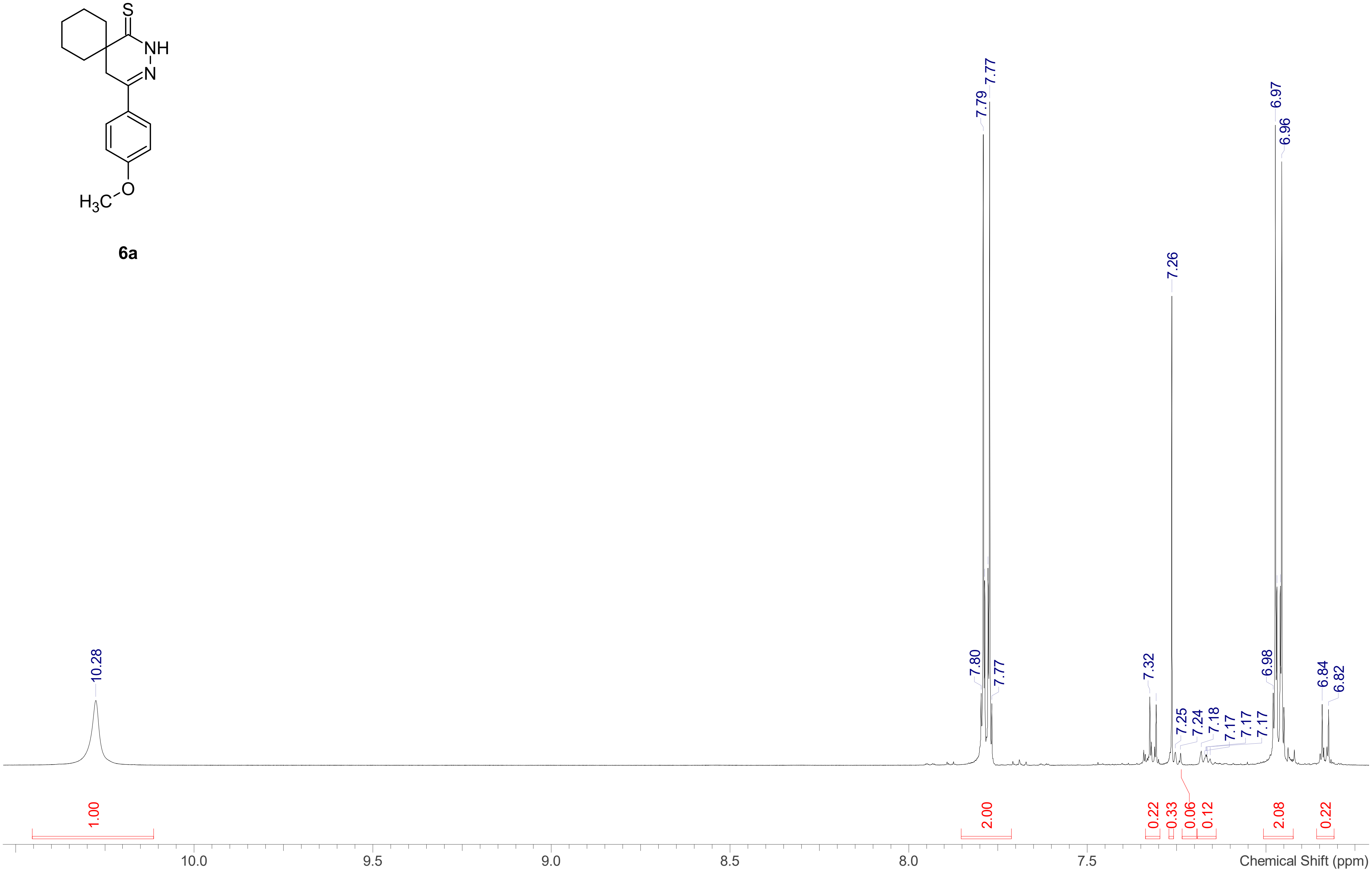

^{1}H NMR (499.9 MHz; $CDCl_3$)

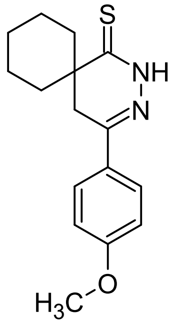

6a

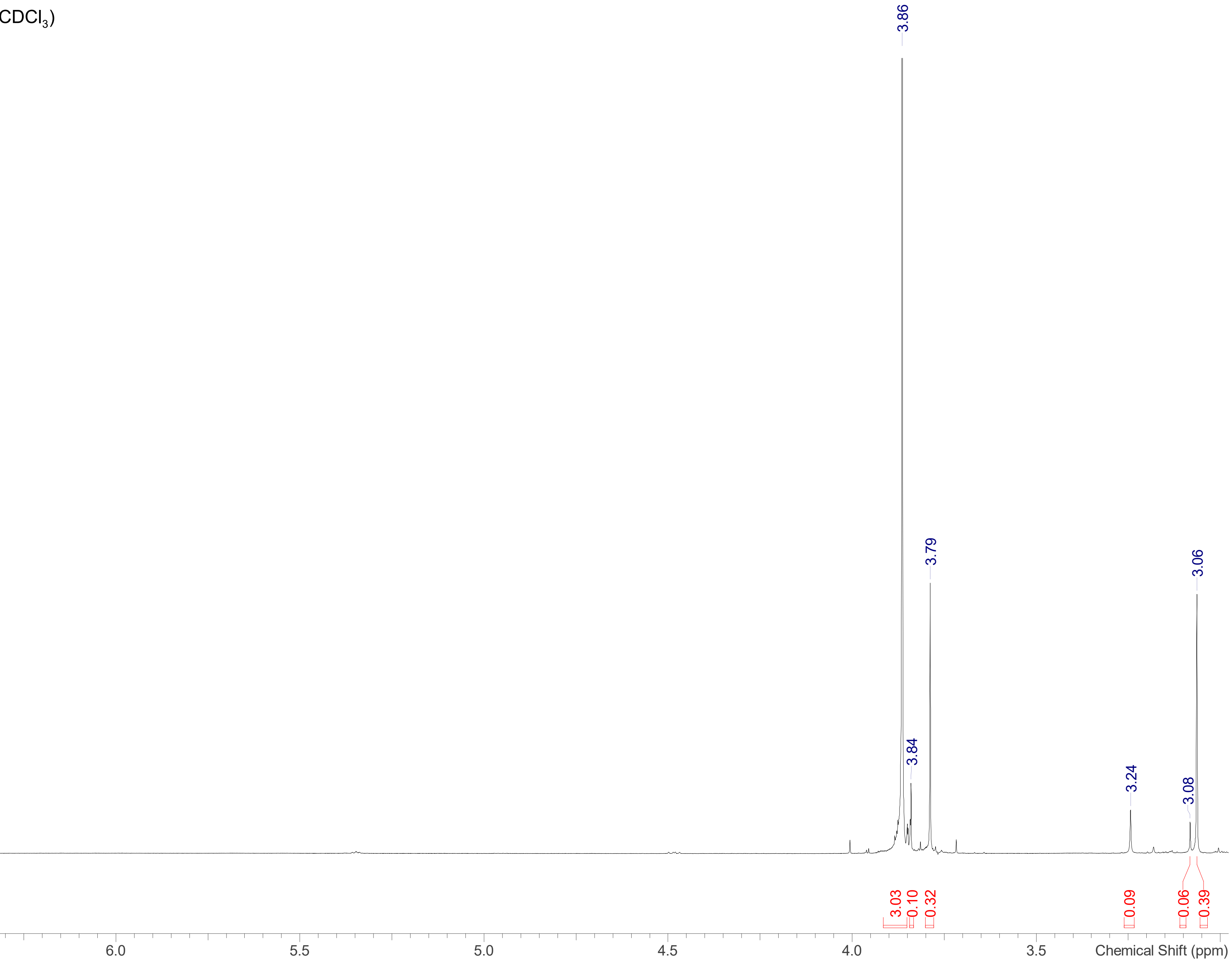

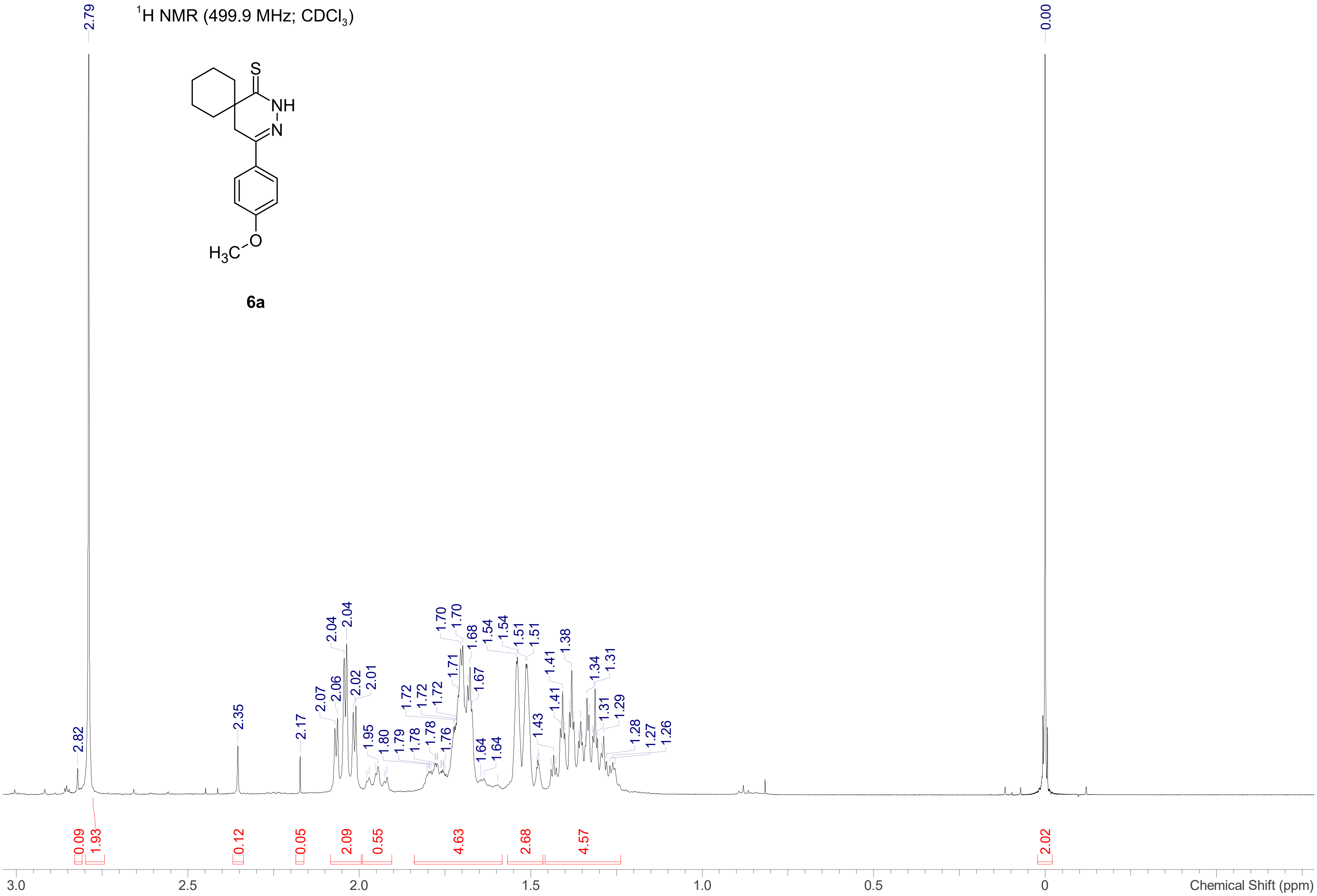

^{1}H NMR (499.9 MHz; $CDCl_3$)
S
NH
N
H_3C
O
6a
2.79
0.00
2.82
2.35
2.17
2.07
2.06
2.04
2.04
2.02
2.01
1.95
1.80
1.79
1.78
1.78
1.76
1.72
1.72
1.72
1.71
1.70
1.70
1.68
1.67
1.64
1.64
1.54
1.54
1.51
1.51
1.43
1.41
1.41
1.38
1.34
1.31
1.31
1.29
1.28
1.27
1.26
0.09
1.93
0.12
0.05
2.09
0.55
4.63
2.68
4.57
2.02
3.0
2.5
2.0
1.5
1.0
0.5
0
Chemical Shift (ppm)

^{13}C NMR (125.7 MHz; $CDCl_3$)

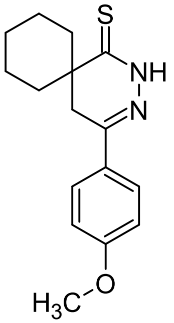

6a

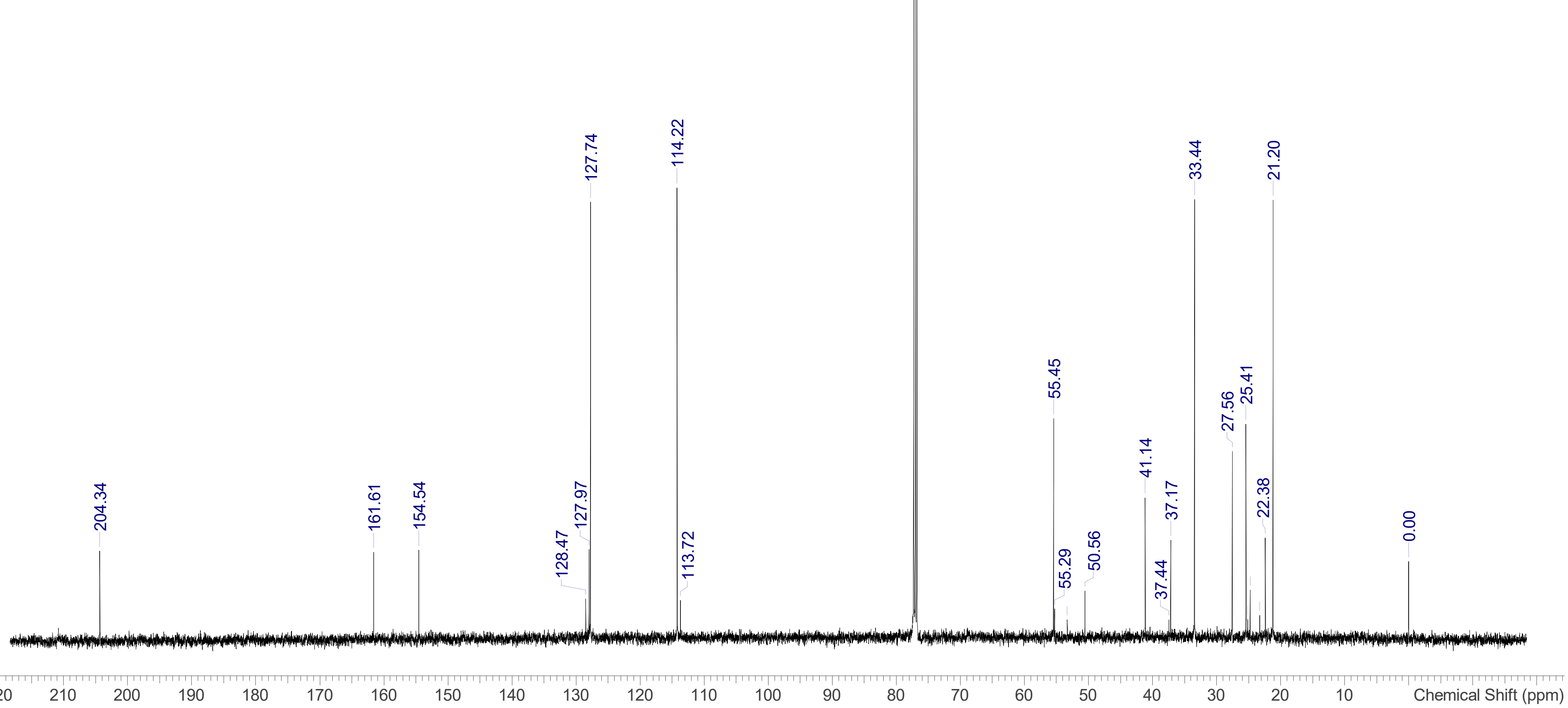

^{13}C NMR (125.7 MHz; $CDCl_3$)

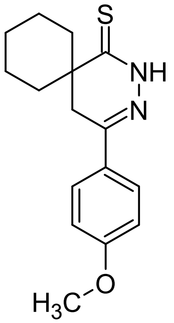

6a

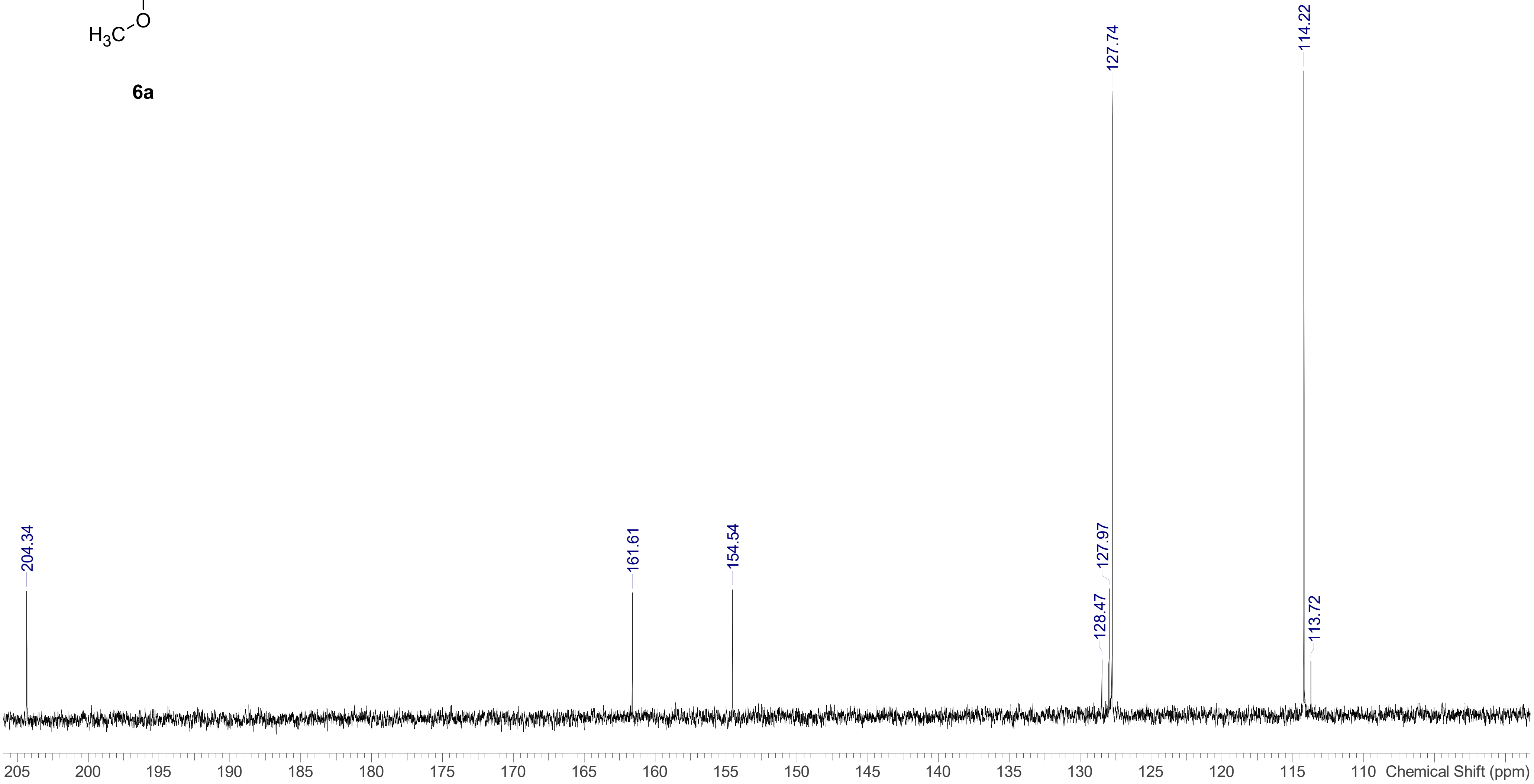

^{13}C NMR (125.7 MHz; $CDCl_3$)

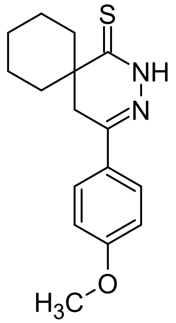

6a

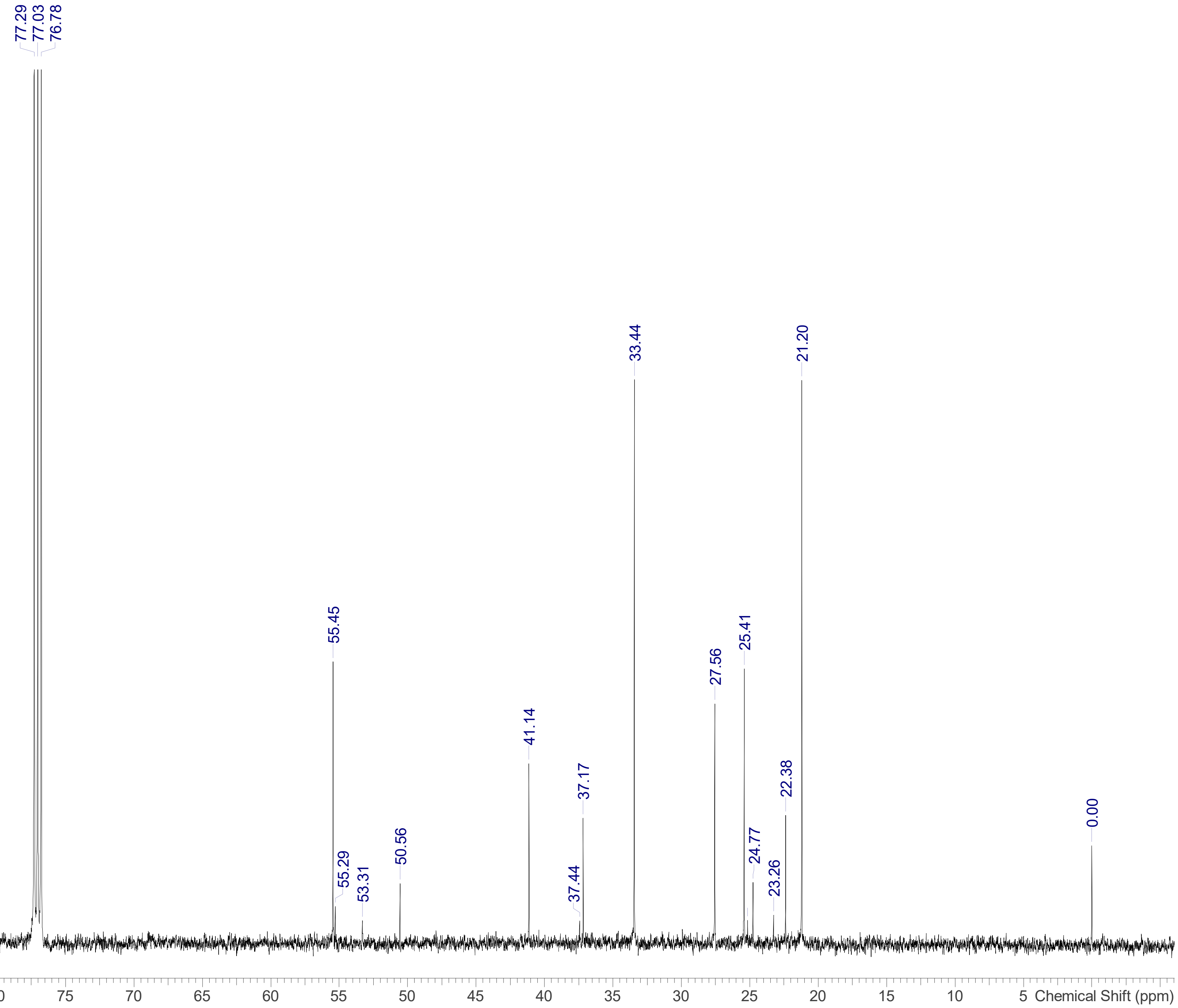

Compound **6a**

HRMS spectrum (ESI)

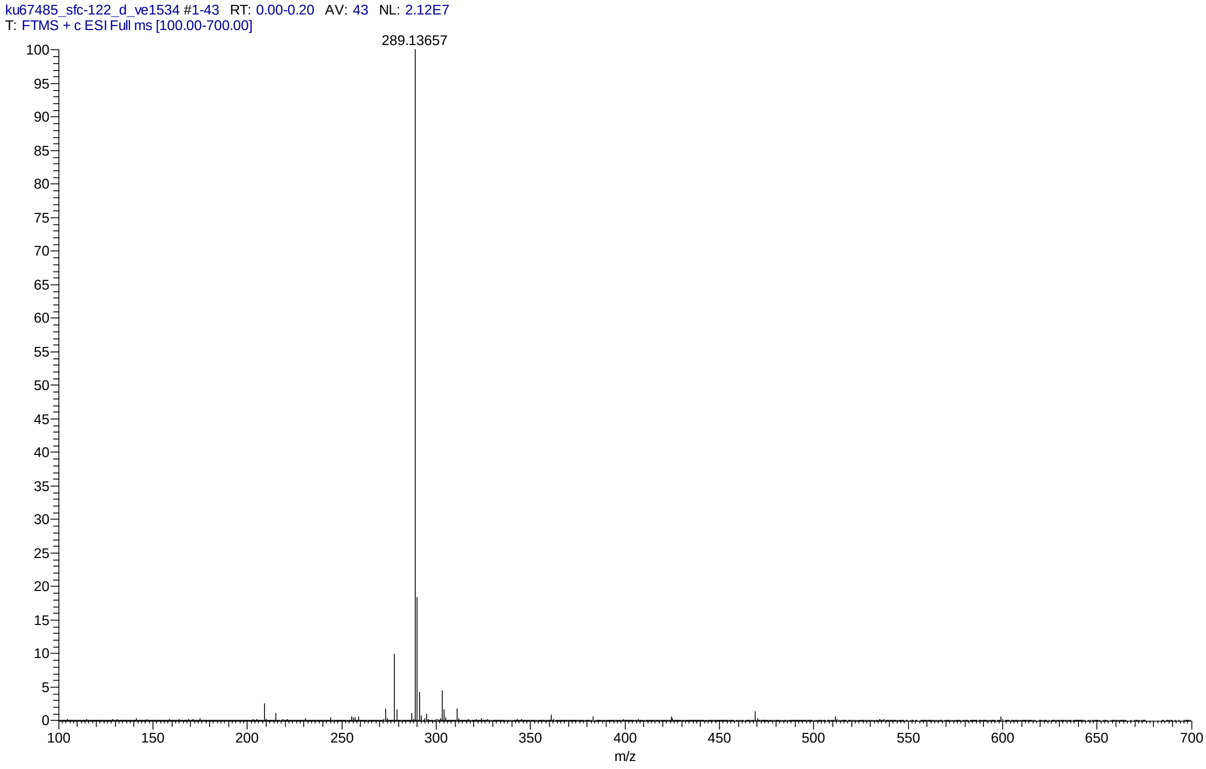

HRMS spectrum (ESI daughter ion)

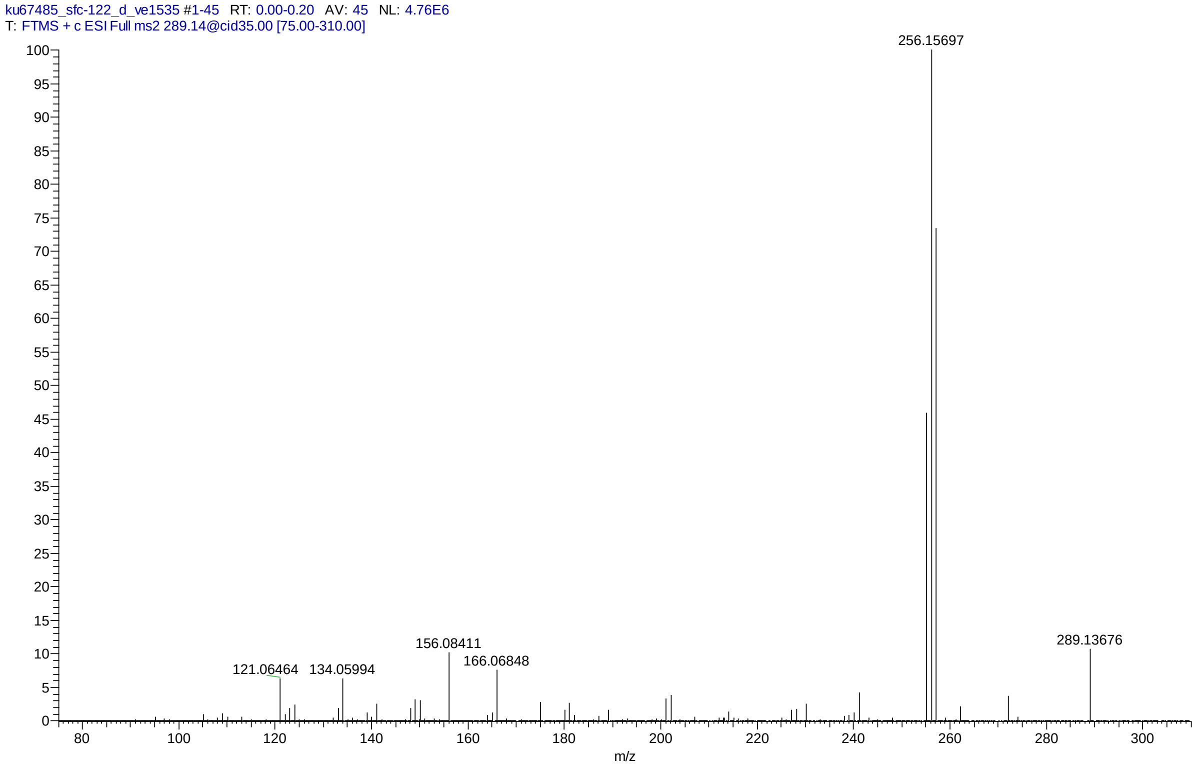

^{1}H NMR (499.9 MHz; DMSO-d_6)

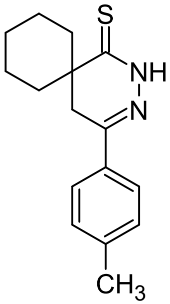

6b

4-(*p*-Tolyl)-2,3-diazaspiro[5.5]undec-3-ene-1-thione

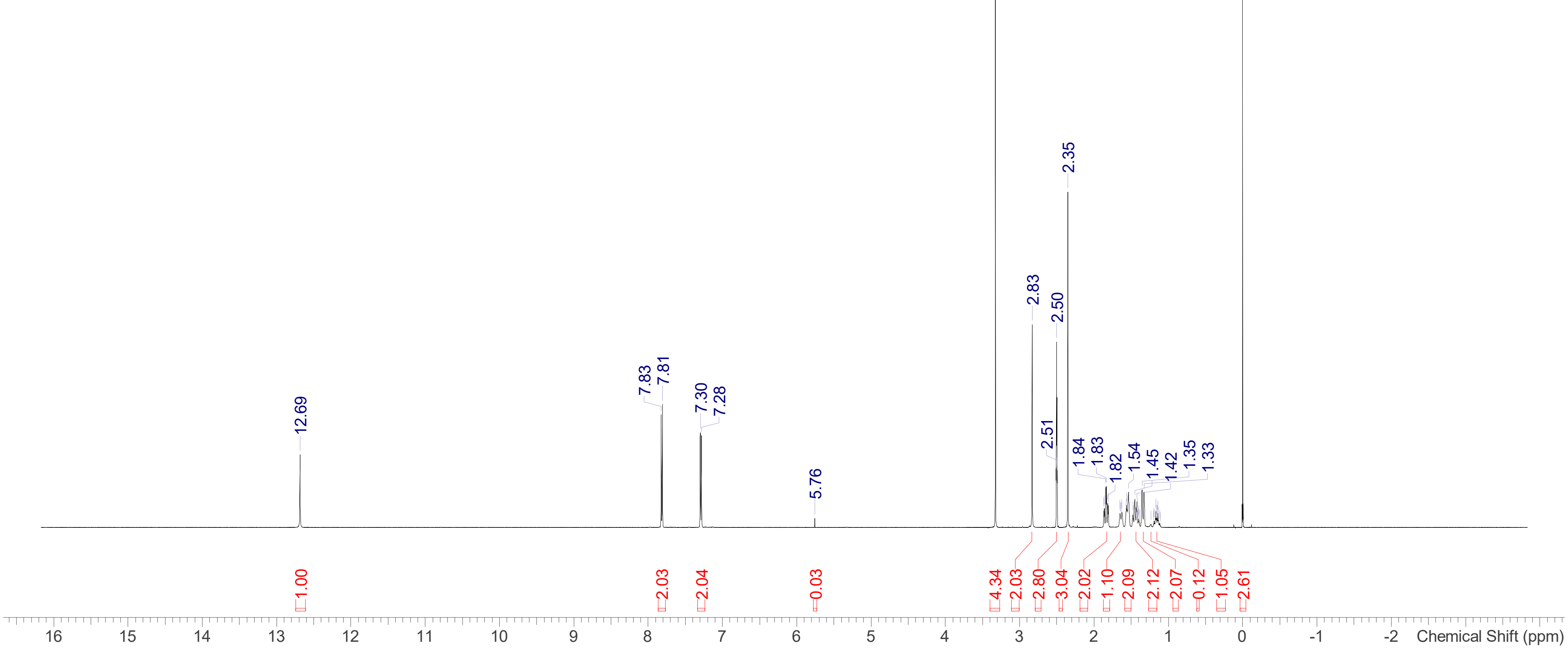

^{1}H NMR (499.9 MHz; DMSO-d_6)

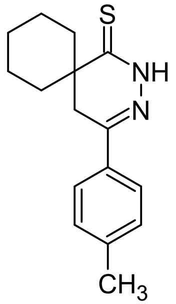

6b

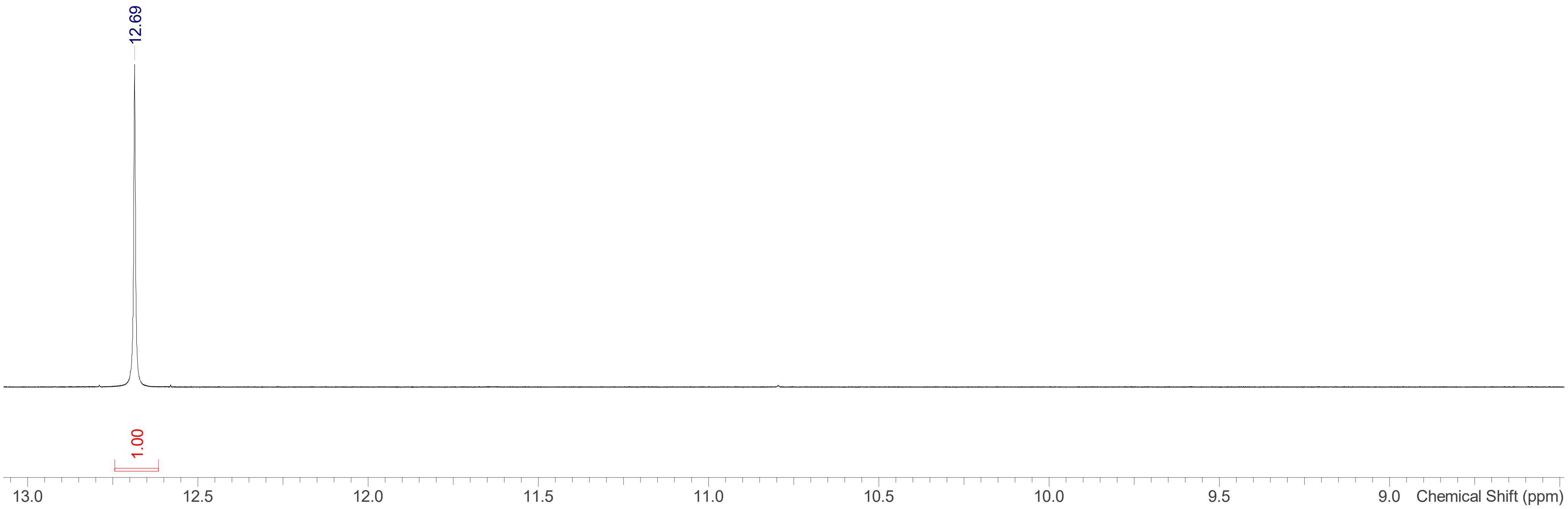

^{1}H NMR (499.9 MHz; DMSO-d_6)

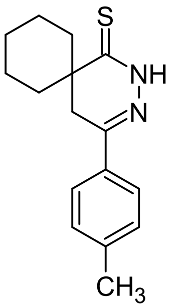

6b

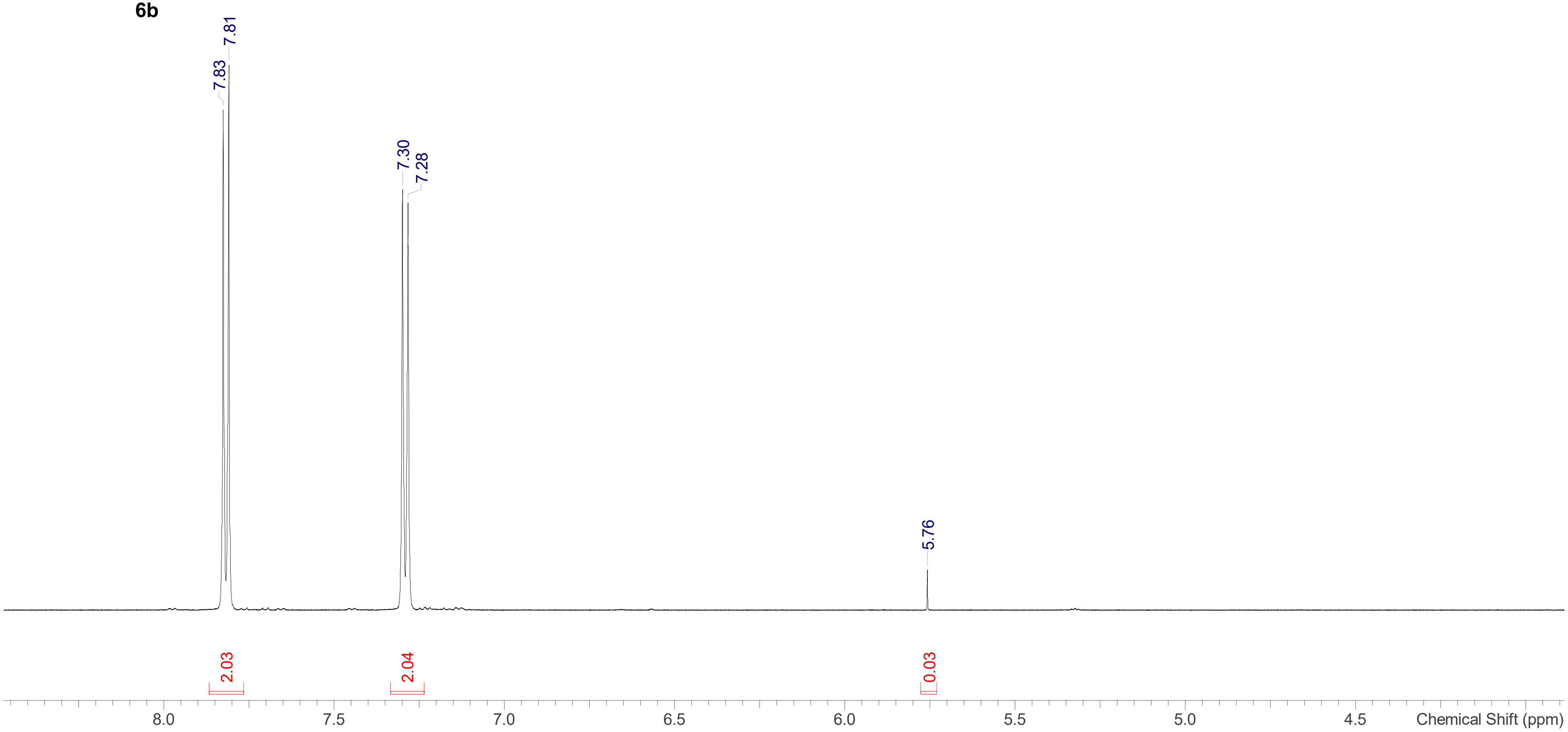

^{1}H NMR (499.9 MHz; DMSO-d_6)

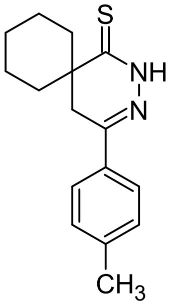

6b

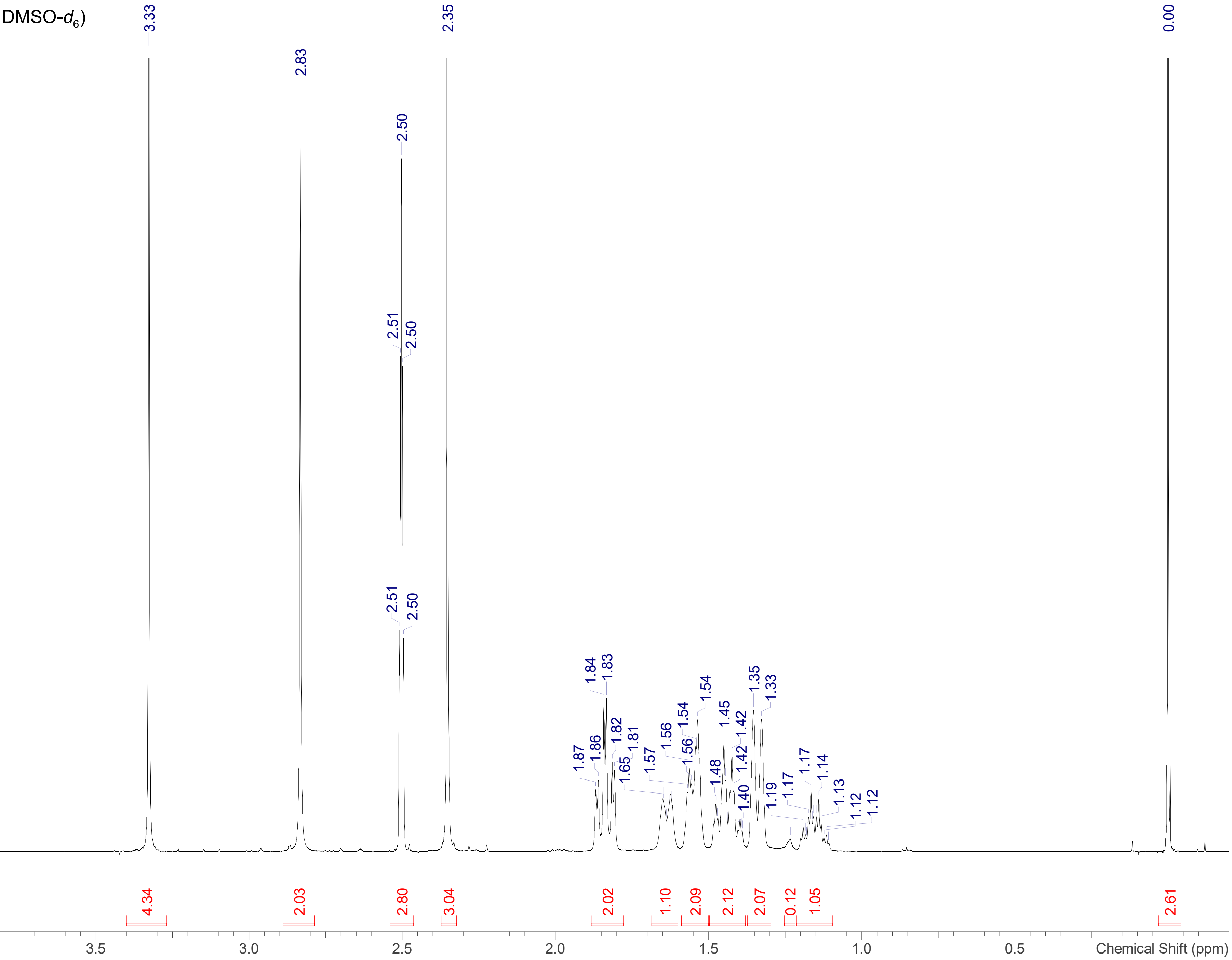

^{13}C NMR (125.7 MHz; DMSO-d_6)

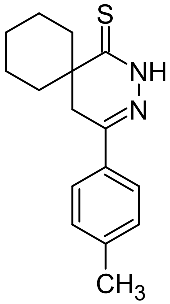

6b

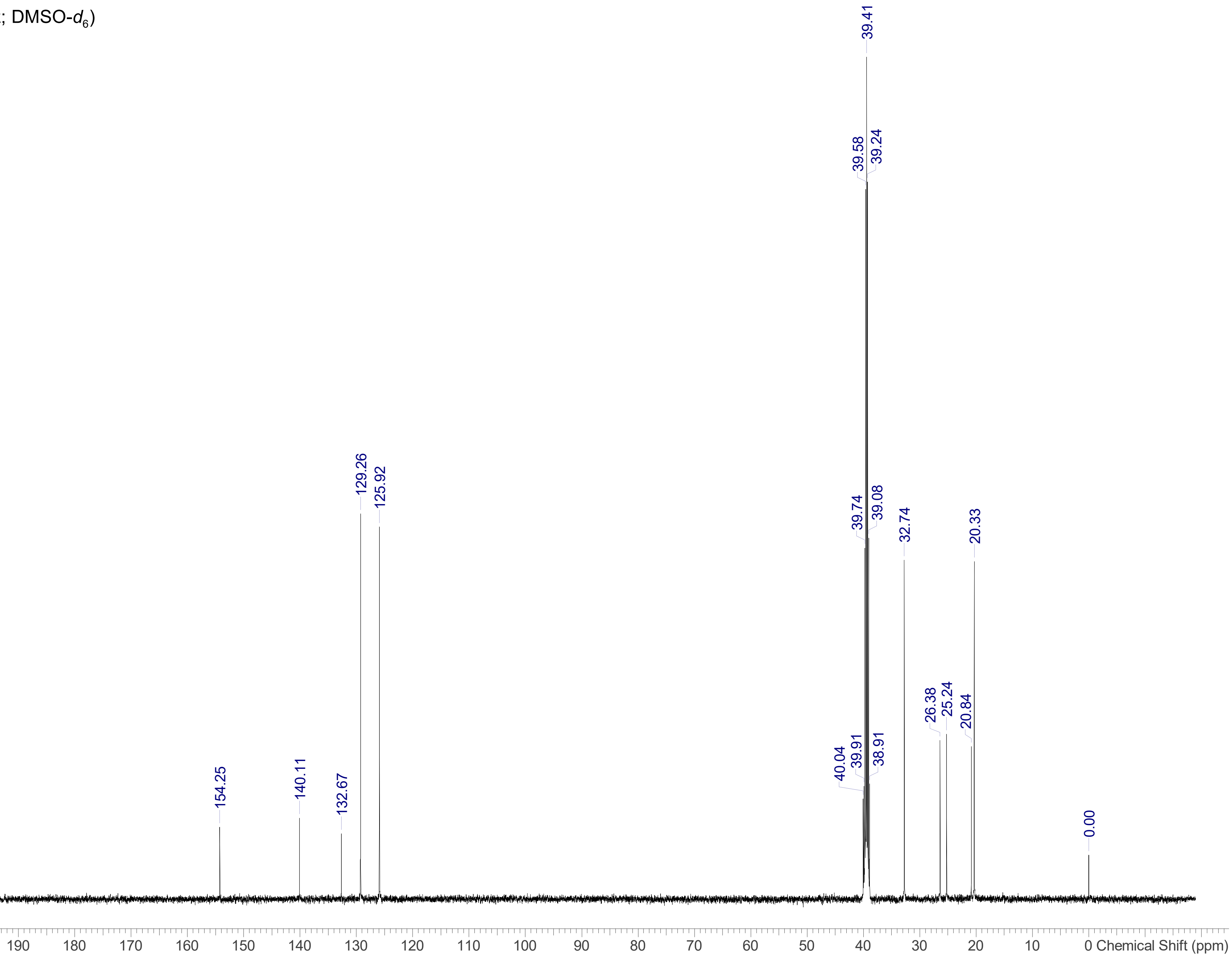

^{13}C NMR (125.7 MHz; DMSO-d_6)

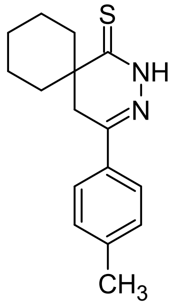

6b

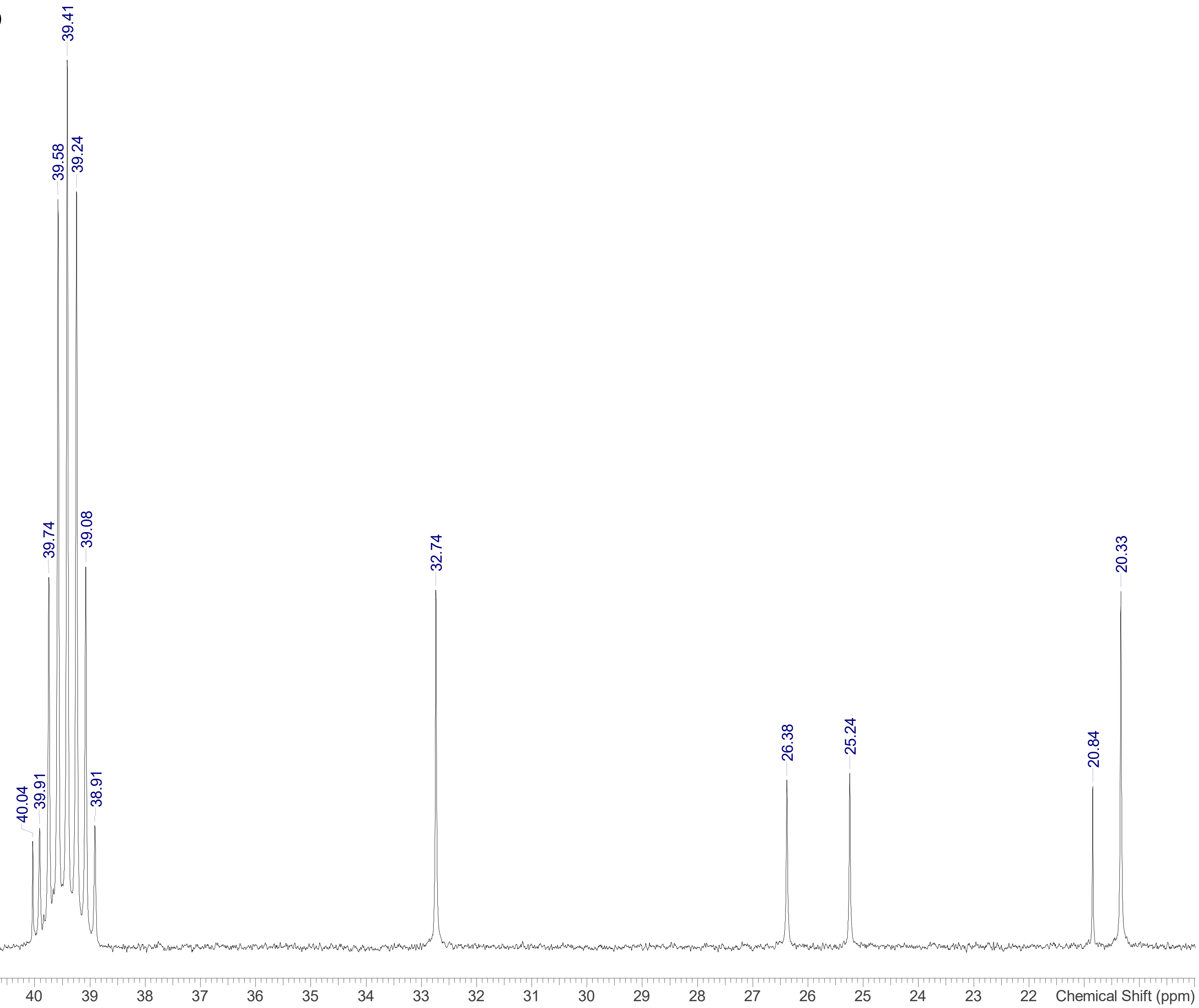

Compound **6b**

HRMS spectrum (ESI)

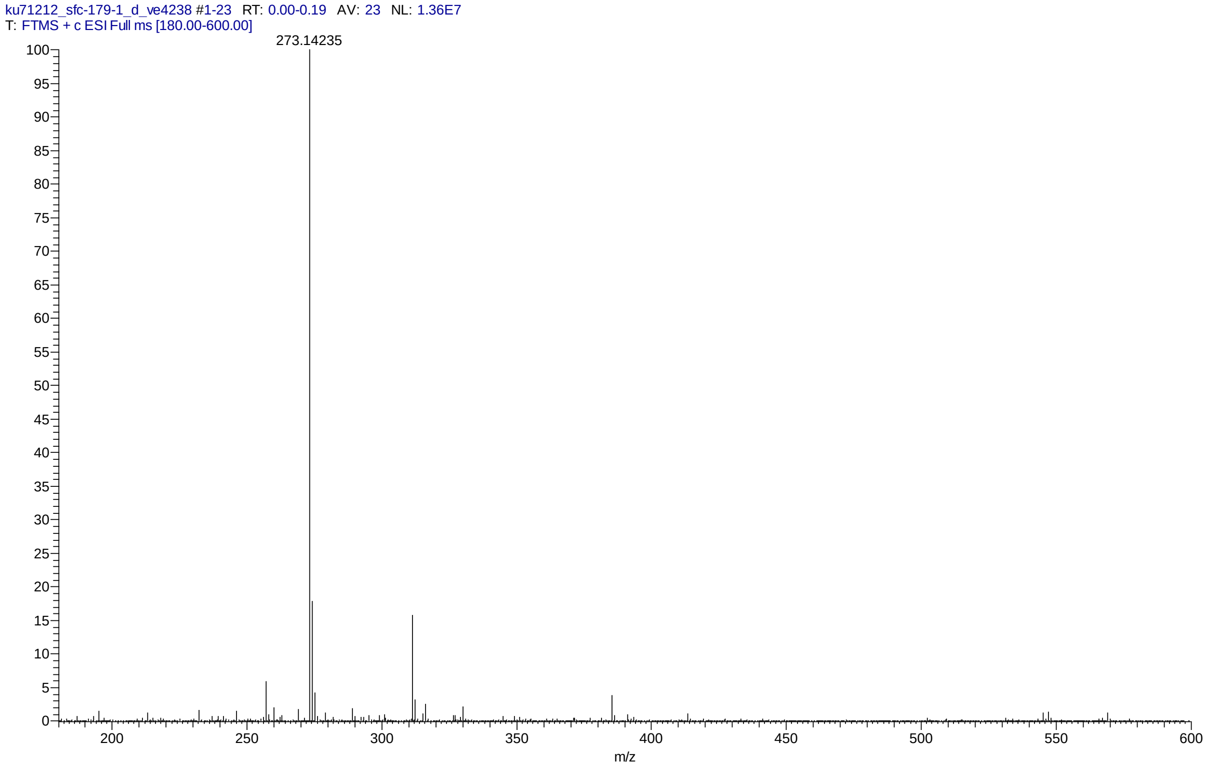

HRMS spectrum (ESI daughter ion)

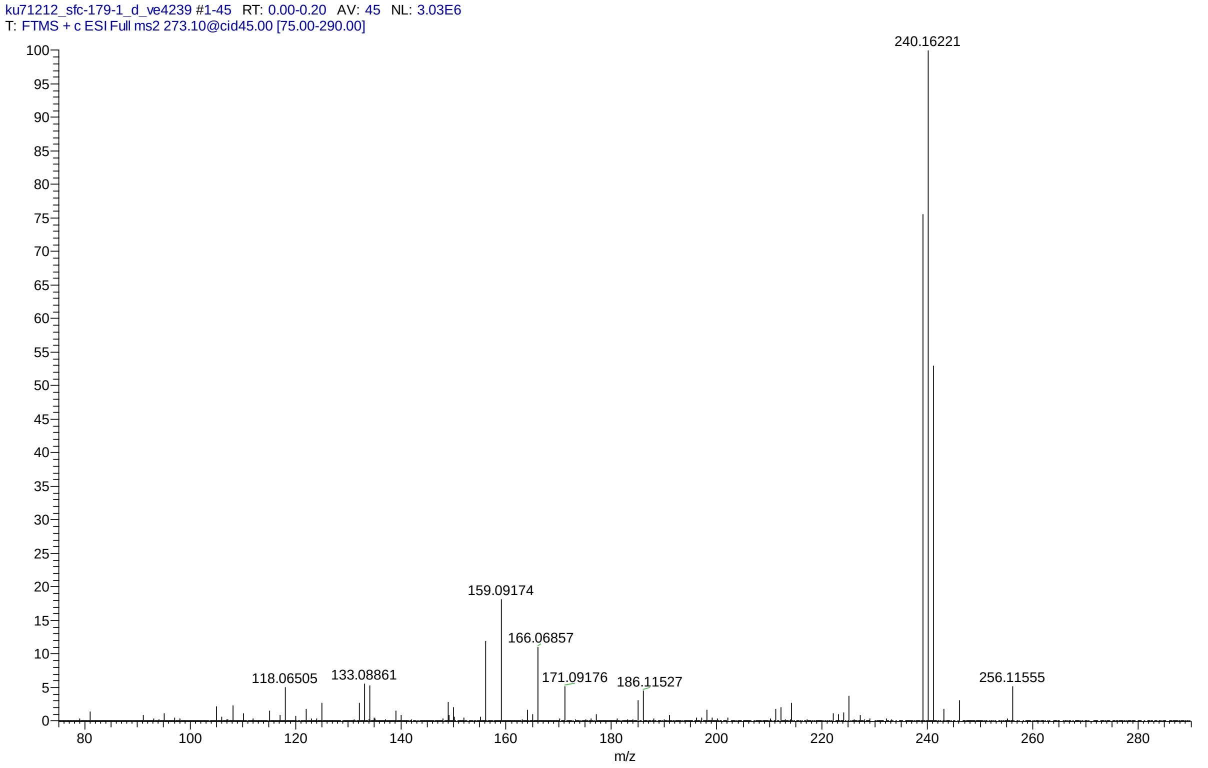

^{1}H NMR (499.9 MHz; DMSO-d_6)

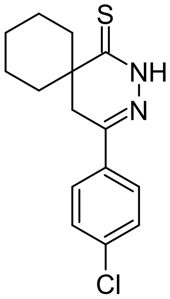

6c

4-(4-Chlorophenyl)-2,3-diazaspiro[5.5]undec-3-ene-1-thione

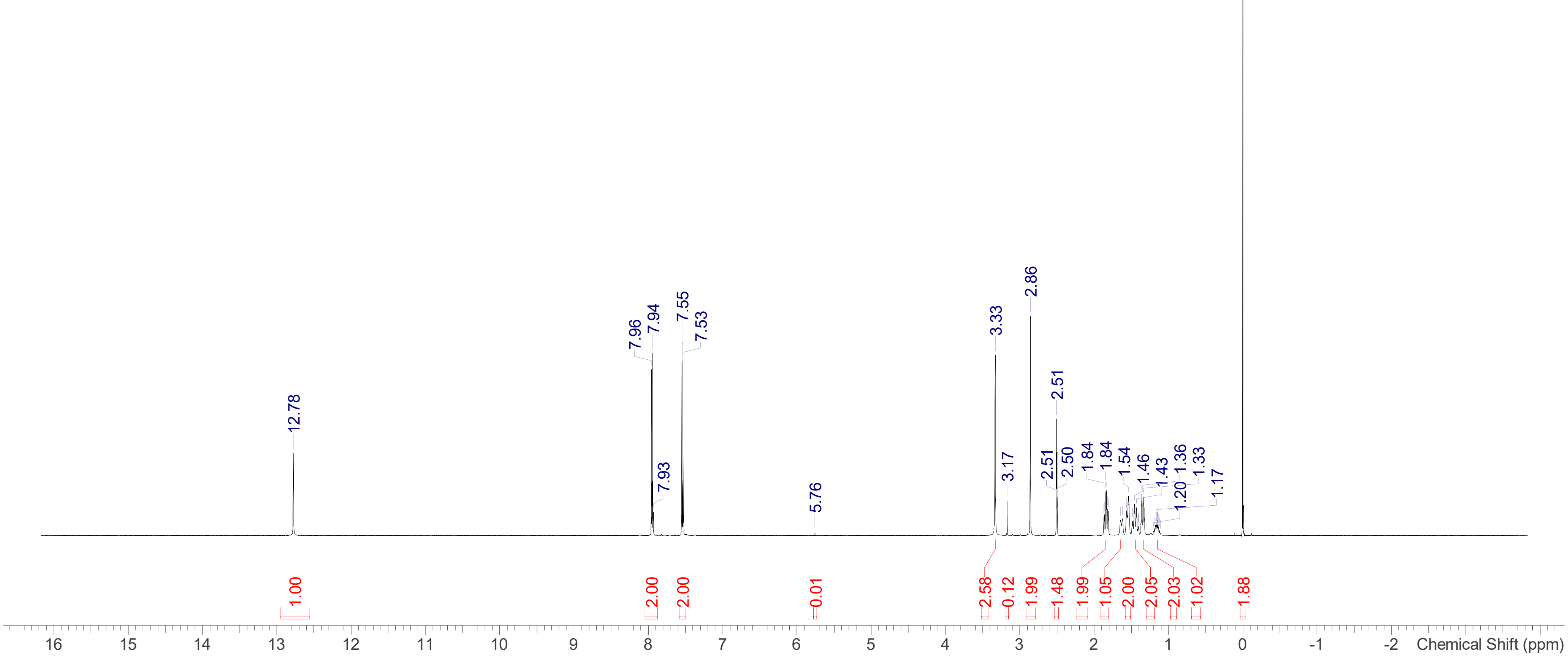

^1H NMR (499.9 MHz; DMSO-d_6)

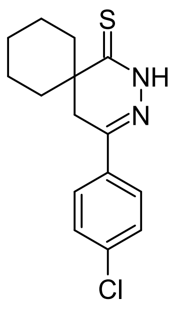

6c

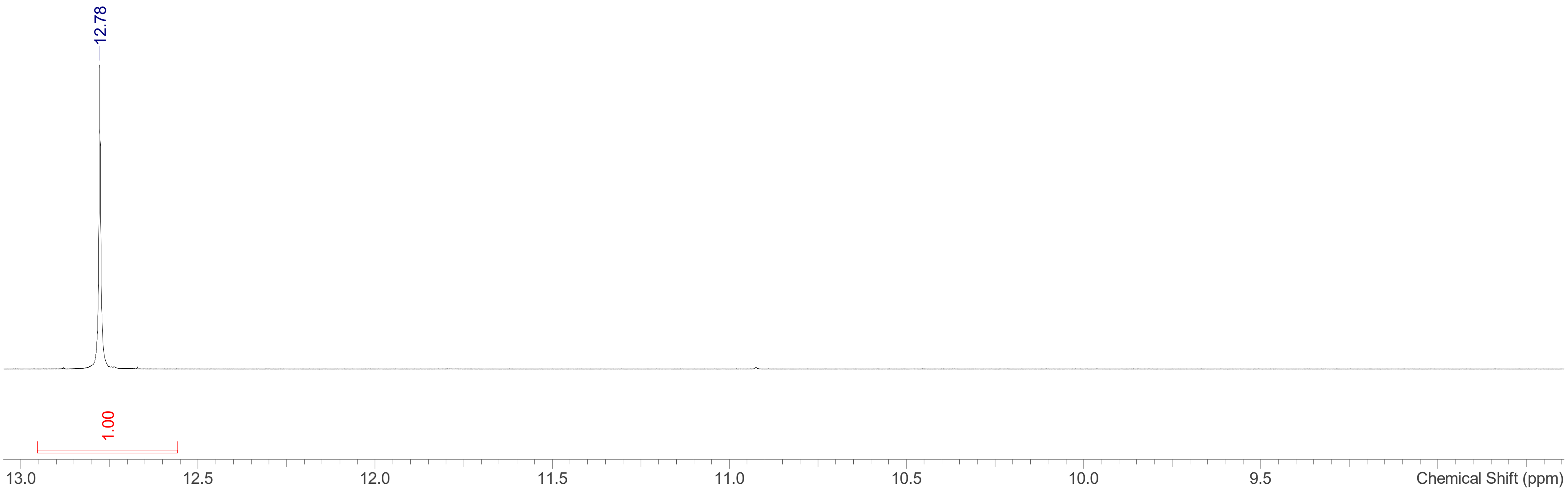

^{1}H NMR (499.9 MHz; DMSO-d_6)

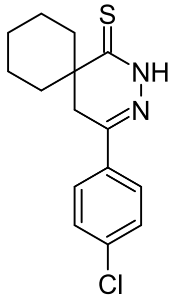

6c

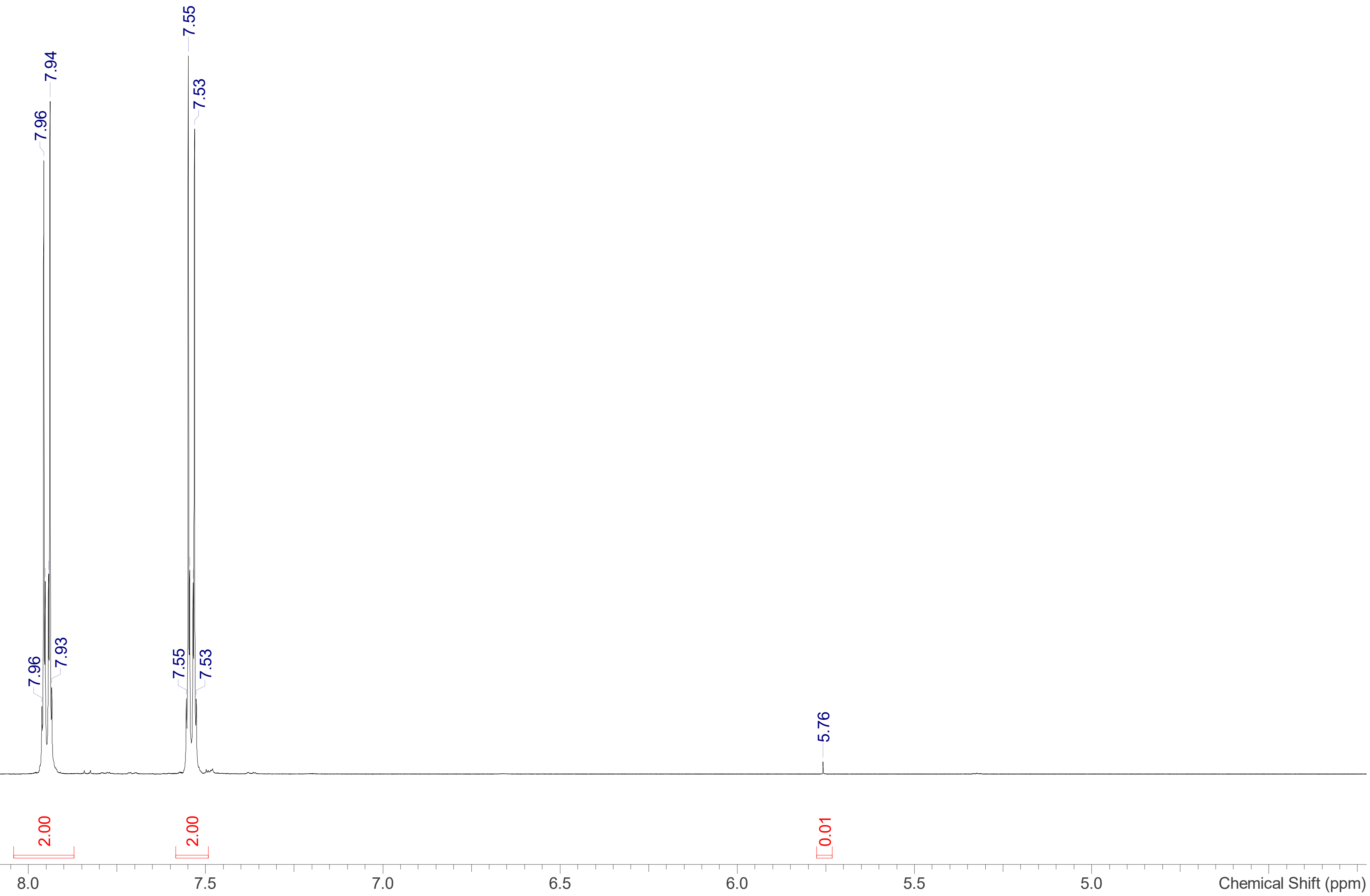

^{1}H NMR (499.9 MHz; DMSO-d_6)

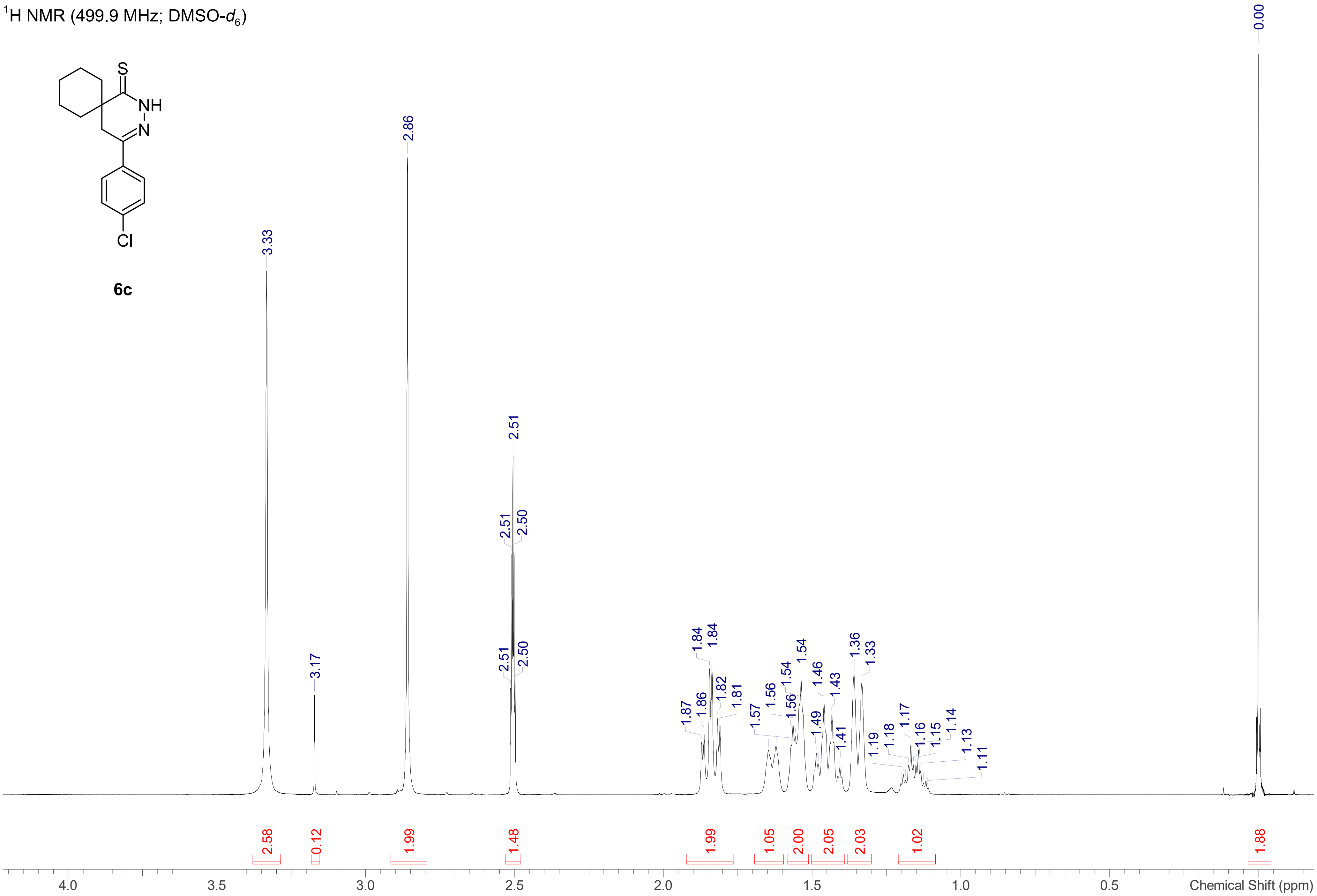

^{13}C NMR (125.7 MHz; DMSO-d_6)

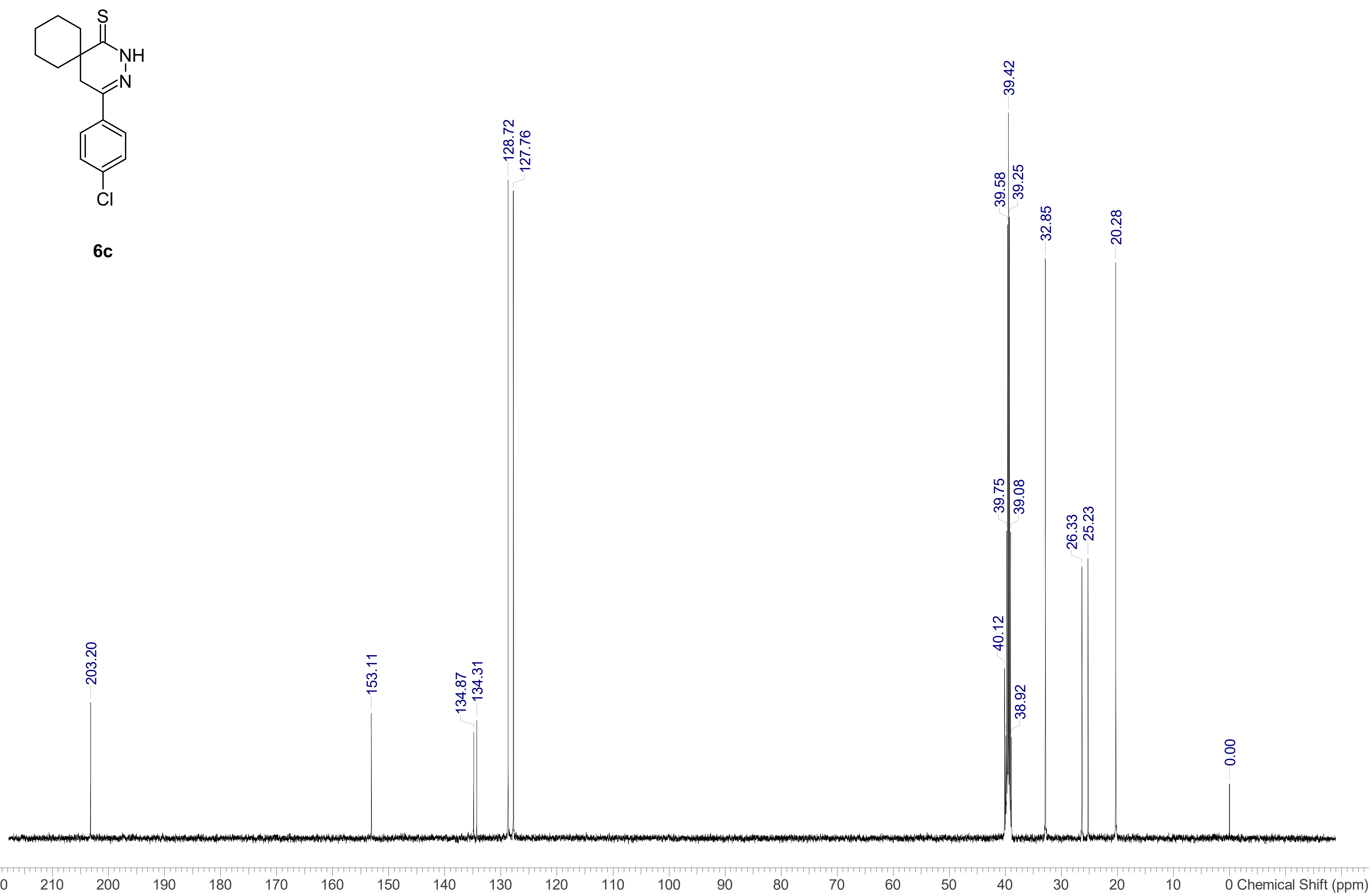

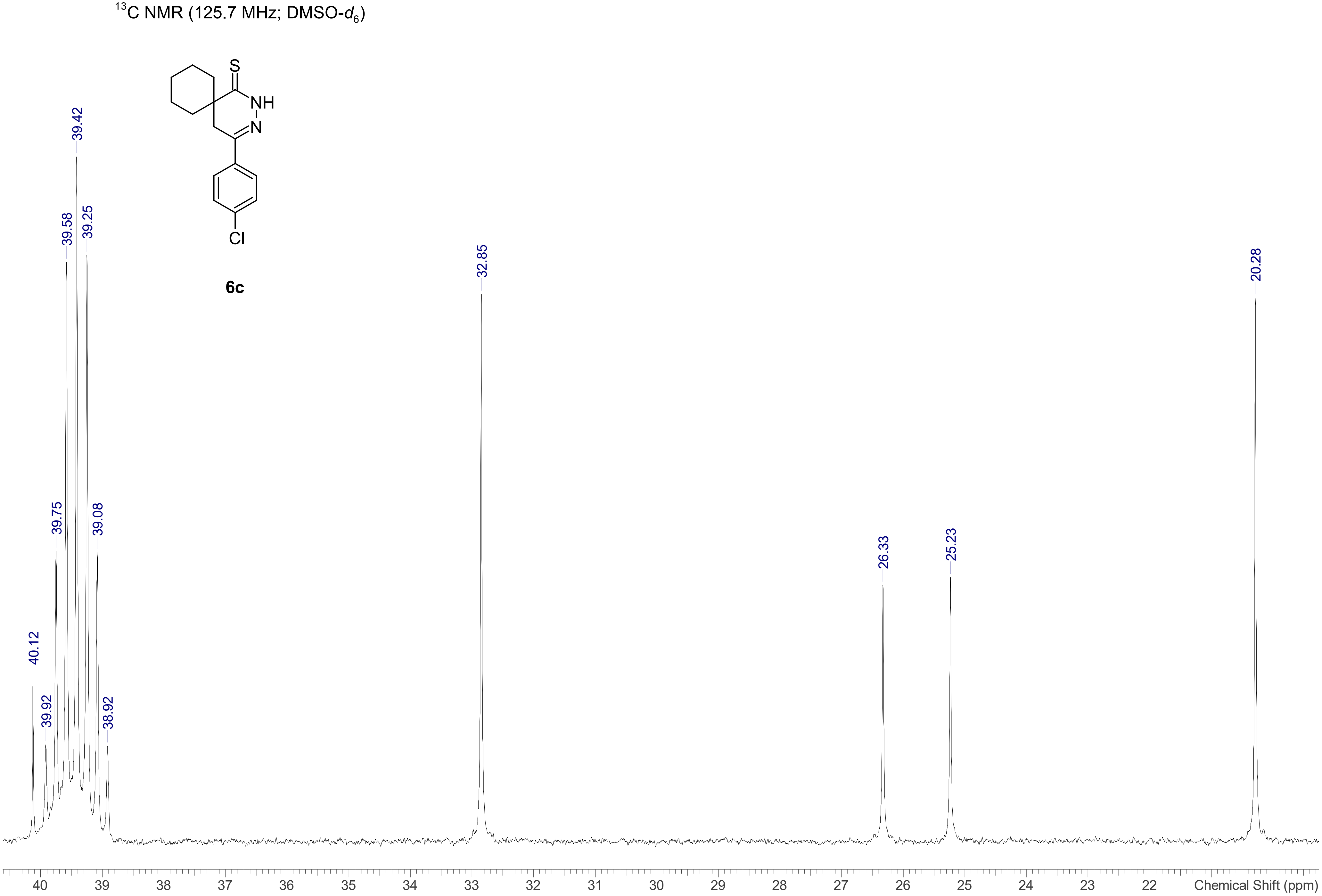
13C NMR (125.7 MHz; DMSO-d6)
S
NH
N
Cl
6c
40.12
39.92
39.75
39.58
39.42
39.25
39.08
38.92
32.85
26.33
25.23
20.28
40
39
38
37
36
35
34
33
32
31
30
29
28
27
26
25
24
23
22
Chemical Shift (ppm)

Compound **6c**

HRMS spectrum (ESI)

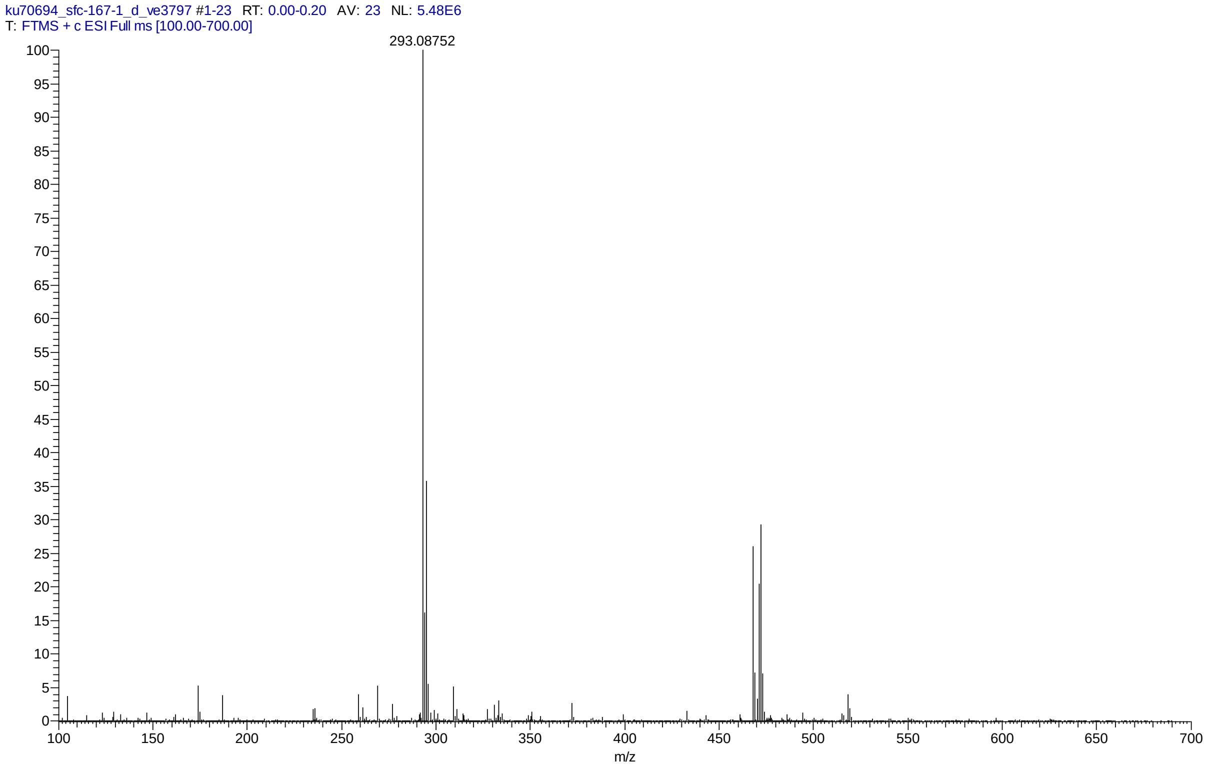

HRMS spectrum (ESI daughter ion)

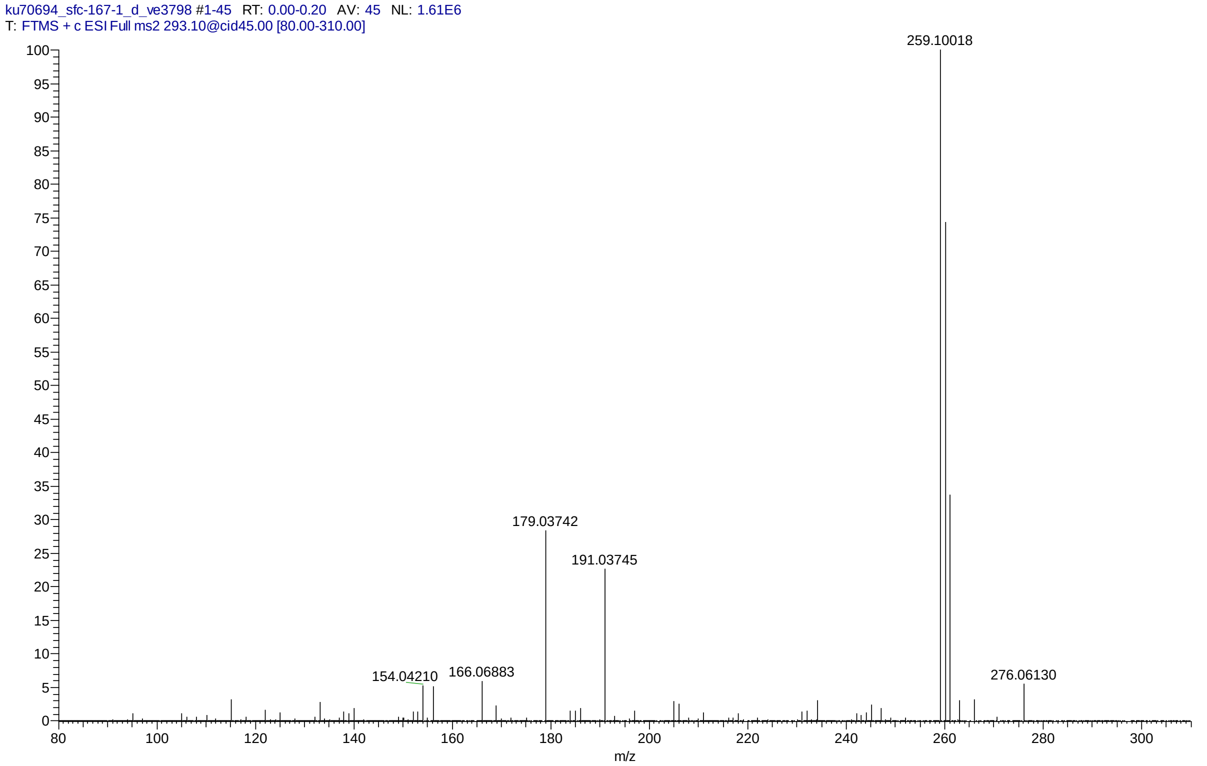

^{1}H NMR (499.9 MHz; $CDCl_3$)

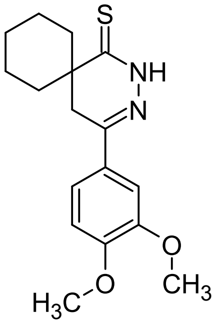

6d

4-(3,4-Dimethoxyphenyl)-2,3-diazaspiro[5.5]undec-3-ene-1-thione

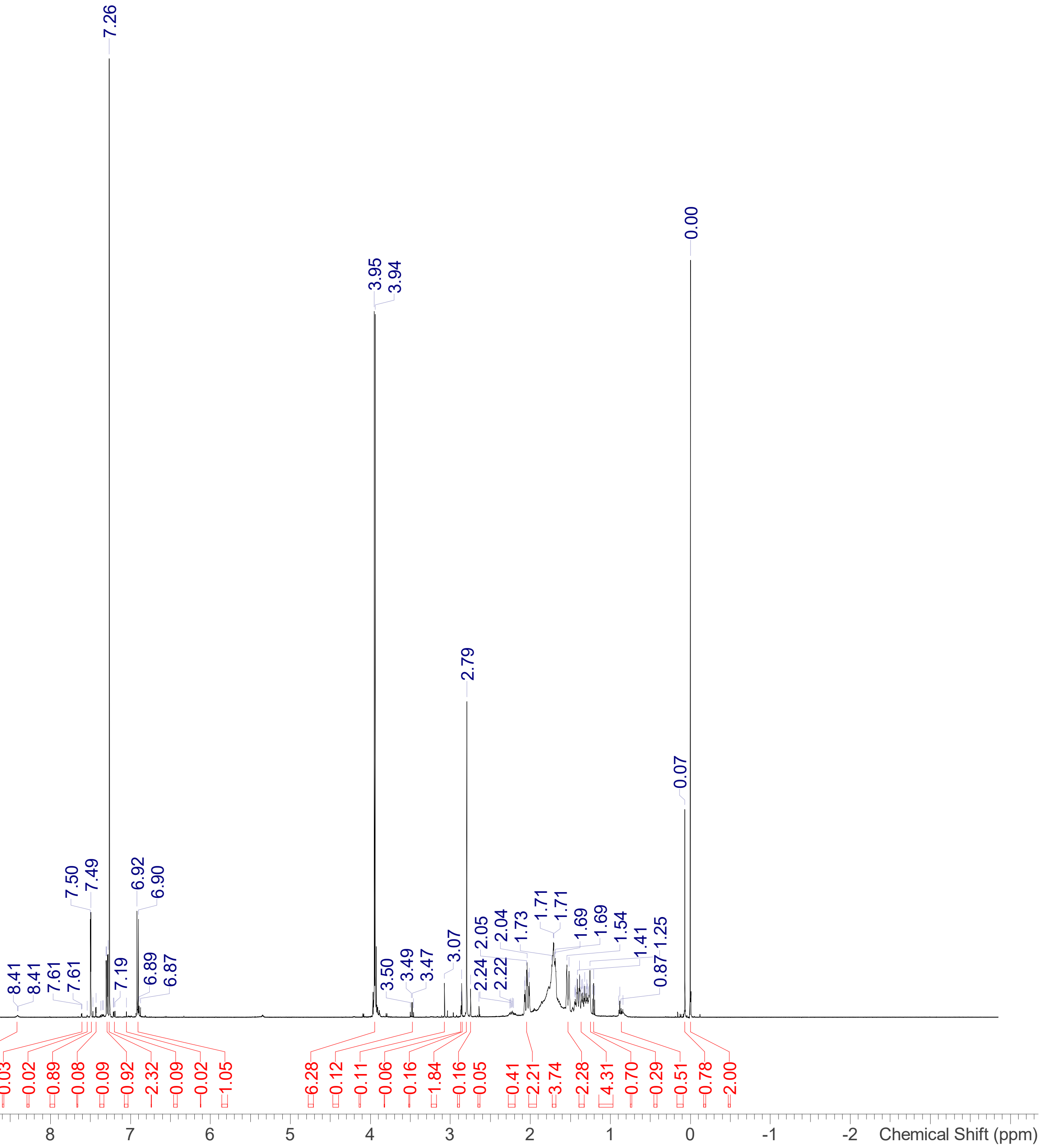

^{1}H NMR (499.9 MHz; $CDCl_3$)

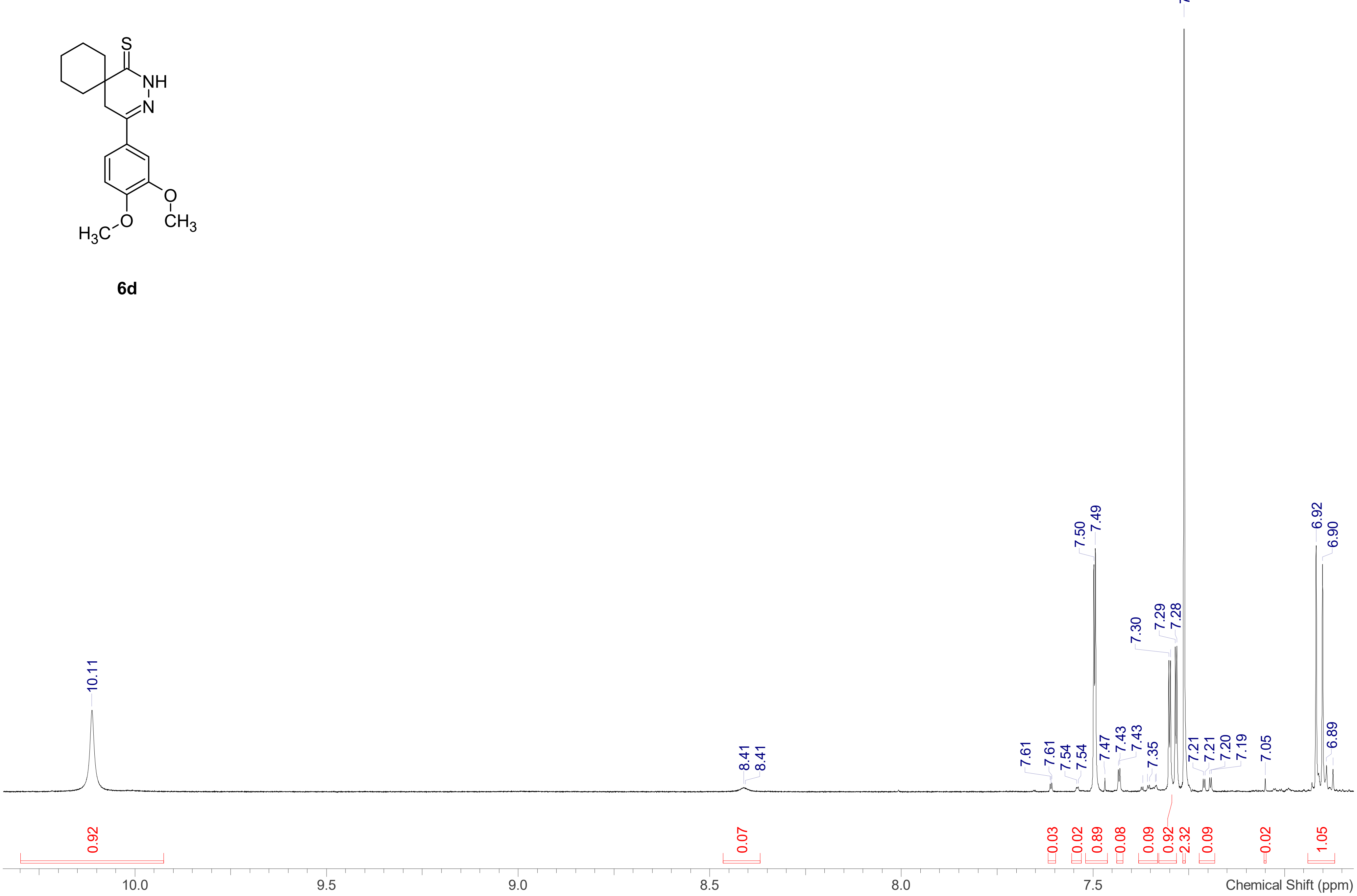

^1H NMR (499.9 MHz; $CDCl_3$)

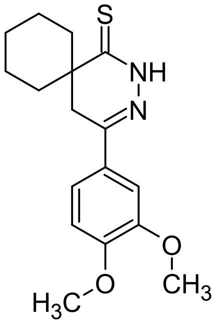

6d

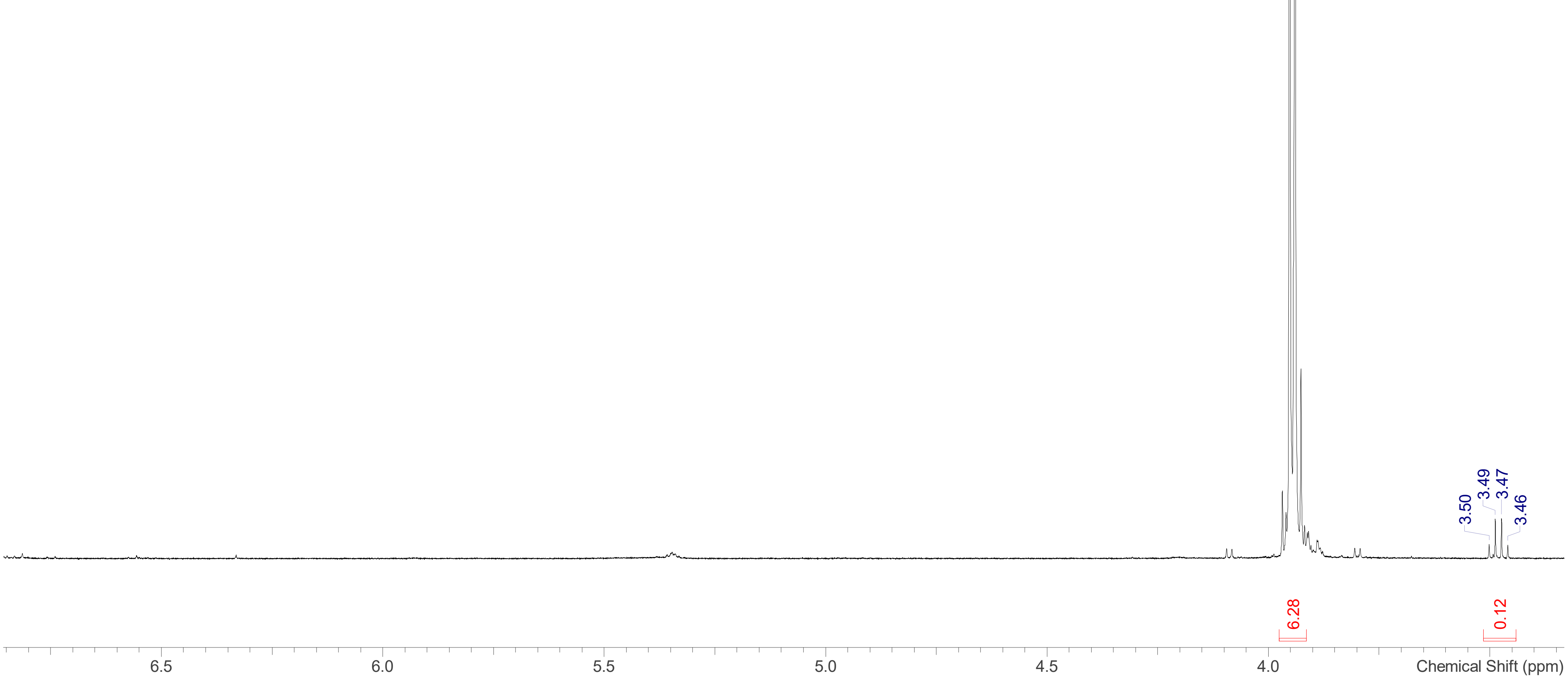

^{1}H NMR (499.9 MHz; $CDCl_3$)

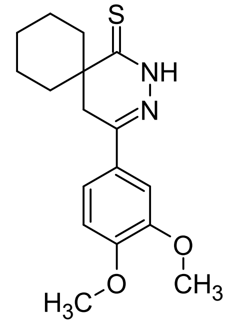

6d

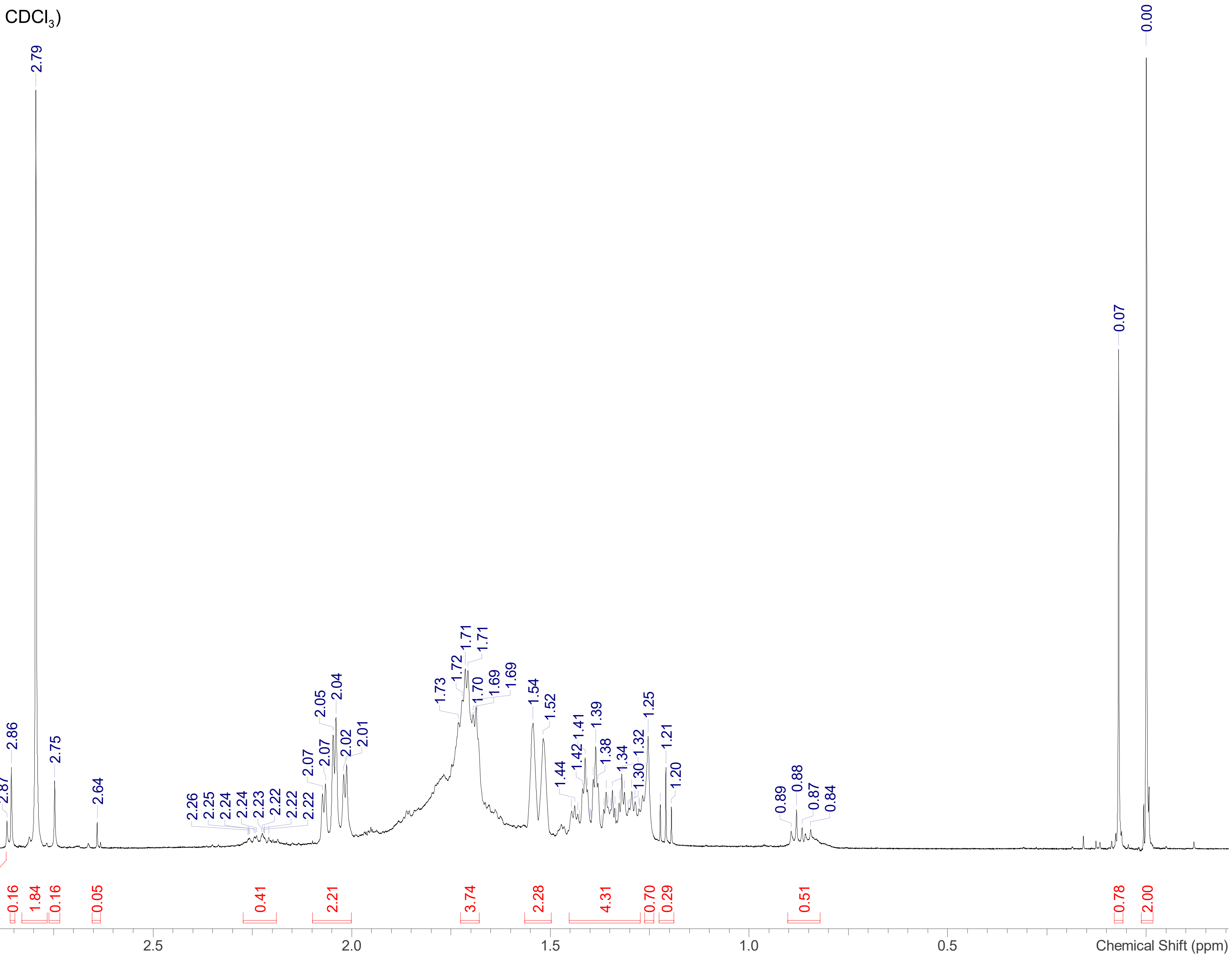

^{13}C NMR (125.7 MHz; $CDCl_3$)

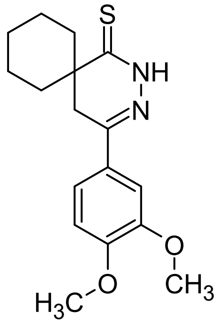

6d

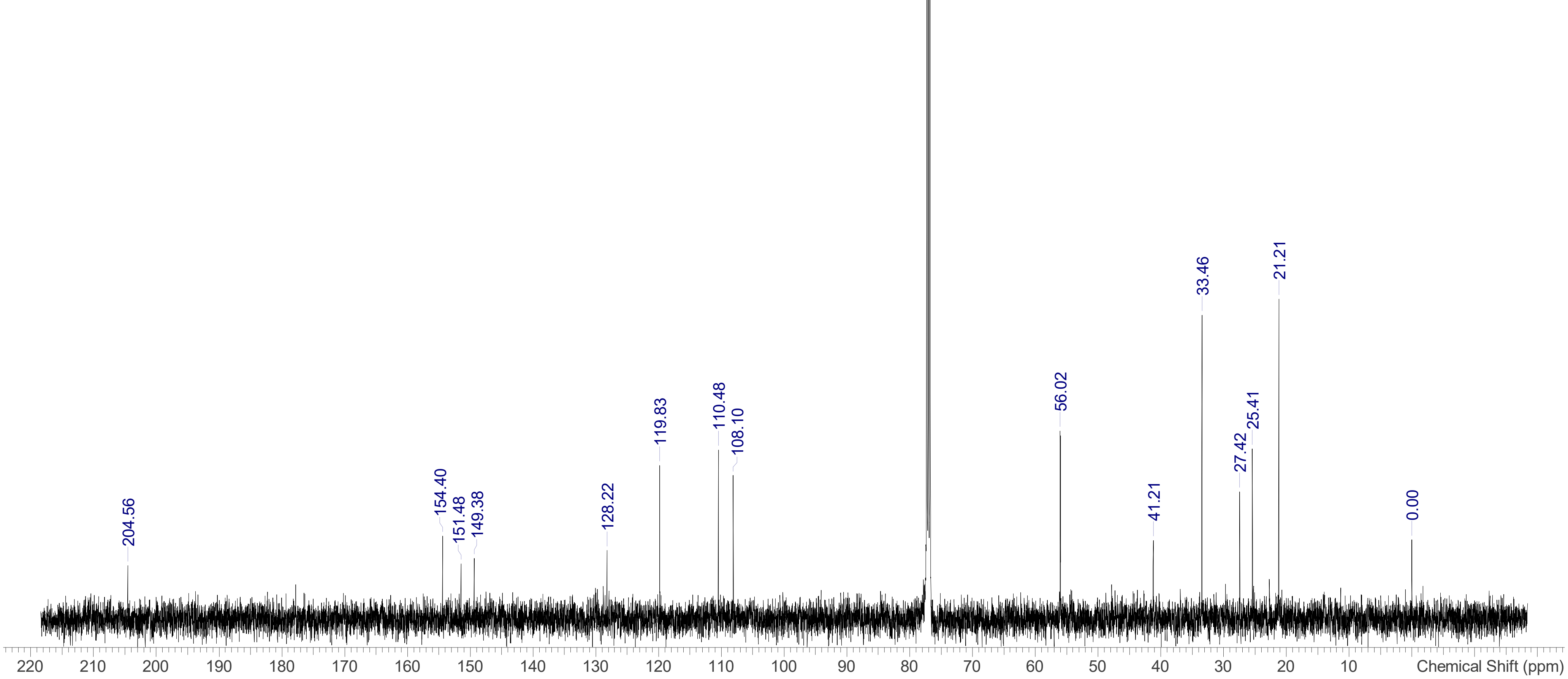

Compound **6d**

HRMS spectrum (ESI)

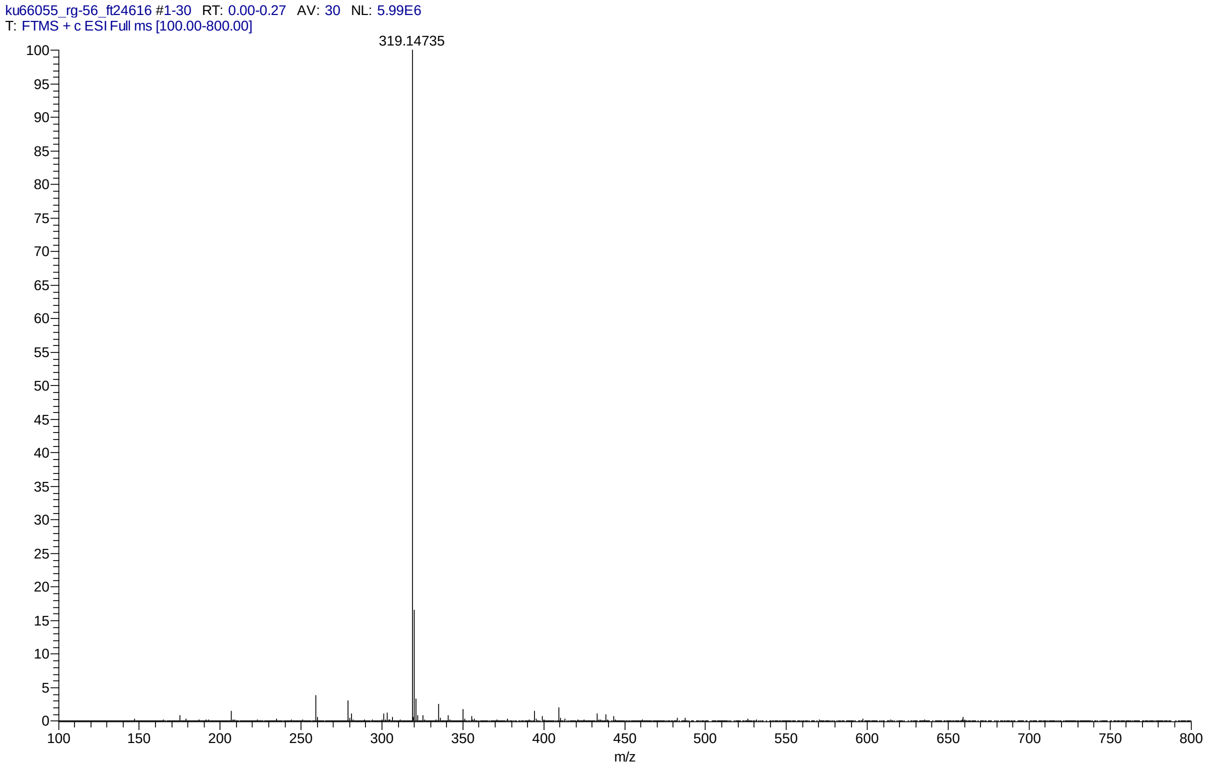

HRMS spectrum (ESI daughter ion)

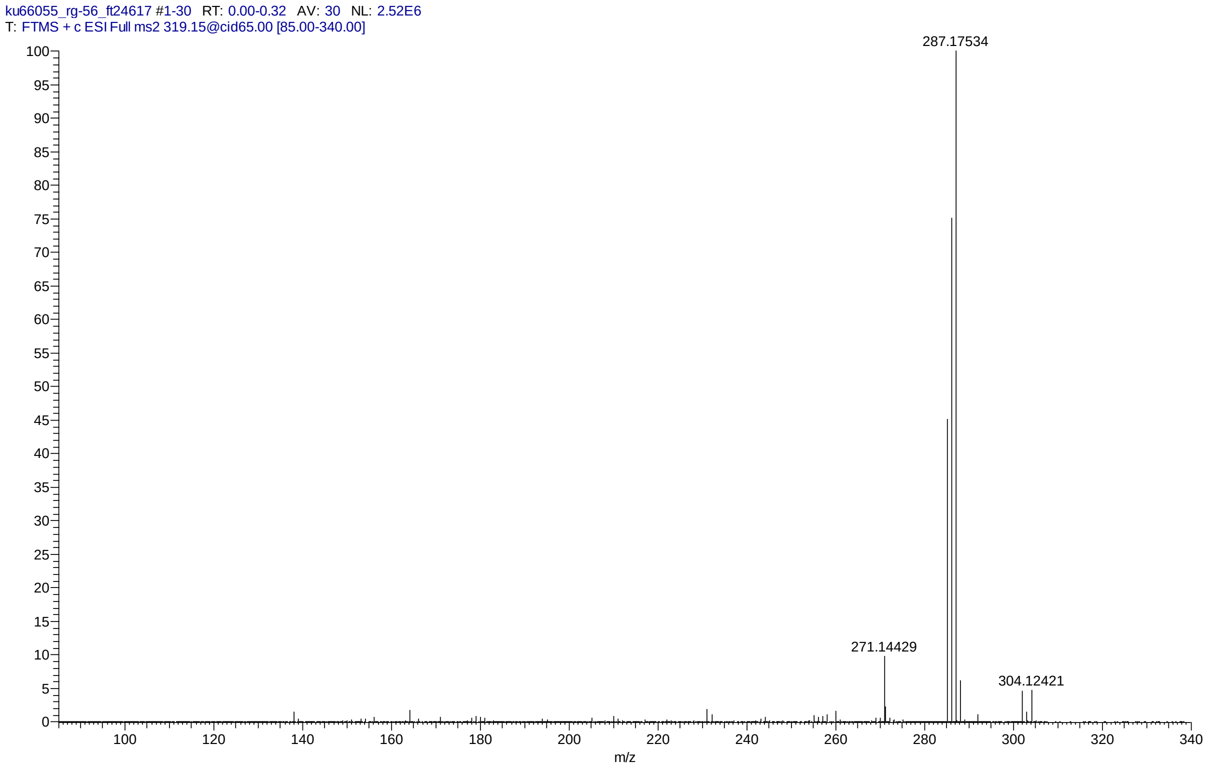

^{1}H NMR (399.8 MHz; $CDCl_3$)

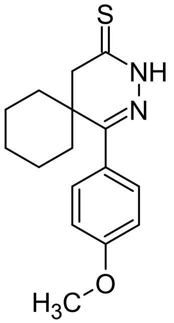

6e

1-(4-Methoxyphenyl)-2,3-diazaspiro[5.5]undec-1-ene-4-thione

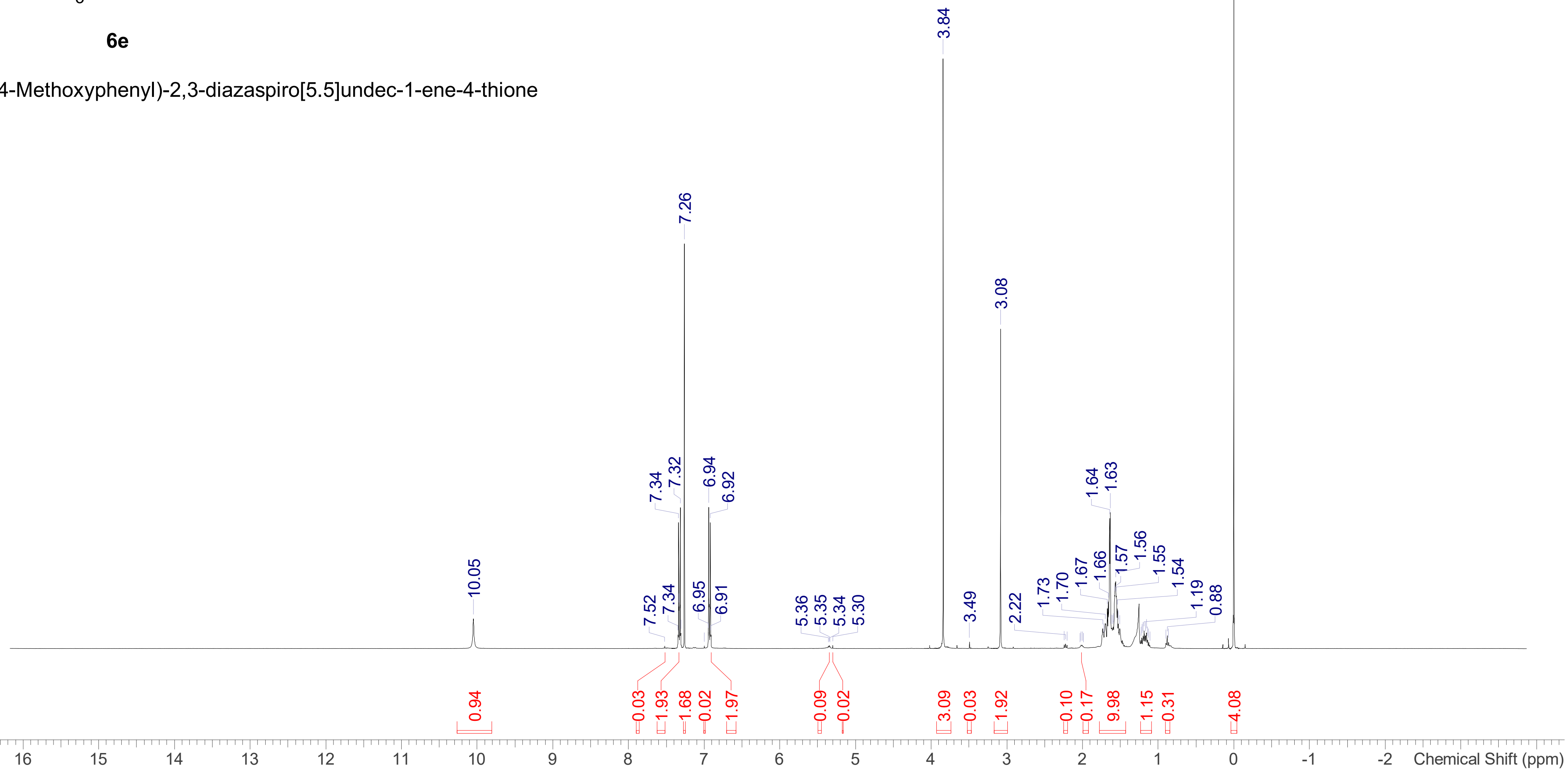

^{1}H NMR (399.8 MHz; $CDCl_3$)

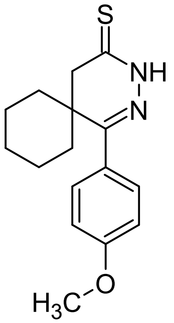

6e

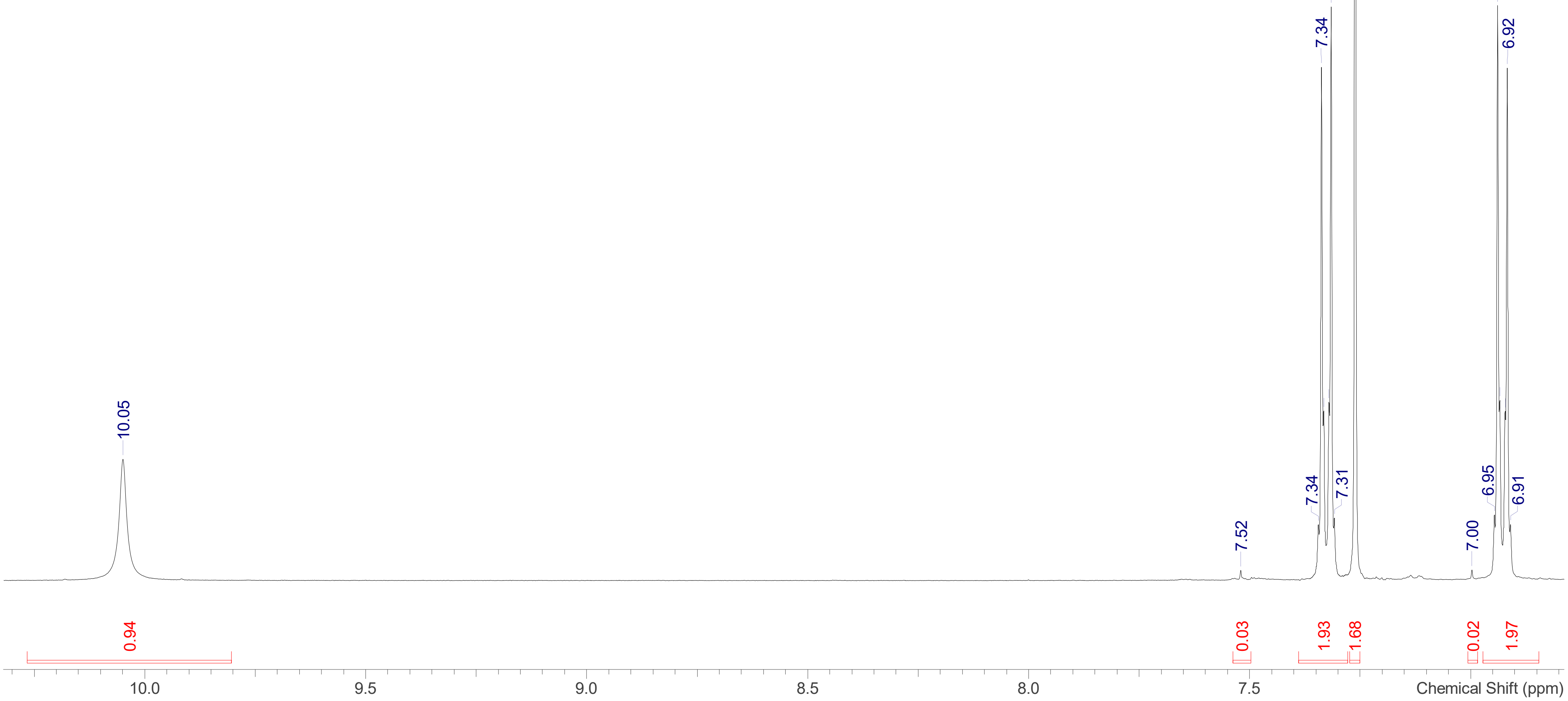

^{1}H NMR (399.8 MHz; $CDCl_3$)

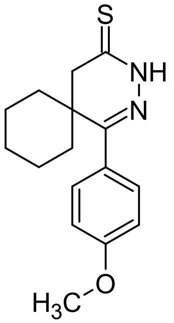

6e

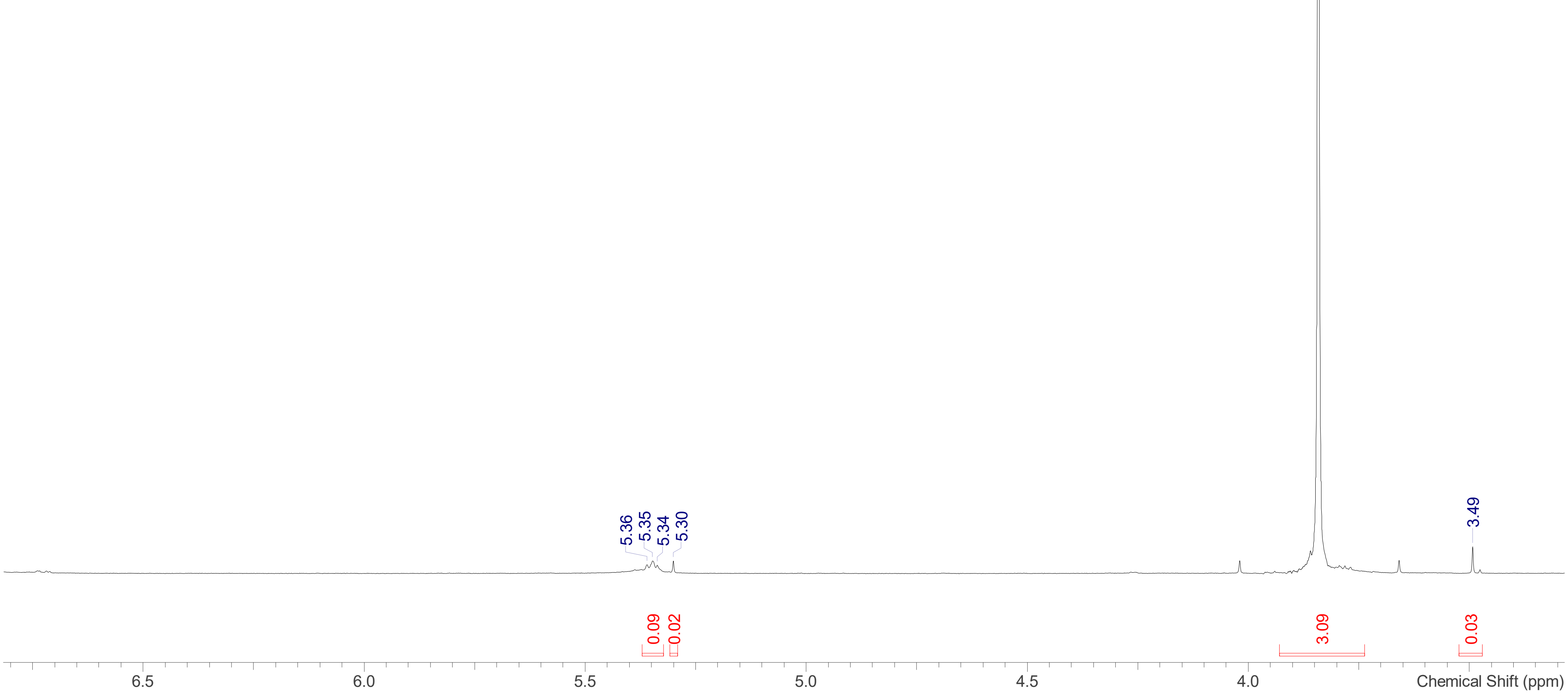

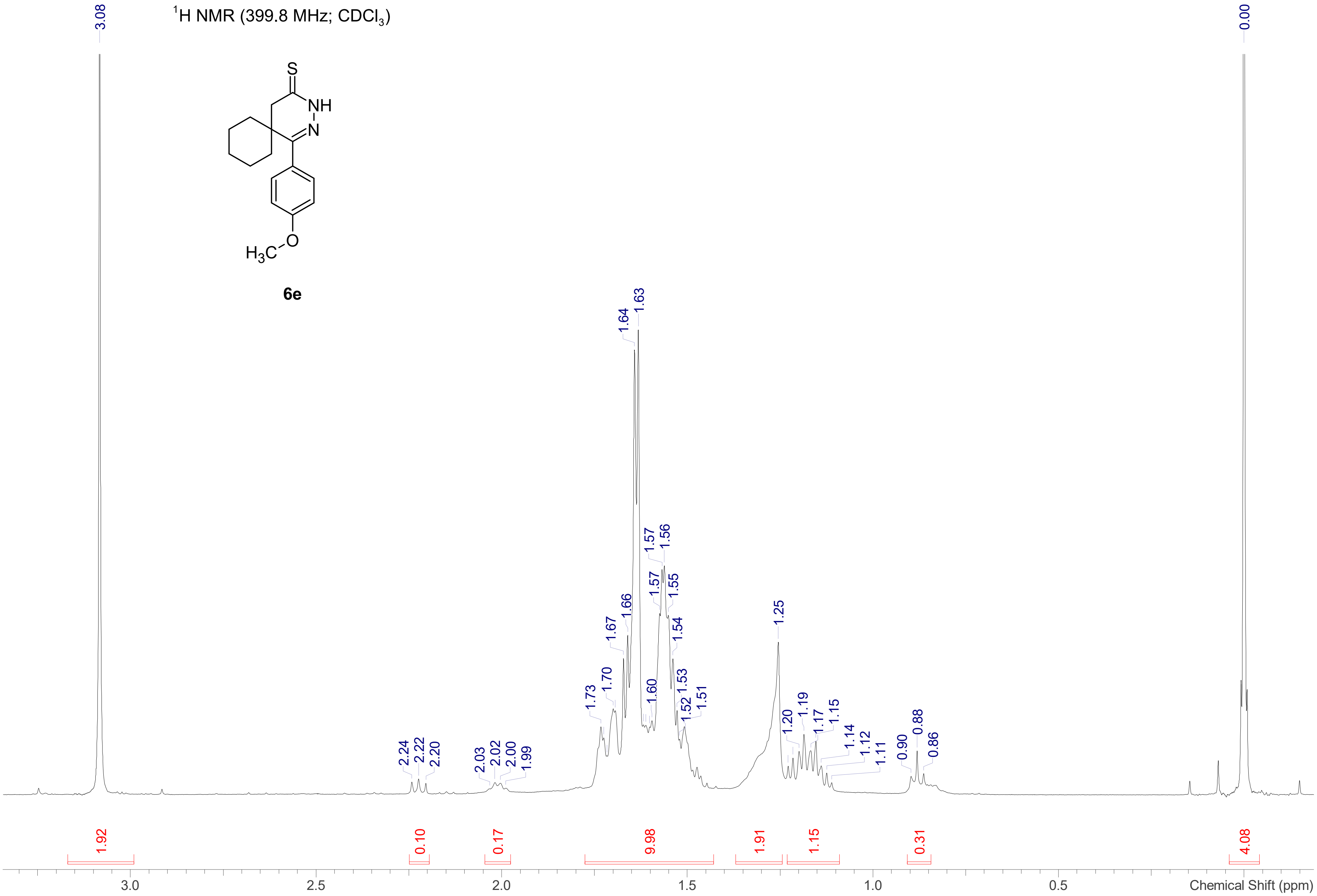

1H NMR (399.8 MHz; CDCl3)
S
NH
N
H3C
O
6e
3.08
0.00
1.64
1.63
1.57
1.56
1.57
1.55
1.66
1.67
1.54
1.73
1.70
1.60
1.53
1.52
1.51
1.25
1.20
1.19
1.17
1.15
1.14
1.12
1.11
0.90
0.88
0.86
2.24
2.22
2.20
2.03
2.02
2.00
1.99
1.92
0.10
0.17
9.98
1.91
1.15
0.31
4.08
3.0
2.5
2.0
1.5
1.0
0.5
Chemical Shift (ppm)

^{13}C NMR (100.5 MHz; $CDCl_3$)

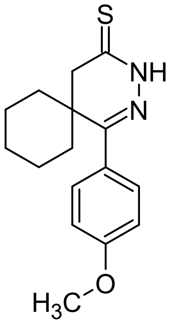

6e

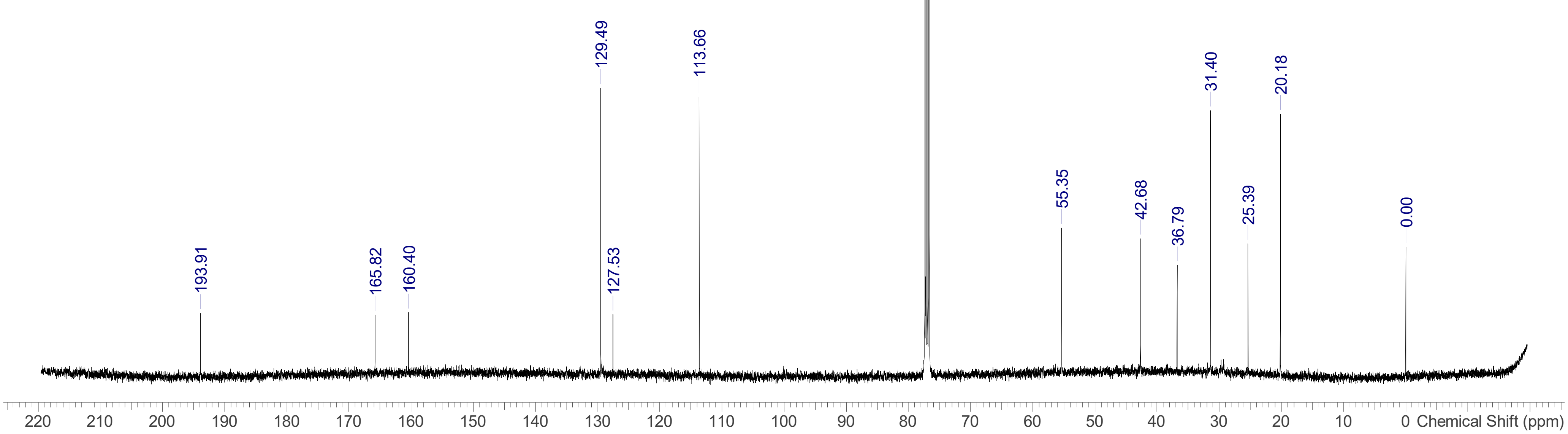

Compound **6e**

HRMS spectrum (ESI)

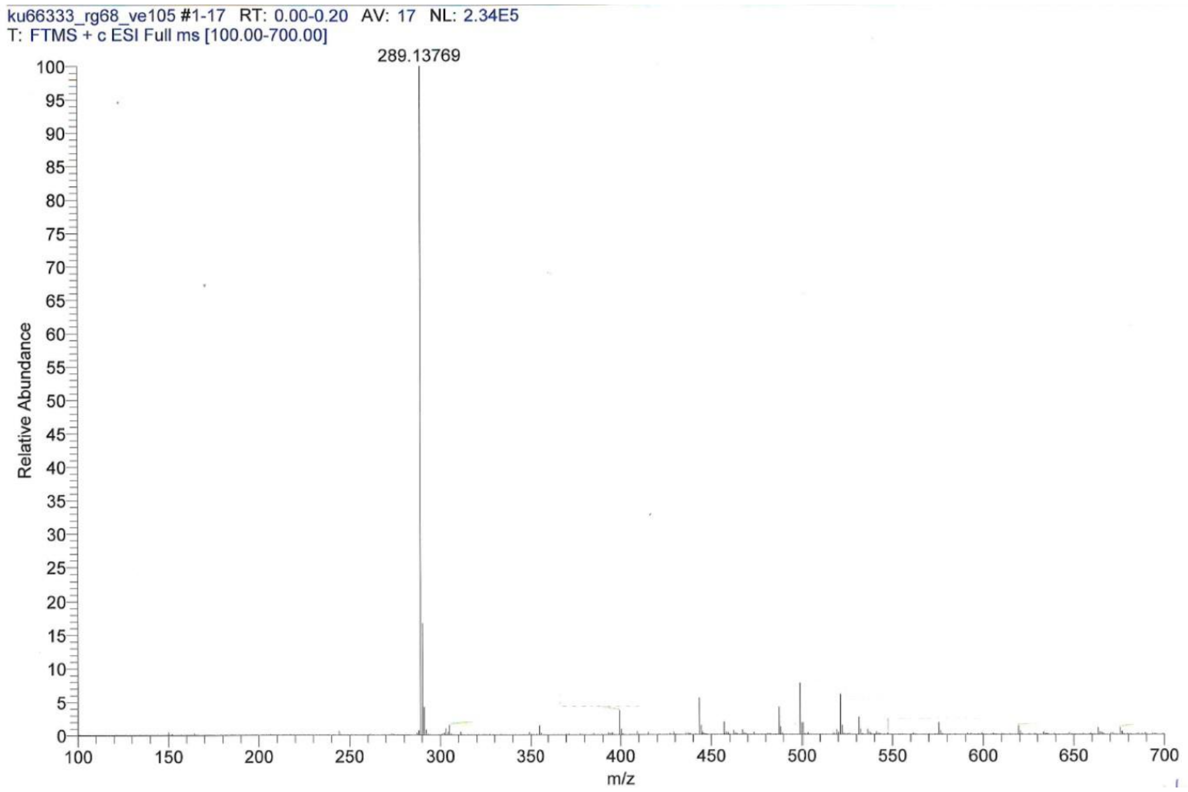

HRMS spectrum (ESI daughter ion)

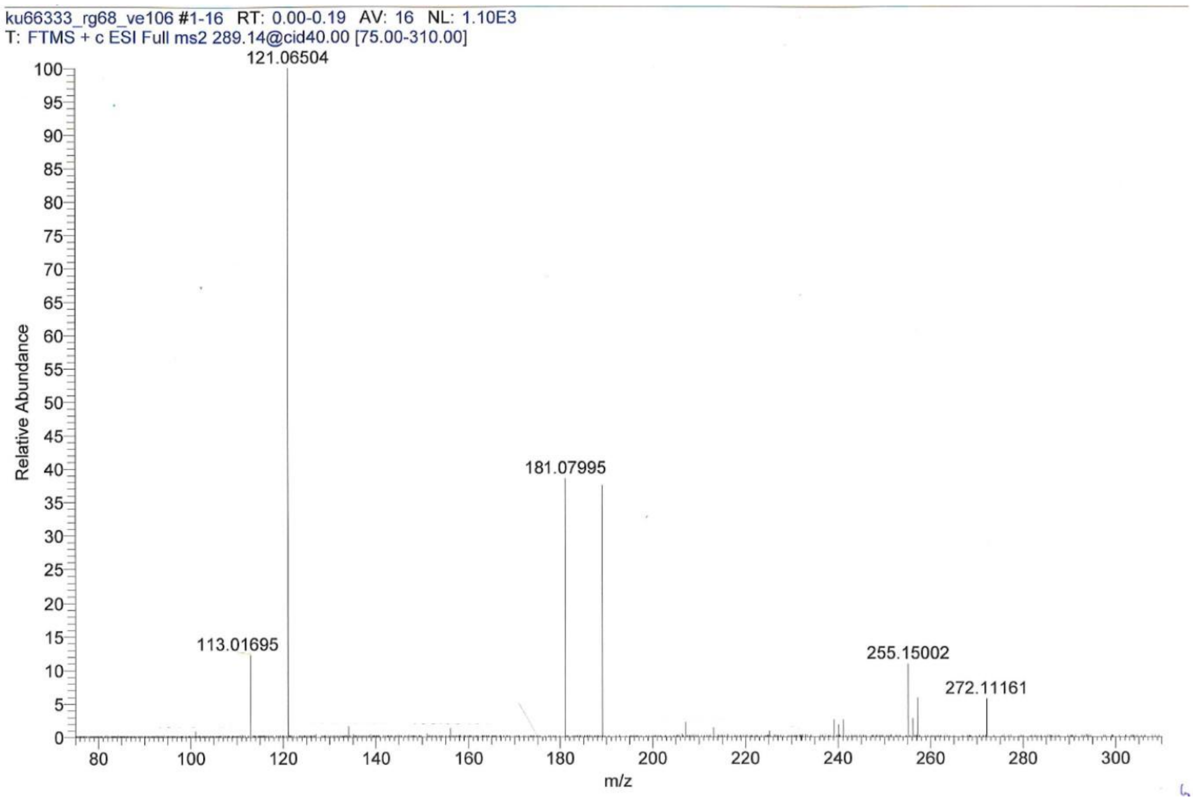

^{1}H NMR (499.9 MHz; $CDCl_3$)

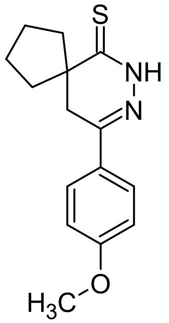

7a

9-(4-Methoxyphenyl)-7,8-diazaspiro[4.5]dec-8-ene-6-thione

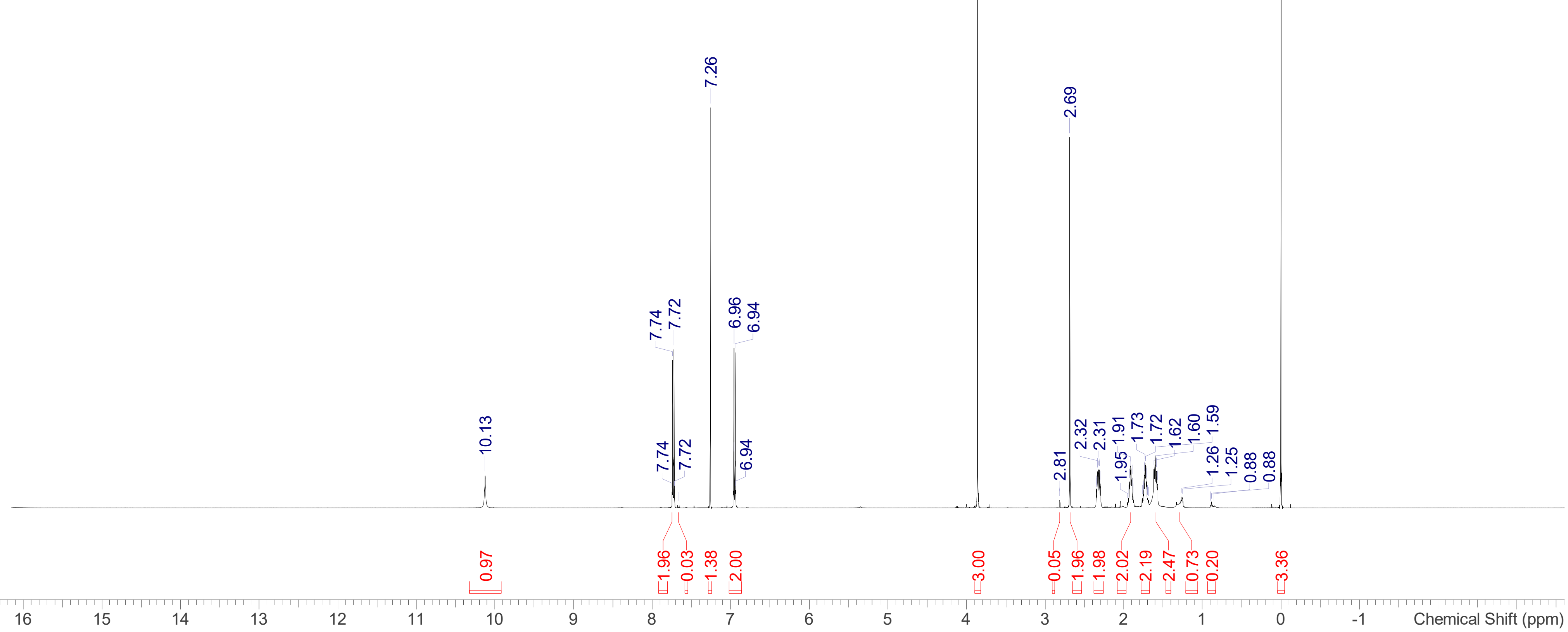

^1H NMR (499.9 MHz; $CDCl_3$)

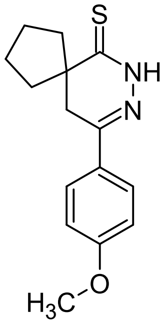

7a

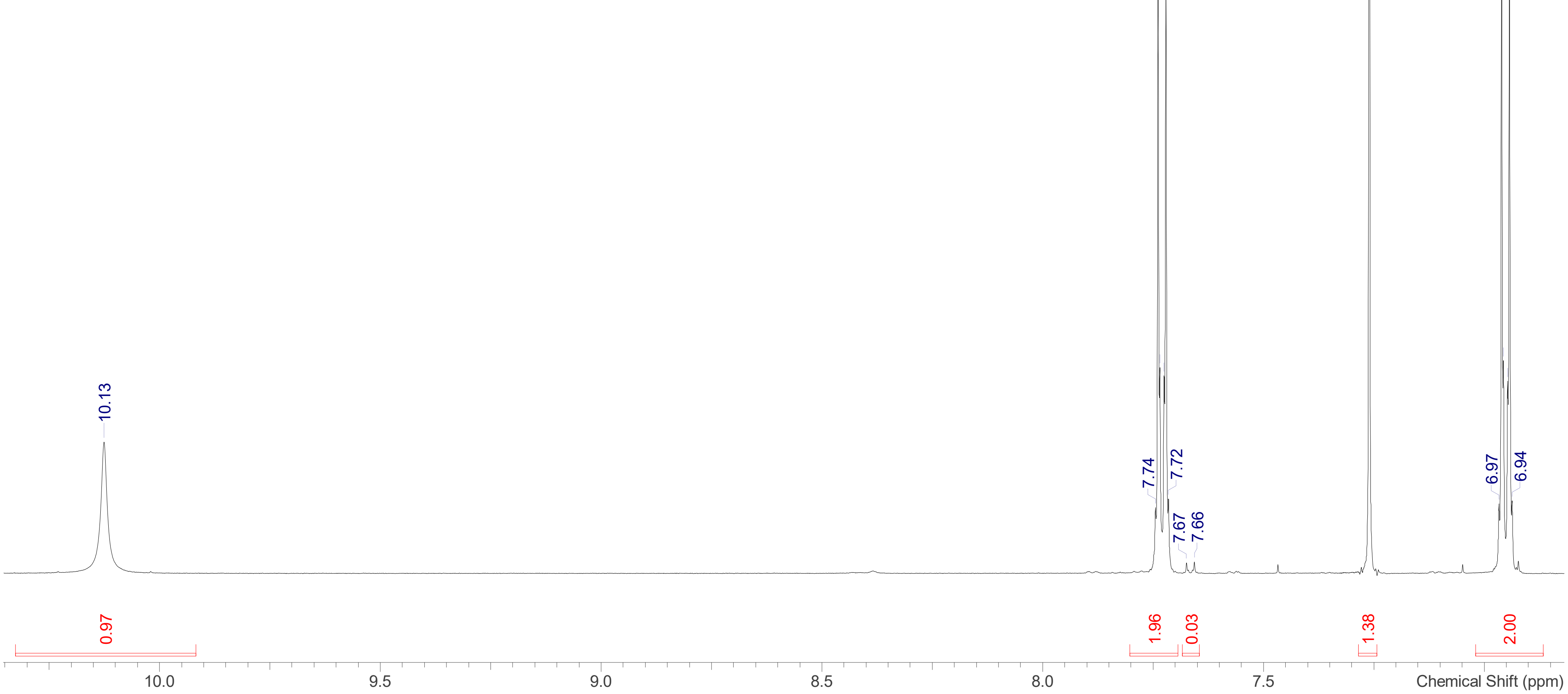

^1H NMR (499.9 MHz; $CDCl_3$)

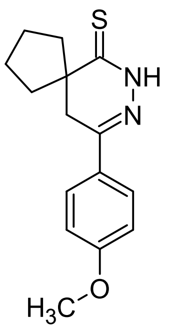

7a

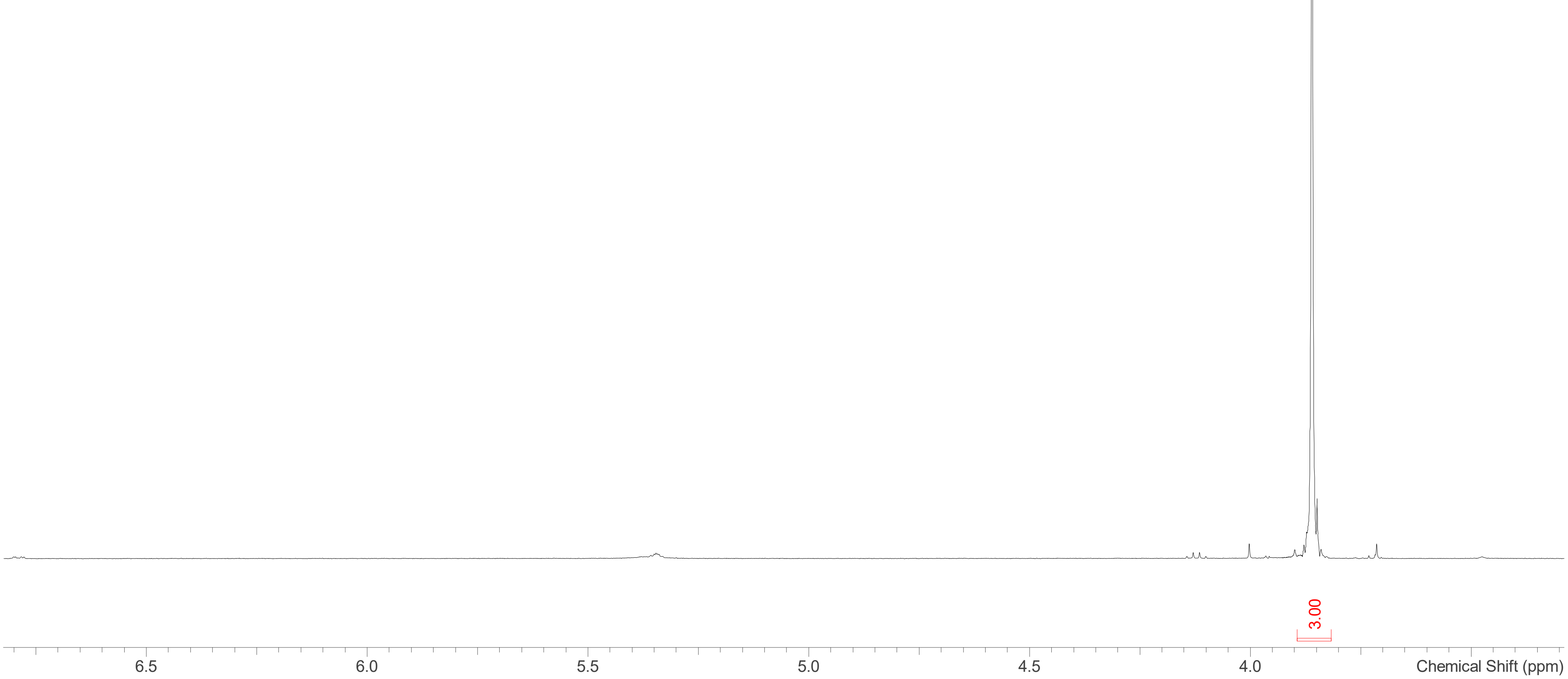

^1H NMR (499.9 MHz; $CDCl_3$)

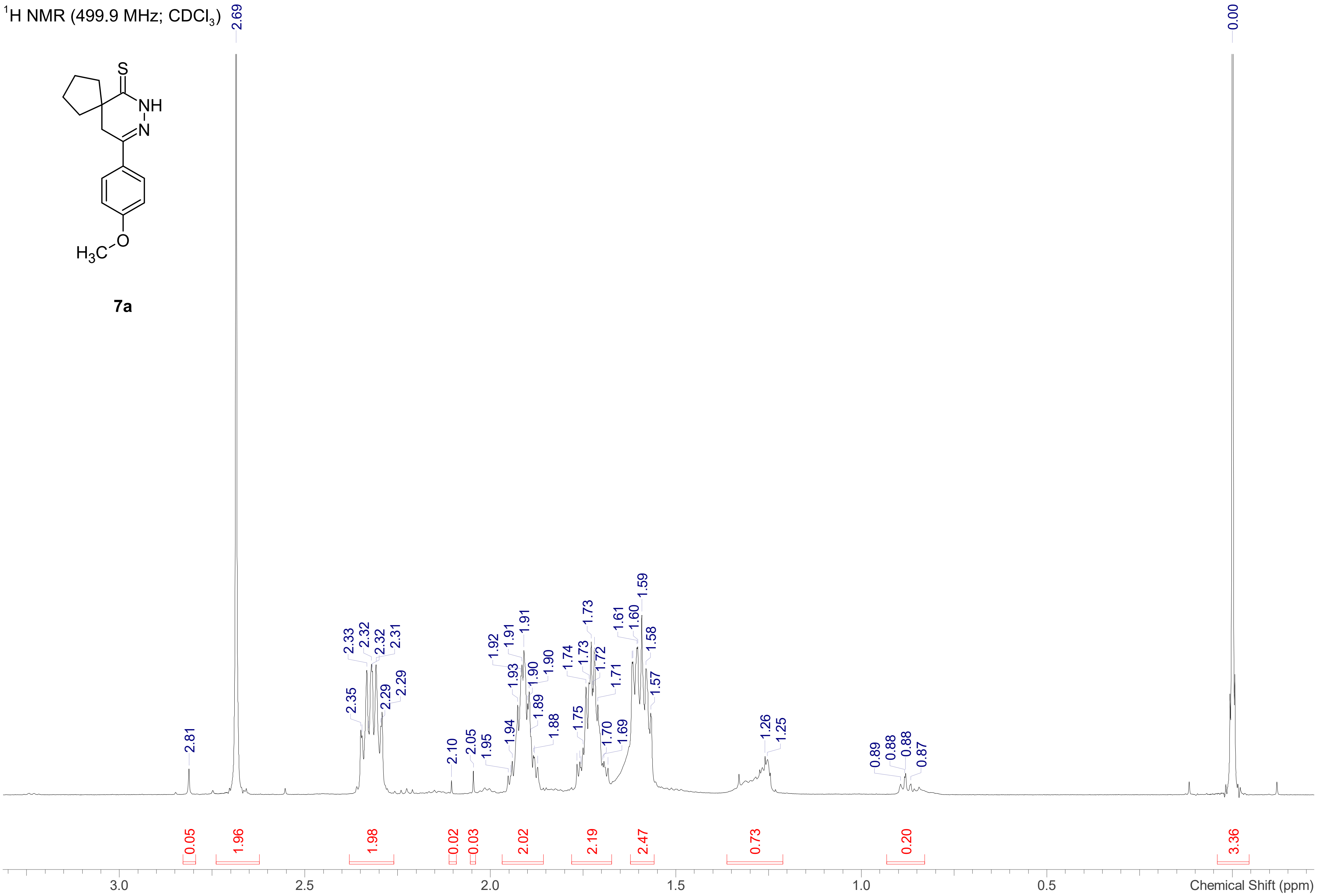

^{13}C NMR (125.7 MHz; $CDCl_3$)

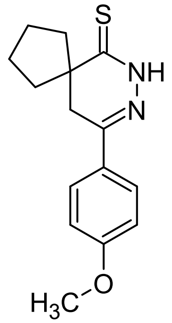

7a

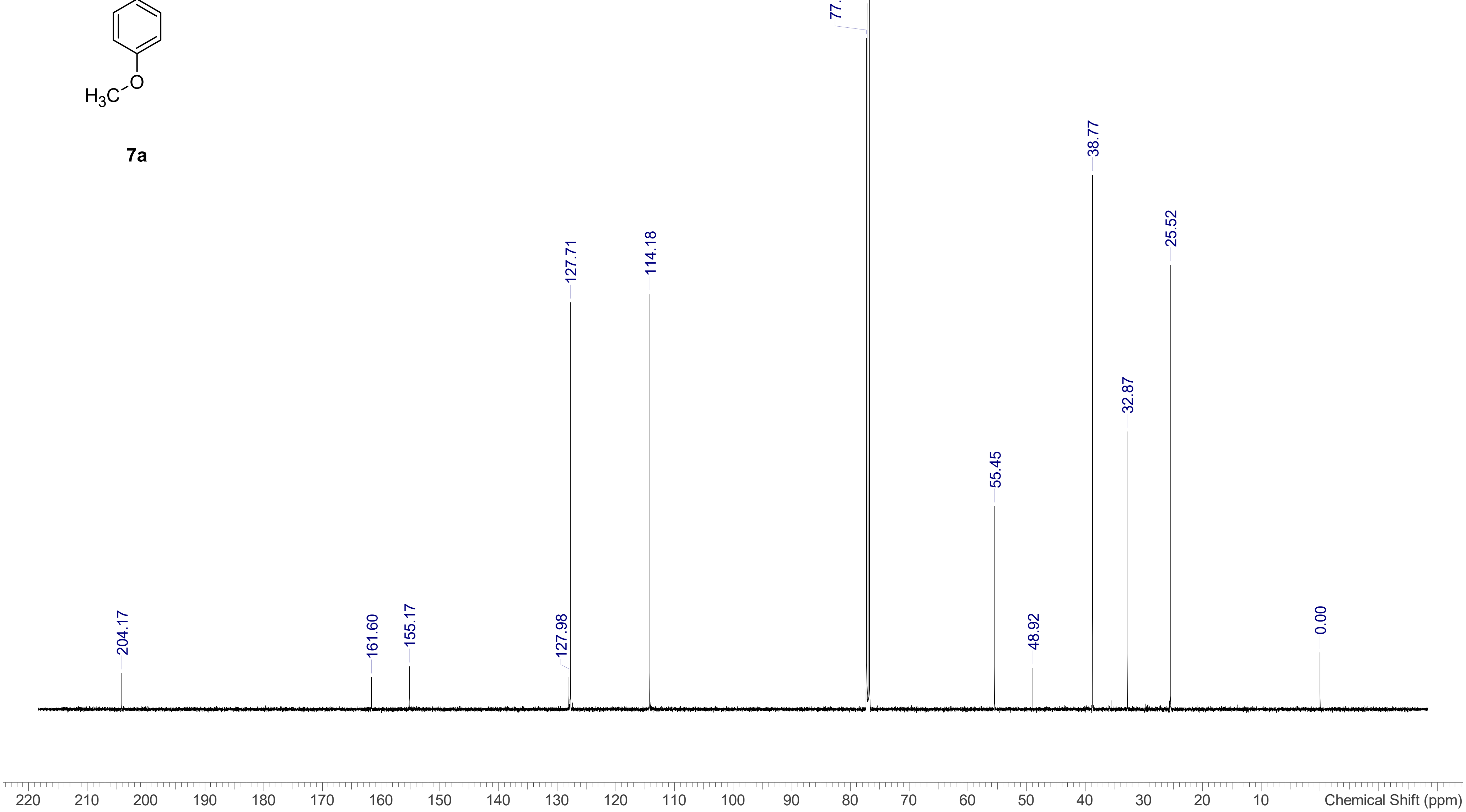

Compound **7a**

HRMS spectrum (ESI)

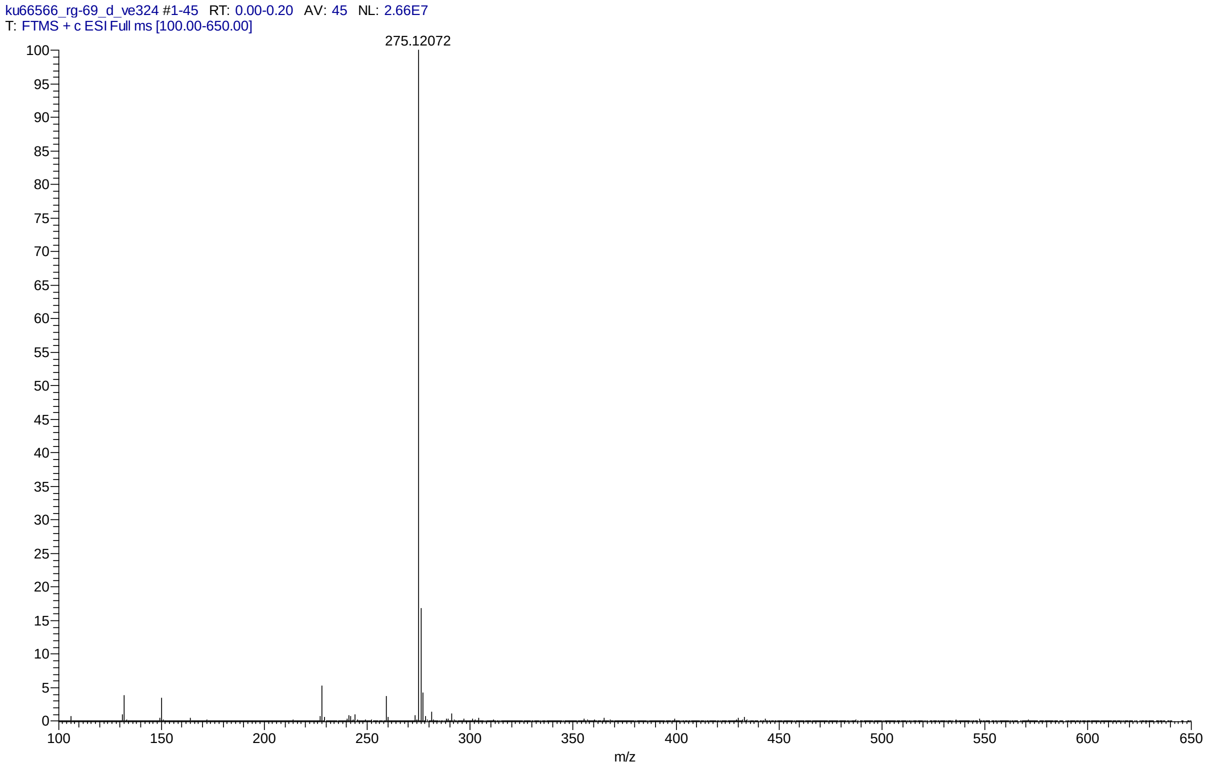

HRMS spectrum (ESI daughter ion)

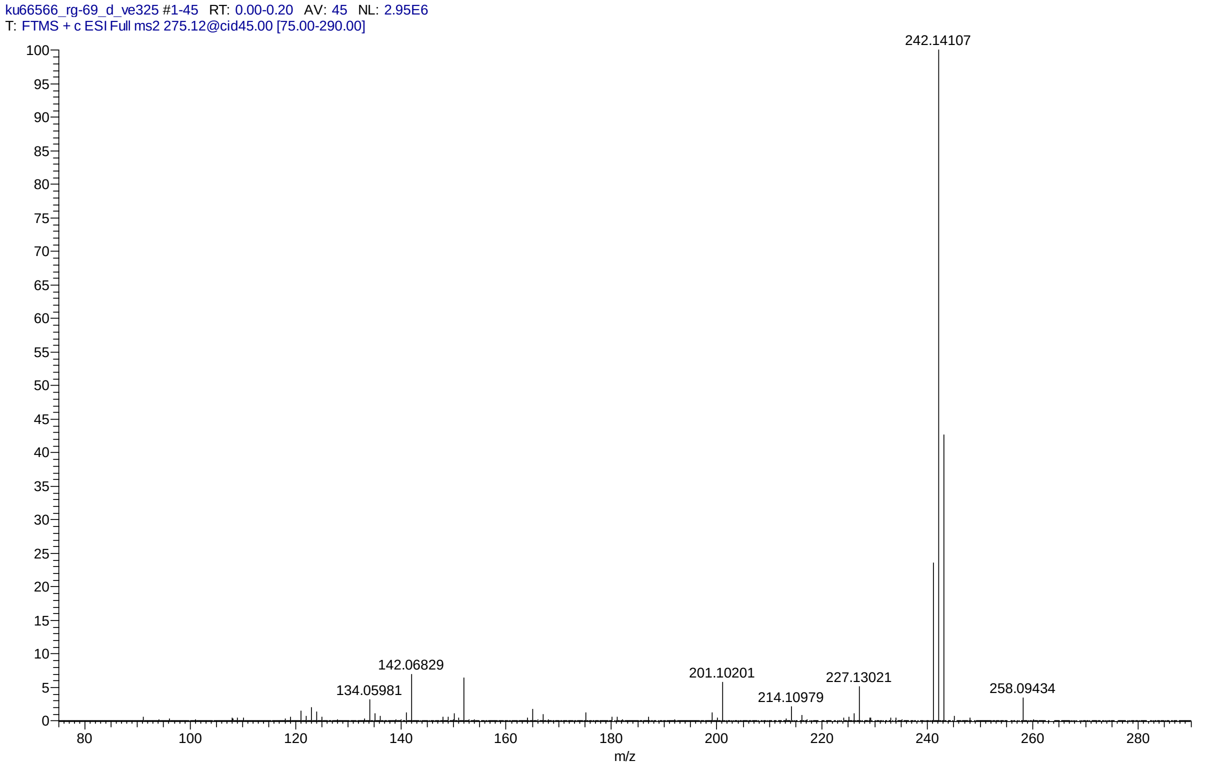

^{1}H NMR (499.9 MHz; $CDCl_3$)

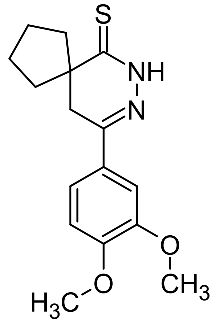

7b

9-(3,4-Dimethoxyphenyl)-7,8-diazaspiro[4.5]dec-8-ene-6-thione

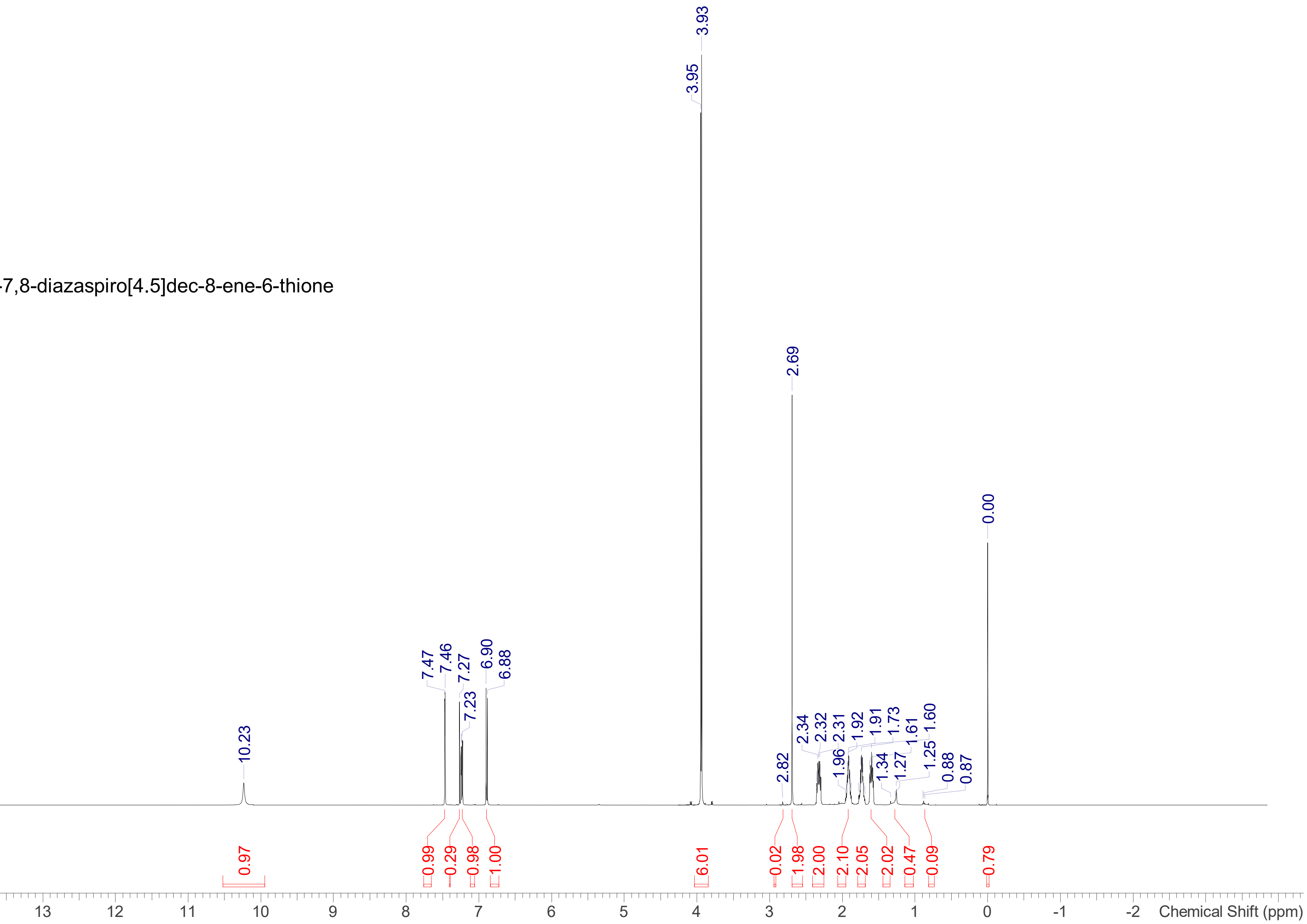

^{1}H NMR (499.9 MHz; $CDCl_3$)

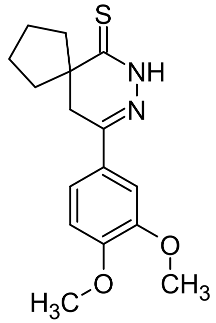

7b

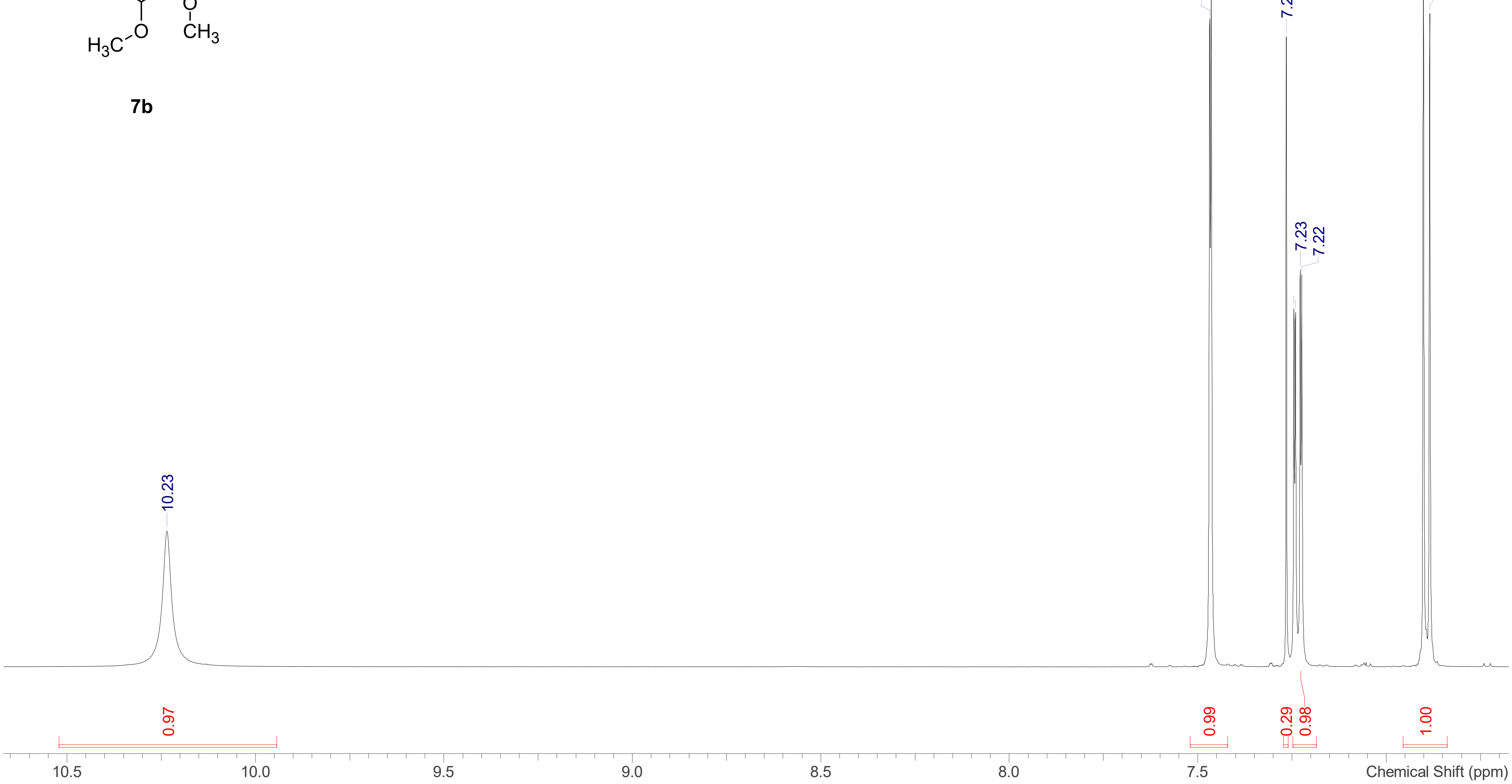

^{1}H NMR (499.9 MHz; $CDCl_3$)

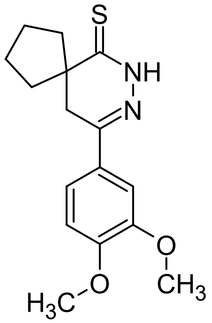

7b

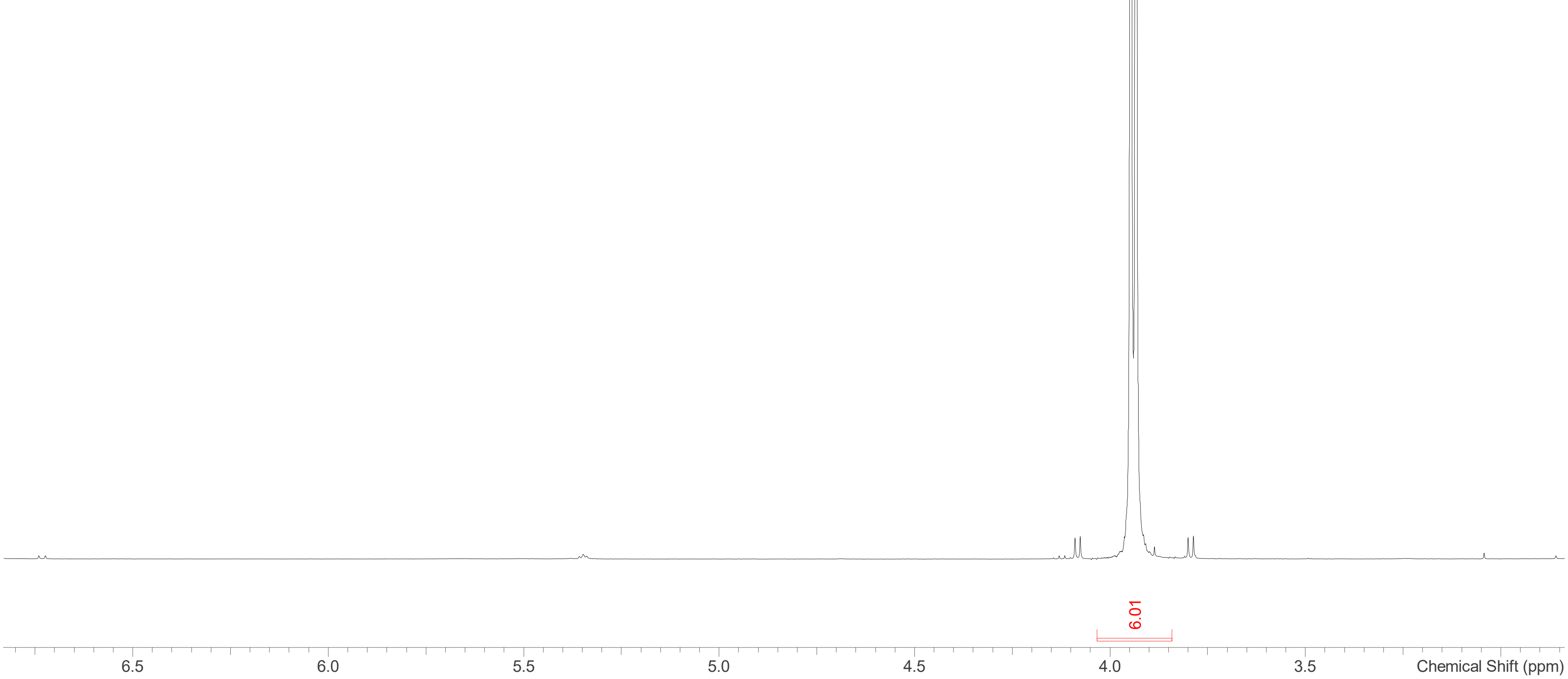

^{1}H NMR (499.9 MHz; $CDCl_3$)

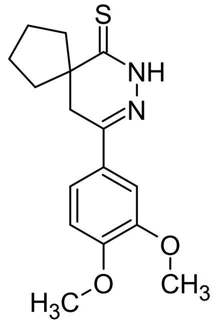

7b

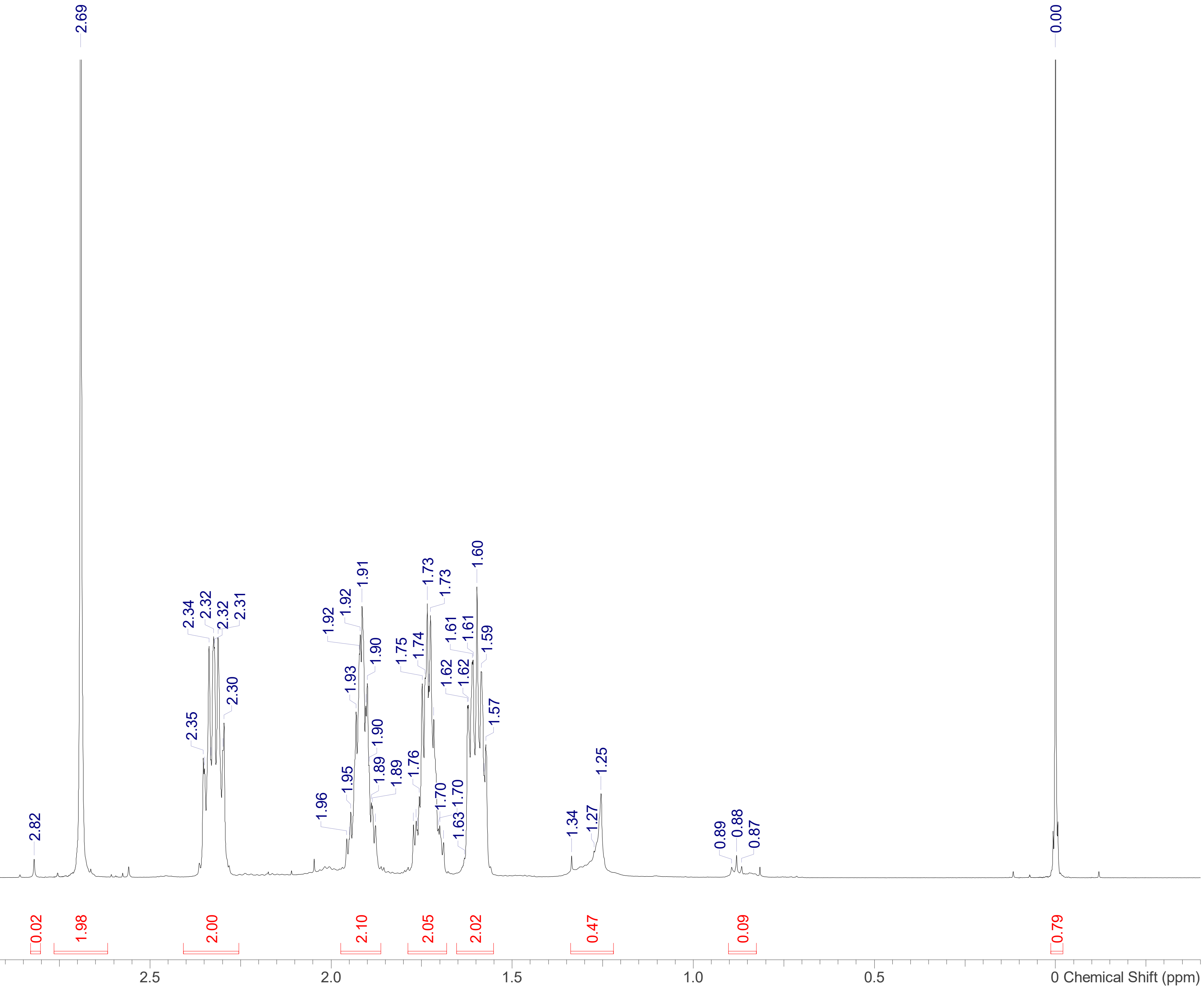

^{13}C NMR (125.7 MHz; $CDCl_3$)

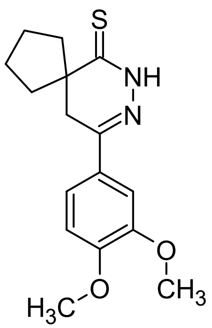

7b

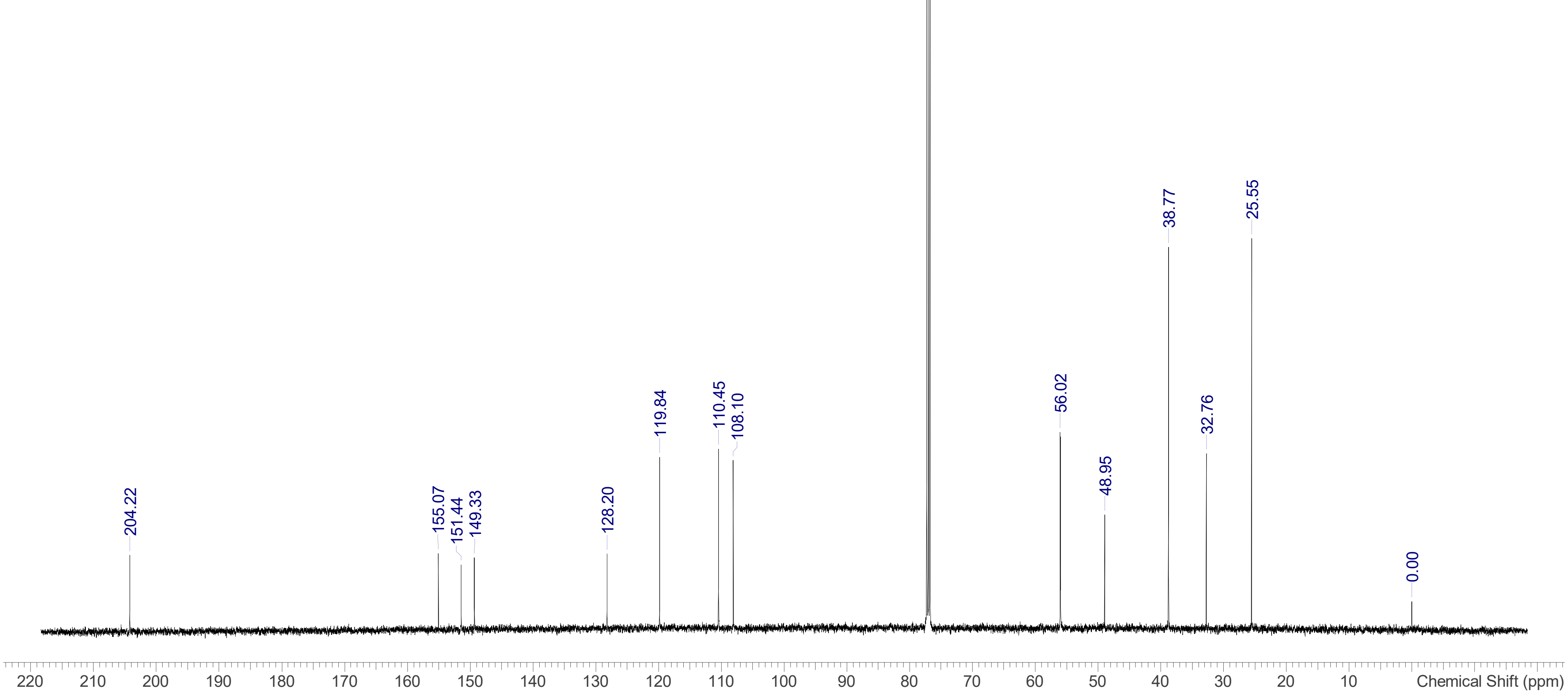

Compound **7b**

HRMS spectrum (ESI)

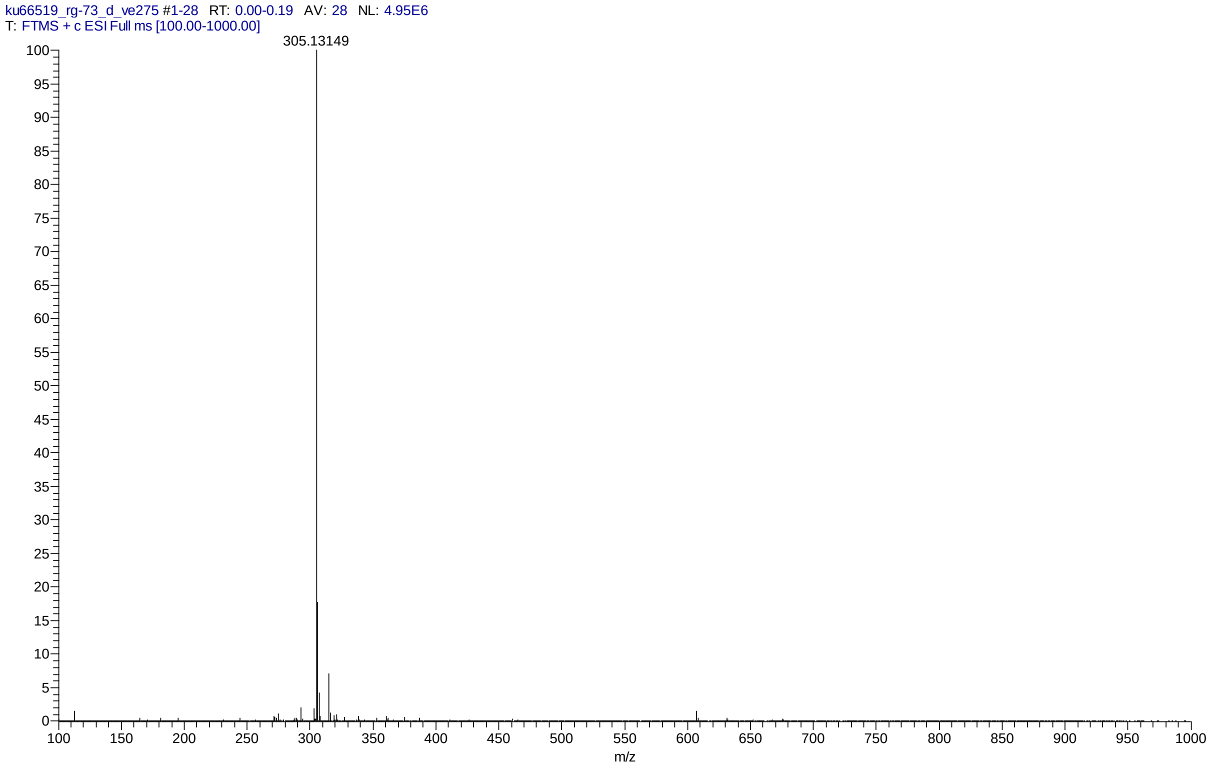

HRMS spectrum (ESI daughter ion)

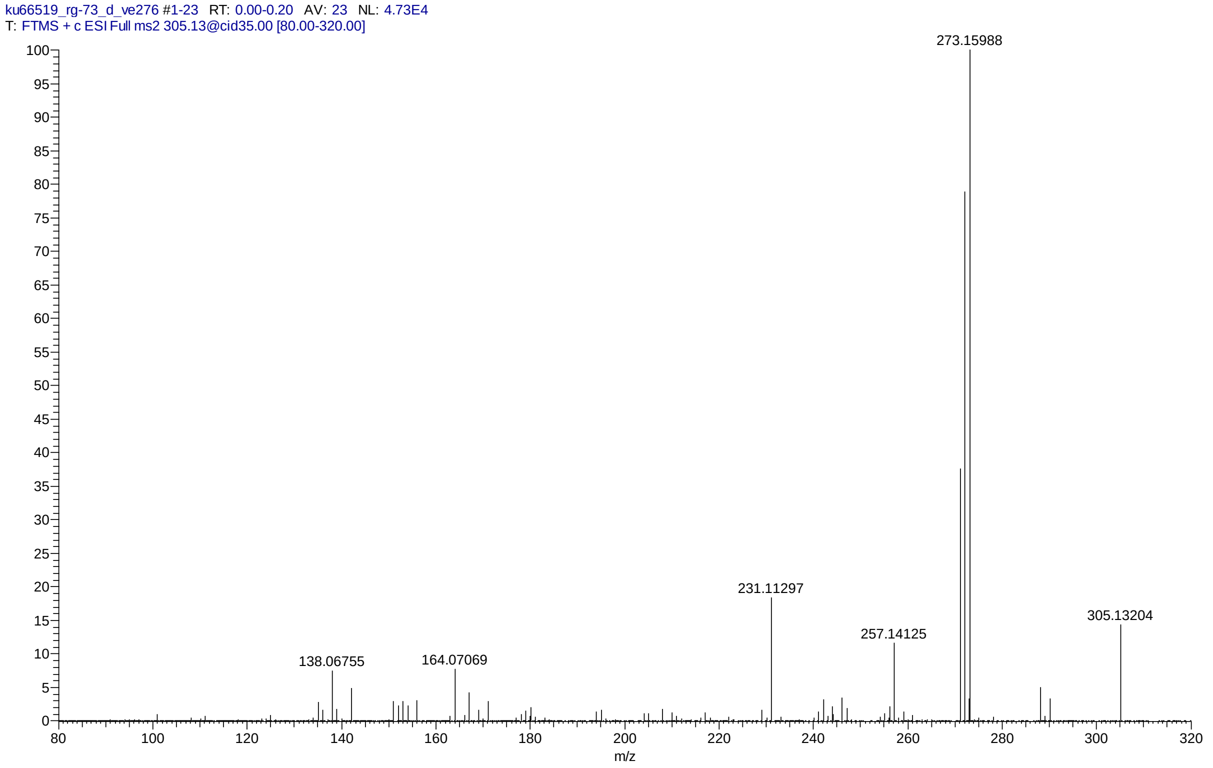

^{1}H NMR (499.9 MHz; DMSO-d_6)

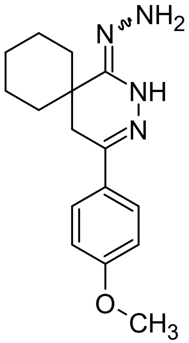

8a

1-Hydrazono-4-(4-methoxyphenyl)-2,3-diazaspiro[5.5]undec-3-ene

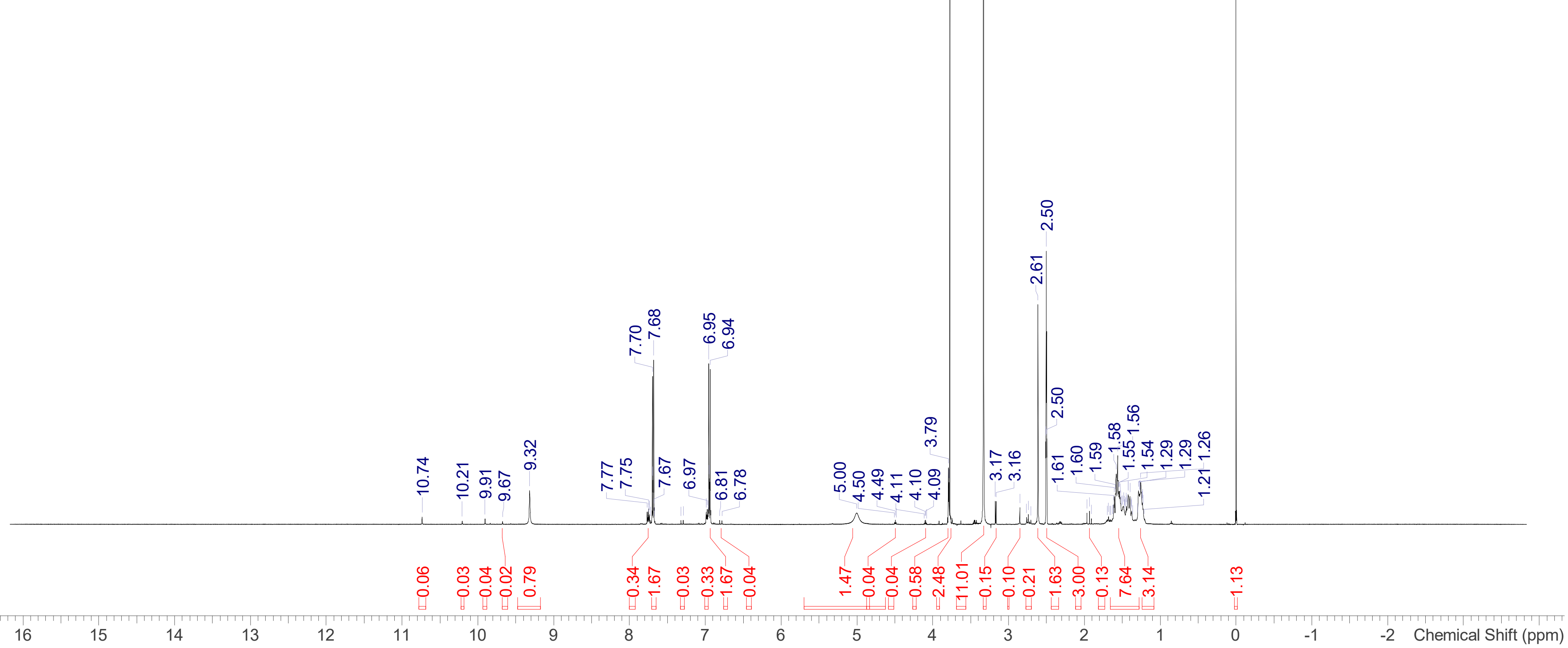

^{1}H NMR (499.9 MHz; DMSO-d_6)

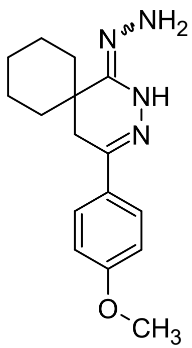

8a

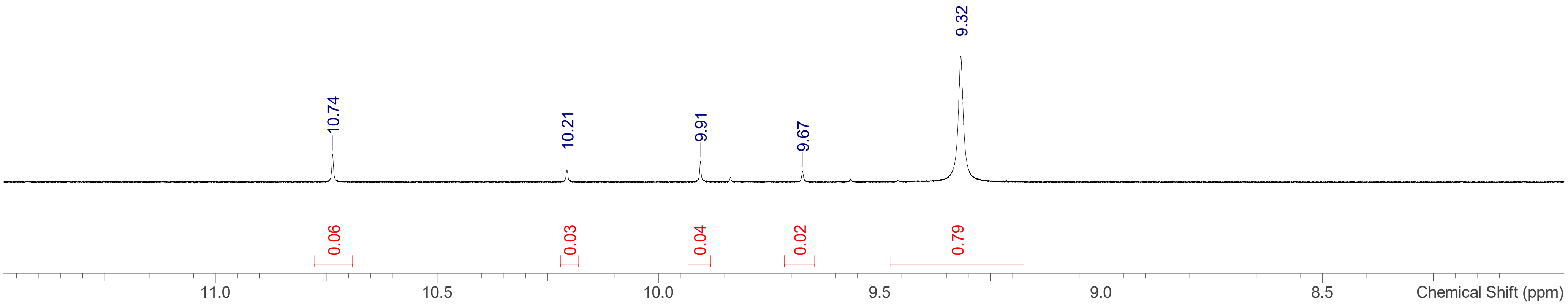

^{1}H NMR (499.9 MHz; DMSO-d_6)

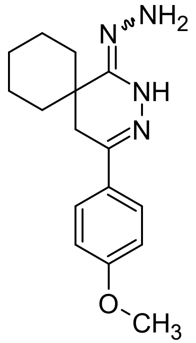

8a

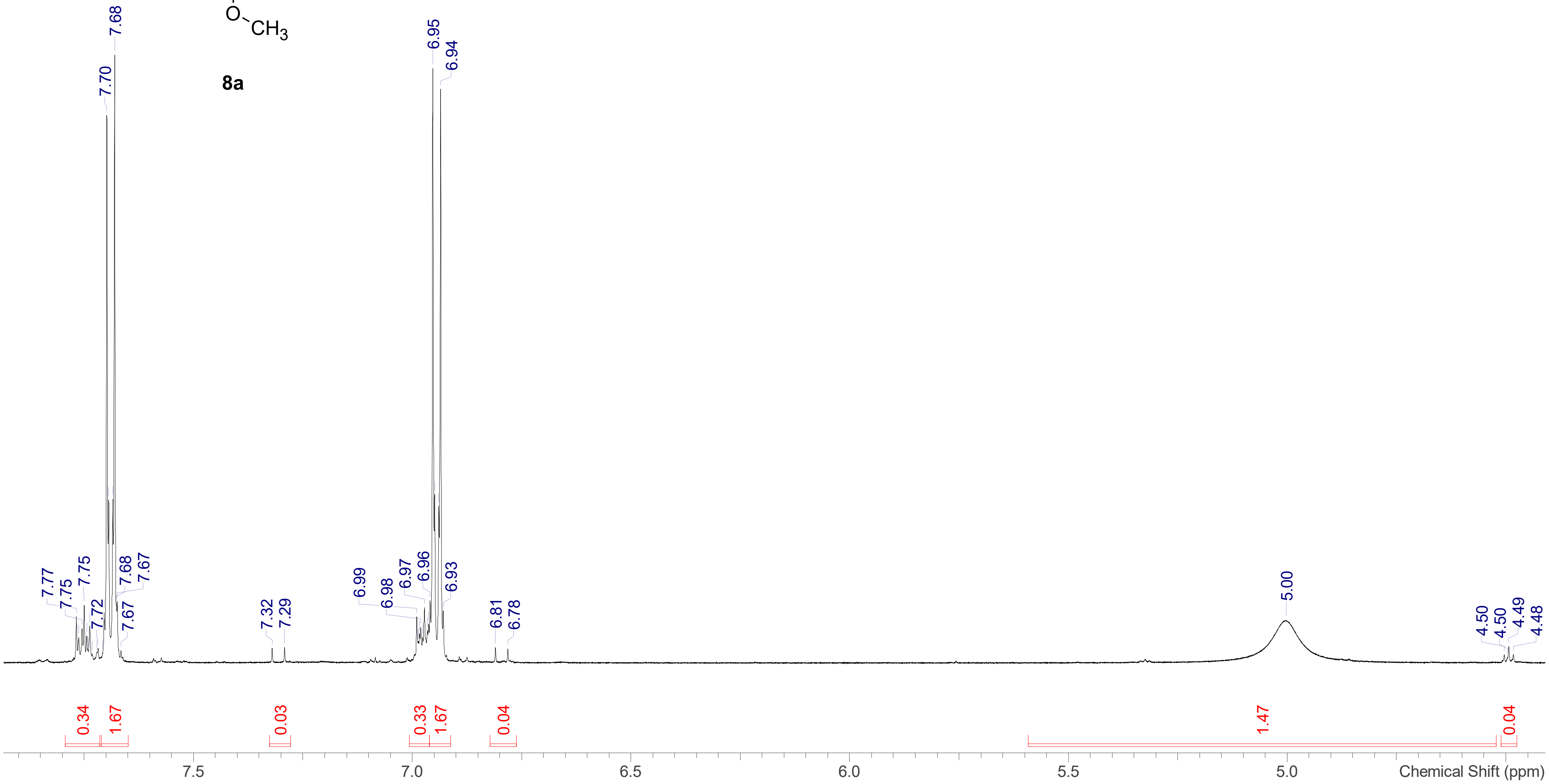

^1H NMR (499.9 MHz; DMSO-d_6)

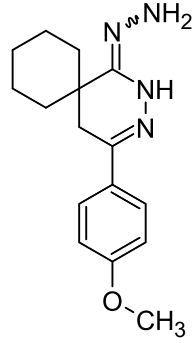

8a

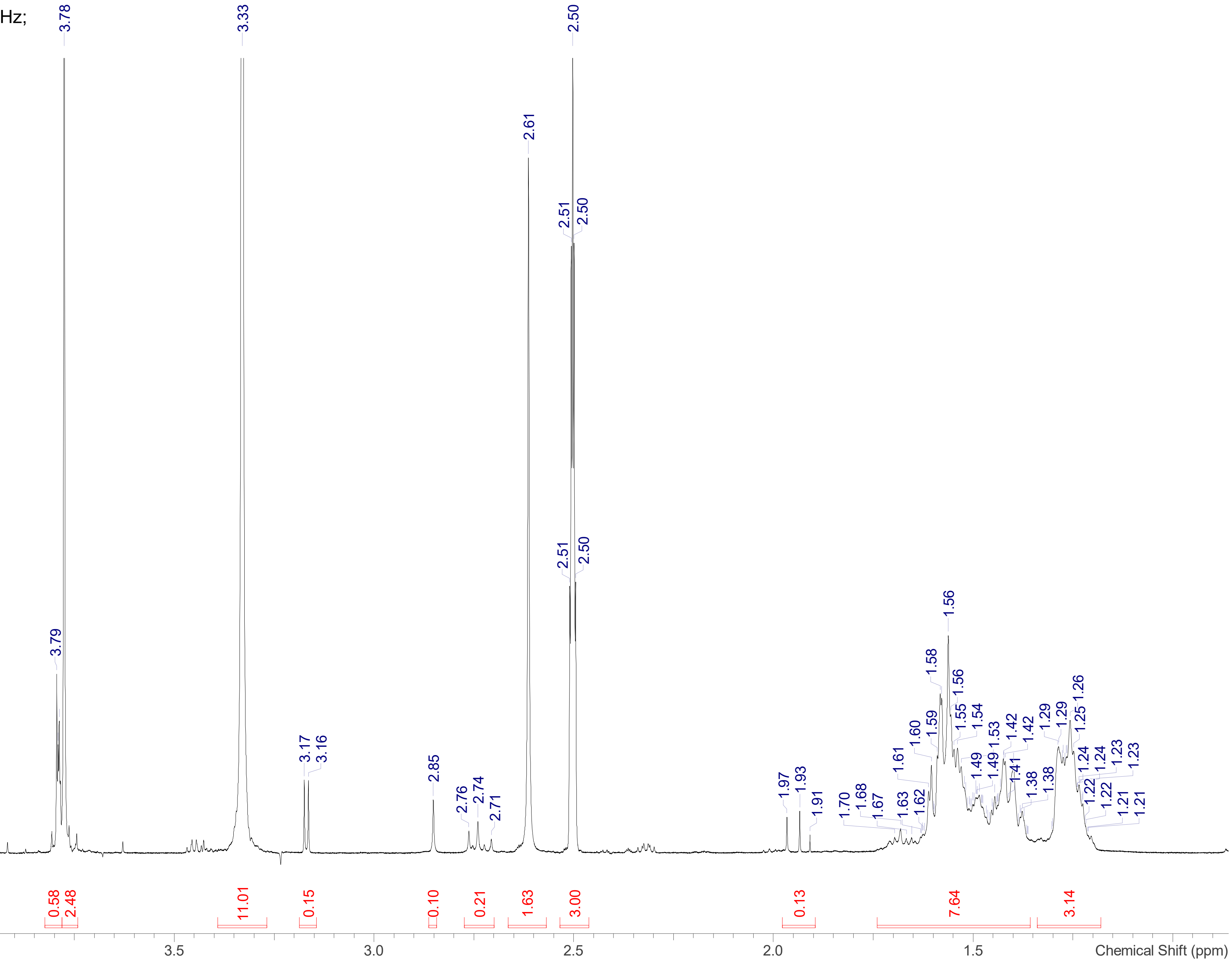

^{13}C NMR (125.7 MHz; DMSO-d_6)

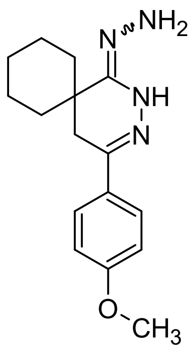

8a

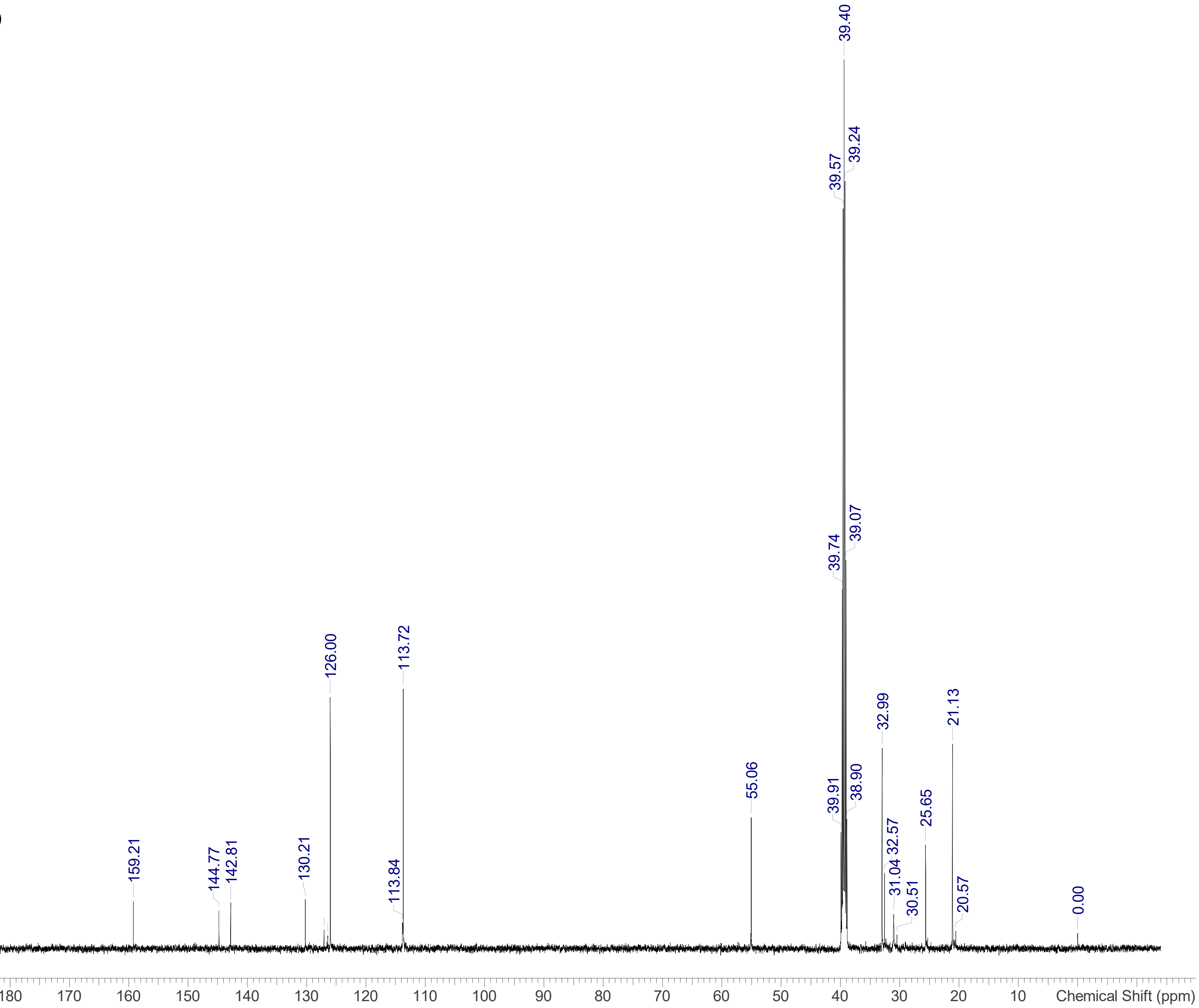

^{13}C NMR (125.7 MHz; DMSO-d_6)

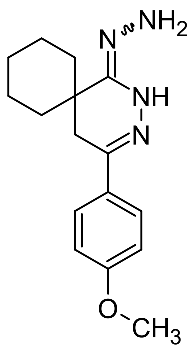

8a

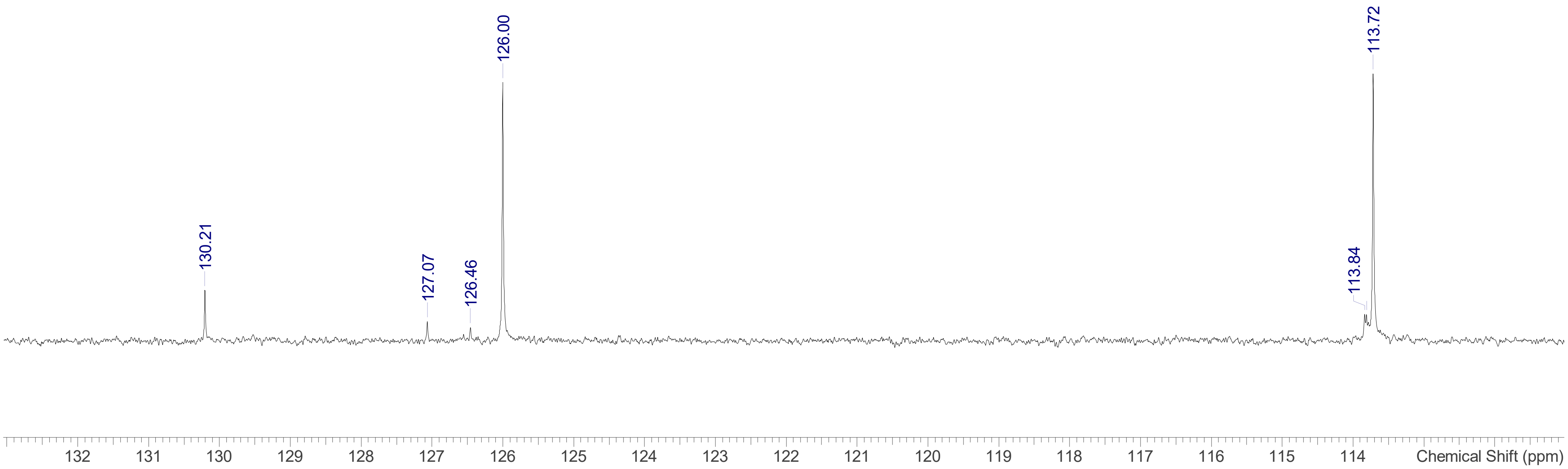

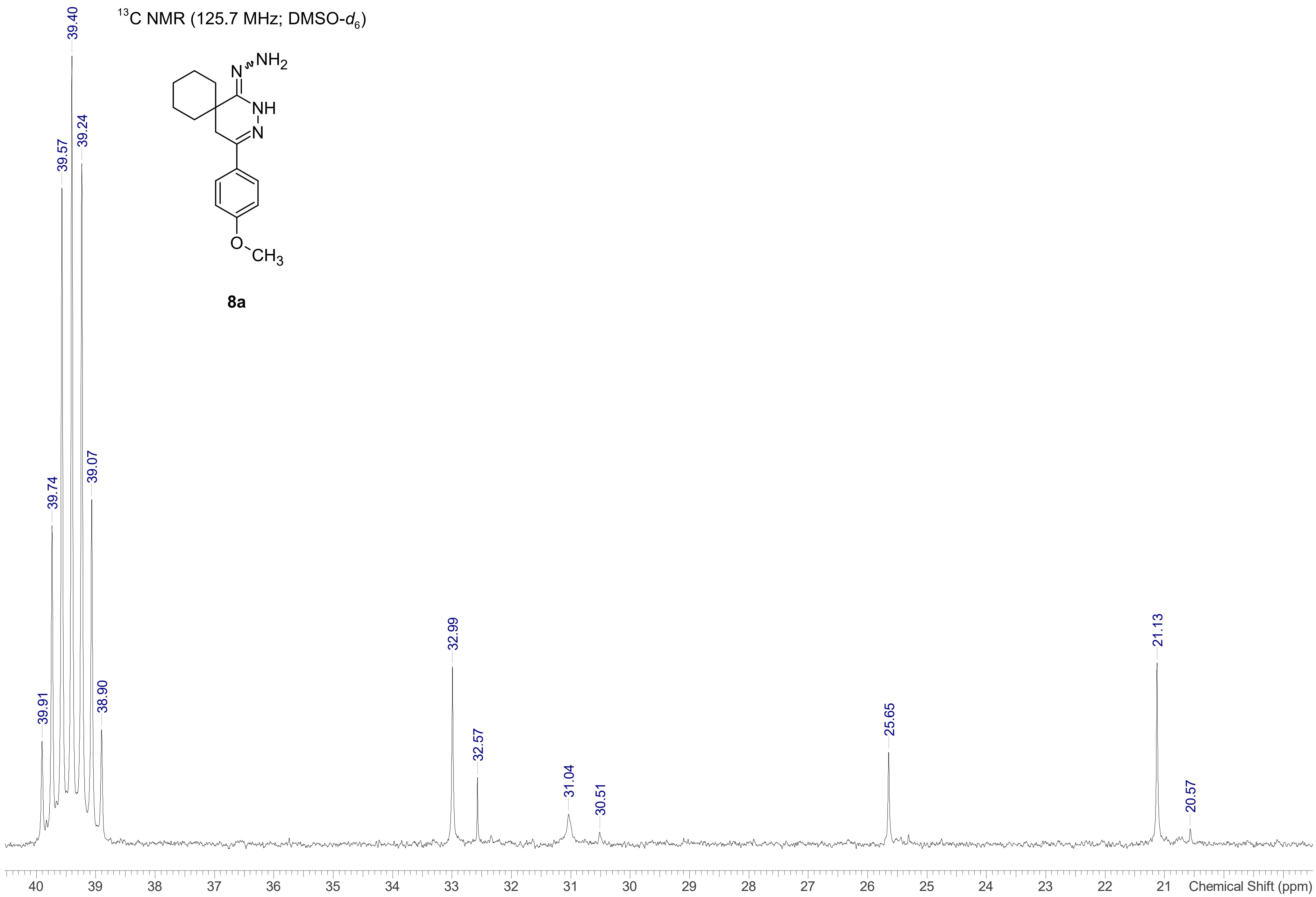

^{13}C NMR (125.7 MHz; DMSO-d_6)
8a
39.91
39.74
39.57
39.40
39.24
39.07
38.90
32.99
32.57
31.04
30.51
25.65
21.13
20.57
40
39
38
37
36
35
34
33
32
31
30
29
28
27
26
25
24
23
22
21
Chemical Shift (ppm)

Compound 8a

HRMS spectrum (ESI)

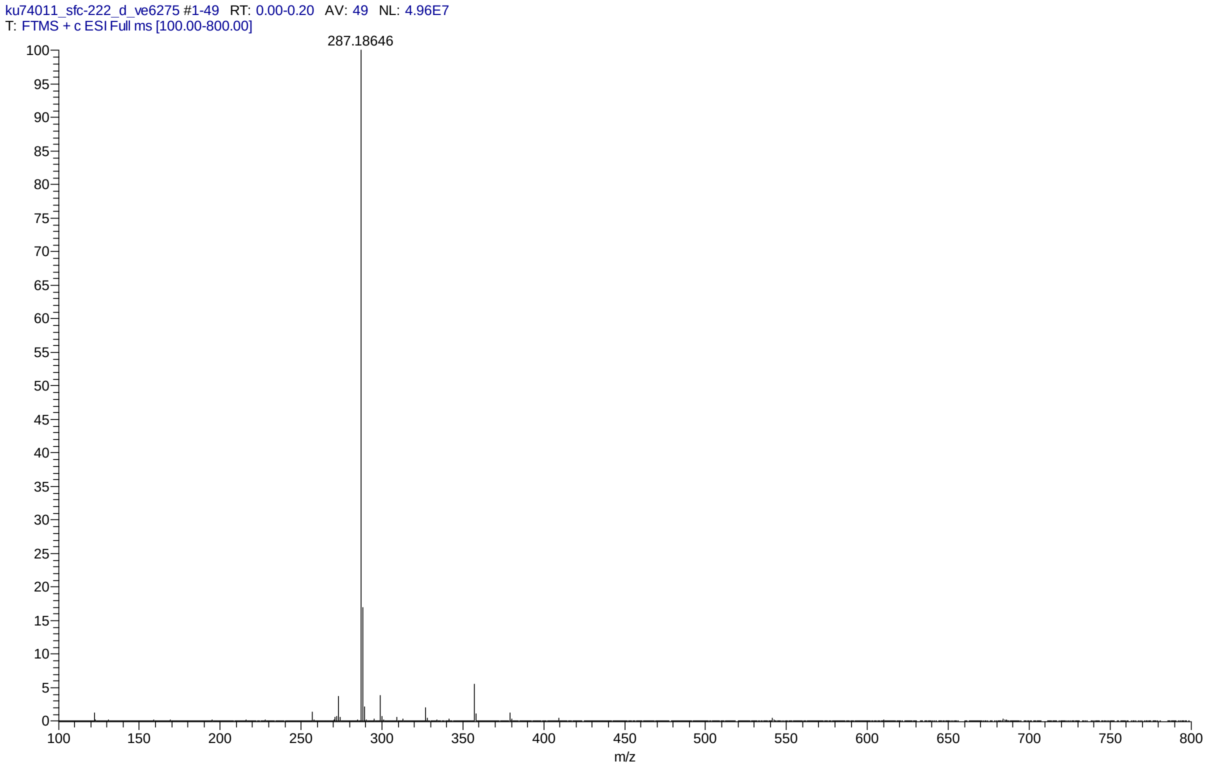

HRMS spectrum (ESI daughter ion)

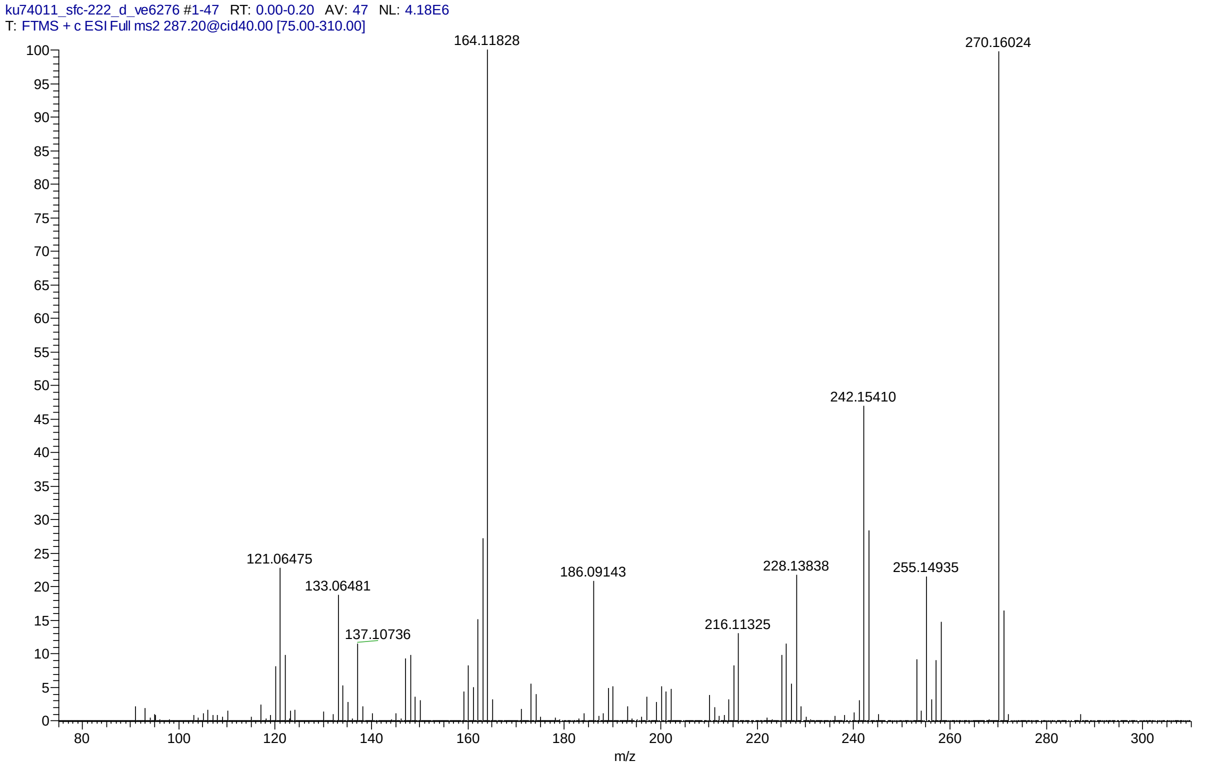

^{1}H NMR (499.9 MHz; DMSO-d_6)

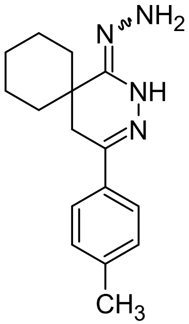

8b

1-Hydrazono-4-(*p*-tolyl)-2,3-diazaspiro[5.5]undec-3-ene

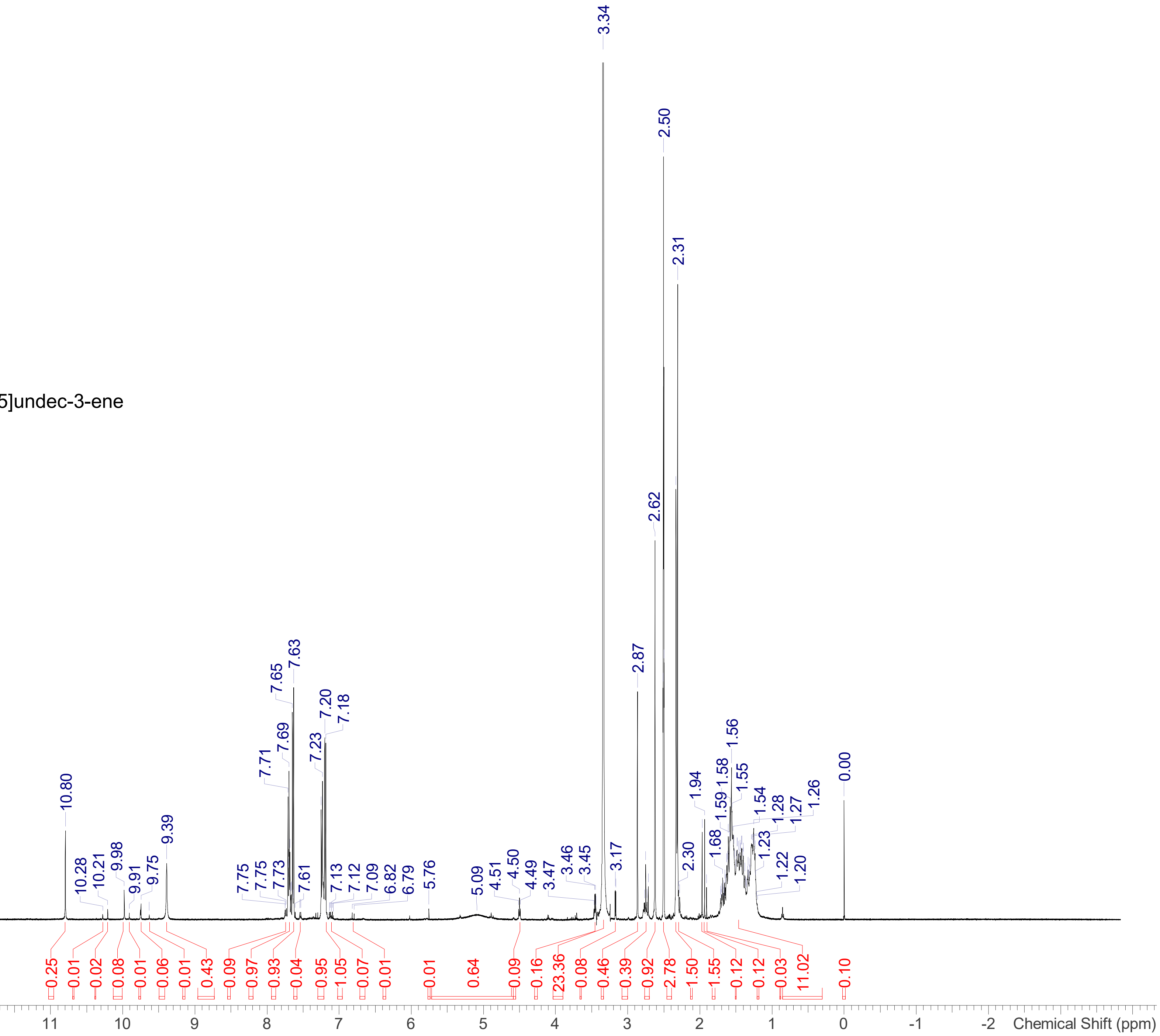

^{1}H NMR (499.9 MHz; DMSO-d_6)

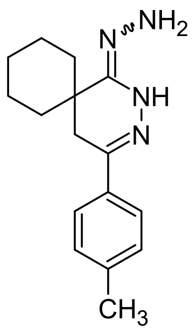

8b

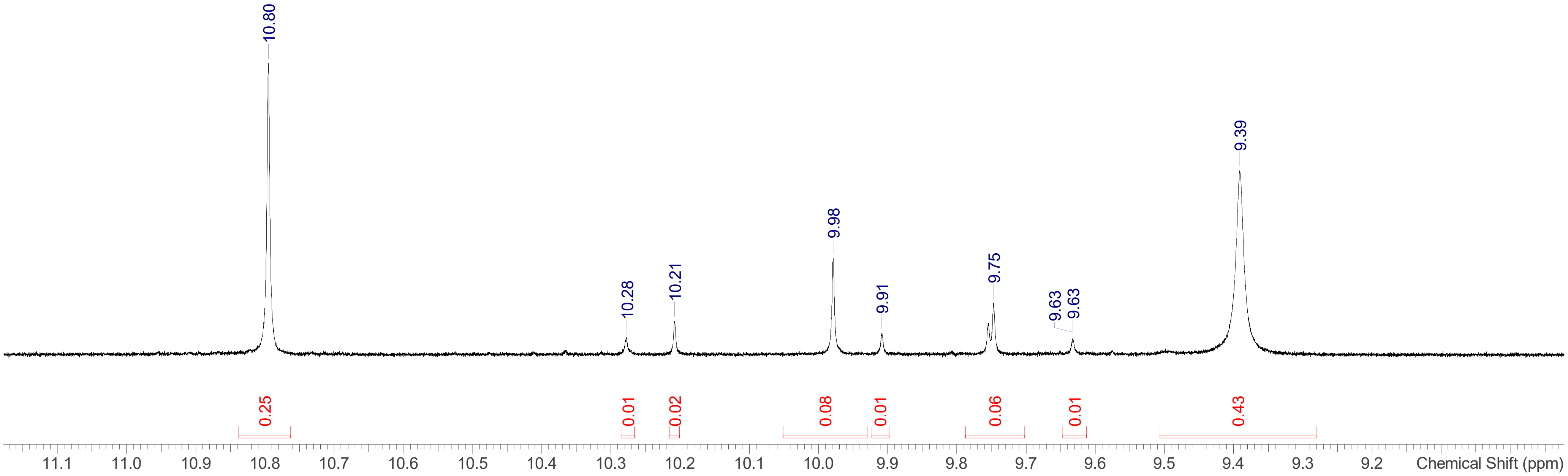

^{1}H NMR (499.9 MHz; DMSO-d_6)

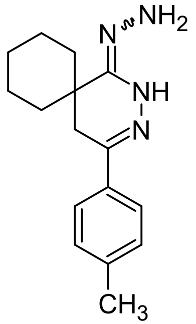

8b

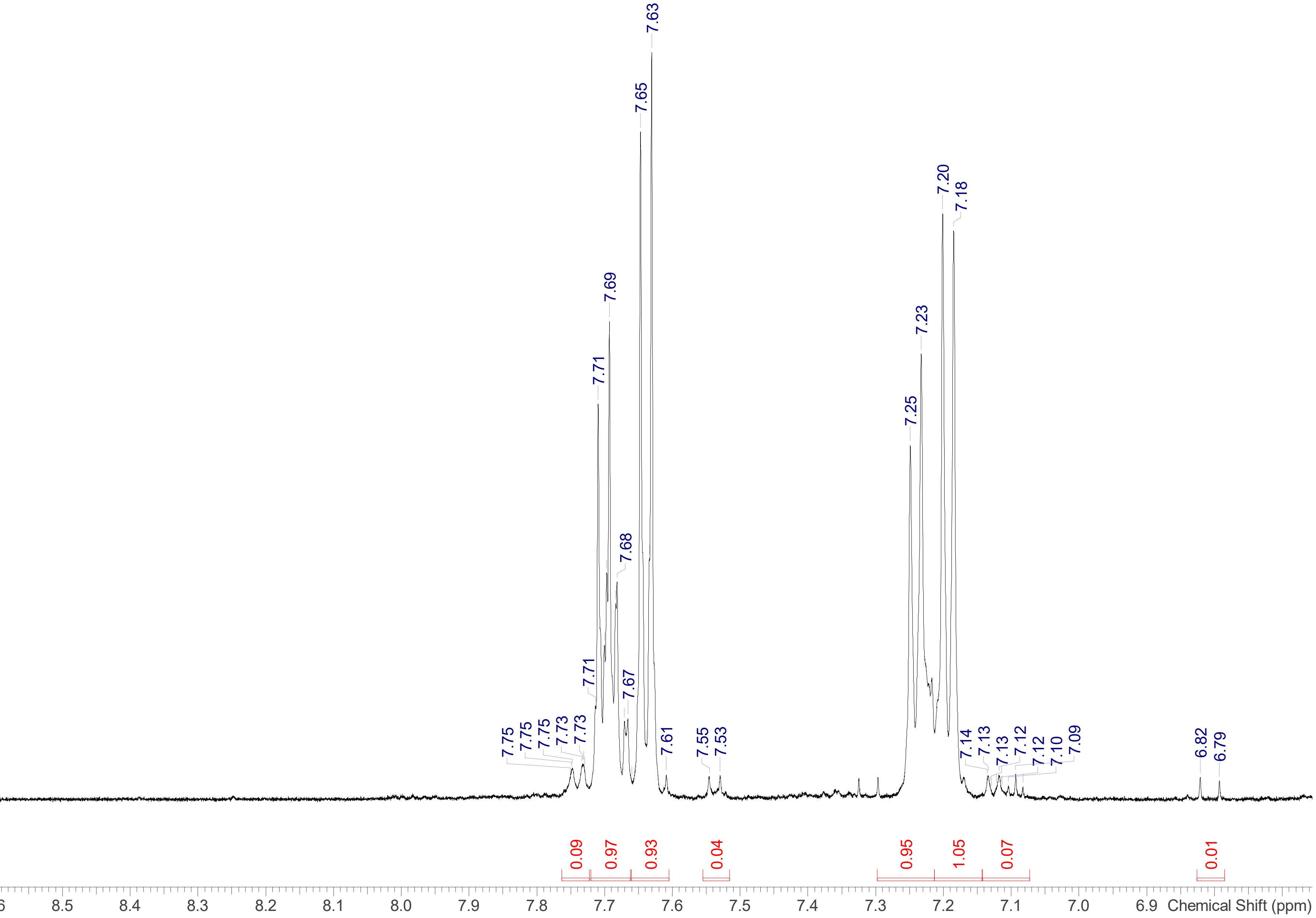

^1H NMR (499.9 MHz; DMSO-d_6)

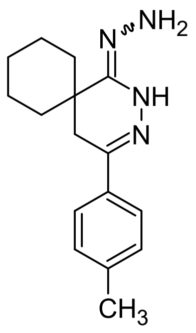

8b

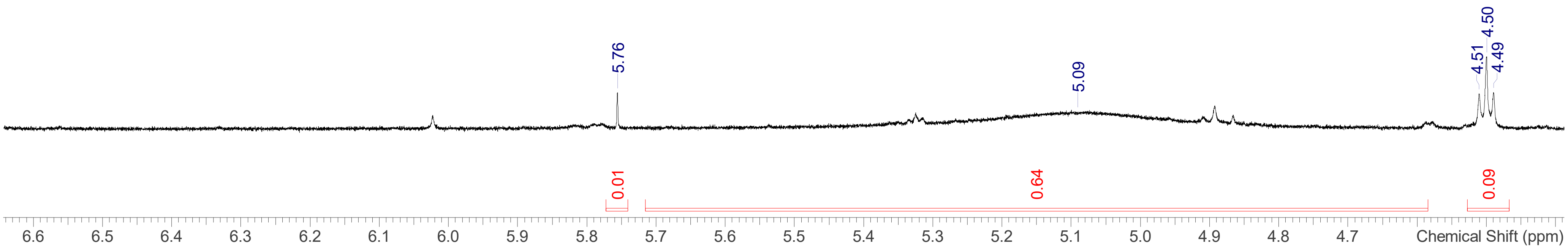

^{1}H NMR (499.9 MHz; DMSO-d_6)

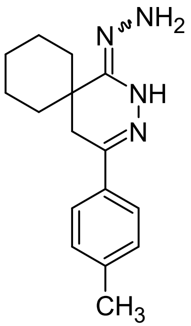

8b

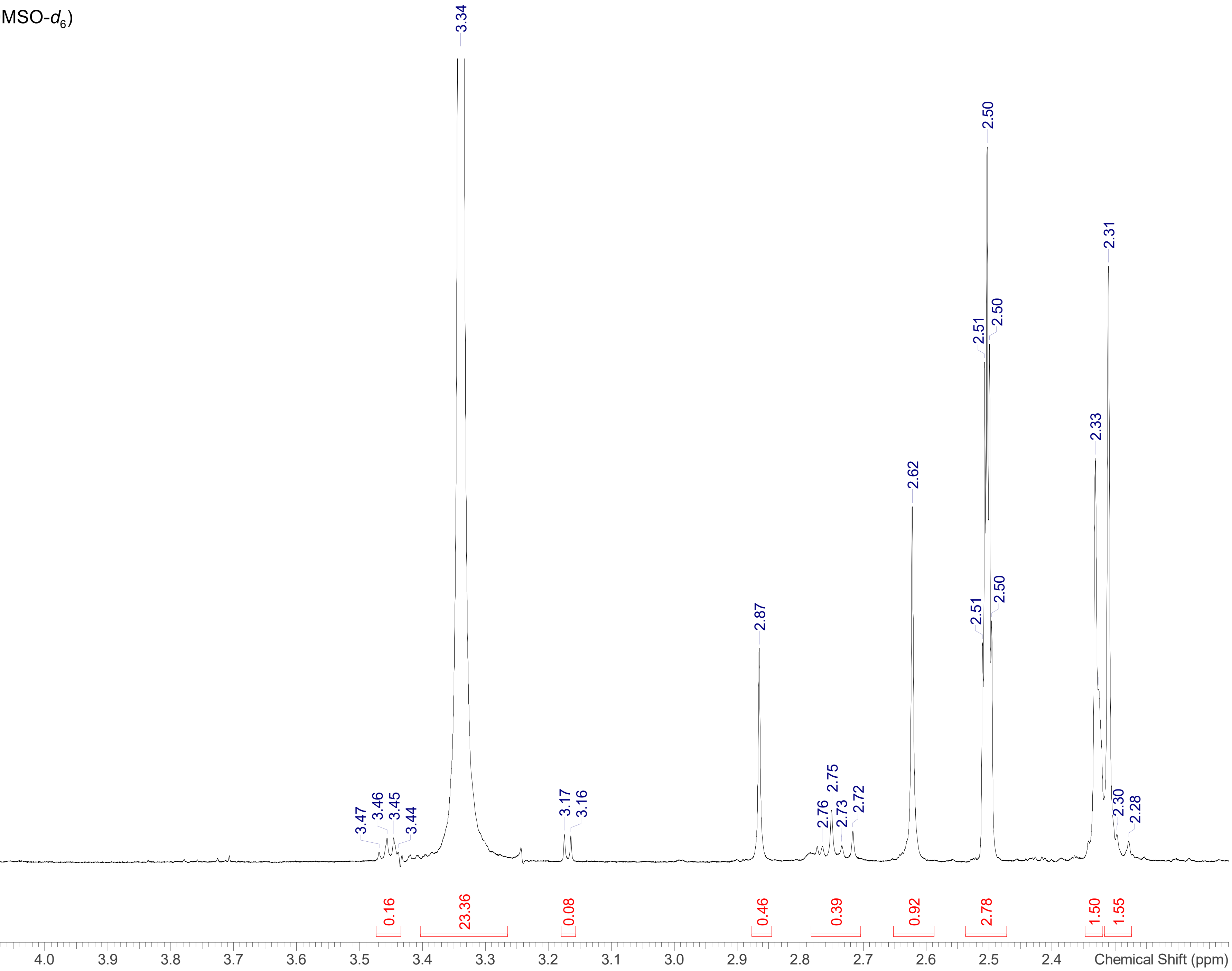

^{1}H NMR (499.9 MHz; DMSO-d_6)

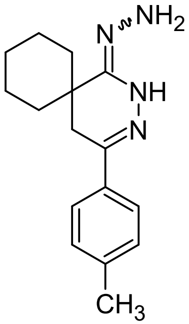

8b

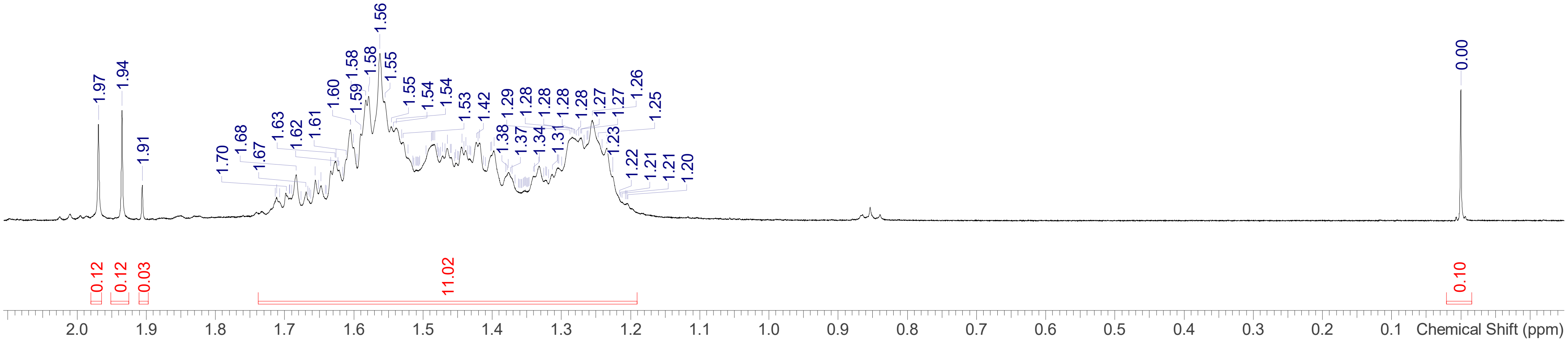

^{13}C NMR (125.7 MHz; DMSO-d_6)

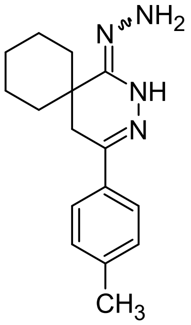

8b

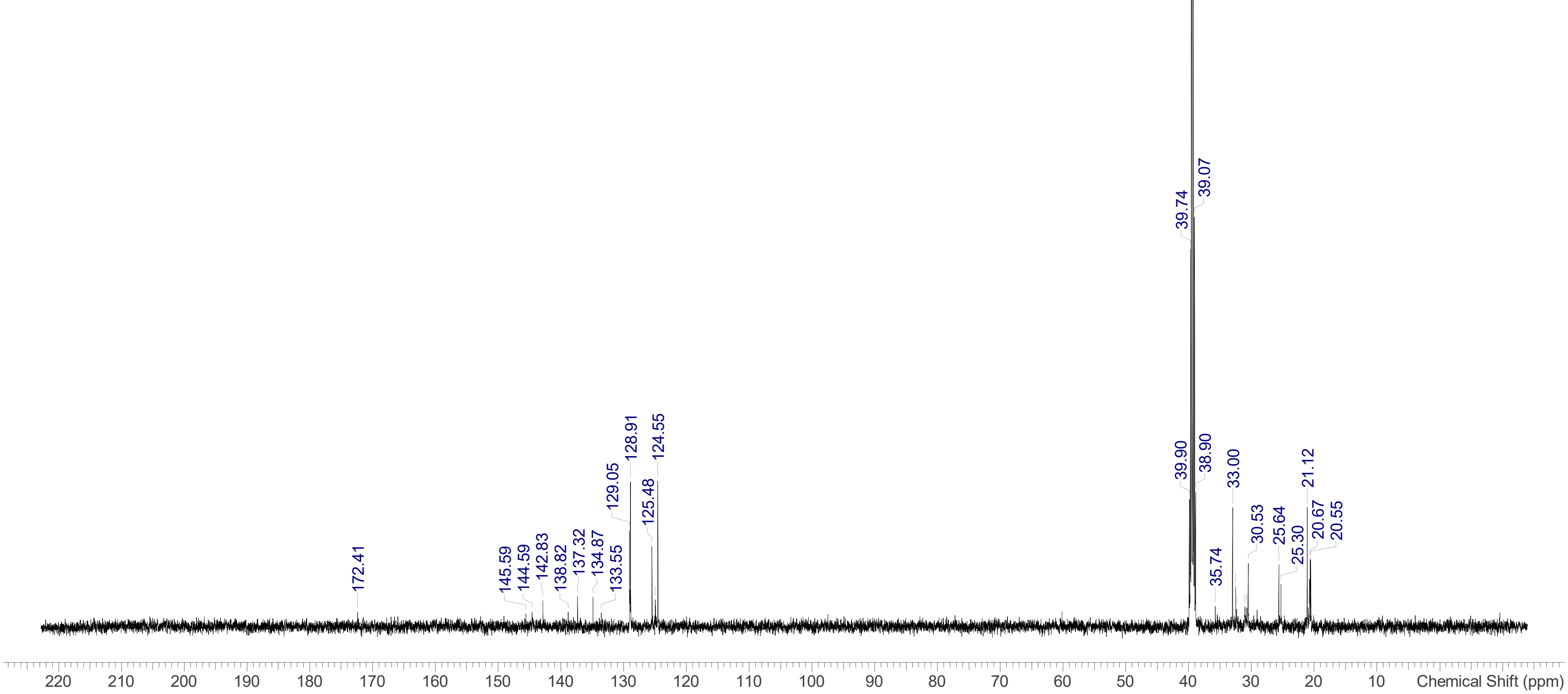

^{13}C NMR (125.7 MHz; DMSO-d_6)

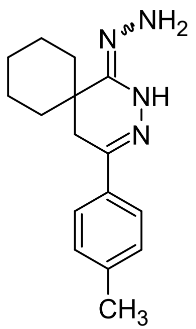

8b

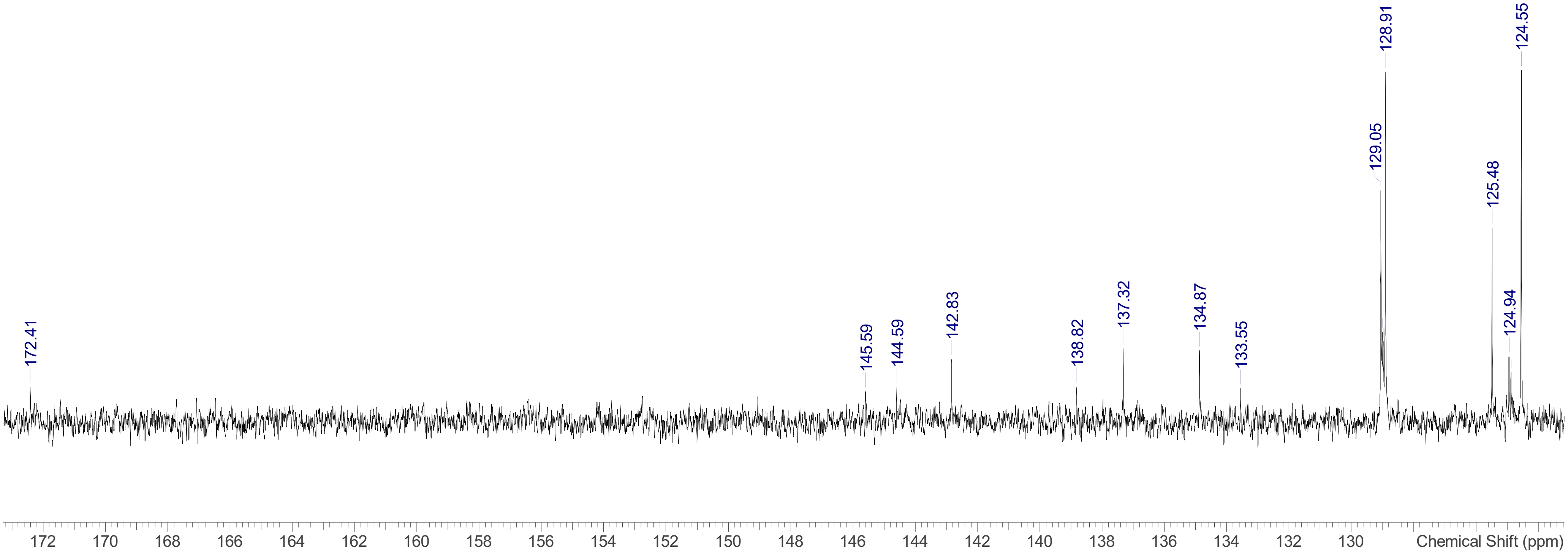

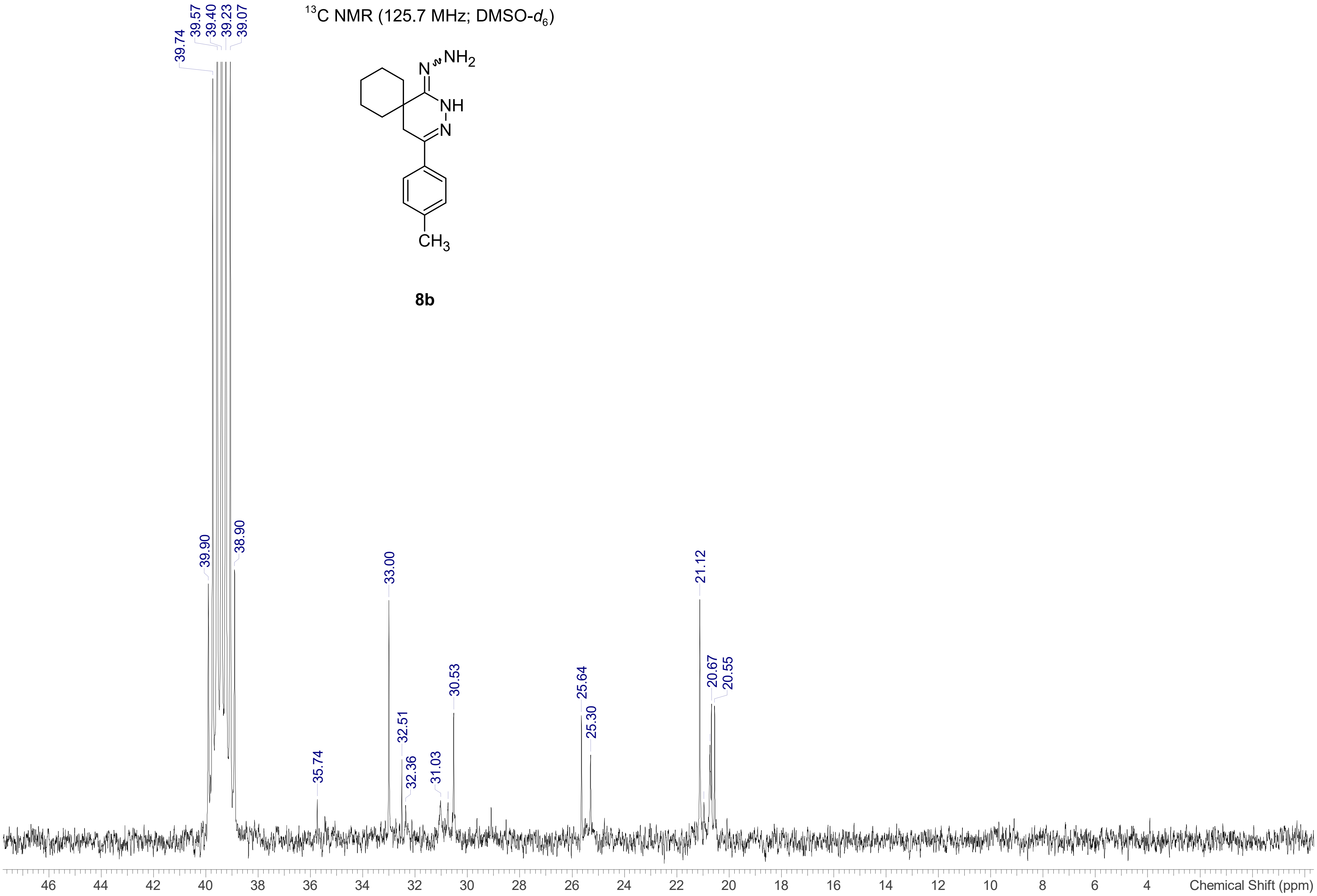

^{13}C NMR (125.7 MHz; DMSO-d_6)
8b
N
NH_2
NH
N
CH_3
39.74
39.57
39.40
39.23
39.07
39.90
38.90
35.74
33.00
32.51
32.36
31.03
30.53
25.64
25.30
21.12
20.67
20.55
46
44
42
40
38
36
34
32
30
28
26
24
22
20
18
16
14
12
10
8
6
4
Chemical Shift (ppm)

Compound **8b**

HRMS spectrum (ESI)

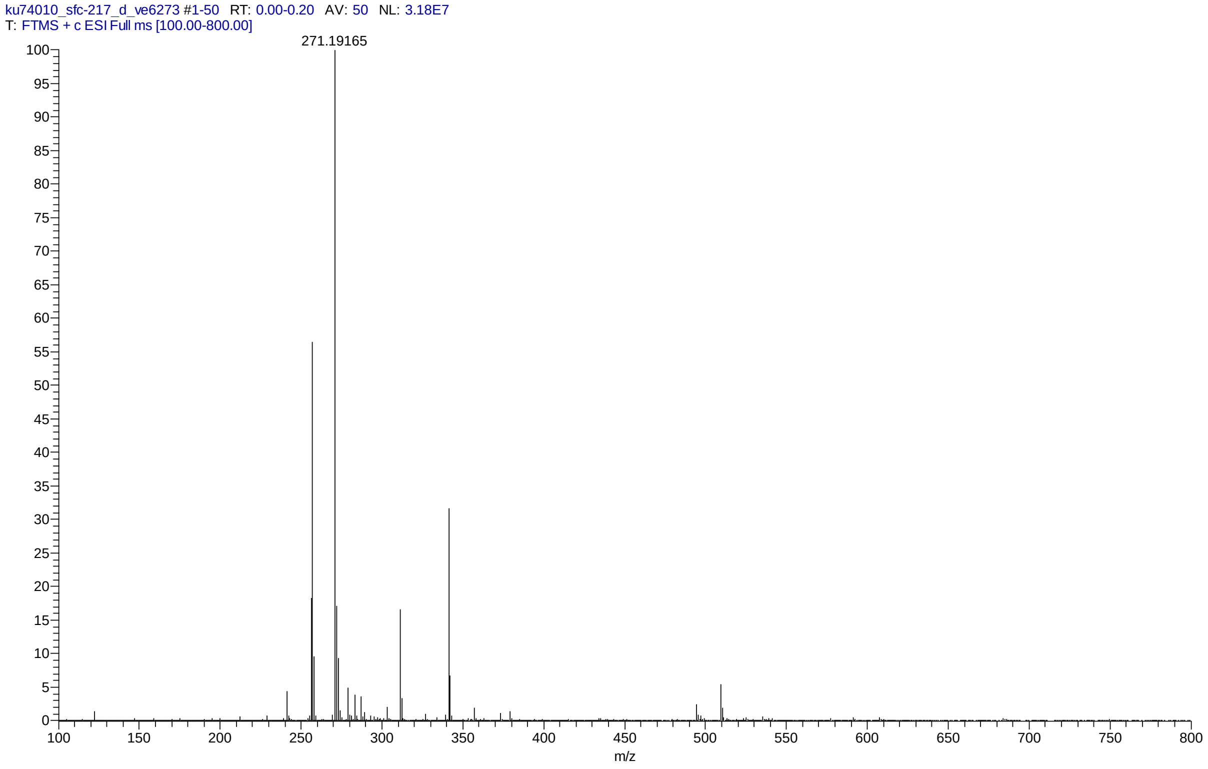

HRMS spectrum (ESI daughter ion)

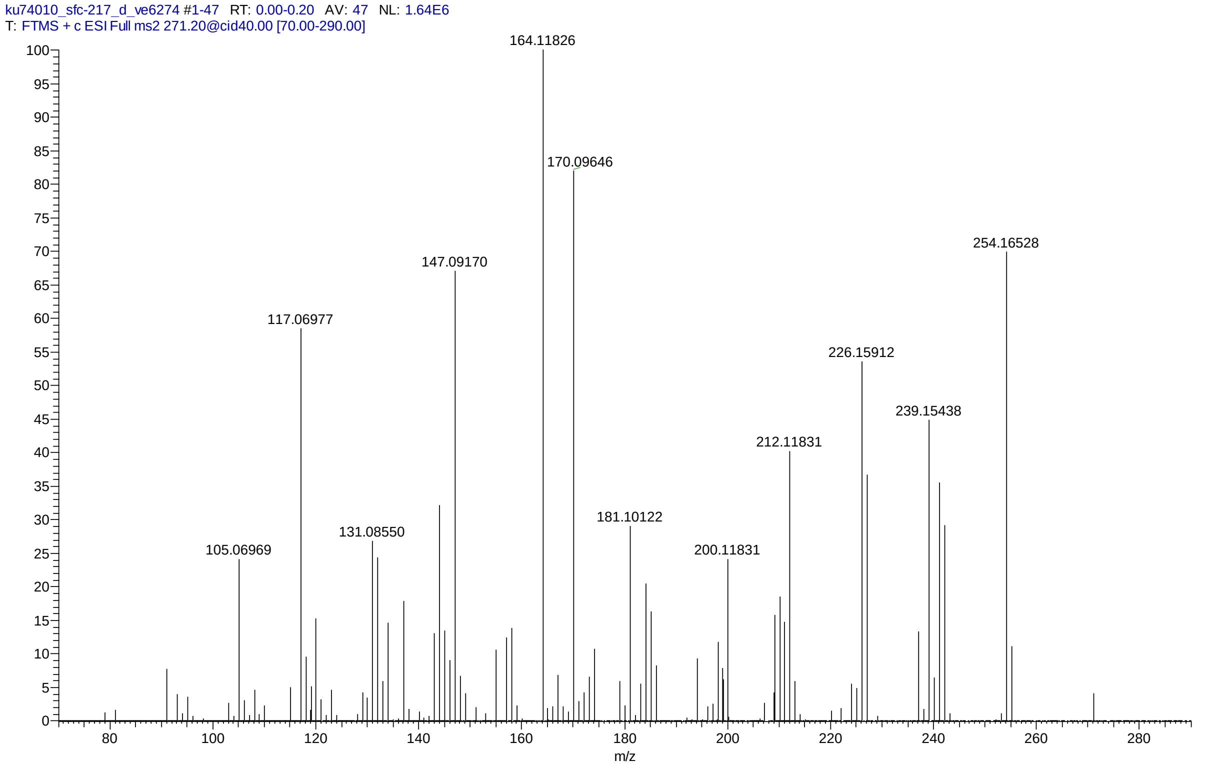

^{1}H NMR (499.9 MHz; DMSO-d_6)

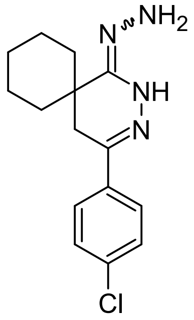

8c

4-(4-Chlorophenyl)-1-hydrazono-2,3-diazaspiro[5.5]undec-3-ene

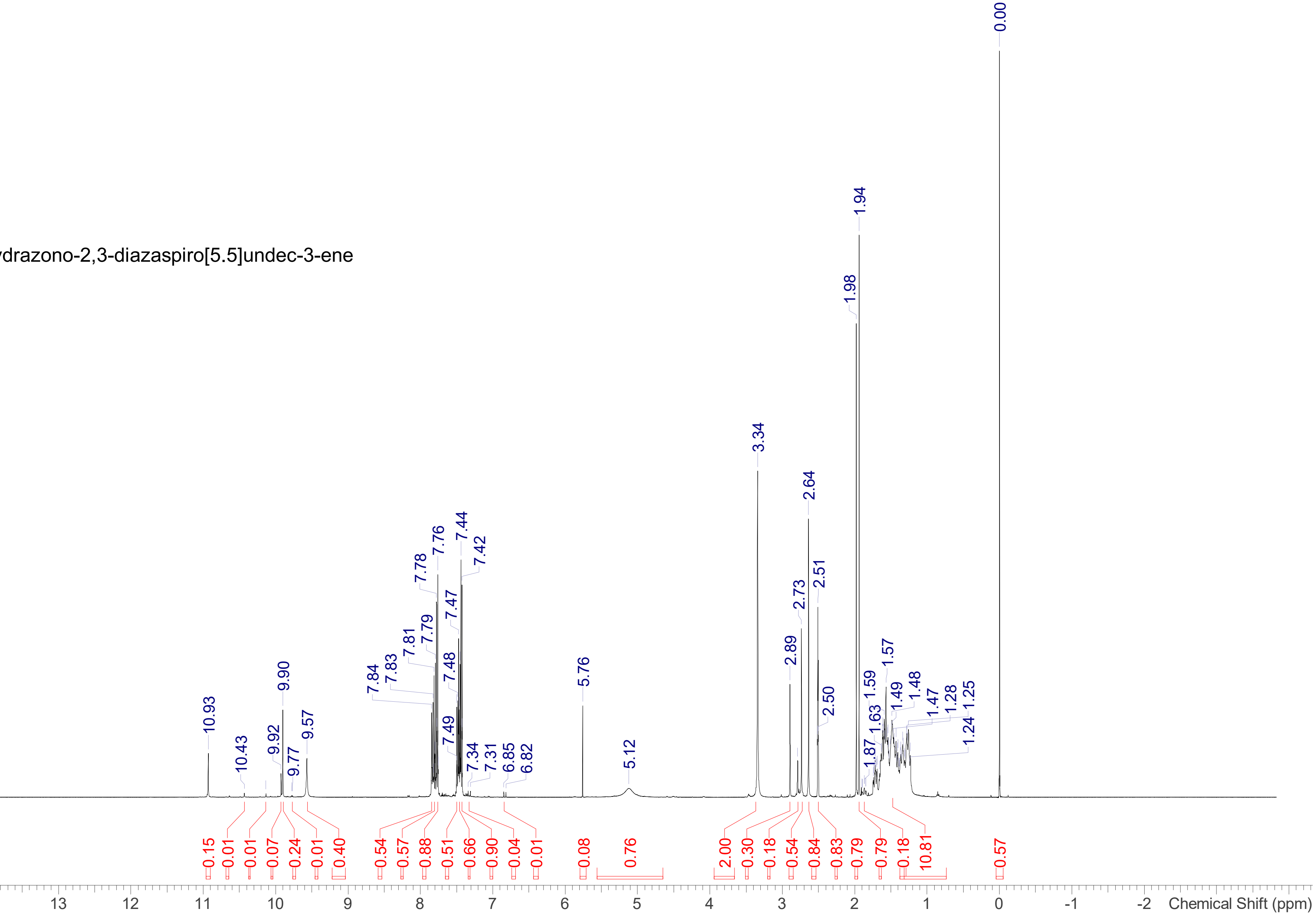

^{1}H NMR (499.9 MHz; DMSO-d_6)

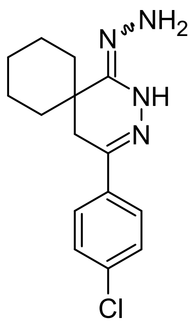

8c

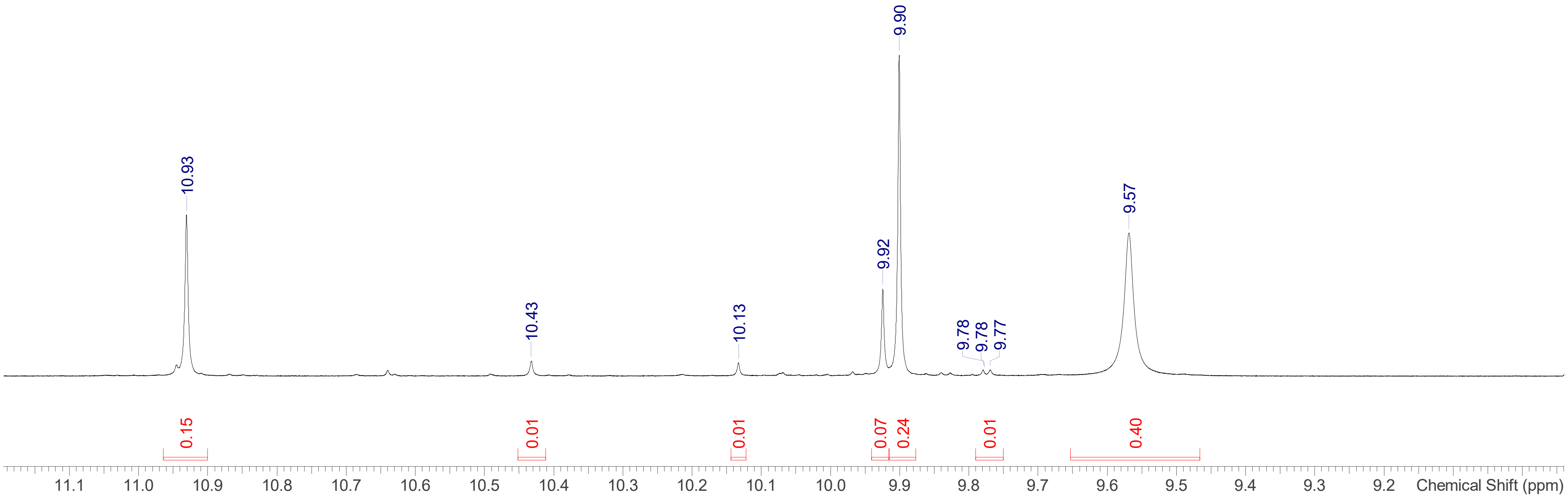

^{1}H NMR (499.9 MHz; DMSO-d_6)

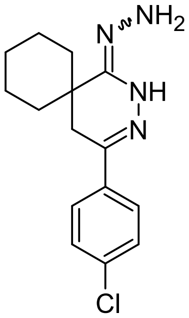

8c

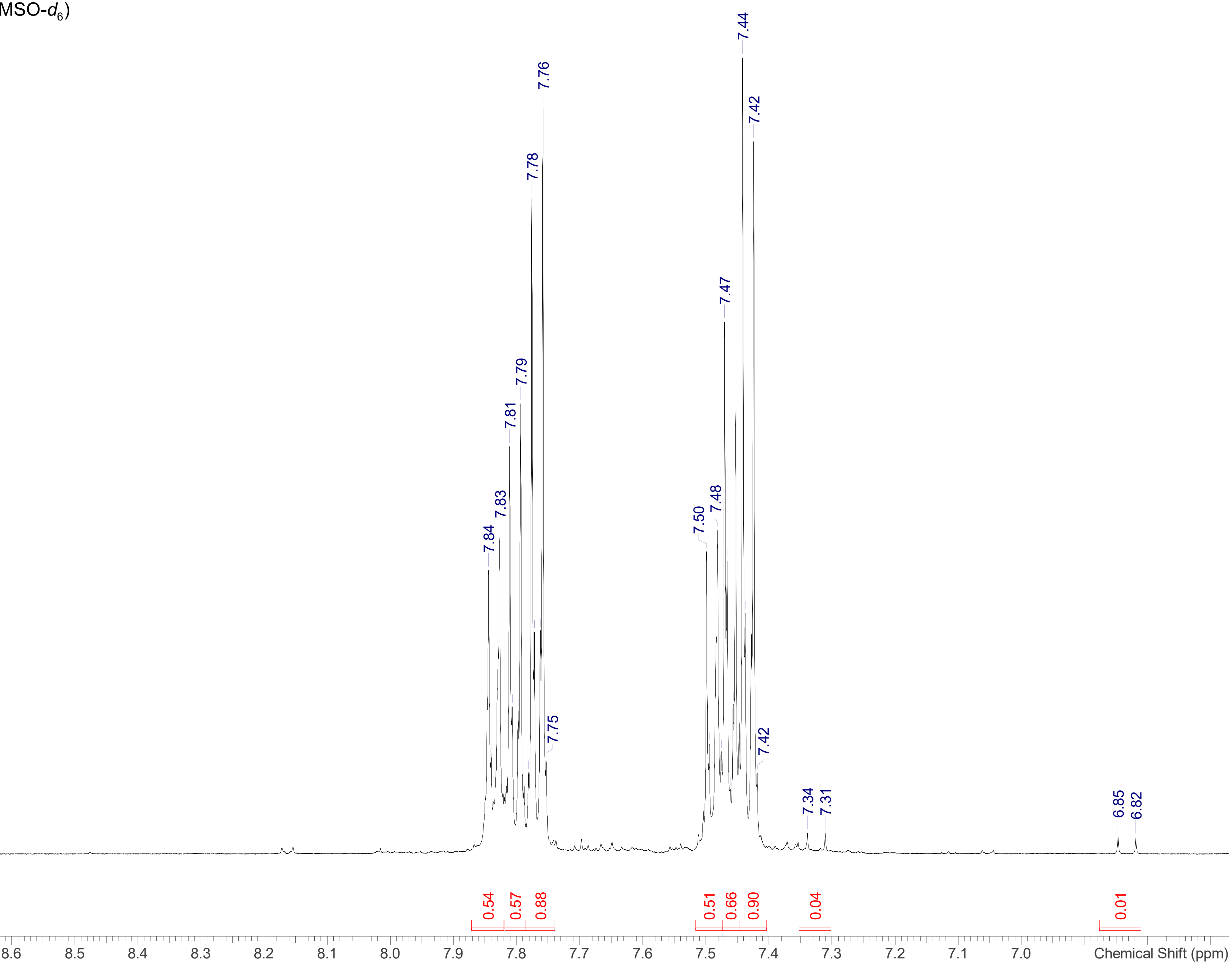

^{1}H NMR (499.9 MHz; DMSO-d_6)

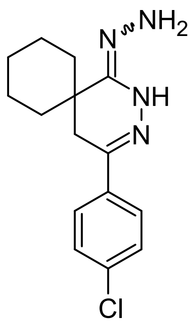

8c

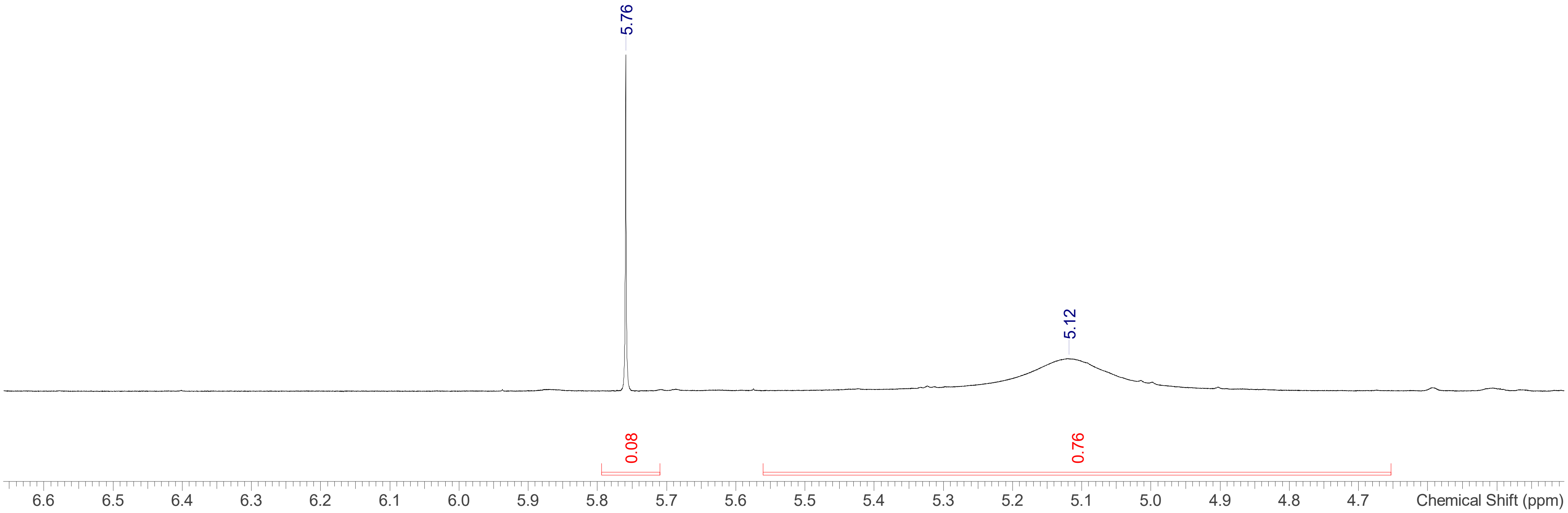

^{1}H NMR (499.9 MHz; DMSO-d_6)

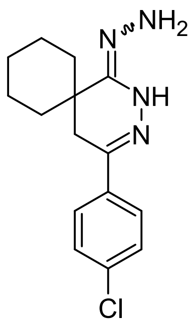

8c

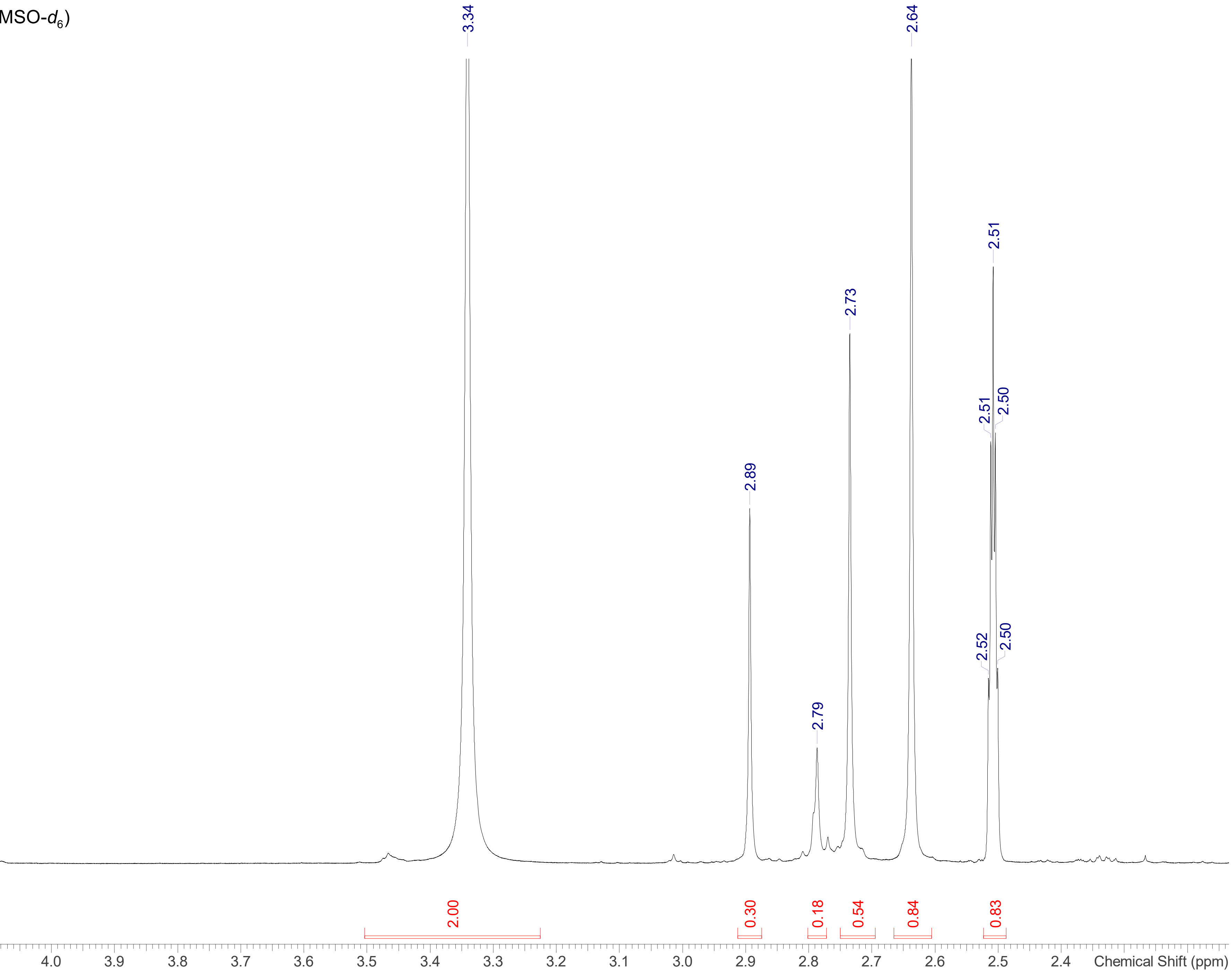

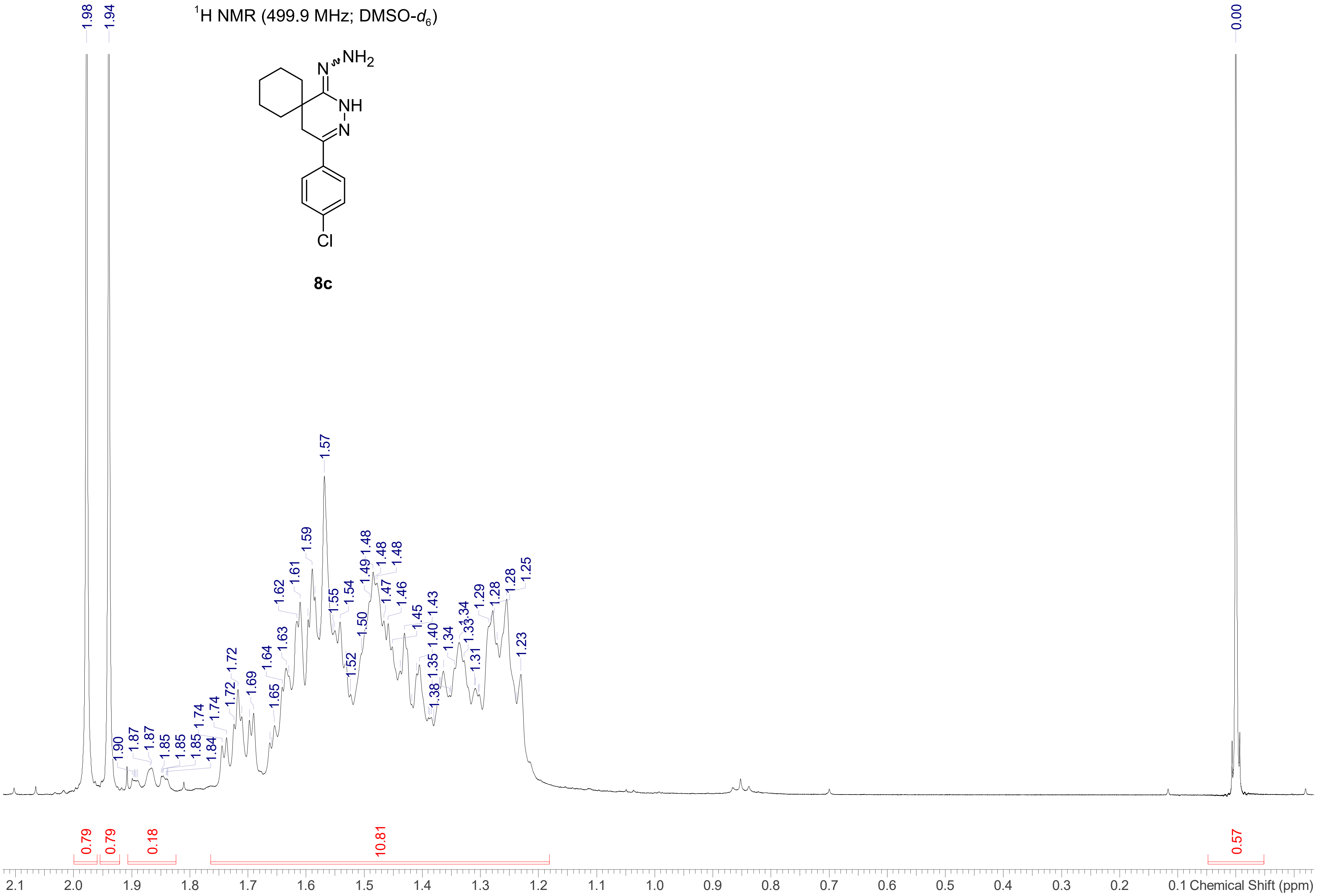

1H NMR (499.9 MHz; DMSO-d6)
NH2
N
NH
N
Cl
8c
1.98
1.94
0.00
1.57
1.59
1.61
1.62
1.63
1.64
1.65
1.72
1.72
1.69
1.74
1.74
1.90
1.87
1.87
1.85
1.85
1.85
1.84
1.55
1.54
1.52
1.50
1.49
1.48
1.48
1.48
1.47
1.46
1.45
1.43
1.40
1.35
1.38
1.34
1.34
1.33
1.31
1.29
1.28
1.28
1.25
1.23
0.79
0.79
0.18
10.81
0.57
2.1 2.0 1.9 1.8 1.7 1.6 1.5 1.4 1.3 1.2 1.1 1.0 0.9 0.8 0.7 0.6 0.5 0.4 0.3 0.2 0.1 Chemical Shift (ppm)

^{13}C NMR (125.7 MHz; DMSO-d_6)

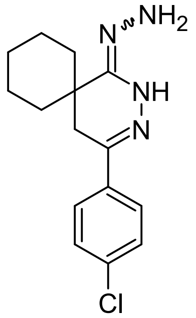

8c

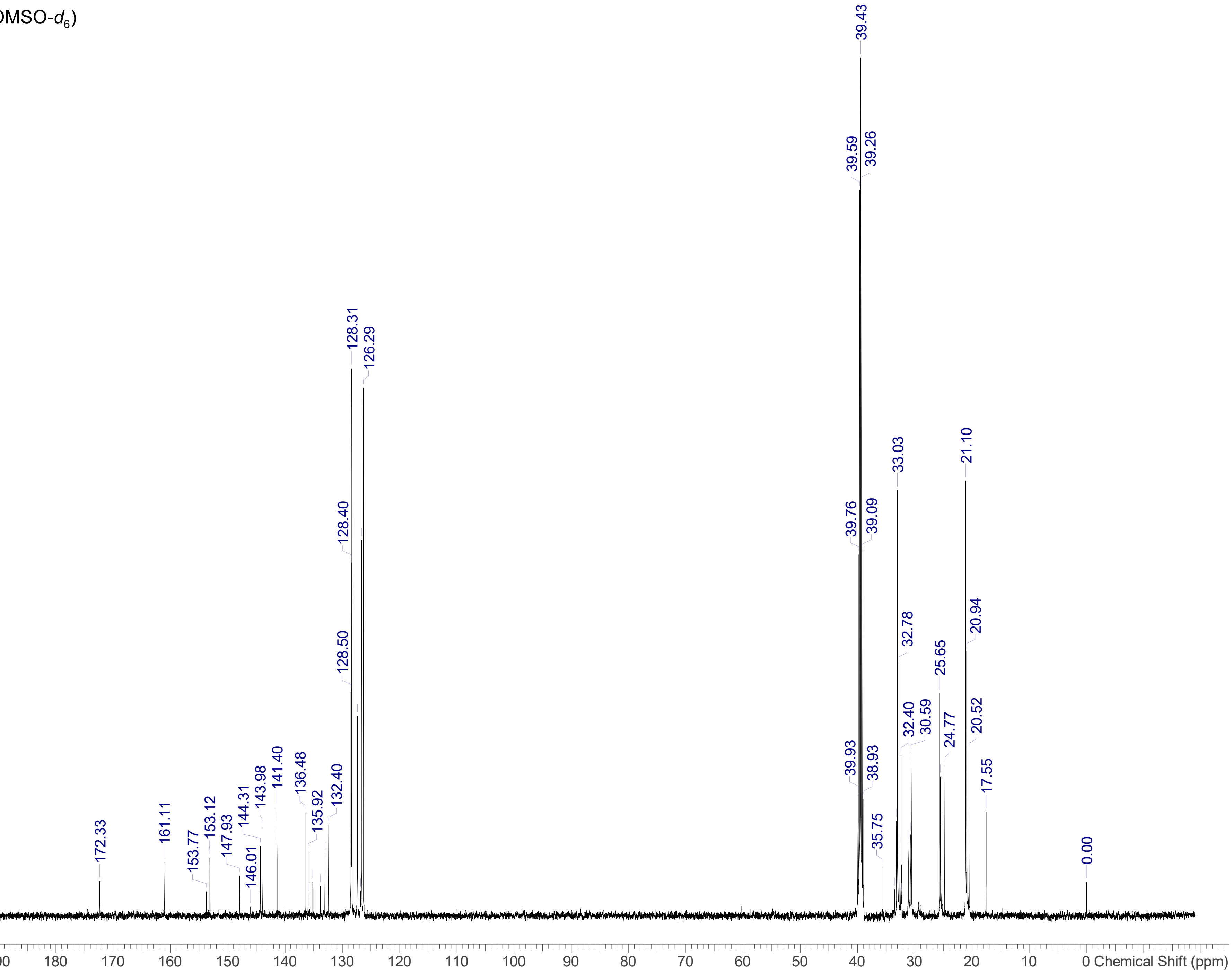

^{13}C NMR (125.7 MHz; DMSO-d_6)

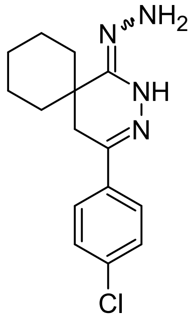

8c

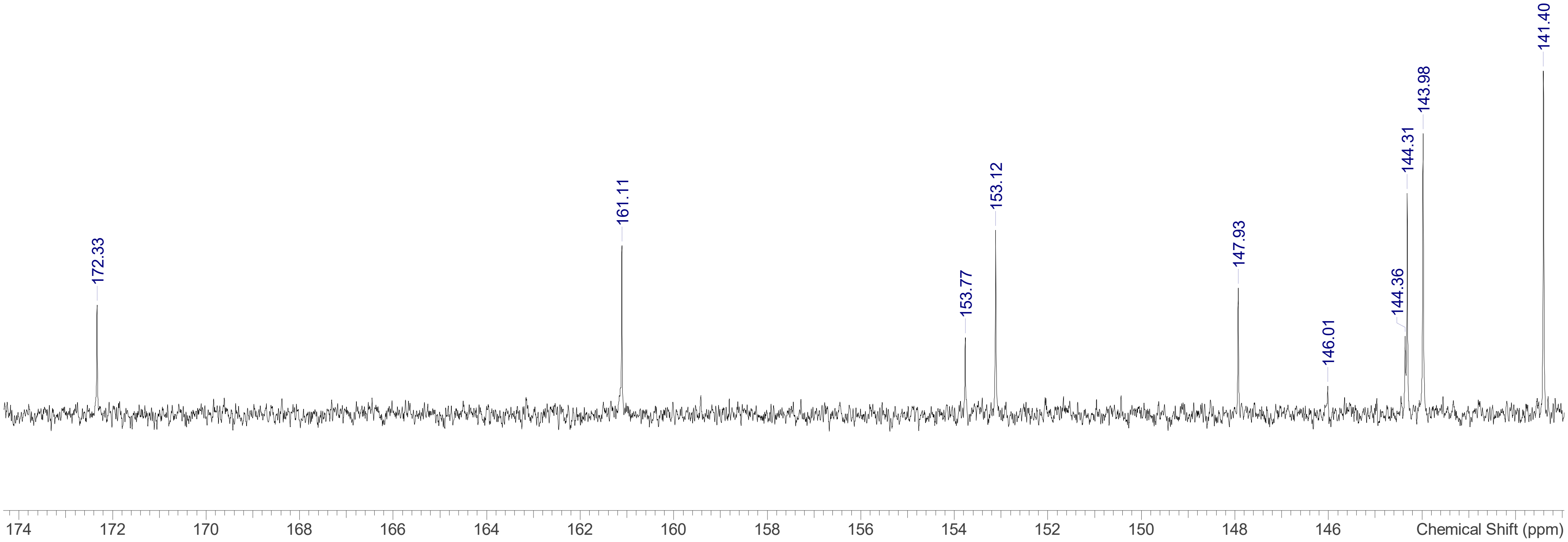

^{13}C NMR (125.7 MHz; DMSO-d_6)

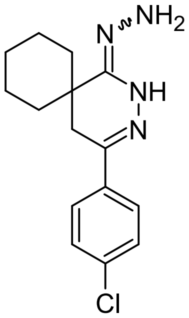

8c

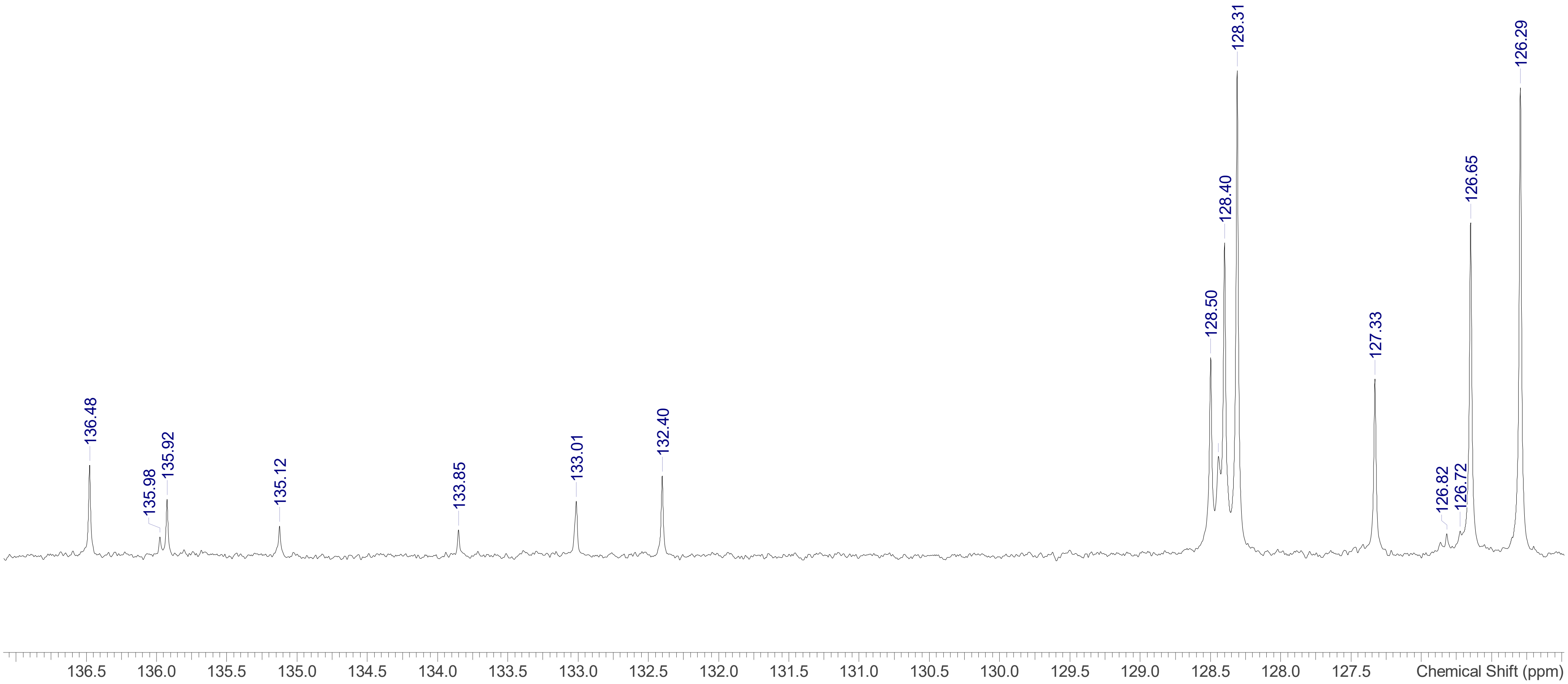

^{13}C NMR (125.7 MHz; DMSO-d_6)

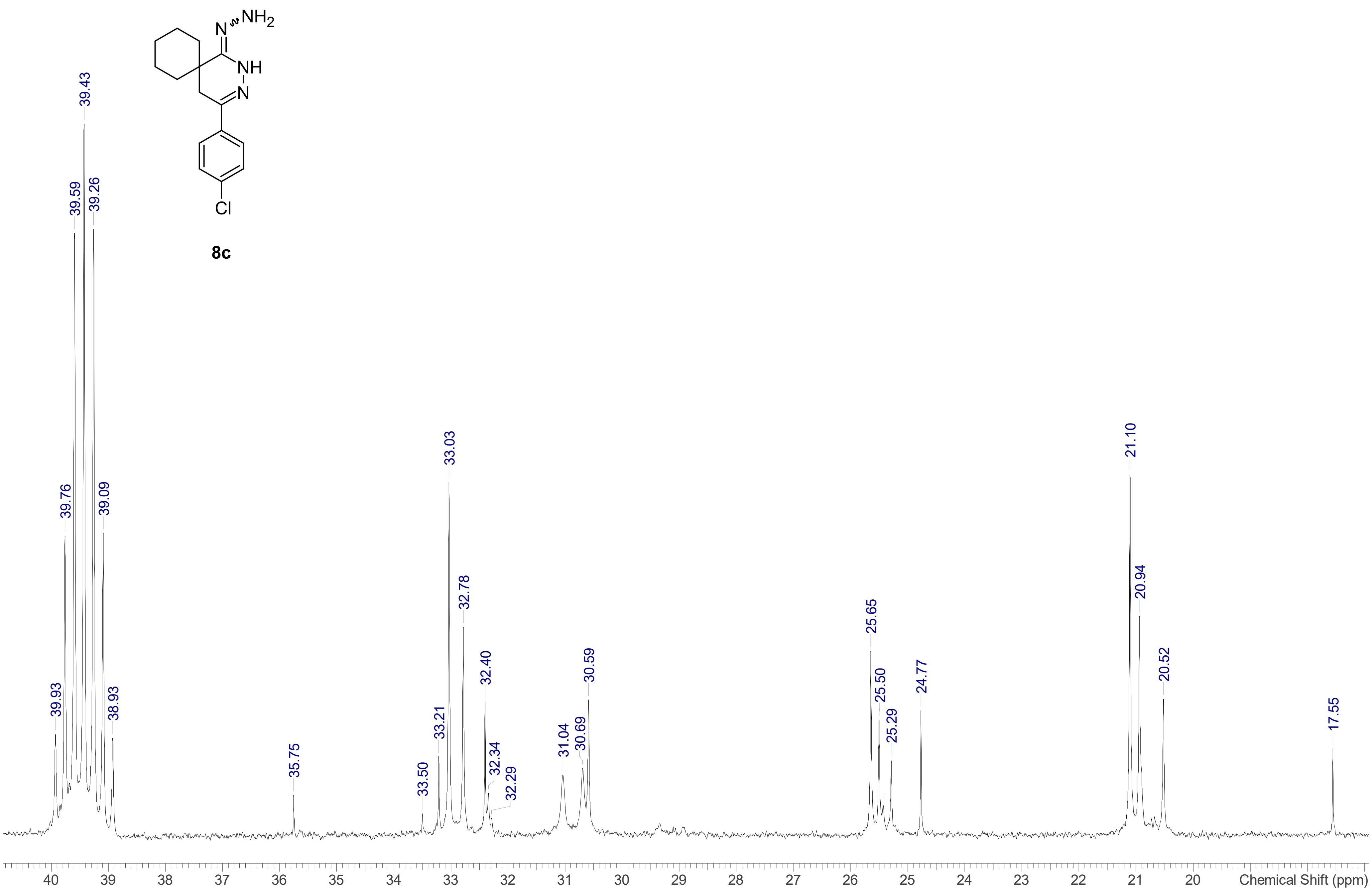

Compound **8c**

HRMS spectrum (ESI)

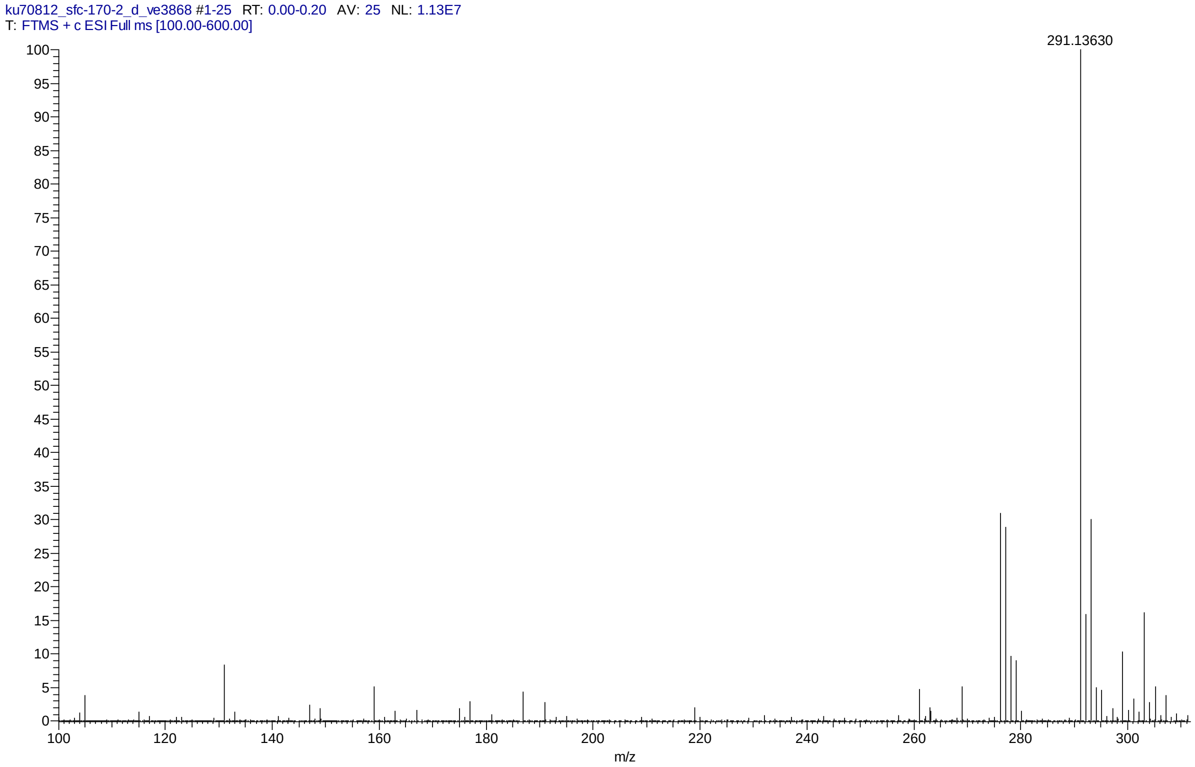

HRMS spectrum (ESI daughter ion)

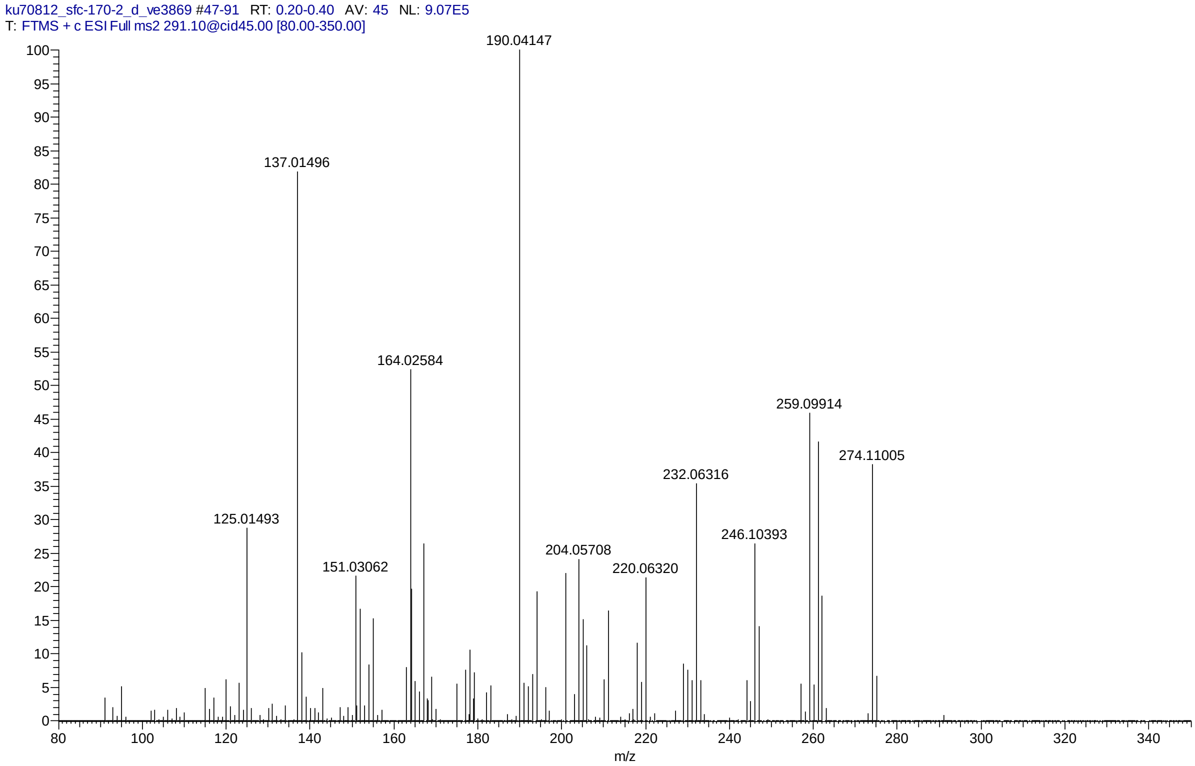

^1H NMR (499.9 MHz; DMSO-d_6)

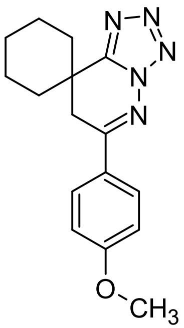

9a

6'-(4-Methoxyphenyl)-7'*H*-spiro[cyclohexane-1,8'-tetrazolo[1,5-*b*]pyridazine]

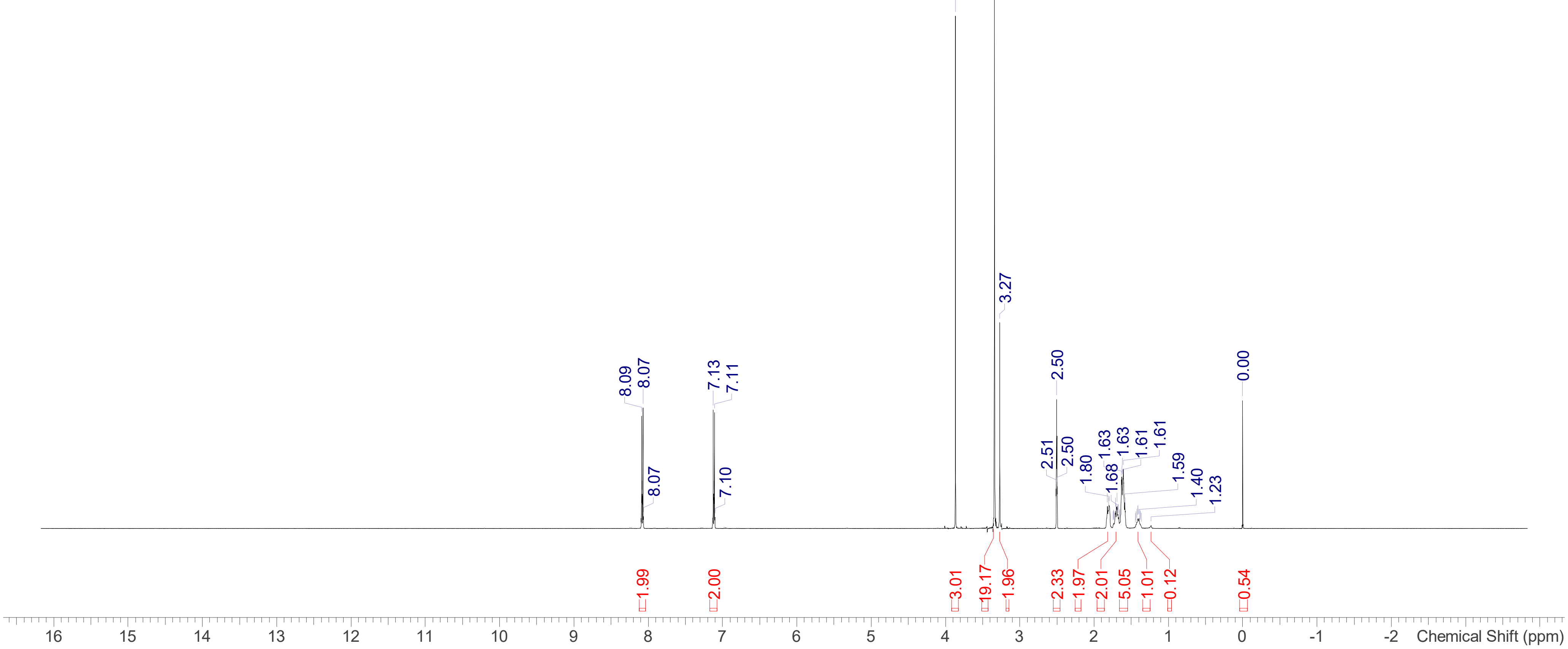

^{1}H NMR (499.9 MHz; DMSO-d_6)

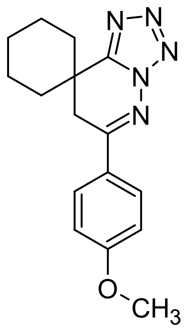

9a

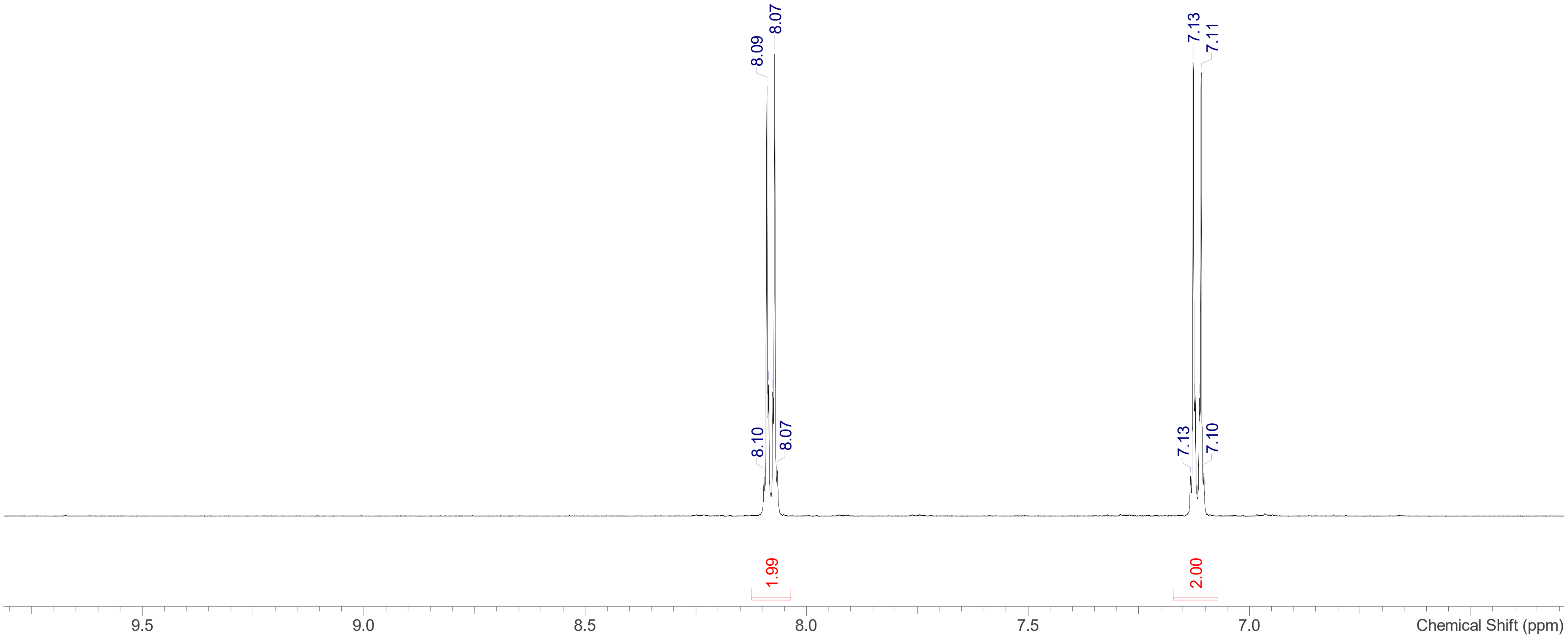

^{1}H NMR (499.9 MHz; DMSO-d_6)

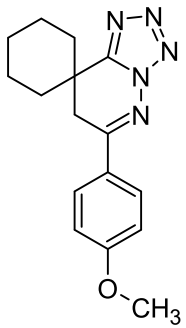

9a

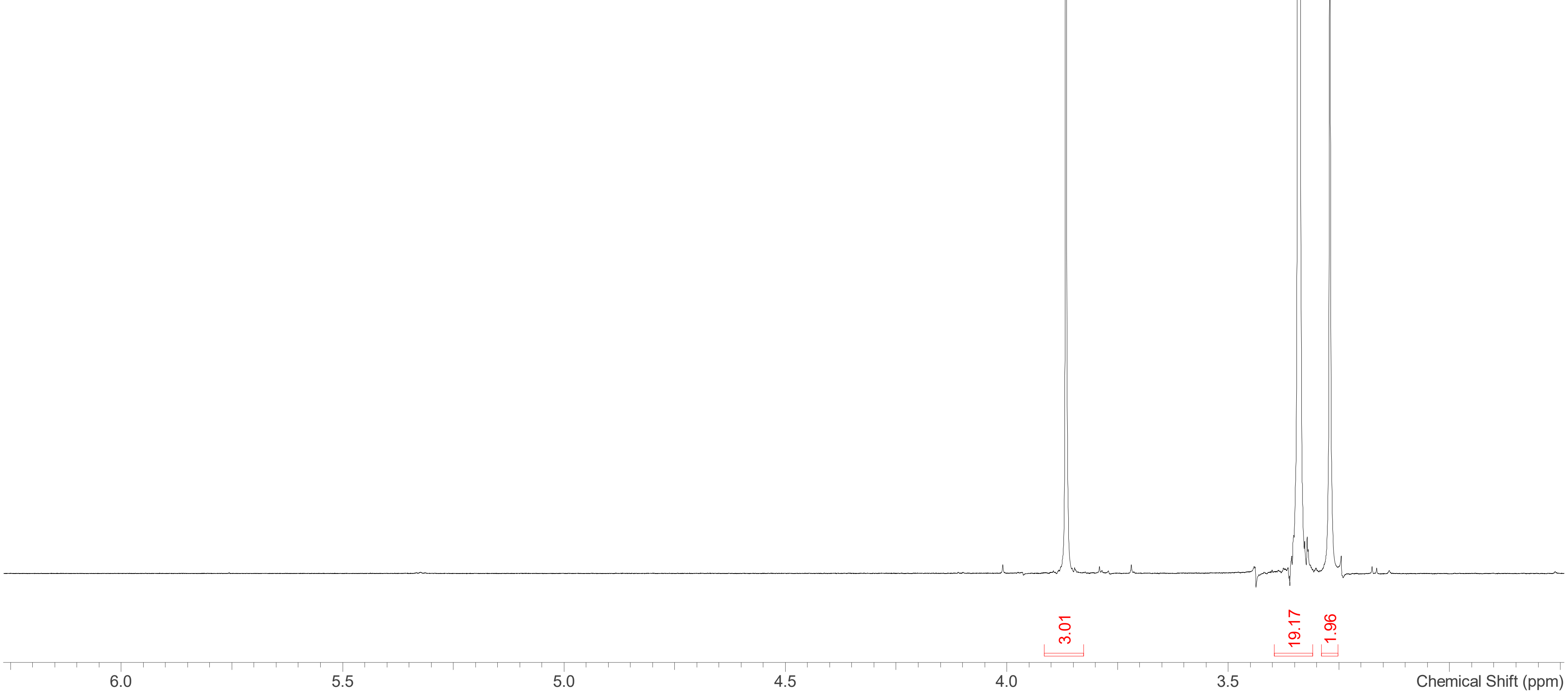

^{1}H NMR (499.9 MHz; DMSO-d_6)

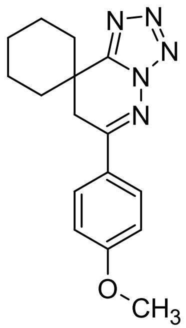

9a

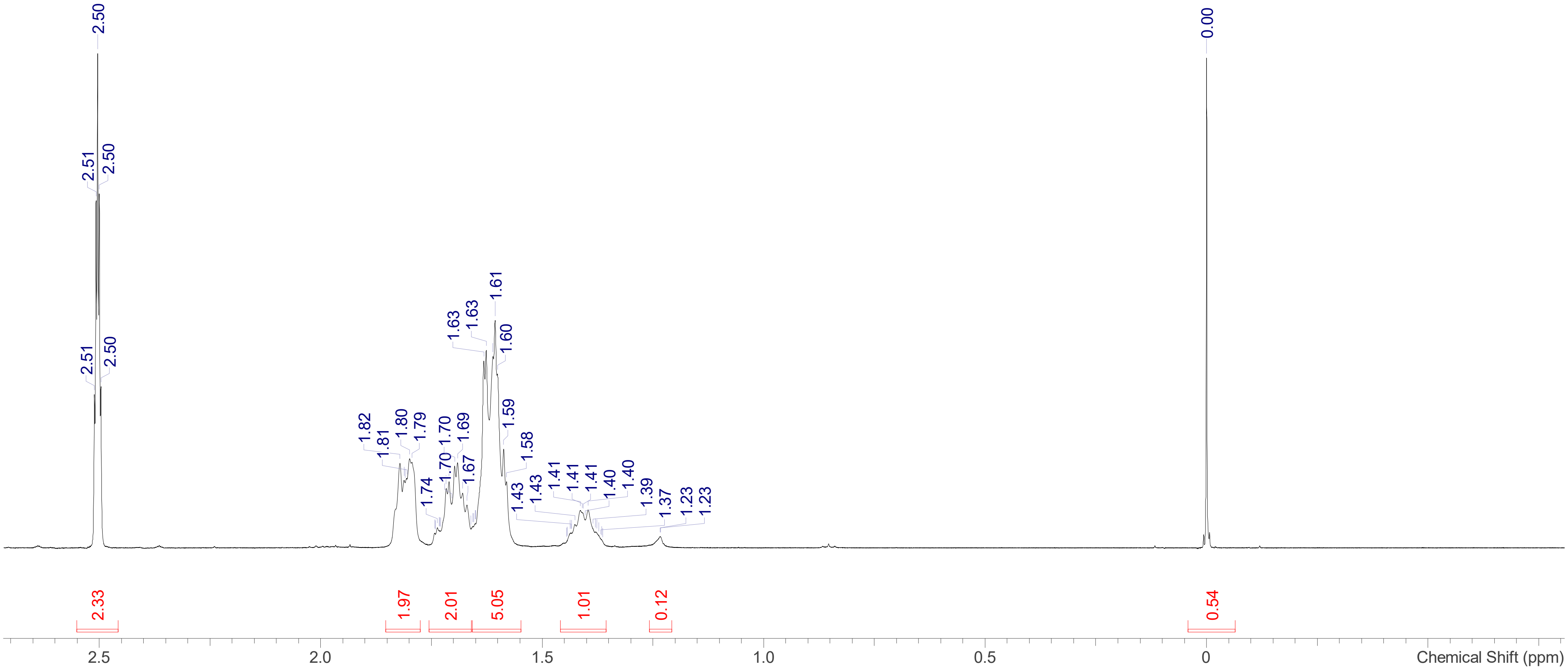

^{13}C NMR (125.7 MHz; DMSO-d_6)

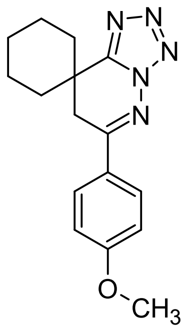

9a

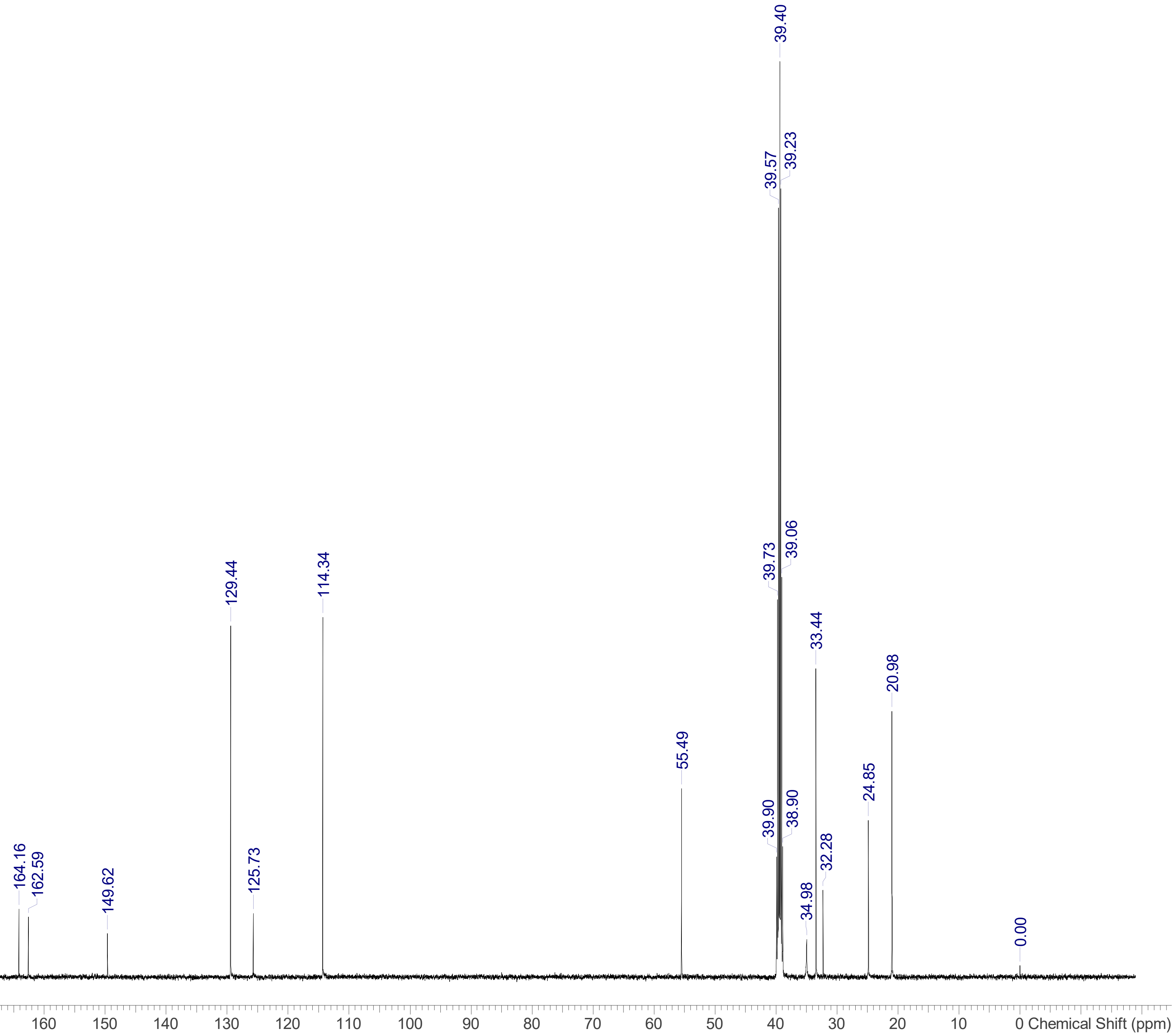

Compound **9a**

HRMS spectrum (ESI)

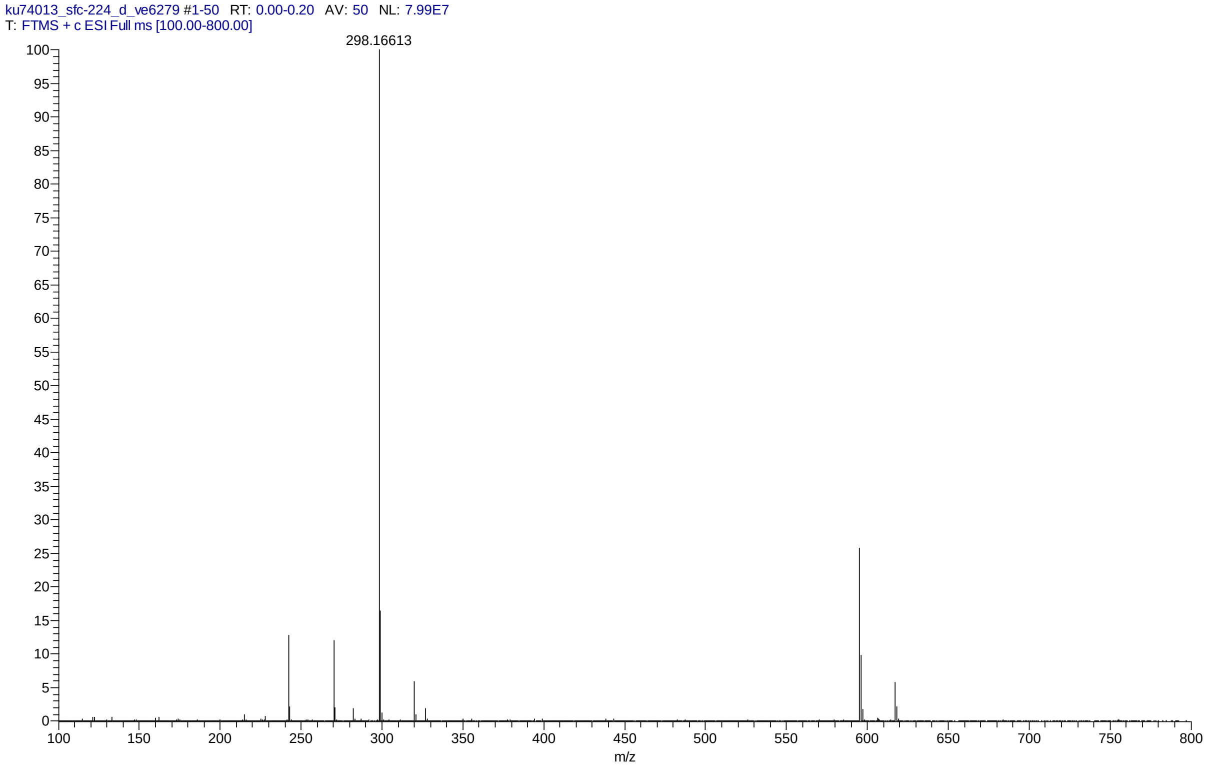

HRMS spectrum (ESI daughter ion)

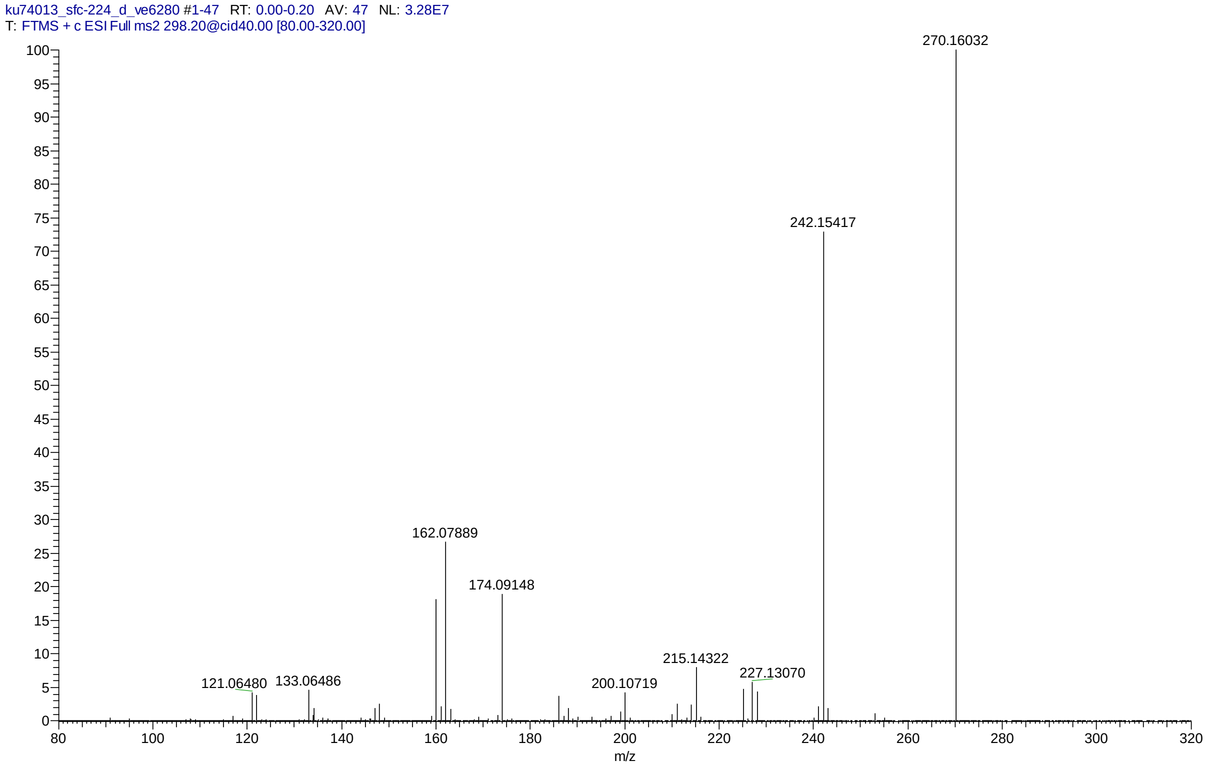

^{1}H NMR (499.9 MHz; DMSO-d_6)

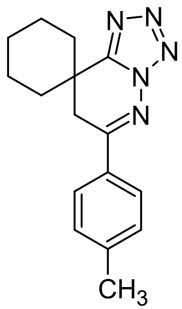

9b

6'-(*p*-Tolyl)-7'*H*-spiro[cyclohexane-1,8'-tetrazolo[1,5-*b*]pyridazine]

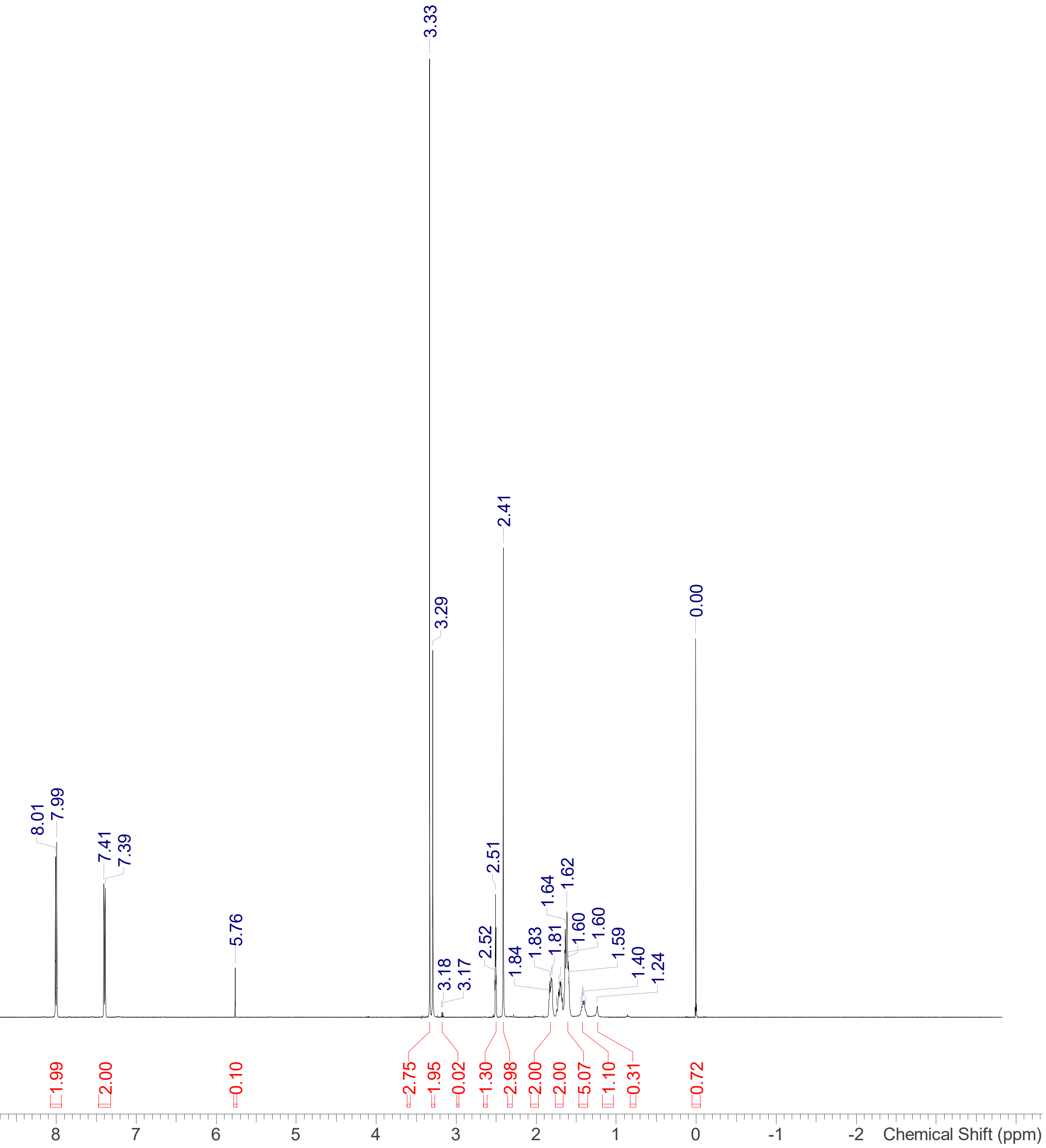

^{1}H NMR (499.9 MHz; DMSO-d_6)

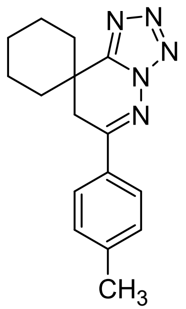

9b

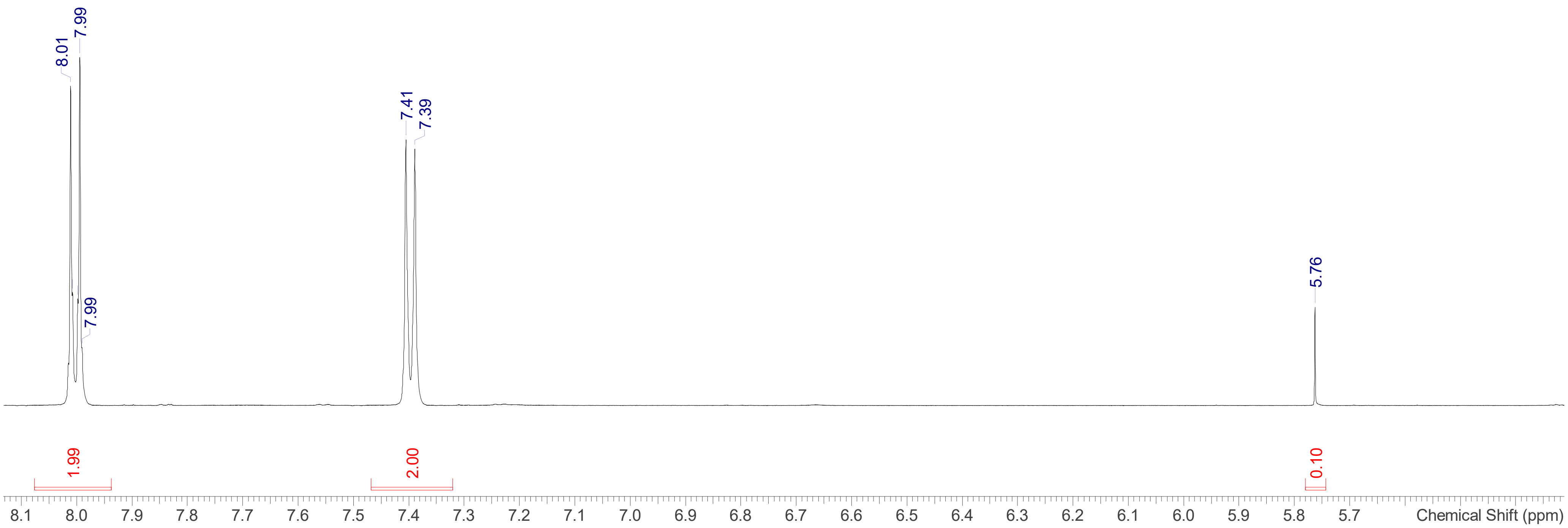

^{1}H NMR (499.9 MHz; DMSO-d_6)

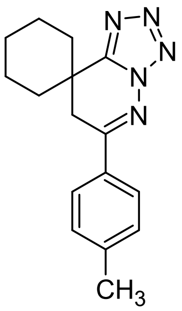

9b

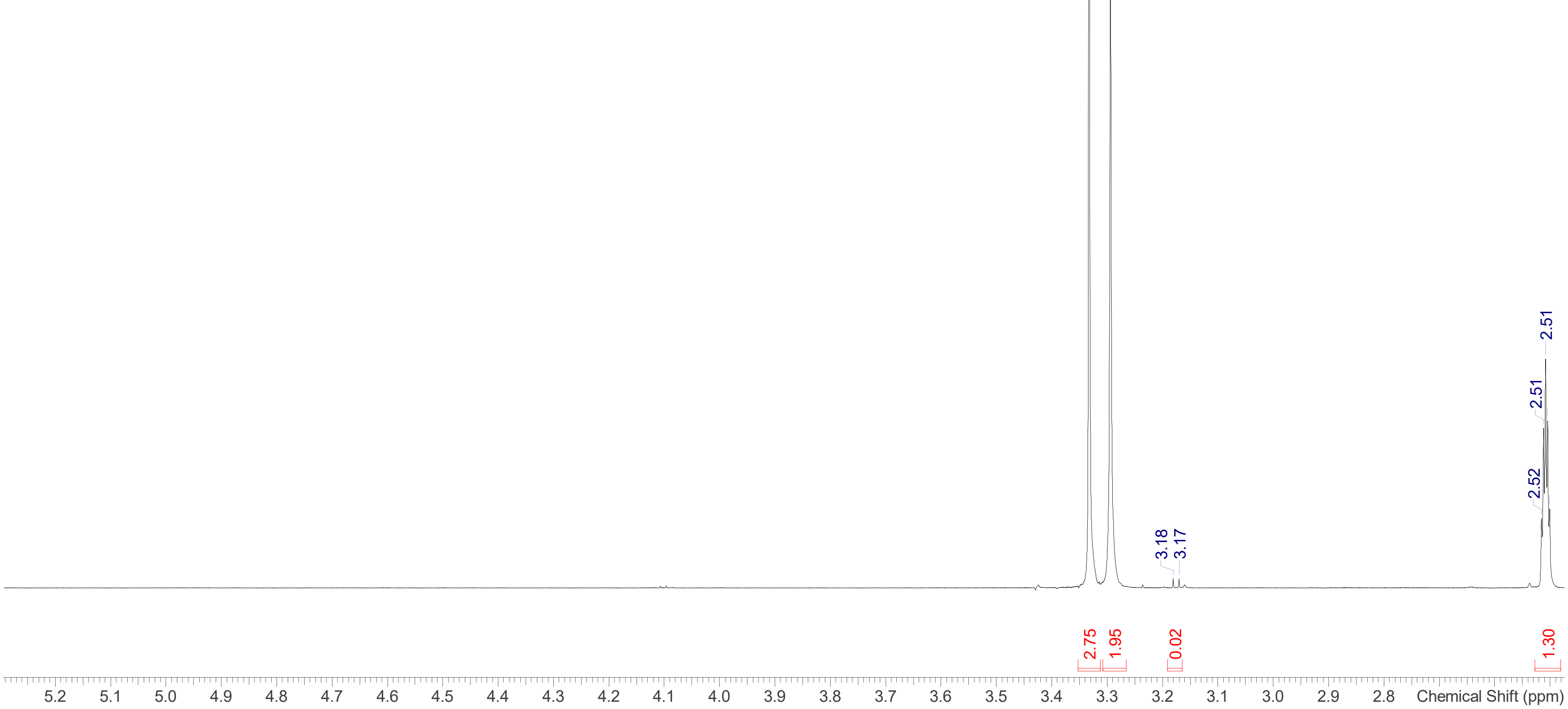

^{1}H NMR (499.9 MHz; DMSO-d_6)

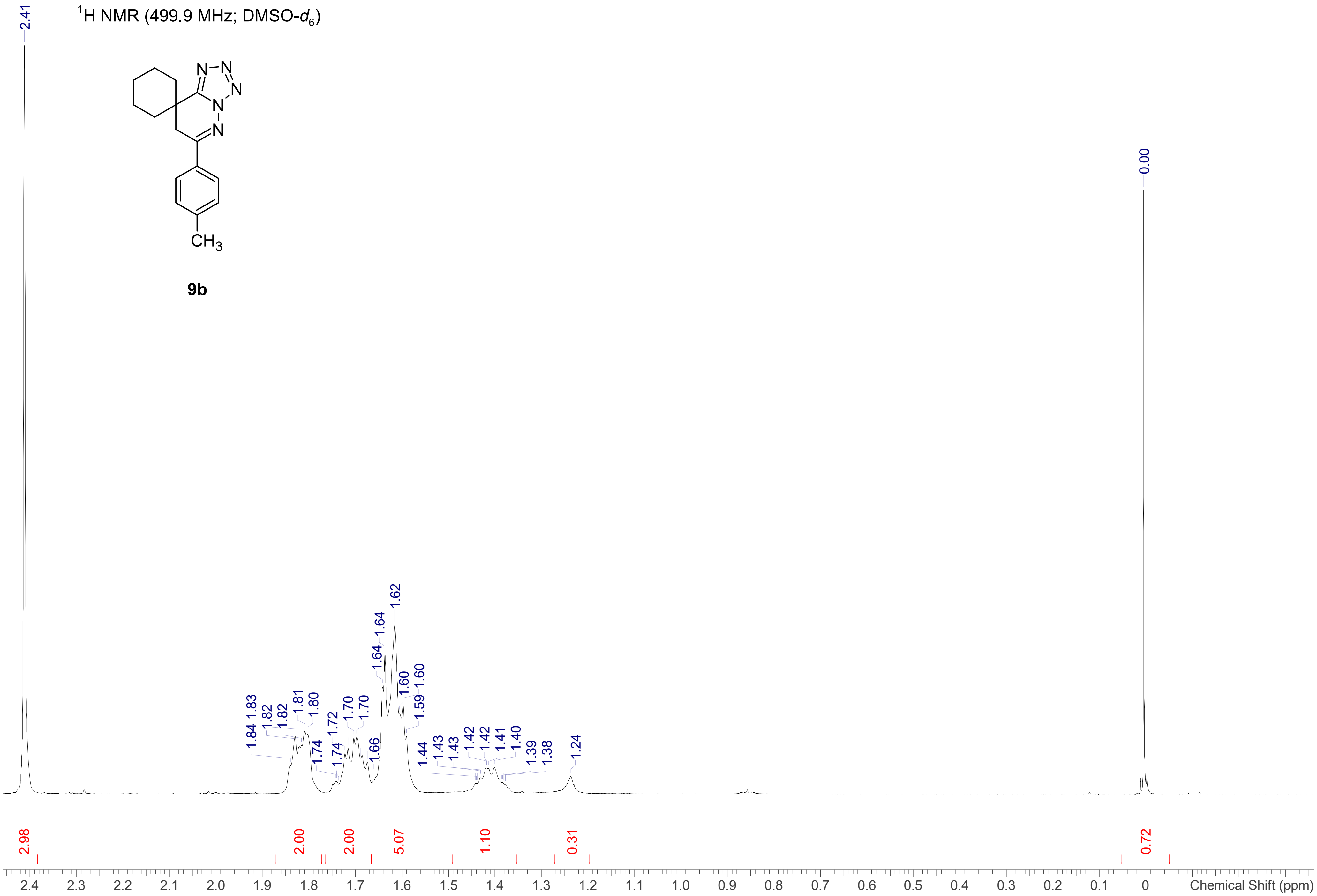

9b

^{13}C NMR (125.7 MHz; DMSO-d_6)

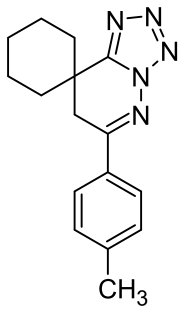

9b

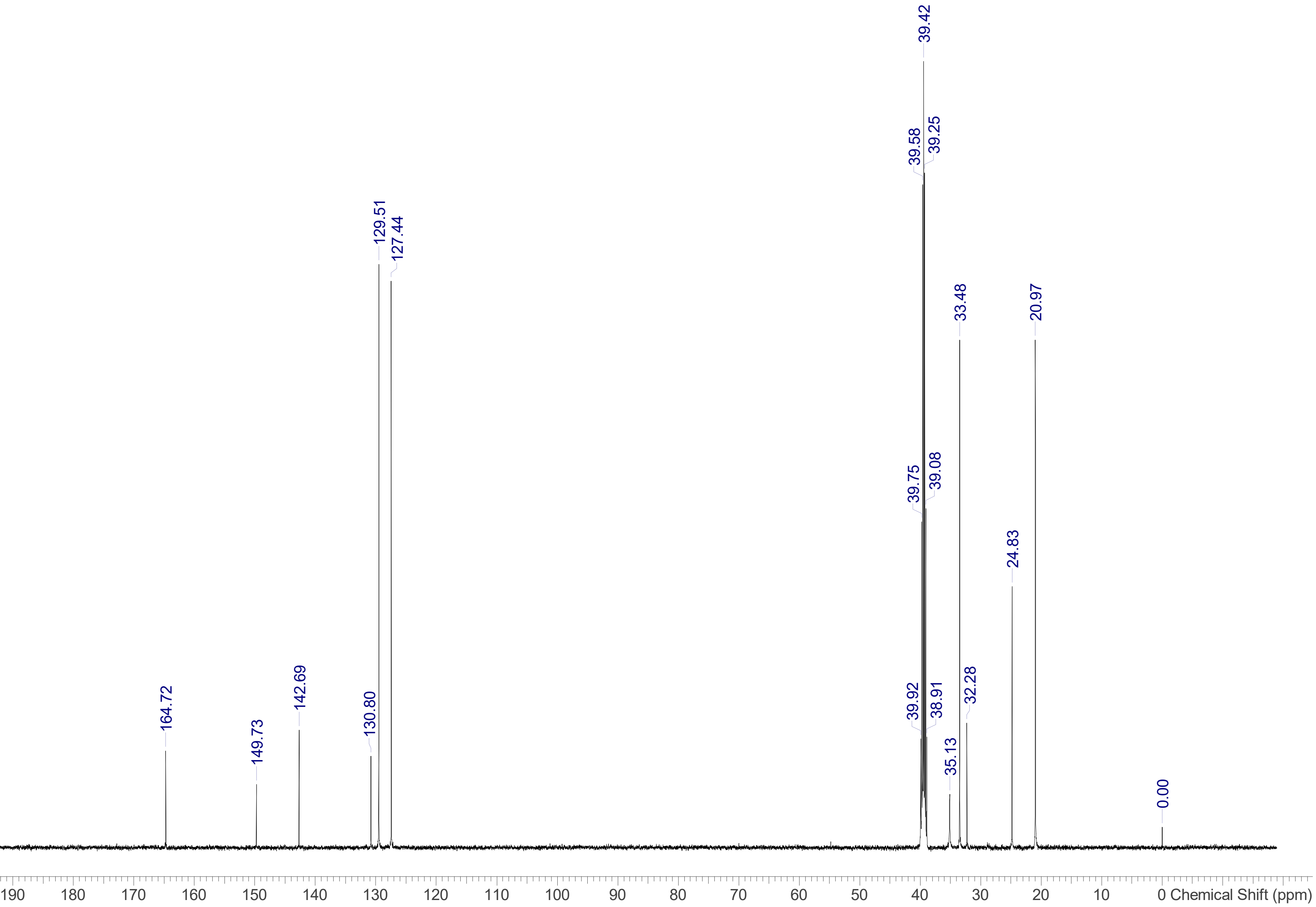

Compound **9b**

HRMS spectrum (ESI)

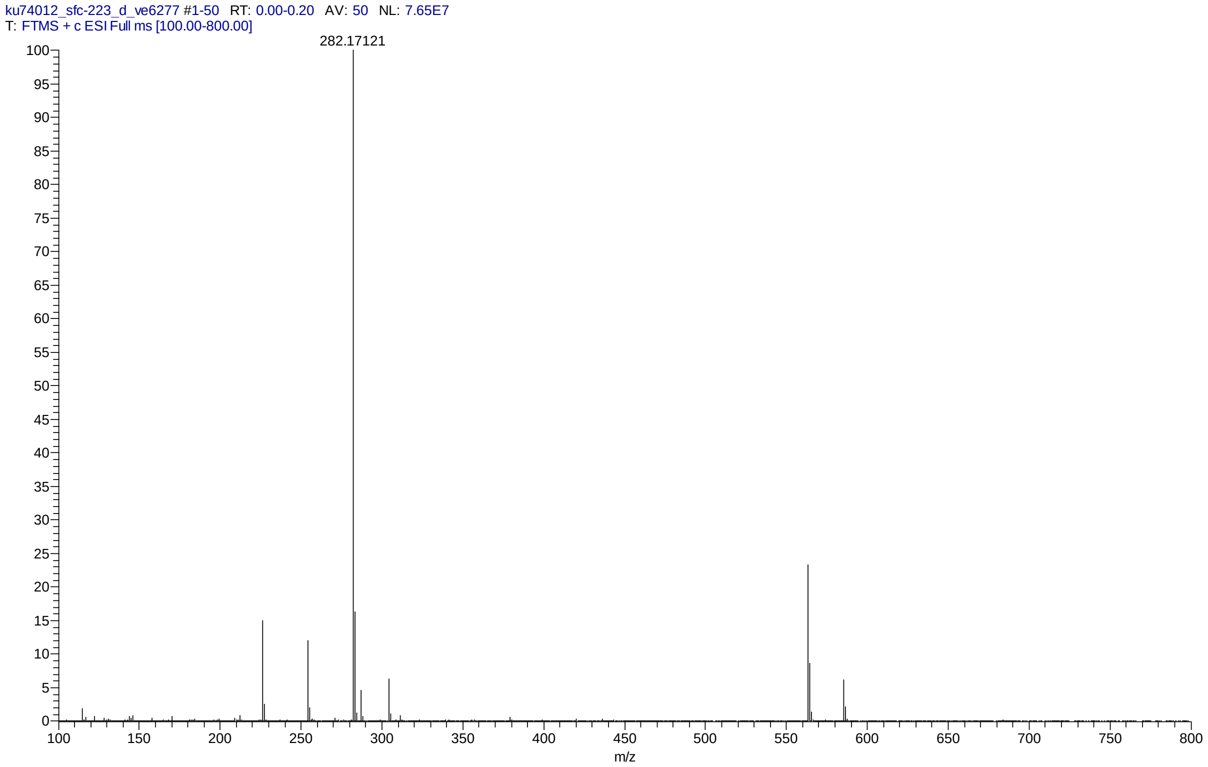

HRMS spectrum (ESI daughter ion)

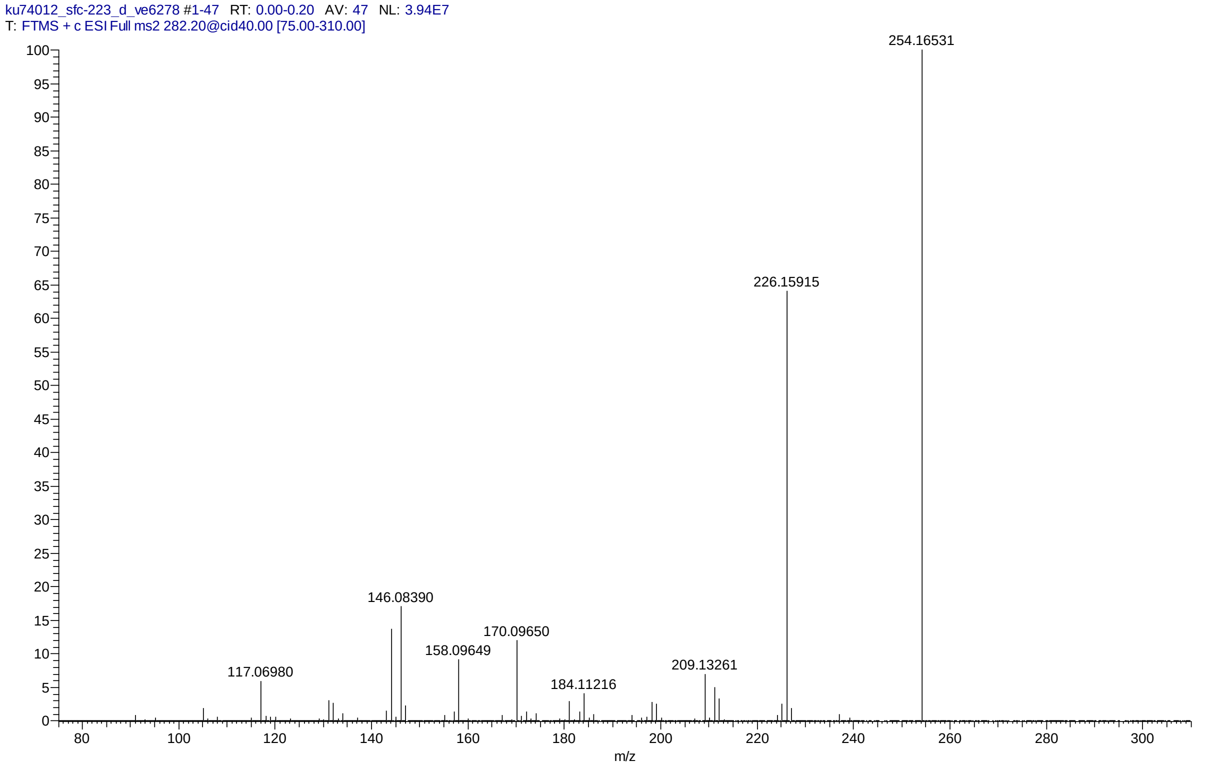

^{1}H NMR (499.9 MHz; DMSO-d_6)

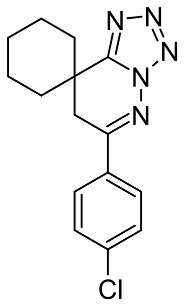

9c

6'-(4-Chlorophenyl)-7'*H*-spiro[cyclohexane-1,8'-tetrazolo[1,5-*b*]pyridazine]

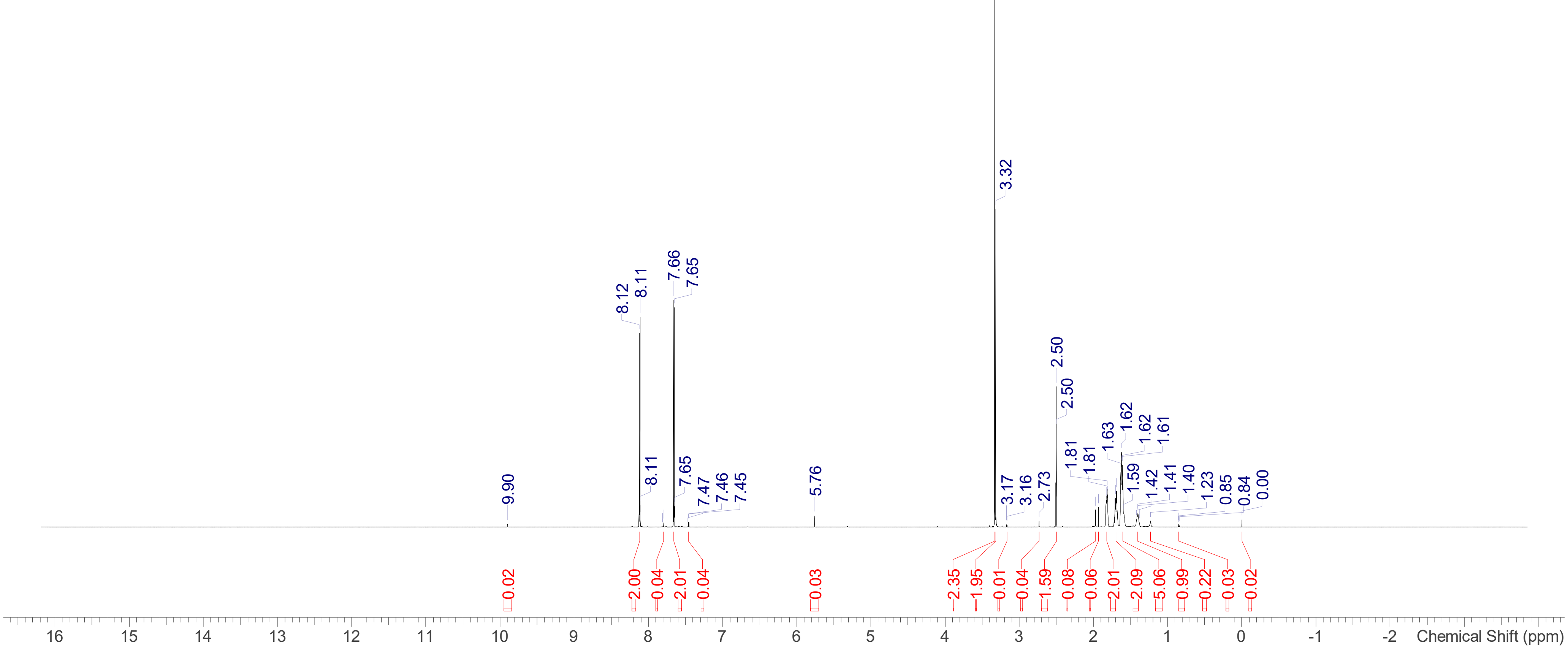

^{1}H NMR (499.9 MHz; DMSO-d_6)

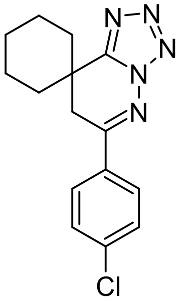

9c

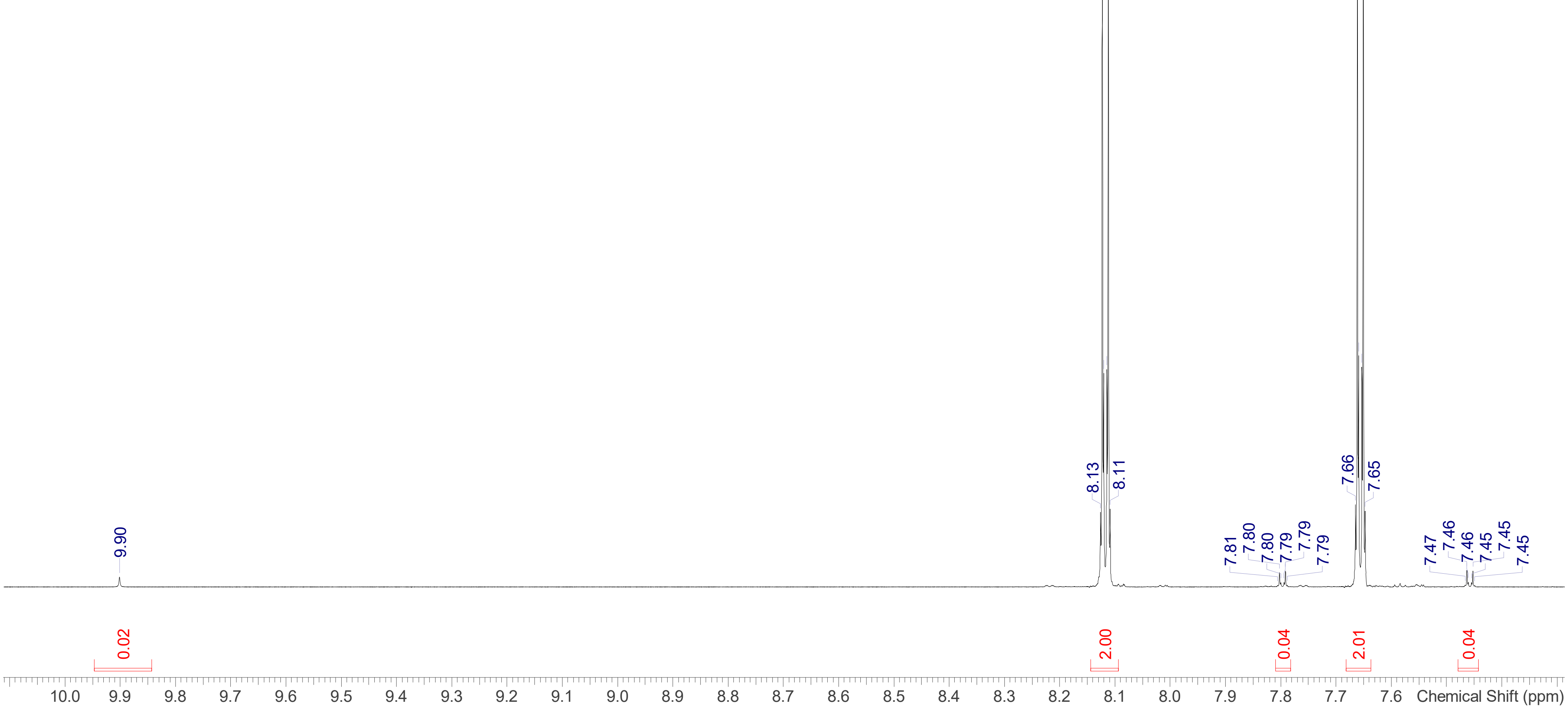

^{1}H NMR (499.9 MHz; DMSO-d_6)

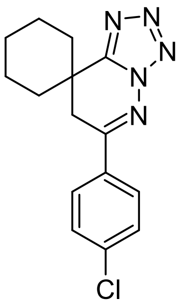

9c

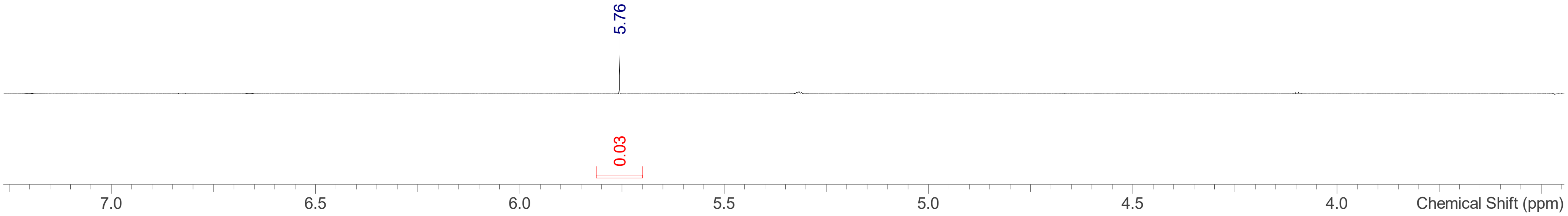

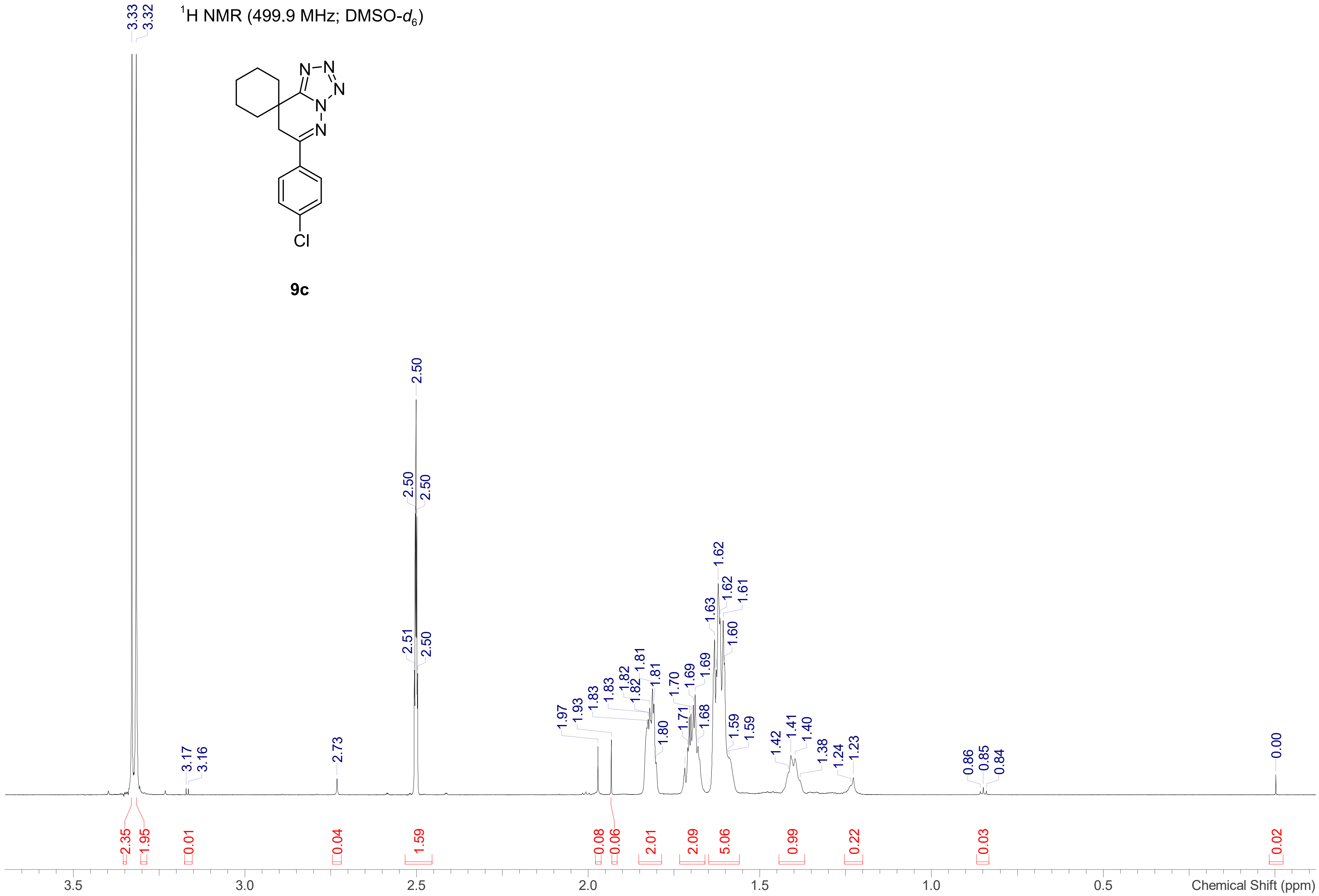

^{1}H NMR (499.9 MHz; DMSO-d_6)
N
N
N
N
N
Cl
9c
3.33
3.32
3.17
3.16
2.73
2.50
2.50
2.50
2.51
2.50
1.97
1.93
1.83
1.83
1.82
1.82
1.81
1.81
1.80
1.71
1.70
1.69
1.69
1.68
1.63
1.62
1.62
1.61
1.60
1.59
1.59
1.42
1.41
1.40
1.38
1.24
1.23
0.86
0.85
0.84
0.00
2.35
1.95
0.01
0.04
1.59
0.08
0.06
2.01
2.09
5.06
0.99
0.22
0.03
0.02
3.5
3.0
2.5
2.0
1.5
1.0
0.5
Chemical Shift (ppm)

^{13}C NMR (125.7 MHz; DMSO-d_6)

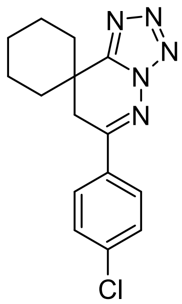

9c

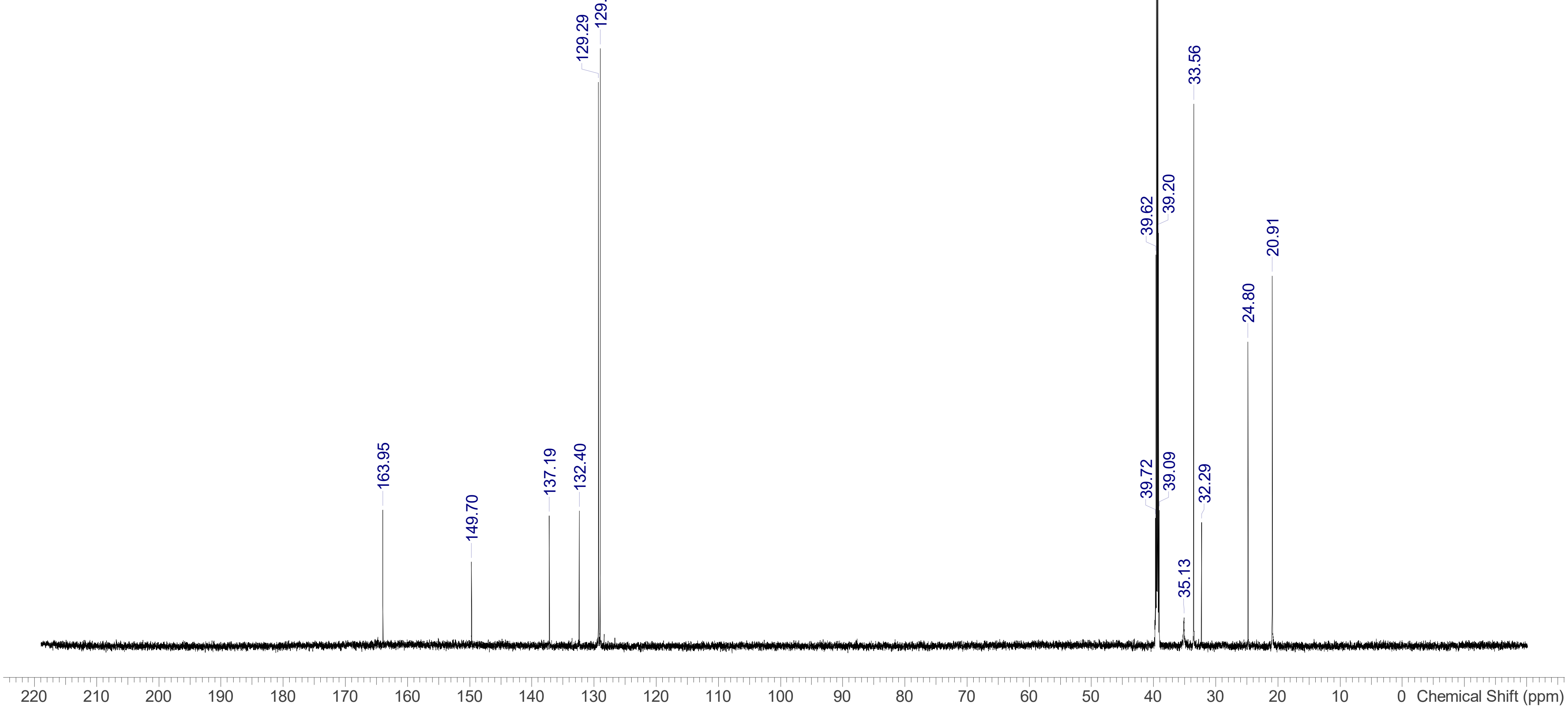

Compound **9c**

HRMS spectrum (ESI)

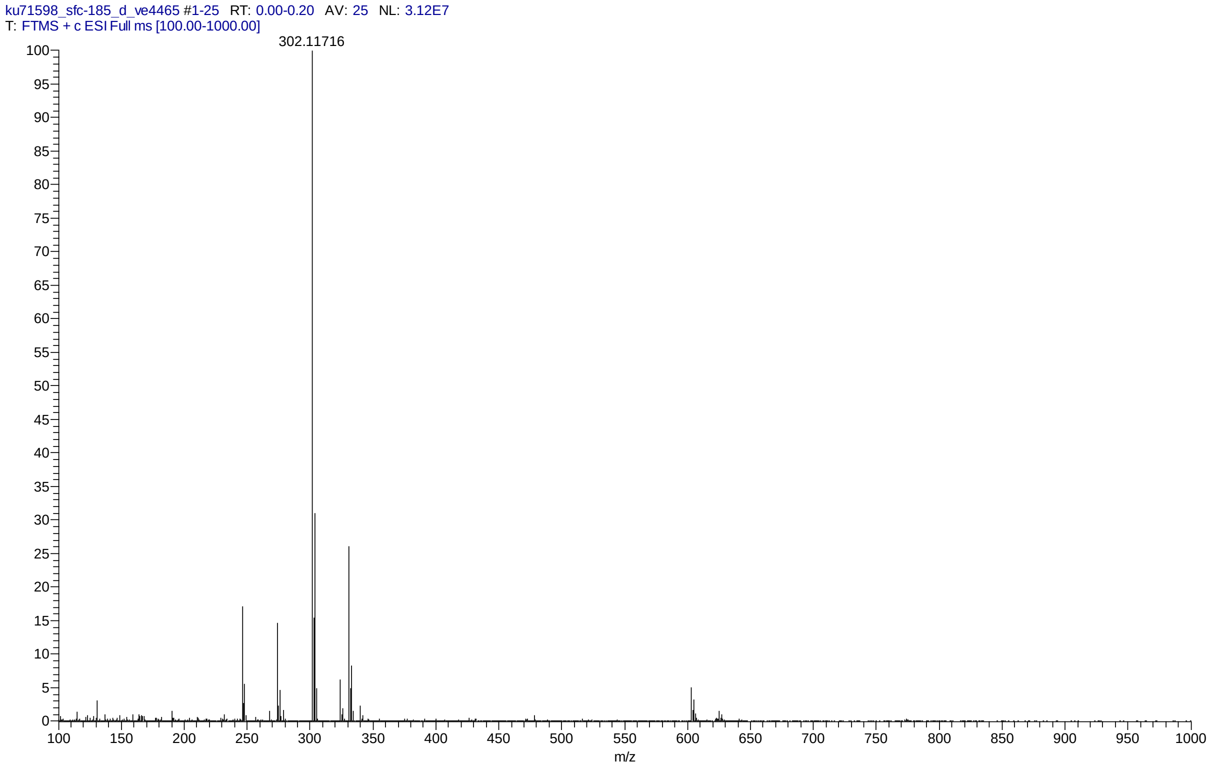

HRMS spectrum (ESI daughter ion)

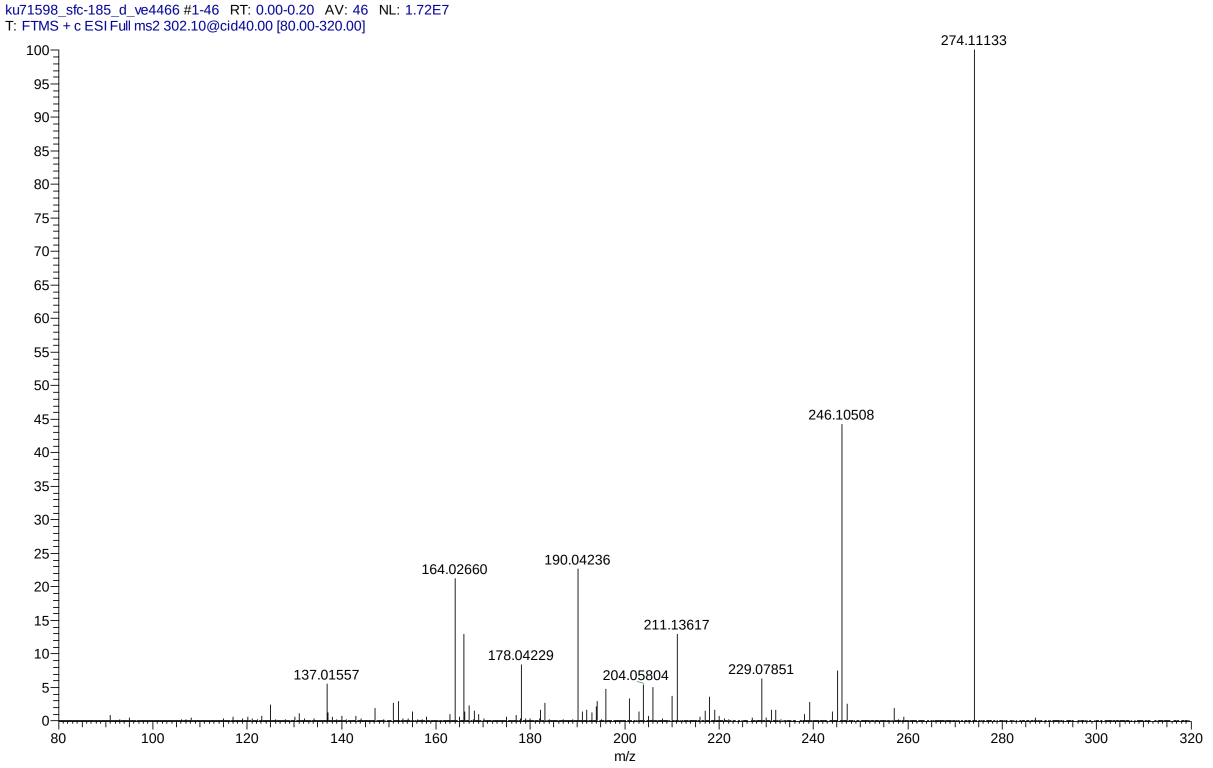

^{1}H NMR (799.7 MHz; DMSO-d_6)

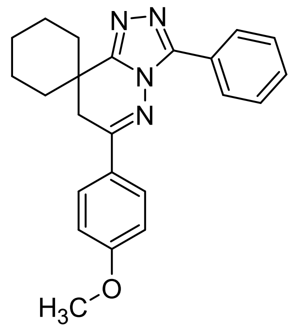

10a

6-(4-Methoxyphenyl)-3-phenyl-7*H*-spiro[[1,2,4]triazolo[4,3-*b*]pyridazine-8,1'-cyclohexane]

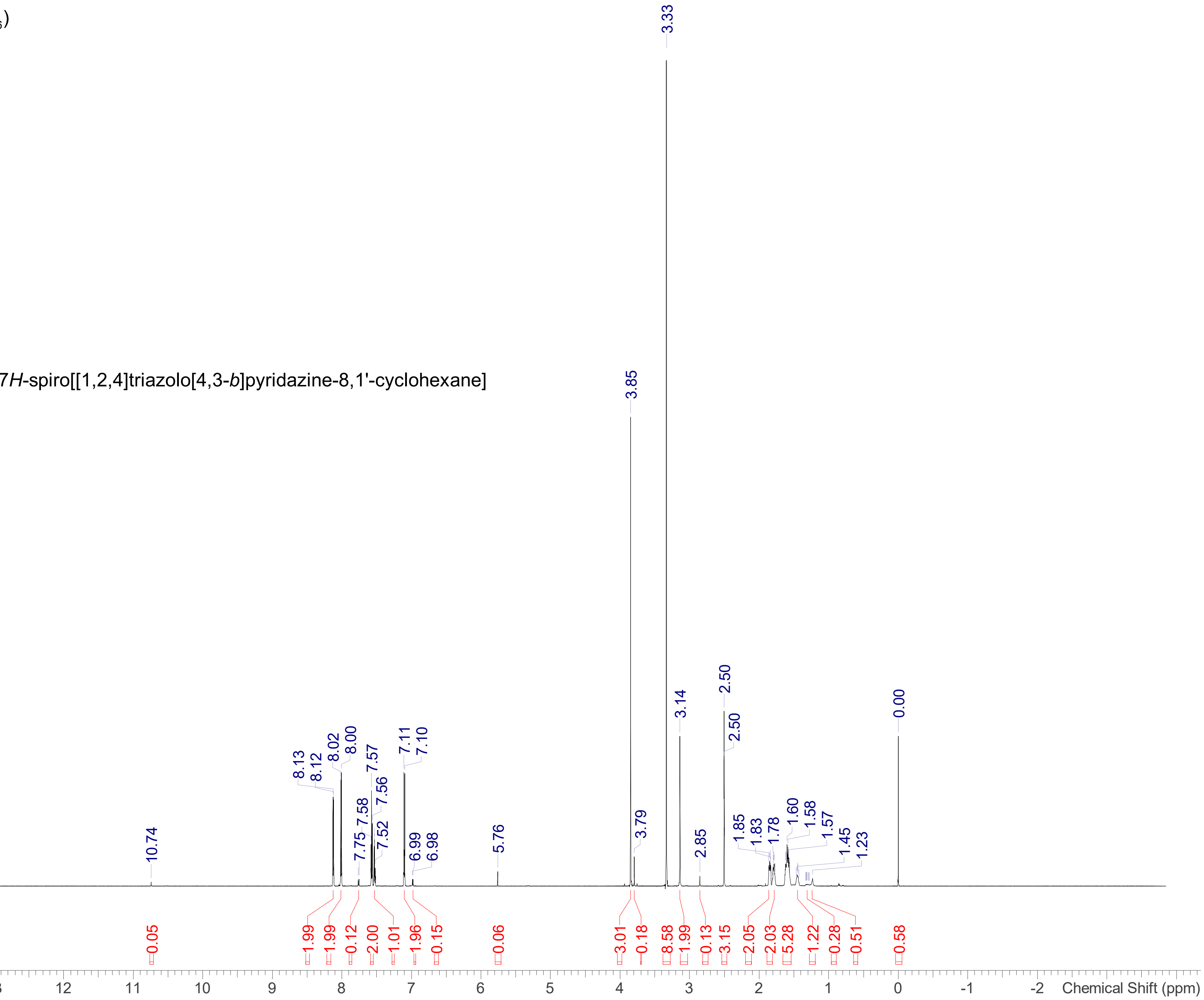

^{1}H NMR (799.7 MHz; DMSO-d_6)

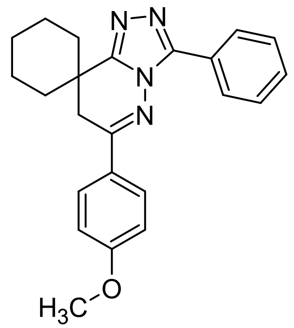

10a

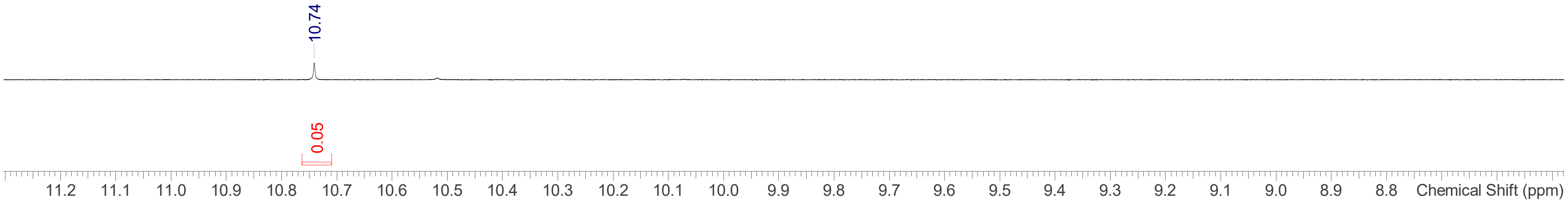

^{1}H NMR (799.7 MHz; DMSO-d_6)

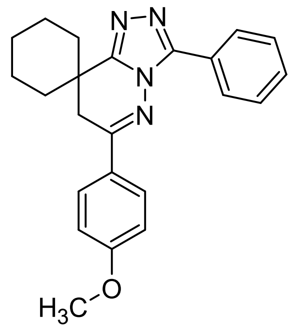

10a

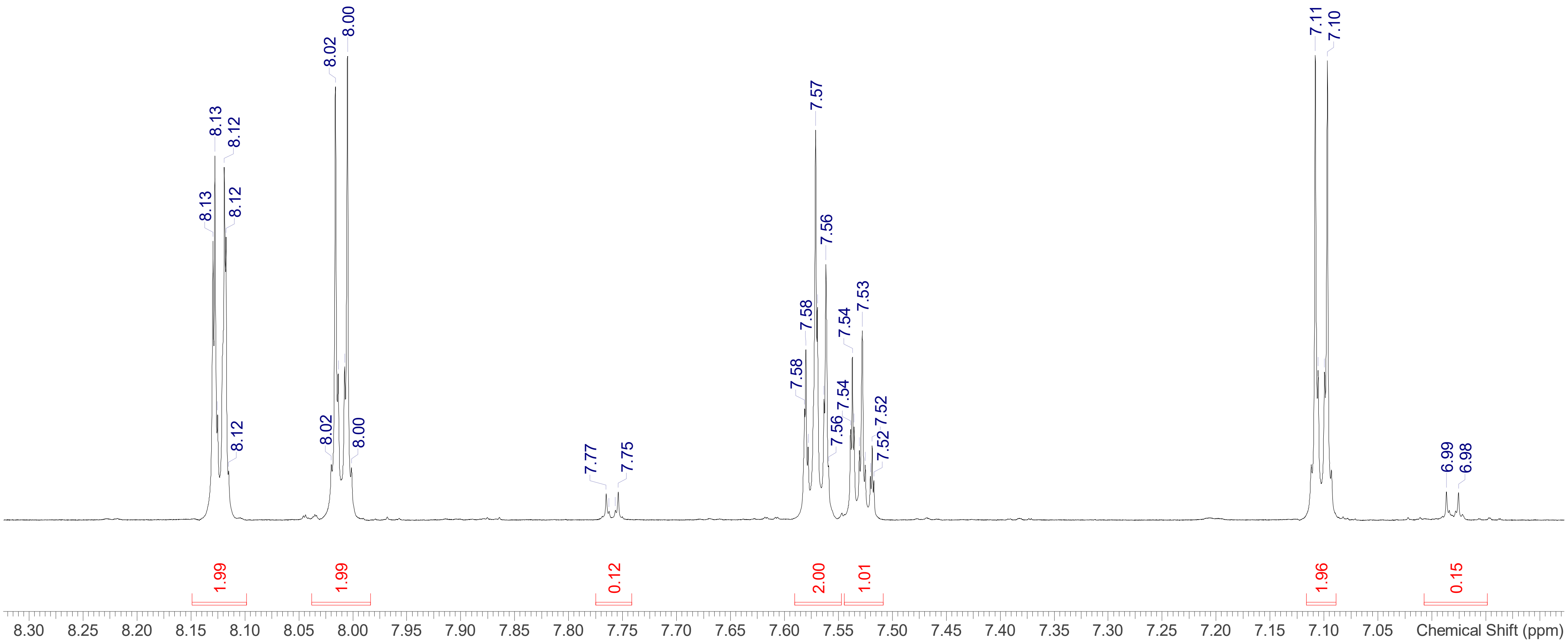

^{1}H NMR (799.7 MHz; DMSO-d_6)

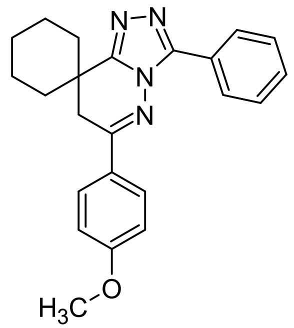

10a

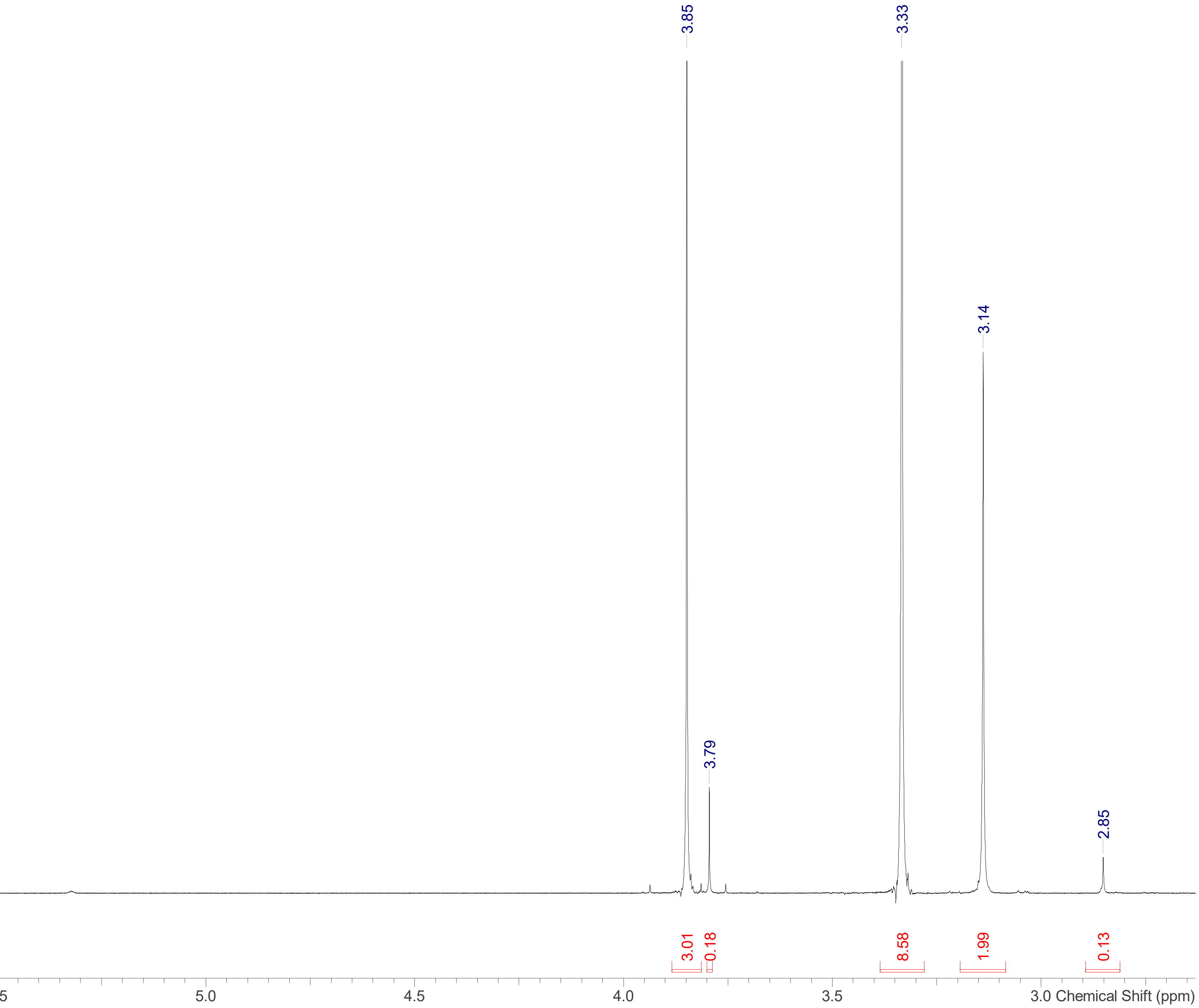

^{1}H NMR (799.7 MHz; DMSO-d_6)

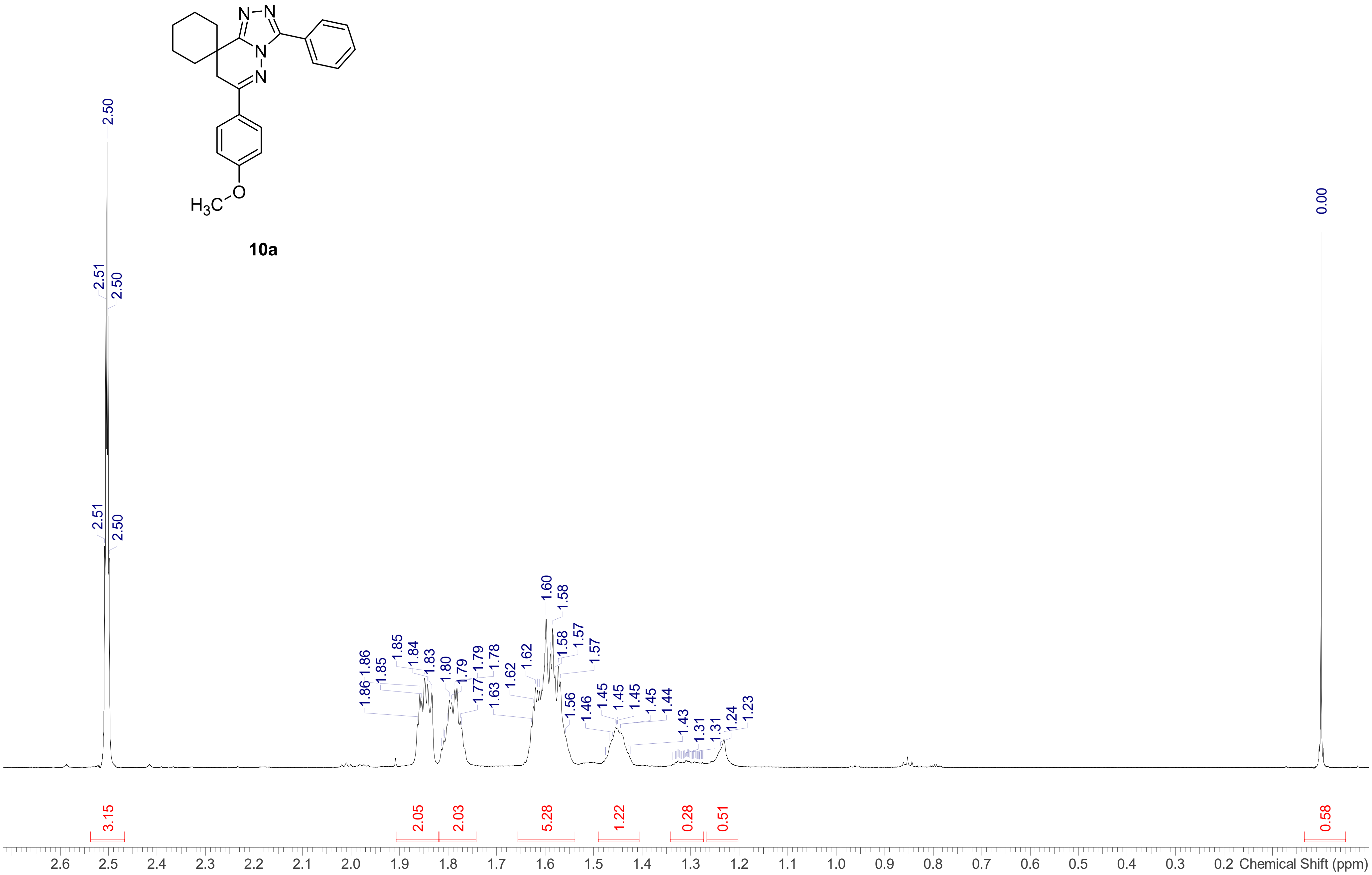

^{13}C NMR (201.1 MHz; DMSO-d_6)

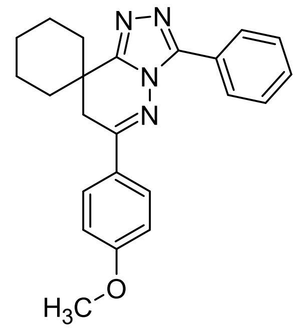

10a

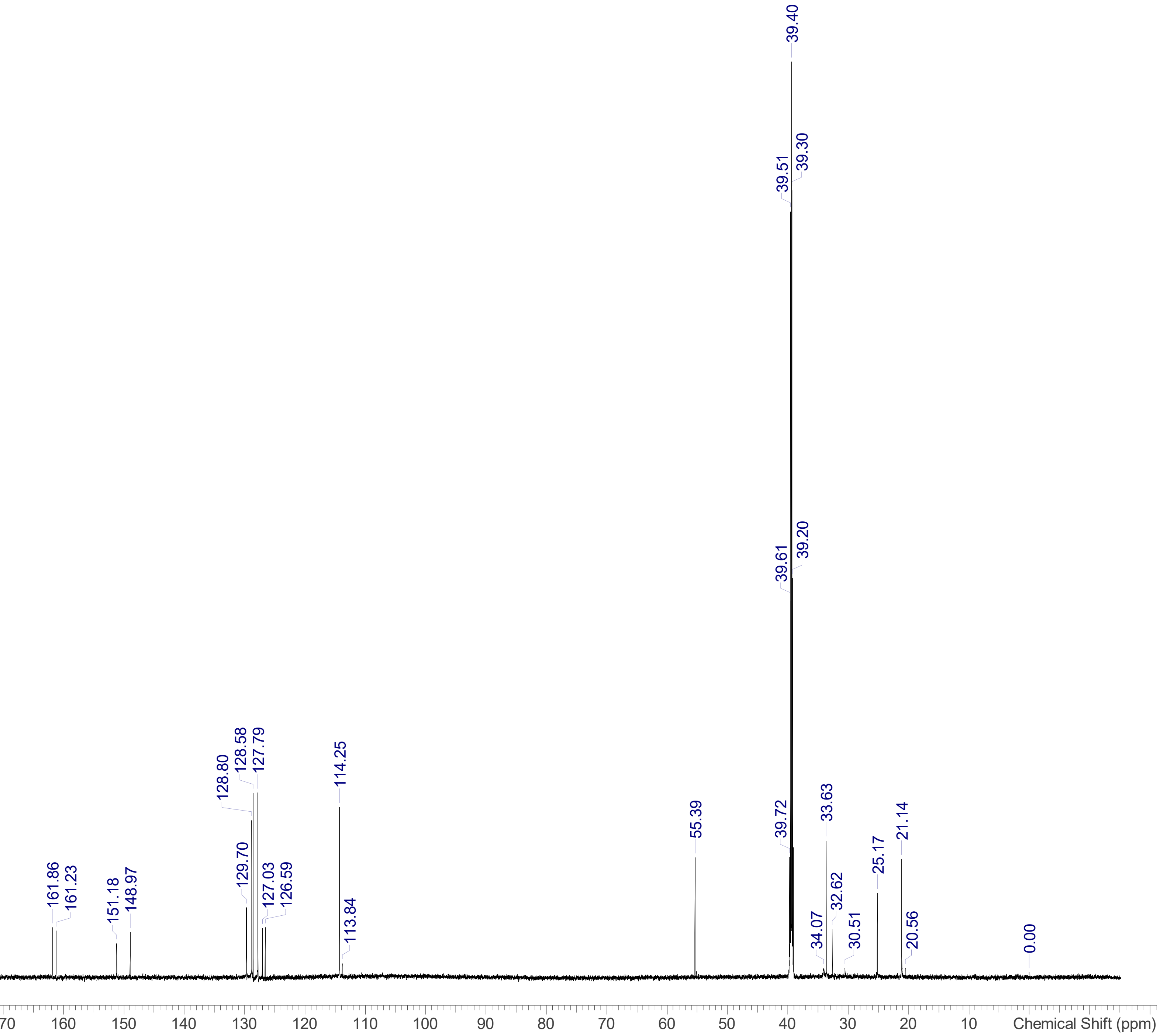

^{13}C NMR (201.1 MHz; DMSO-d_6)

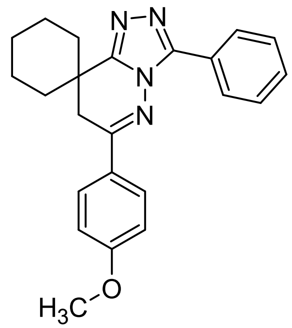

10a

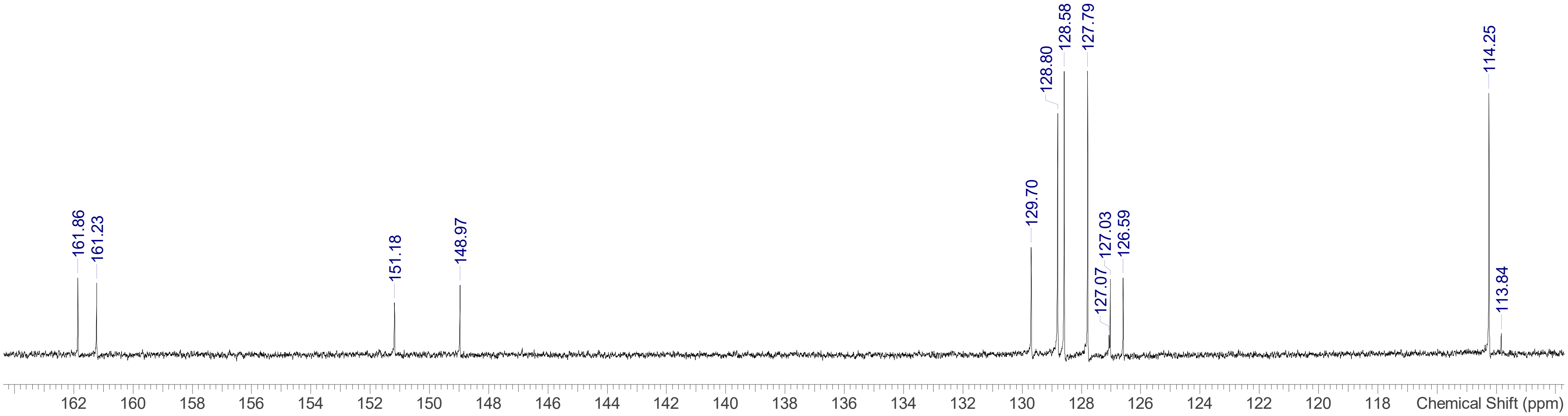

^{13}C NMR (201.1 MHz; DMSO-d_6)

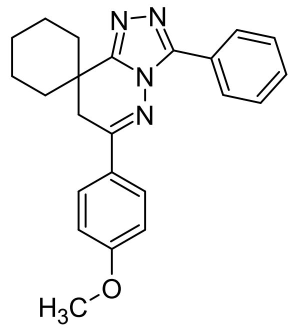

10a

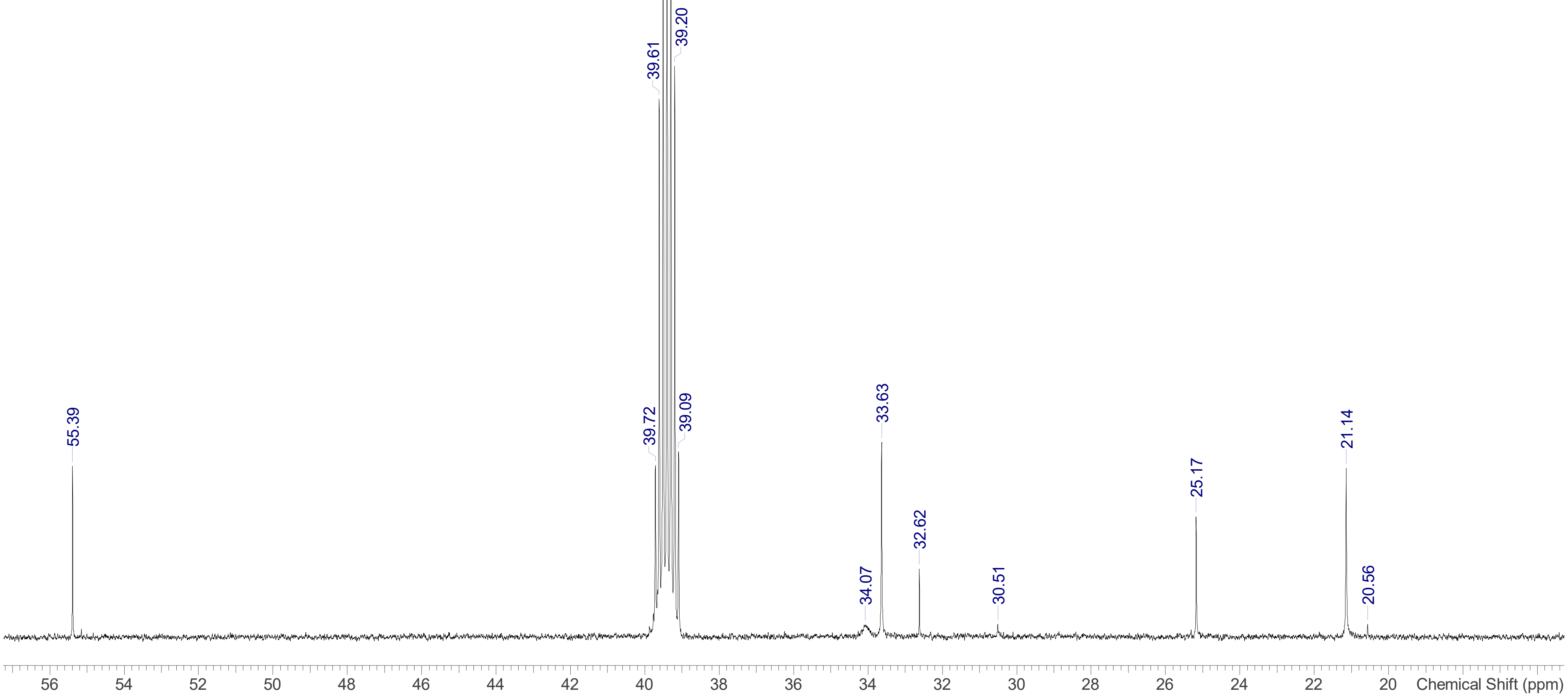

Compound **10a**

HRMS spectrum (ESI)

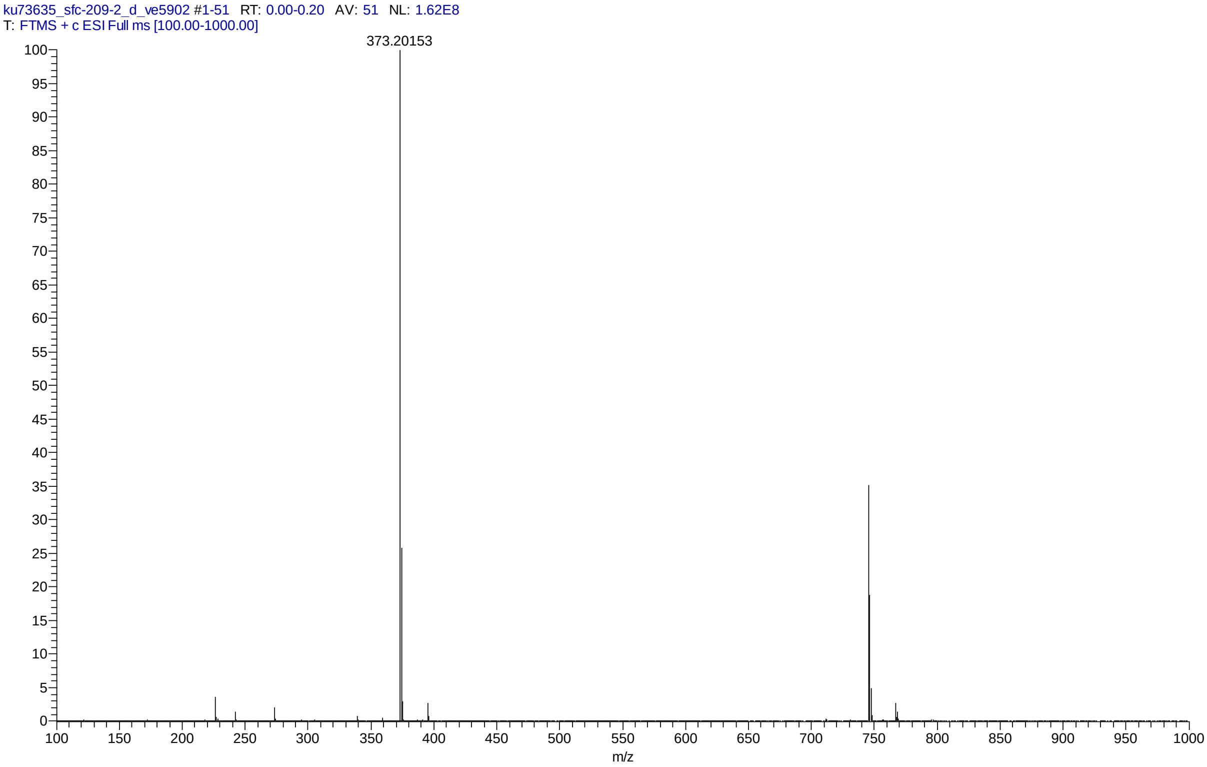

HRMS spectrum (ESI daughter ion)

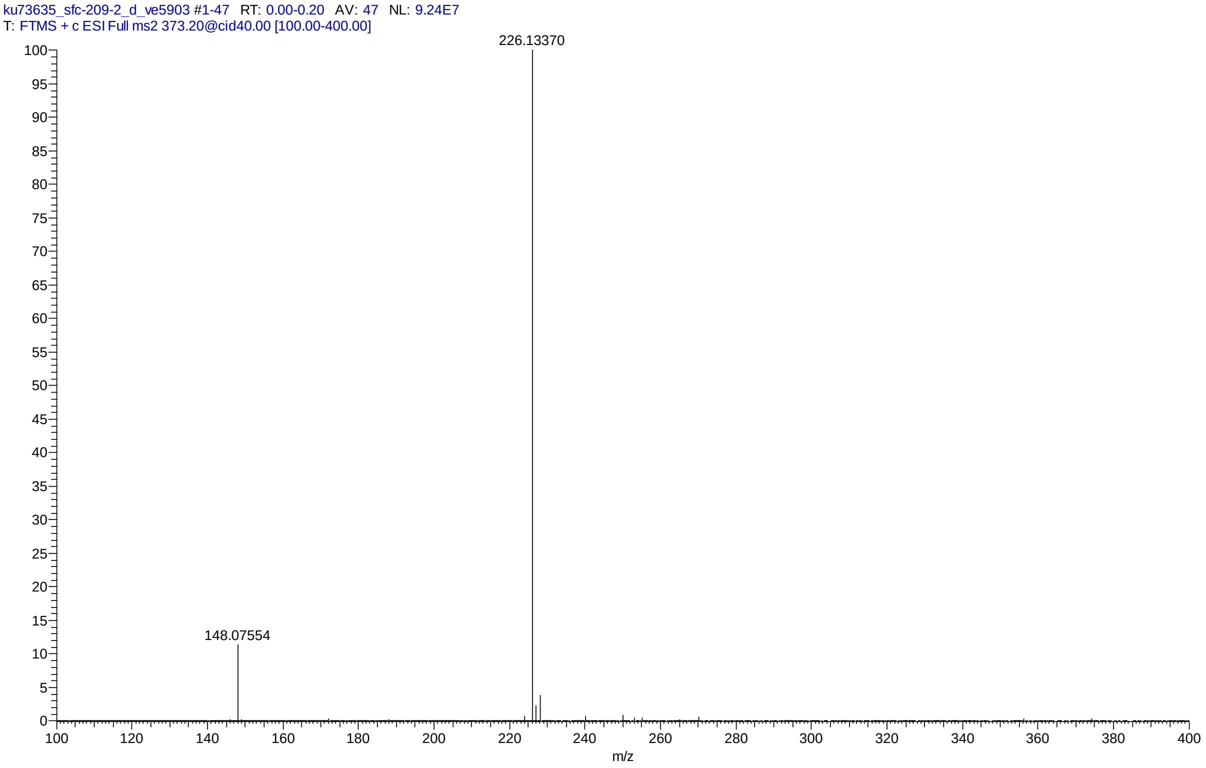

^{1}H NMR (499.9 MHz; DMSO-d_6)

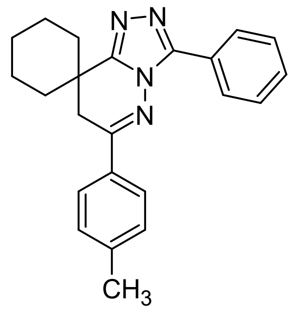

10b

3-Phenyl-6-(*p*-tolyl)-7*H*-spiro[[1,2,4]triazolo[4,3-*b*]pyridazine-8,1'-cyclohexane]

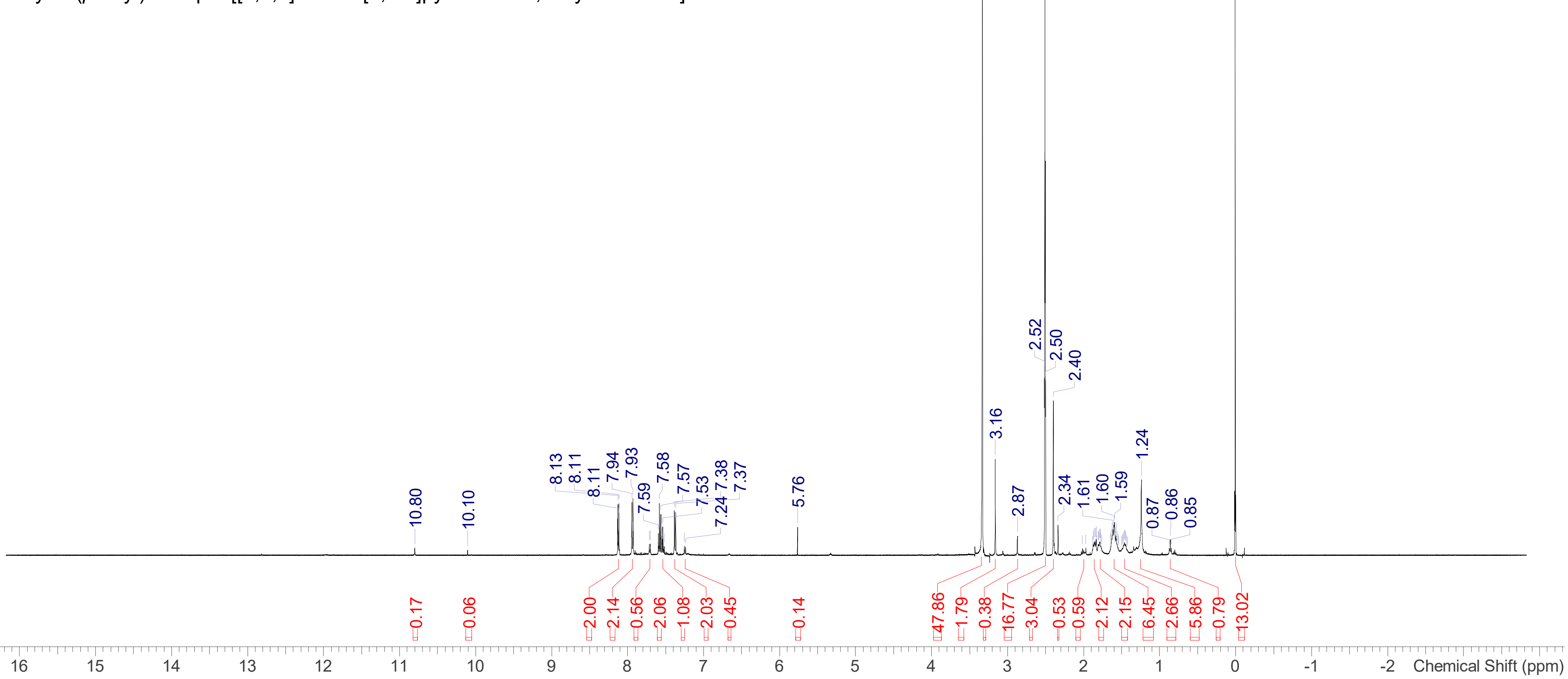

^{1}H NMR (499.9 MHz; DMSO-d_6)

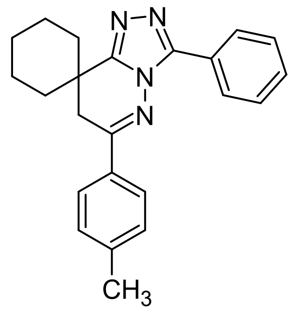

10b

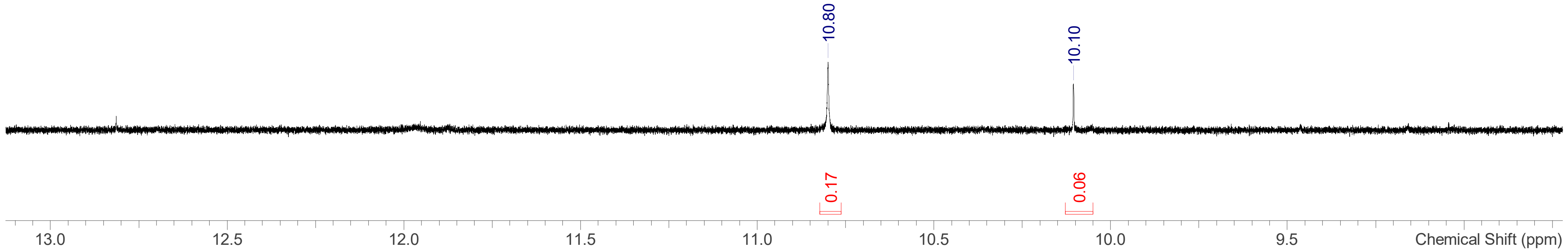

^{1}H NMR (499.9 MHz; DMSO-d_6)

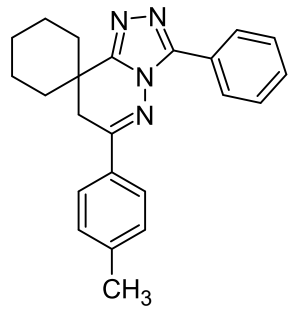

10b

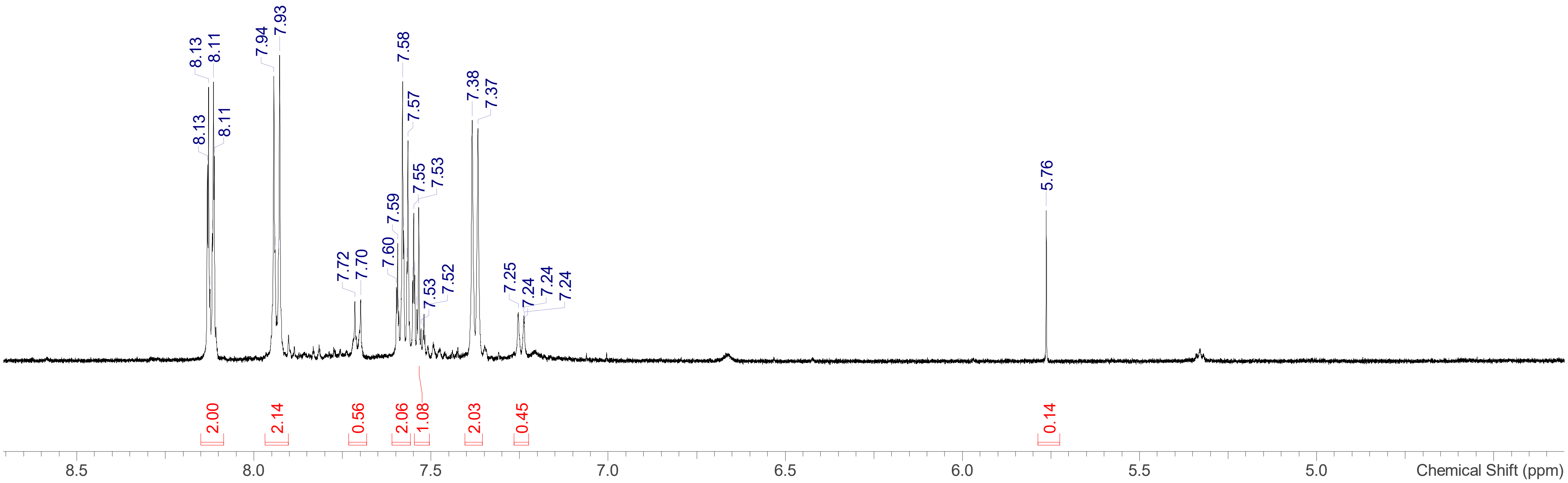

^{1}H NMR (499.9 MHz; DMSO-d_6)

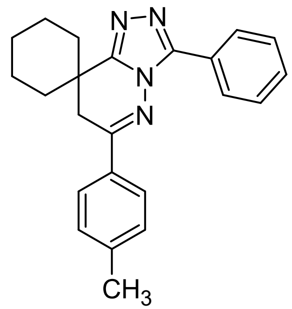

10b

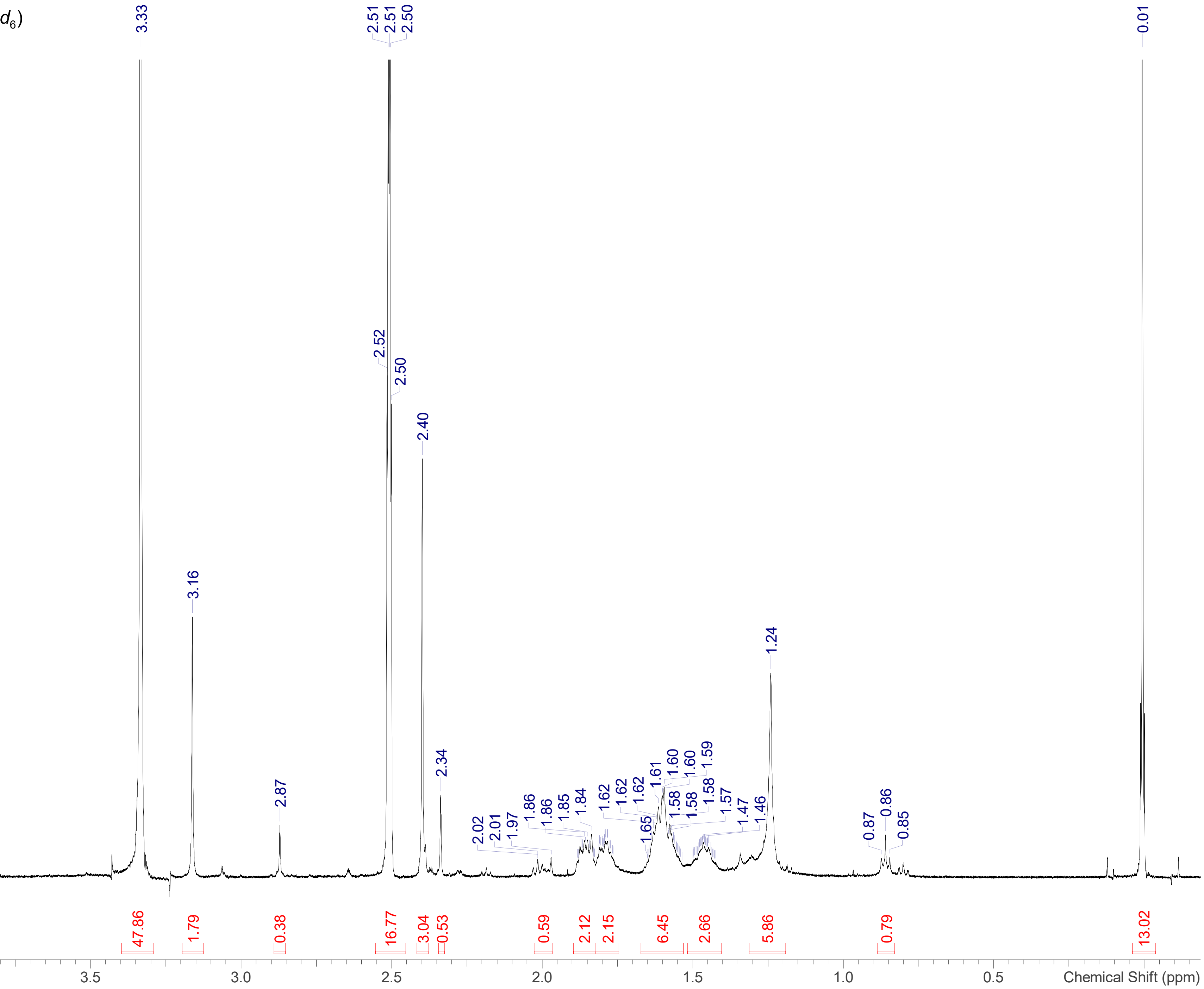

^{13}C NMR (125.7 MHz; DMSO-d_6)

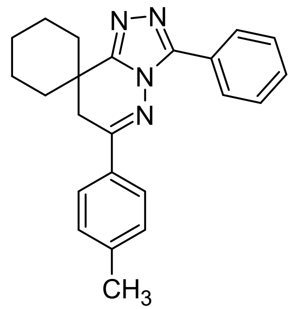

10b

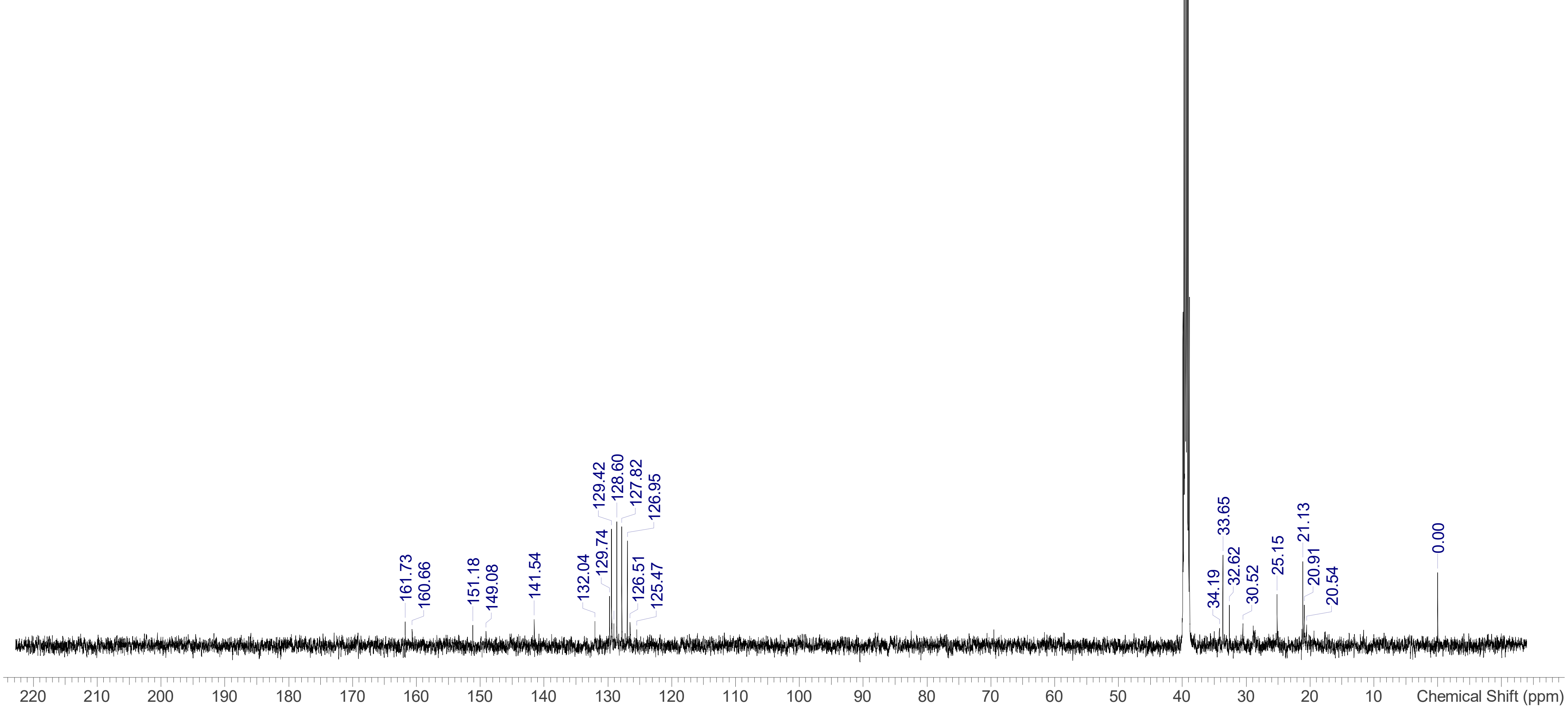

^{13}C NMR (125.7 MHz; DMSO-d_6)

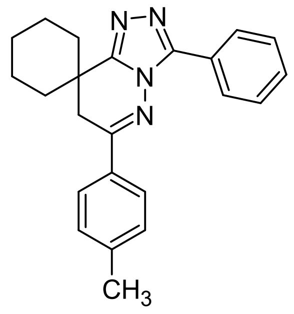

10b

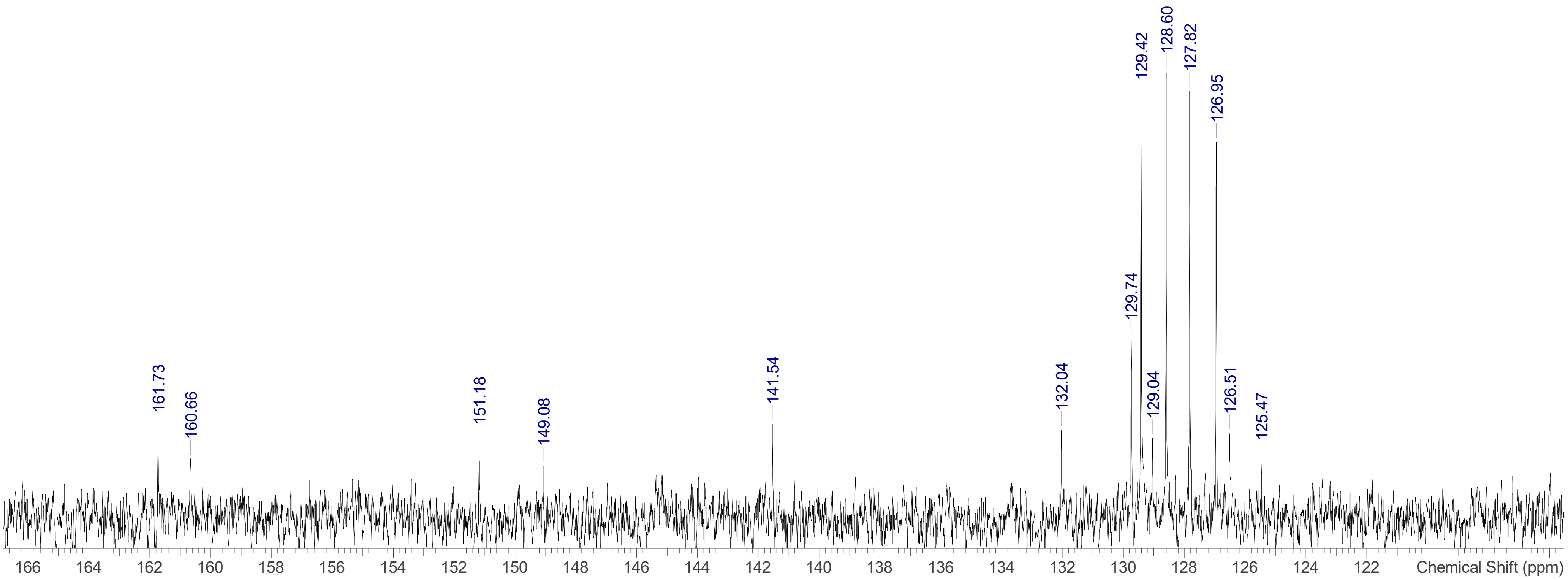

^{13}C NMR (125.7 MHz; DMSO-d_6)

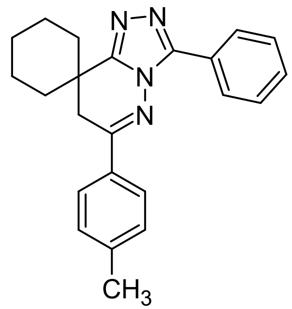

10b

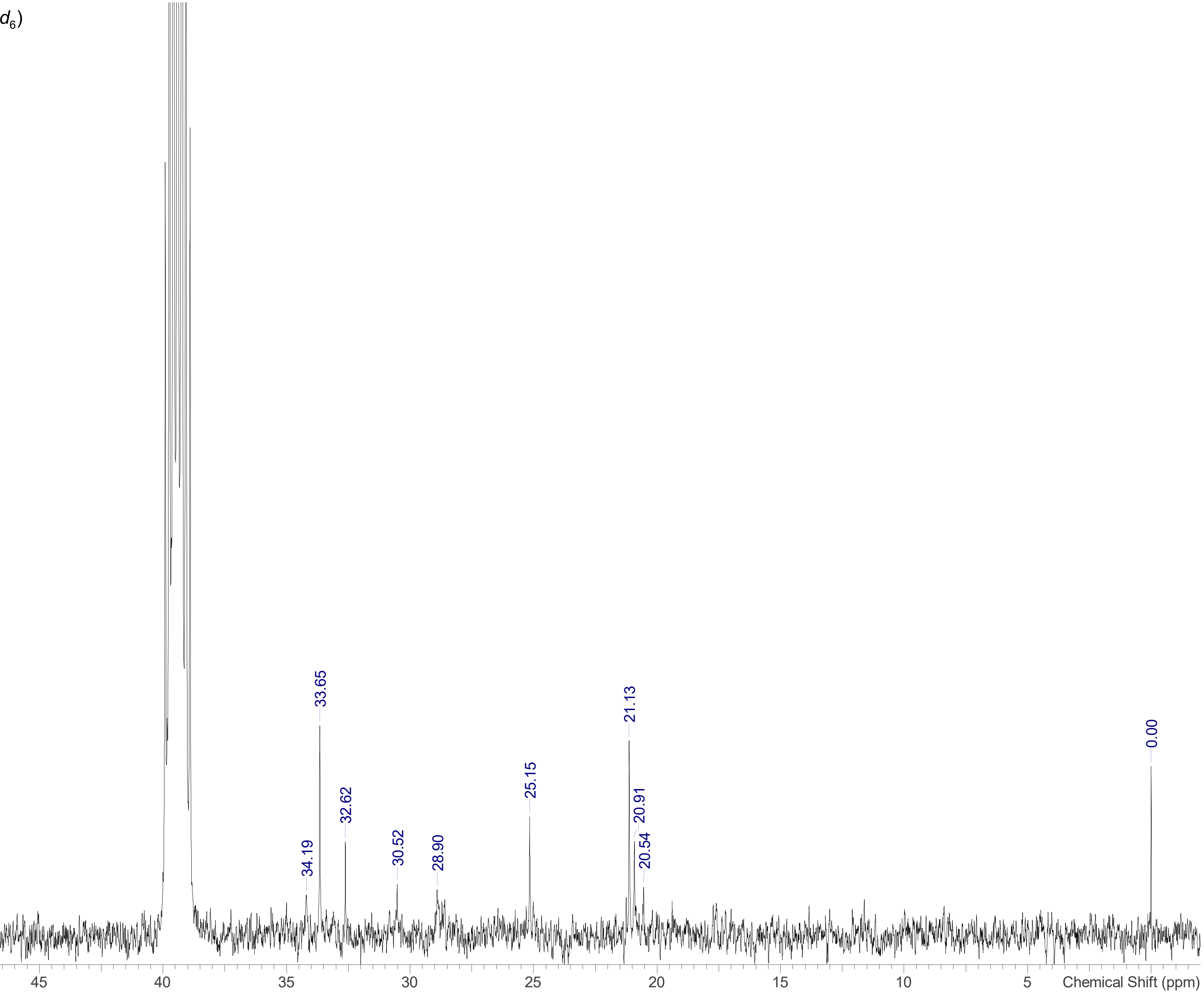

Compound **10b**

HRMS spectrum (ESI)

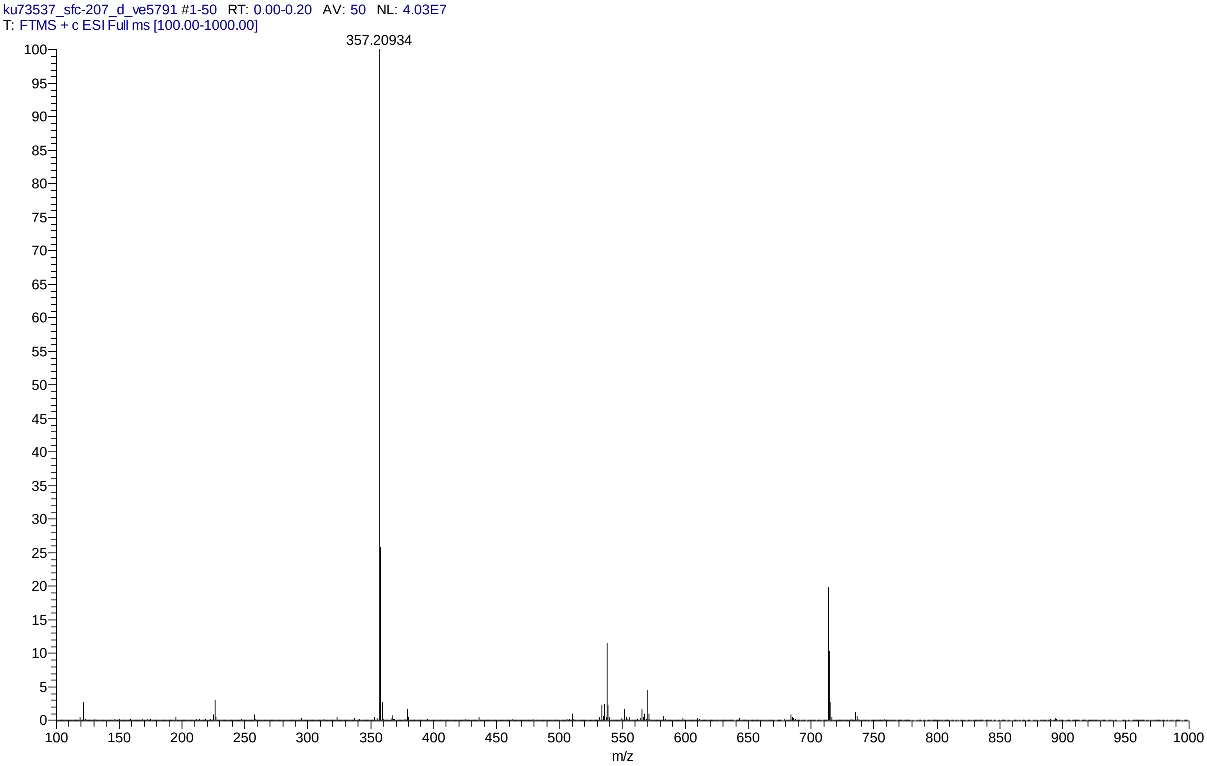

HRMS spectrum (ESI daughter ion)

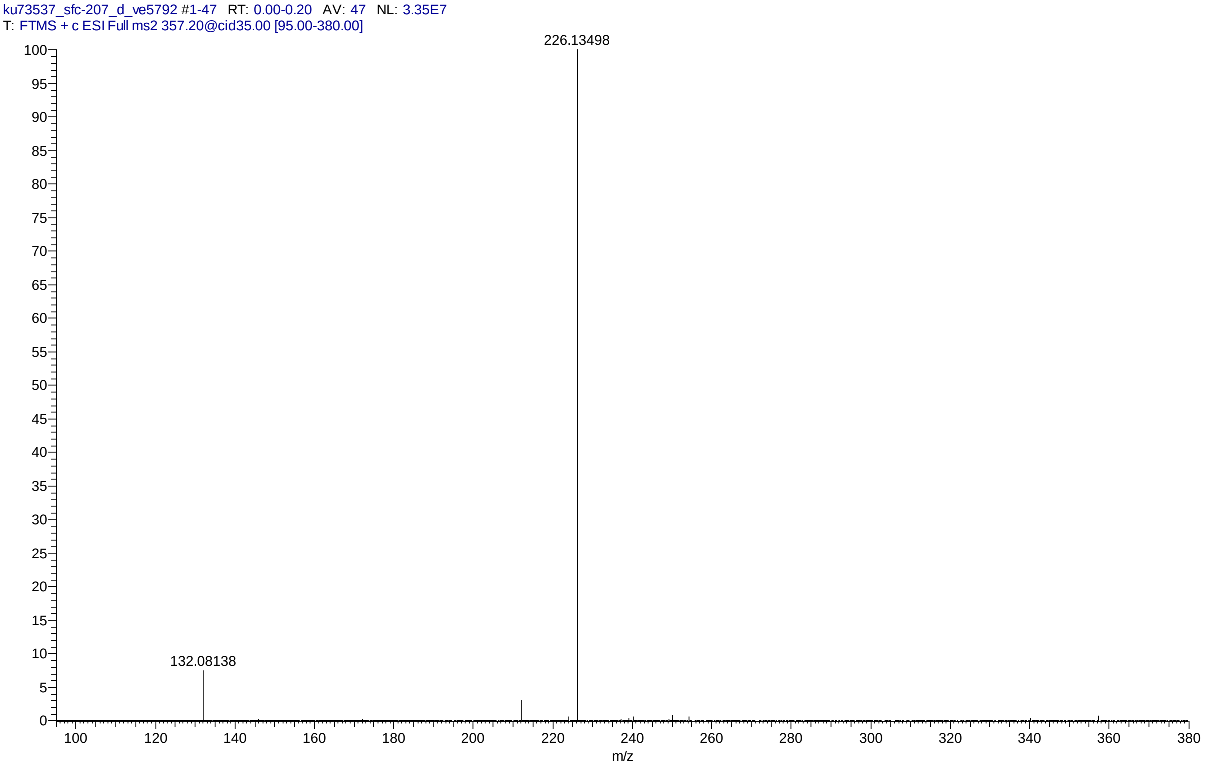

^{1}H NMR (499.9 MHz; DMSO-d_6)

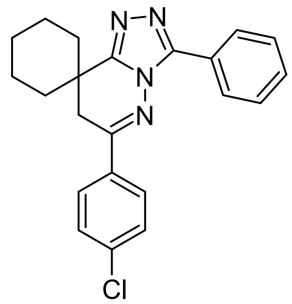

10c

6-(4-Chlorophenyl)-3-phenyl-7*H*-spiro[[1,2,4]triazolo[4,3-*b*]pyridazine-8,1'-cyclohexane]

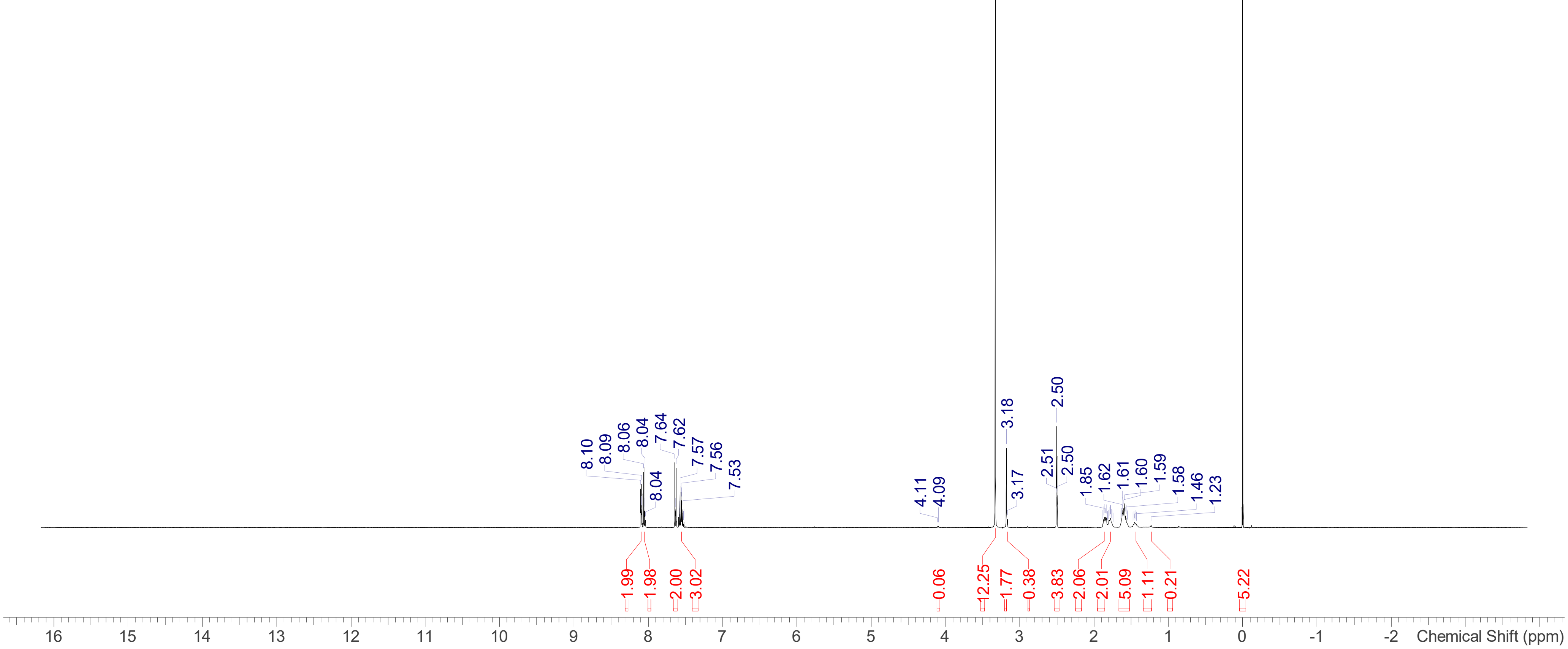

^{1}H NMR (499.9 MHz; DMSO-d_6)

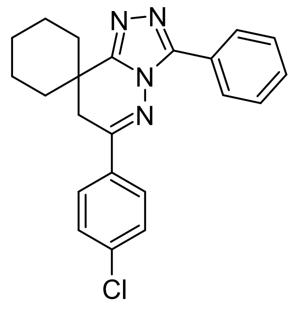

10c

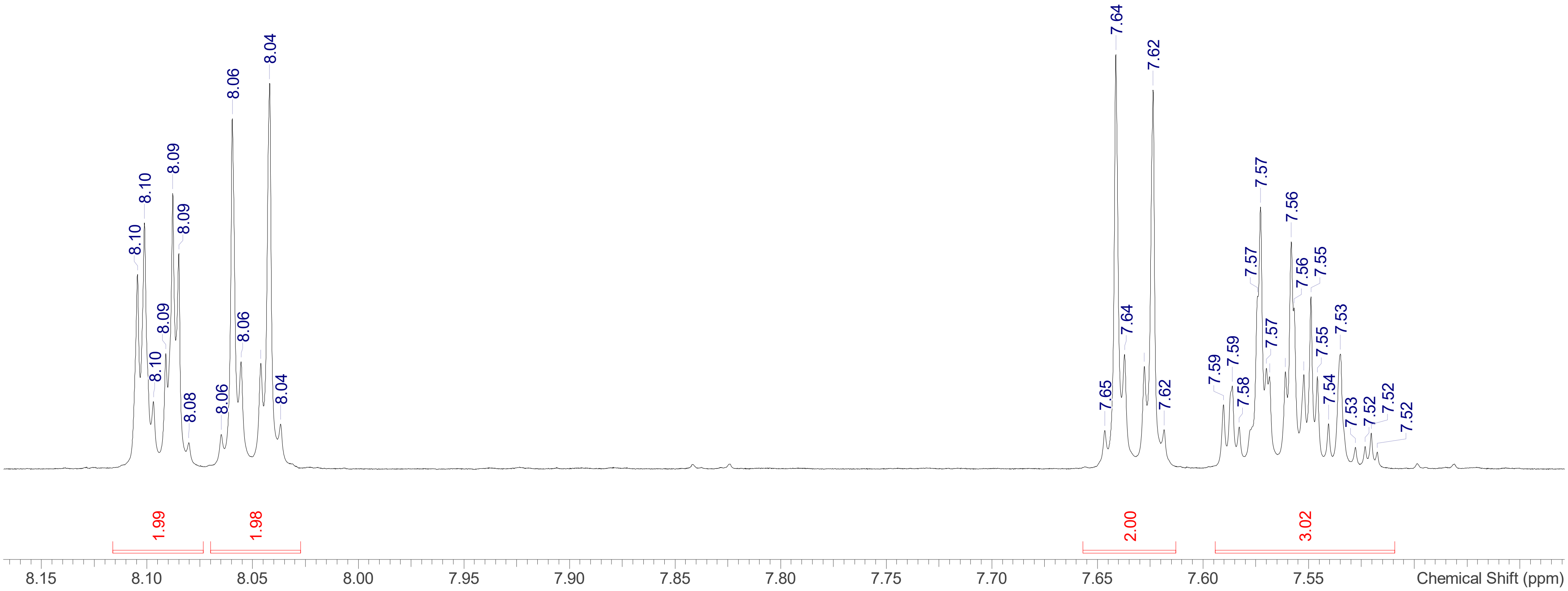

^1H NMR (499.9 MHz; DMSO-d_6)

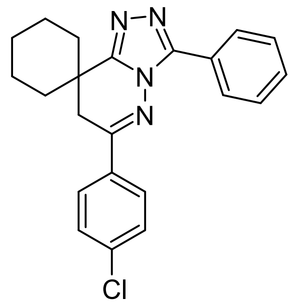

10c

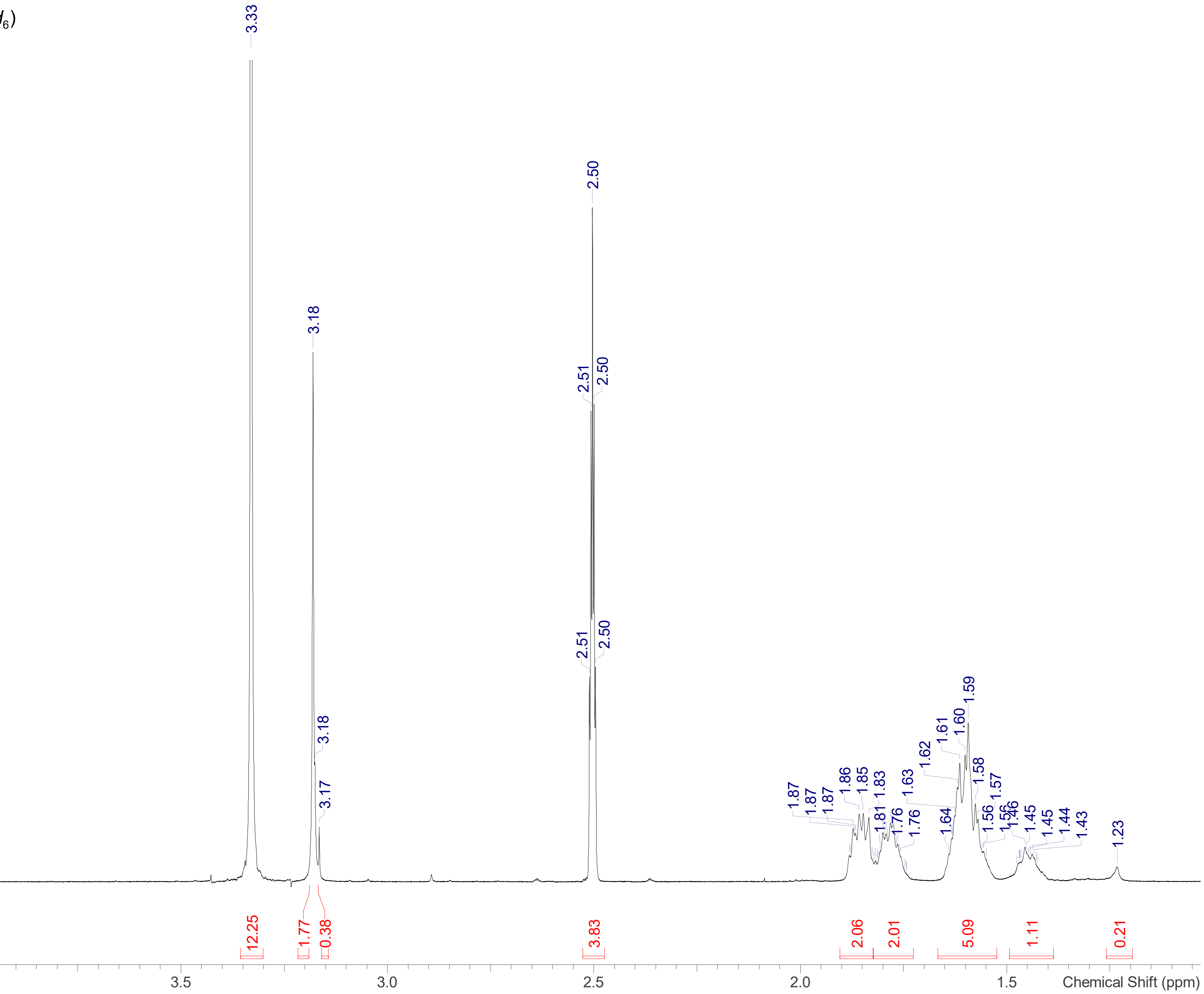

^{13}C NMR (125.7 MHz; DMSO-d_6)

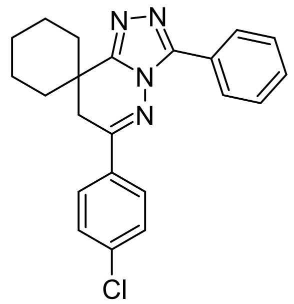

10c

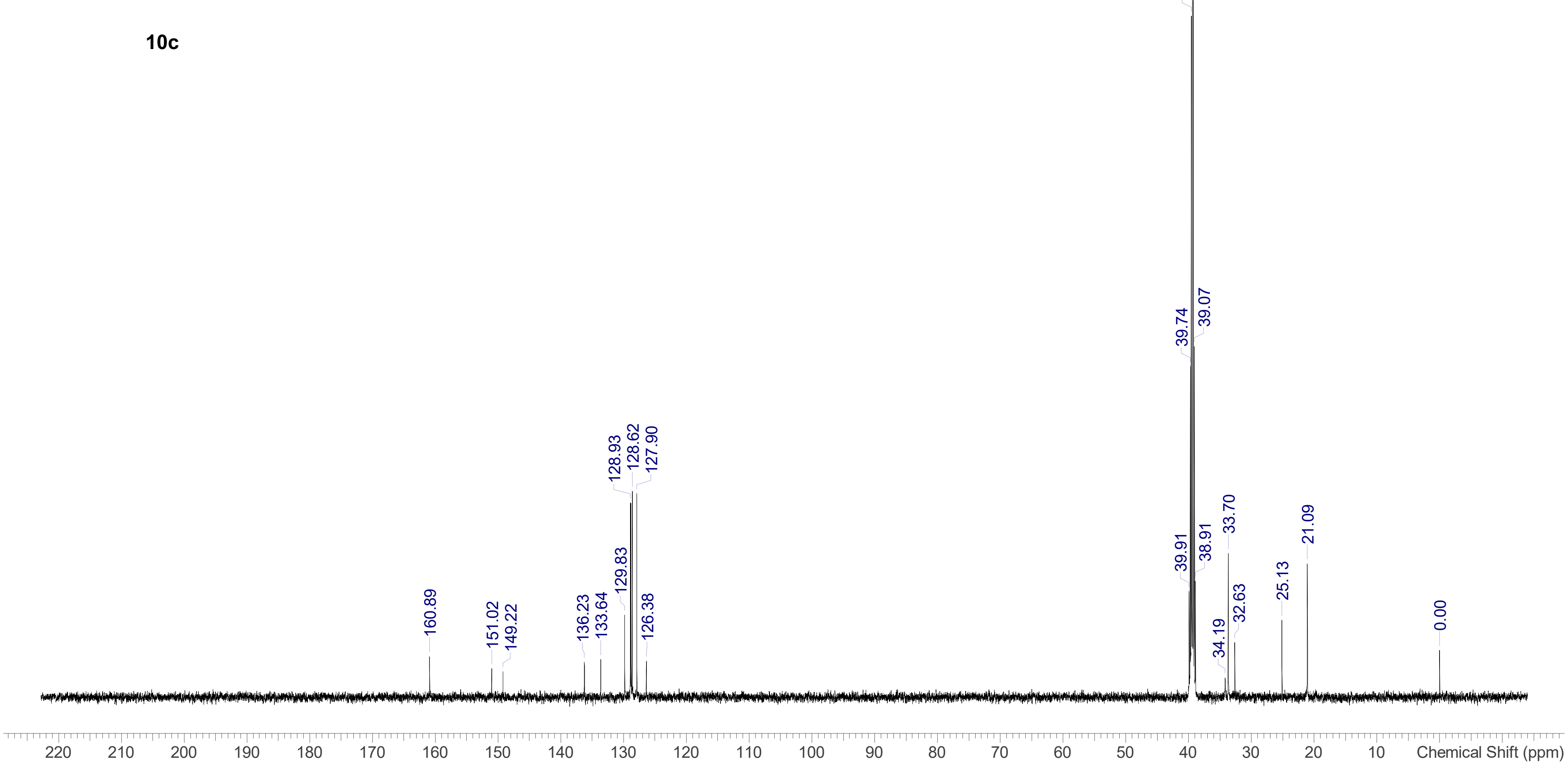

^{13}C NMR (125.7 MHz; DMSO-d_6)

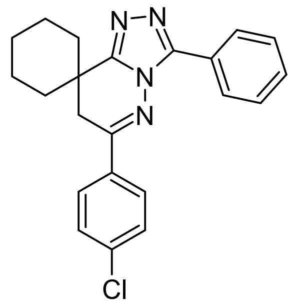

10c

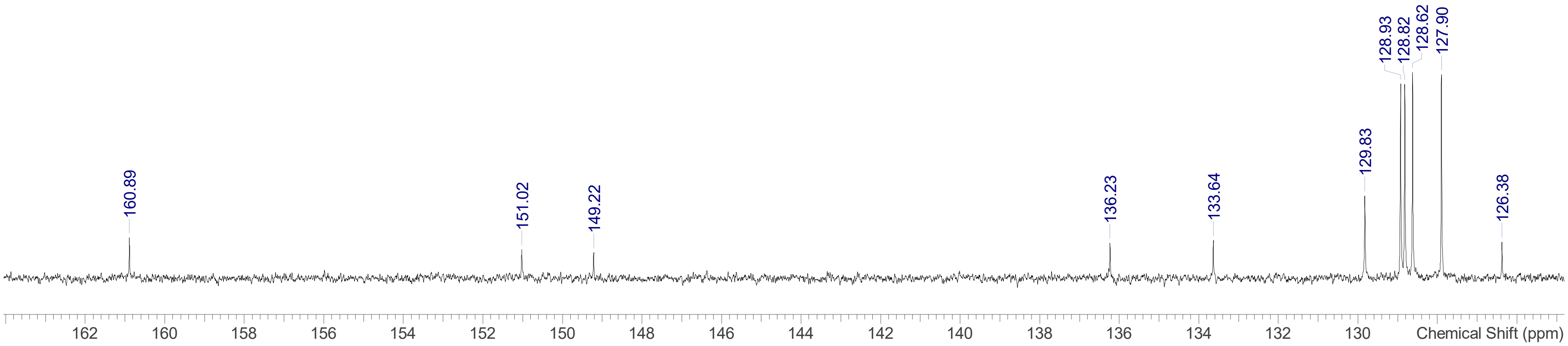

^{13}C NMR (125.7 MHz; DMSO-d_6)

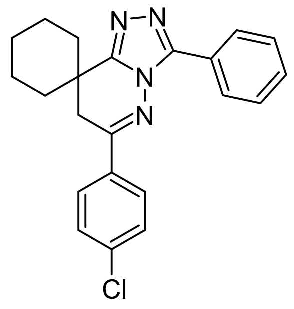

10c

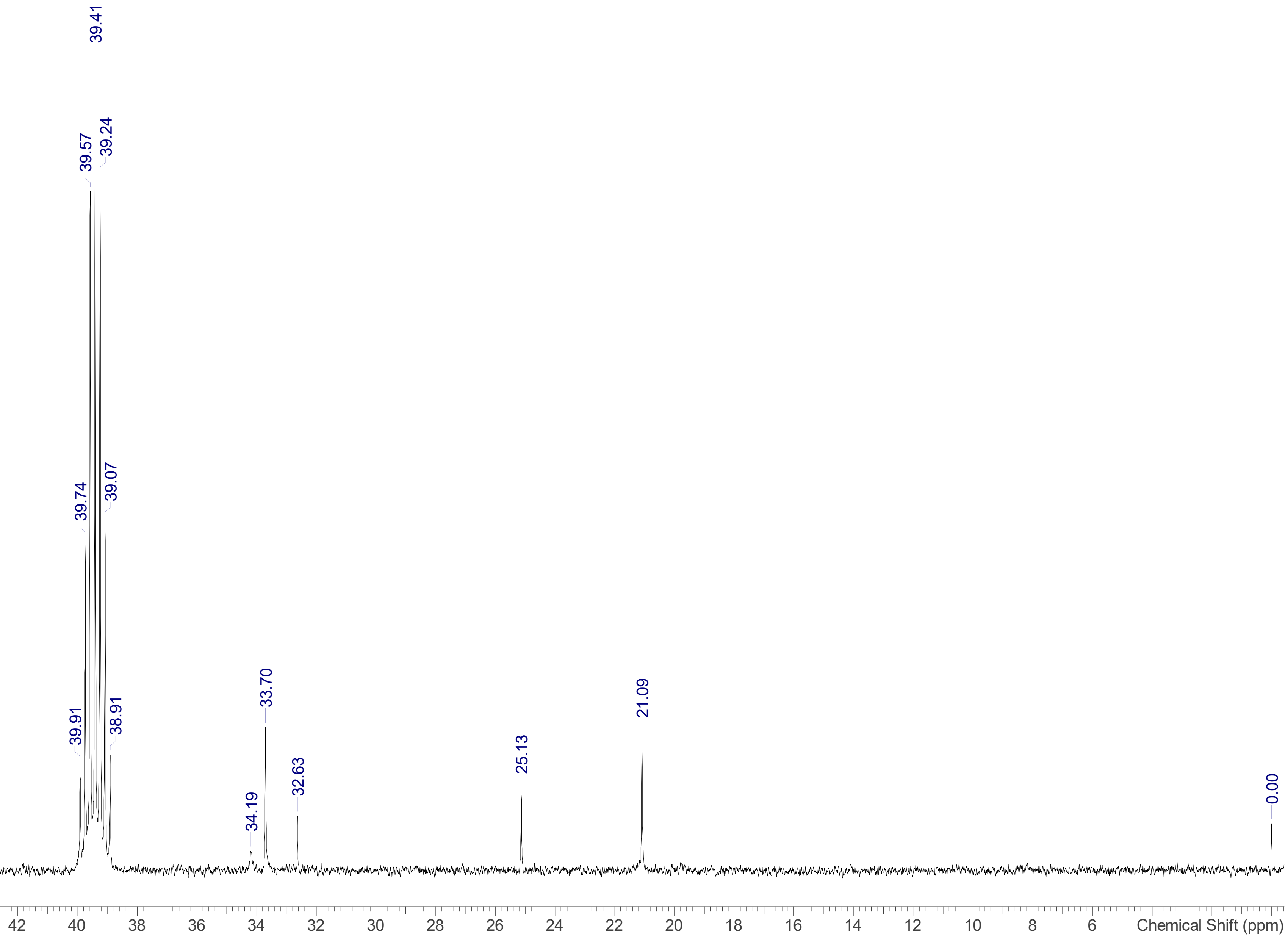

Compound **10c**

HRMS spectrum (ESI)

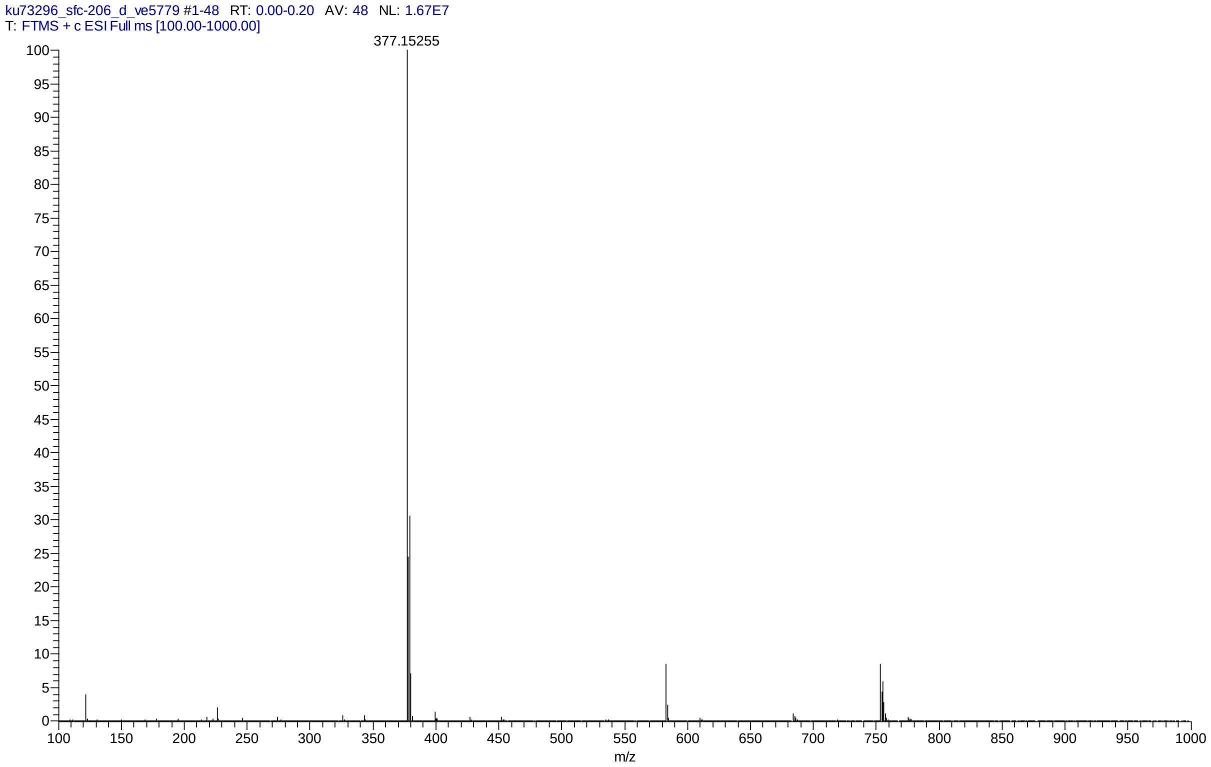

HRMS spectrum (ESI daughter ion)

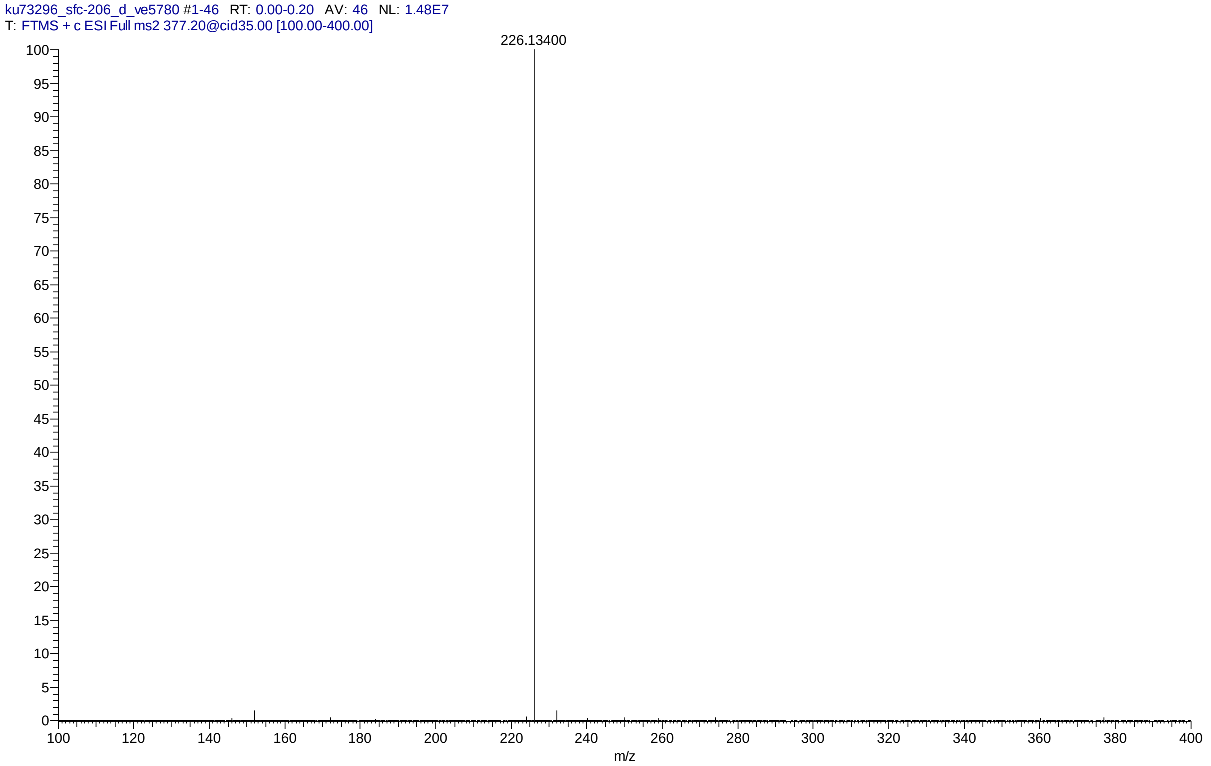

Supplement: Supplementary file 1 [file molecules-26-02140-s001.pdf]
